# e-Sweet: A Machine-Learning Based Platform for the Prediction of Sweetener and Its Relative Sweetness

**DOI:** 10.3389/fchem.2019.00035

**Published:** 2019-01-30

**Authors:** Suqing Zheng, Wenping Chang, Wenxin Xu, Yong Xu, Fu Lin

**Affiliations:** ^1^School of Pharmaceutical Sciences, Wenzhou Medical University, Wenzhou, China; ^2^Chemical Biology Research Center, Wenzhou Medical University, Wenzhou, China; ^3^Center of Chemical Biology, Guangzhou Institutes of Biomedicine and Health, Chinese Academy of Sciences, Guangzhou, China

**Keywords:** sweet taste, sweetener prediction, relative sweetness prediction, machine learning method, QSAR

## Abstract

Artificial sweeteners (AS) can elicit the strong sweet sensation with the low or zero calorie, and are widely used to replace the nutritive sugar in the food and beverage industry. However, the safety issue of current AS is still controversial. Thus, it is imperative to develop more safe and potent AS. Due to the costly and laborious experimental-screening of AS, *in-silico* sweetener/sweetness prediction could provide a good avenue to identify the potential sweetener candidates before experiment. In this work, we curate the largest dataset of 530 sweeteners and 850 non-sweeteners, and collect the second largest dataset of 352 sweeteners with the relative sweetness (RS) from the literature. In light of these experimental datasets, we adopt five machine-learning methods and conformational-independent molecular fingerprints to derive the classification and regression models for the prediction of sweetener and its RS, respectively via the consensus strategy. Our best classification model achieves the 95% confidence intervals for the accuracy (0.91 ± 0.01), precision (0.90 ± 0.01), specificity (0.94 ± 0.01), sensitivity (0.86 ± 0.01), F1-score (0.88 ± 0.01), and NER (Non-error Rate: 0.90 ± 0.01) on the test set, which outperforms the model (NER = 0.85) of Rojas et al. in terms of NER, and our best regression model gives the 95% confidence intervals for the R^2^(test set) and ΔR^2^ [referring to |R^2^(test set)- R^2^(cross-validation)|] of 0.77 ± 0.01 and 0.03 ± 0.01, respectively, which is also better than the other works based on the conformation-independent 2D descriptors (e.g., 2D Dragon) according to R^2^(test set) and ΔR^2^. Our models are obtained by averaging over nineteen data-splitting schemes, and fully comply with the guidelines of Organization for Economic Cooperation and Development (OECD), which are not completely followed by the previous relevant works that are all on the basis of only one random data-splitting scheme for the cross-validation set and test set. Finally, we develop a user-friendly platform “e-Sweet” for the automatic prediction of sweetener and its corresponding RS. To our best knowledge, it is a first and free platform that can enable the experimental food scientists to exploit the current machine-learning methods to boost the discovery of more AS with the low or zero calorie content.

## Introduction

Sweet taste, eliciting a pleasant sensation, provides an instinctive means to find the energy source such as the carbohydrates, which usually taste sweet. The taste perception of the sweetness is a complex mechanism involving the multiple disciplines (e.g., chemistry, biology, and physiology), however, it is generally assumed to be predominantly mediated by the taste receptors type 1 (Tas1Rs) on the taste buds in the oral cavity (Roper and Chaudhari, 2017). Interestingly, Tas1Rs are also expressed in numerous different organs (e.g., gut and pancreas), implicating that they are intricately participated in various physiological processes such as intestinal absorption, glucose homeostasis, and metabolic regulation (Laffitte et al., 2014).

Human sweet taste receptor (*h*STR) functions as a heterodimer of two subunits (*h*Tas1R2 and *h*Tas1R3) belonging to the class C family of G-protein coupled receptors (GPCRs), whereas each subunit contains three distinct domains: a large extracellular venus flytrap domain (VFD), a short cysteine-rich domain (CRD), and seven-transmembrane domain (TMD) (Meyers and Brewer, 2008). *h*STR harbors at least four different binding sites revealed by the biochemical characterization such as the chimeras or site-directed mutagenesis experiment, and thereby can recognize a variety of sweeteners (Masuda et al., 2012): sugars (e.g., sucrose and glucose), amino acids (e.g., D-trypotophan and D-glycine), artificial sweeteners (e.g., saccharin and aspartame), and sweet proteins (e.g., monellin and thaumatin). According to the content of calorie, these chemically diverse sweeteners can be generally categorized into two types (Dubois and Prakash, 2012): the nutritive sweeteners with the high calorie (e.g., sucrose), and the non-nutritive sweeteners (e.g., saccharin and aspartame) with the low or zero calorie that mainly refer to the artificial sweeteners in this work.

Nowadays, the non-nutritive sweeteners are broadly used as the food additives to substitute for the nutritive sweeteners such as sucrose, since the over-consumption of high-calorie nutritive sweeteners in the functional food and beverage will lead to the elevated risks of the metabolic disorders (e.g., type II diabetes) and cardiovascular diseases (Fernstrom, 2015). Therefore, a multitude of non-nutritive sweeteners with the low calorie yet preserving the sweetness have been manually synthesized or directly extracted from the natural plants to prevent these risks.

Hitherto, none of the currently available non-nutritive sweeteners (especially the artificial sweeteners) can accurately replicate the same sweetness profile (e.g., concentration/response function, temporal profile, and adaption behaviors) of the natural sucrose (Dubois, 2016), since they usually exhibit the slow sweetness onset, lingering sweetness aftertaste, apparent off-taste, or moderate/strong adaption upon the iterative tasting, which are generally not preferred by most of consumers. Moreover, the heavy use of the artificial sweeteners, one major class of non-nutritive sweeteners, are reported to cause some side-effects such as an increased risk of cancer in human (Mishra et al., 2015). Therefore, it is still desirable to discover more novel and safe non-nutritive sweeteners.

As we know, the sweetener discovery using the human taste-panel or cell-based high-throughput screening is an expensive, laborious and slow process. Hence *in-silico* sweetener prediction could be a good alternative to rapidly identify the most likely sweetener candidates with the high potency prior to the time-consuming and arduous experiment. Currently, there are two main computational methods for the sweetener prediction: structure-based and ligand-based methods. Structure-based method is to rationally design the compound based on *h*STR. Nevertheless, the crystal structure of full *h*STR is still unraveled, albeit there are several homology models based on the templates with the limited sequence identities (Shrivastav and Srivastava, 2013; Jean-Baptiste et al., 2017; Kim et al., 2017; Acevedo et al., 2018). In addition, a compound that can bind with *h*STR could be also a sweetness inhibitor (e.g., lactisole) (Jiang et al., 2005), rather than the sweetener of our interest. However, the data-driven machine-learning method, emerging as a vibrant area of ligand-based method, can directly predict the sweetener and its relative sweetness (RS), provided that there is sufficient experimental dataset to build the predictive model. More specifically, the sweetener/non-sweetener classification models based on the machine-learning methods can be employed to predict the sweetener, and the regression models derived from the machine-learning methods can be utilized to forecast the RS of the sweetener.

Rojas et al. comprehensively review the sweet/bitter (Rojas et al., 2016a; Banerjee and Preissner, 2018), sweet/tasteless (Rojas et al., 2016a), and sweet/sweetless (Rojas et al., 2017) classification models, and also provide a systematic overview on the regression models for the RS prediction of sweetener (Rojas et al., 2016a,b,c). In our study, only the typical works about the sweet/sweetless classification model on the relatively large dataset are briefly summarized here, because the sweet/sweetless pair is more reasonable and practical for the sweetener prediction due to the inclusion of bitter and tasteless compounds into the sweetless dataset. Meanwhile, only the representative works regarding to the regression model also on the basis of the comparatively large dataset will be shortly recapitulated in our study, while the pioneering works of the sweeteners prediction model based on the congeneric systems or small dataset, contributing significantly to the subsequent works in this research area, have been thoroughly summarized in Rojas et al. (2016b) and thereby will be not reviewed here due to the limited space. It should be noted that only the works about the classification and regression models on the basis of the comparatively large dataset will be shortly reviewed in this study and the relatively large dataset here refers to the dataset with at least two hundreds samples, since the relatively large dataset affords the more extended applicability-domain of model.

As for the classification model, Rojas et al. develop the sweet/sweetless classification model based on the relatively large dataset (649 compounds) consisting of 435 sweeteners and 214 non-sweeteners (133 tasteless and 81 bitterants). In their work, the partial least squares discriminant analysis (PLSDA) and K-nearest neighbors (KNN) coupled with the 2D Dragon descriptors (https://chm.kode-solutions.net/) are used to train the models, respectively, which are combined to form a consensus model. Their consensus model gives the sensitivity, specificity and NER (Non-Error Rate, the average of sensitivity and specificity in the binary classification) of 0.88, 0.82, and 0.85, respectively on the test set including 108 sweeteners and 53 non-sweeteners that are randomly selected from the whole dataset (Rojas et al., 2017). However, only 81 bitterants are adopted as the sweetless compounds in their work. Hence the numerous bitterants curated by BitterDB (Wiener et al., 2012) could be treated as the sweetless compounds to further leverage the applicability-domain of the sweeteners/non-sweeteners classification model.

Regarding to the regression model, Zhong et al. (2013) build the regression models based on the comparatively large dataset including the 320 sweeteners (214 for the training set and 106 for the hold-out test set) with RS. The regression models are trained with the multi-linear regression (MLR) and support vector machine (SVM), respectively in combination with the mixed 2D and 3D descriptors from ADRIANA.Code program (Molecular Networks GmbH, Erlangen, Germany). The MLR and SVM models give the R^2^ of 0.77 and 0.78, respectively on the test set consisting of 106 randomly selected sweeteners. Moreover, Goel et al. harness the genetic function approximation (GFA) and artificial neural network (ANN) coupled with the mixed 2D and 3D molecular descriptors (e.g., LUMO eigenvalue) from Material Studio v6.0 (MS6) (BIOVIA, San Diego, USA) to establish the regression model on the dataset of 455 sweeteners (319 for the training set and 136 for the hold-out test set), which is the largest so far. Both GFA and ANN models offer the impressive performance with the same R^2^ of 0.83 on the test set consisting of 136 randomly selected sweeteners (Goel et al., 2018). However, the conformation-dependent 3D descriptors are included in both works from Zhong et al. and Goel et al. and this will hamper the reproducibility of prediction result due to the versatile conformations for the same flexible compound, because most of the sweeteners are quite flexible. Moreover, some other potential issues introduced by the 3D descriptors have been discussed in the work of Rojas et al. (2016a).

Therefore, the conformation-independent 2D descriptors are advocated to be used in the prediction of RS, especially for the rapid and large-scale screening of potent sweeteners. Rojas et al. employ MLR and 2D Dragon descriptors to establish the regression model on the dataset of 233 sweeteners (163 for the training set and 70 for the hold-out test set). This model provides R^2^ of 0.70 on the test set including 70 sweeteners, which are selected by the K-mean cluster analysis (Rojas et al., 2016c). Ojha et al. utilize the partial least squares regression analysis (PLSRA) and 2D Dragon/PaDEL descriptors (Wei, 2011) to build the regression model on the dataset of 299 sweeteners (239 for the training set and 60 for the hold-out test set). This model achieves R^2^ of 0.75 on the test set composed of 60 randomly selected sweeteners (Ojha and Roy, 2017). Cheron et al. make full use of random forest (RF) and SVM methods combined with the 2D and 3D Dragon descriptors, respectively to construct the regression model on the dataset of 225 sweeteners (134 for the training set and 91 for the hold-out test set). The RF-2D, SVM-2D, RF-3D, and SVM-3D models offer R^2^ of 0.74, 0.83, 0.76, and 0.85, respectively on the test set comprising of 91 randomly chosen sweeteners. Nevertheless, their models may be prone to the over-fitting or under-fitting, since the respective model performances on the training set and test set differ significantly, which can be observed from R^2^ of 0.96, 0.69, 0.98, and 0.69 for RF-2D, SVM-2D, RF-3D, and SVM-3D models, respectively on the training set (Chéron et al., 2017). Thus, the performance evaluation by only R^2^(test set) is probably not enough.

In spite of the individual merits and pitfalls in each work, there are several common concerns in the aforementioned works about the classification and regression models. Firstly, only one data-splitting scheme for the training set and hold-out test set is used in those works, which may lead to the biased performance of the models. Thus, model would be more robust if it can be trained on the multiple data-splitting schemes to alleviate the bias from the single random data-splitting. Secondly, all these works fail to fully comply with the guidelines of Organization for Economic Cooperation and Development (OECD), since most of works are short of either Y-randomization test to evaluate the robustness of their models, or the clear and pragmatic definition for the domain-applicability of their models. Thirdly, all the works do not provide any convenient and practical programs for the users to predict the sweeteners and their RS, which will greatly restrict the application of their models. At last, all these works adopt PLSDA, PLSRA, MLR, KNN, SVM, RF, GFA, or ANN method, while the current state-of-the-art machine-learning methods such as Deep Neuron Network (DNN) and Gradient Boosting Machine (GBM), which often demonstrate the encouraging performance in the Kaggle competitions, were never exploited in the prediction of sweetener or RS before. Therefore, it is highly desirable to overcome these issues and develop a convenient and comprehensive software for the experimental food scientists to predict the sweetener and its corresponding RS.

In order to tentatively address the problems as mentioned above, we plan to build the informative models for the prediction of sweetener and its RS, which will be systematically derived with diverse machine-learning methods (KNN, SVM, GBM, RF, and DNN) and conformation-independent 2D molecular fingerprints based on the multiple data-splitting schemes and will be completely in accordance with the guidelines of OECD. For the convenience of the experimental food scientists, a machine-learning based platform called “e-Sweet” will be developed to automate the prediction of sweetener and its RS via the simple mouse-click on the graphic user interface. The detail of these functions and their implementation will be elaborated below.

## Materials and Methods

### Sweetener Prediction Based on the Multiple Machine Learning Methods

**Figure 1:**
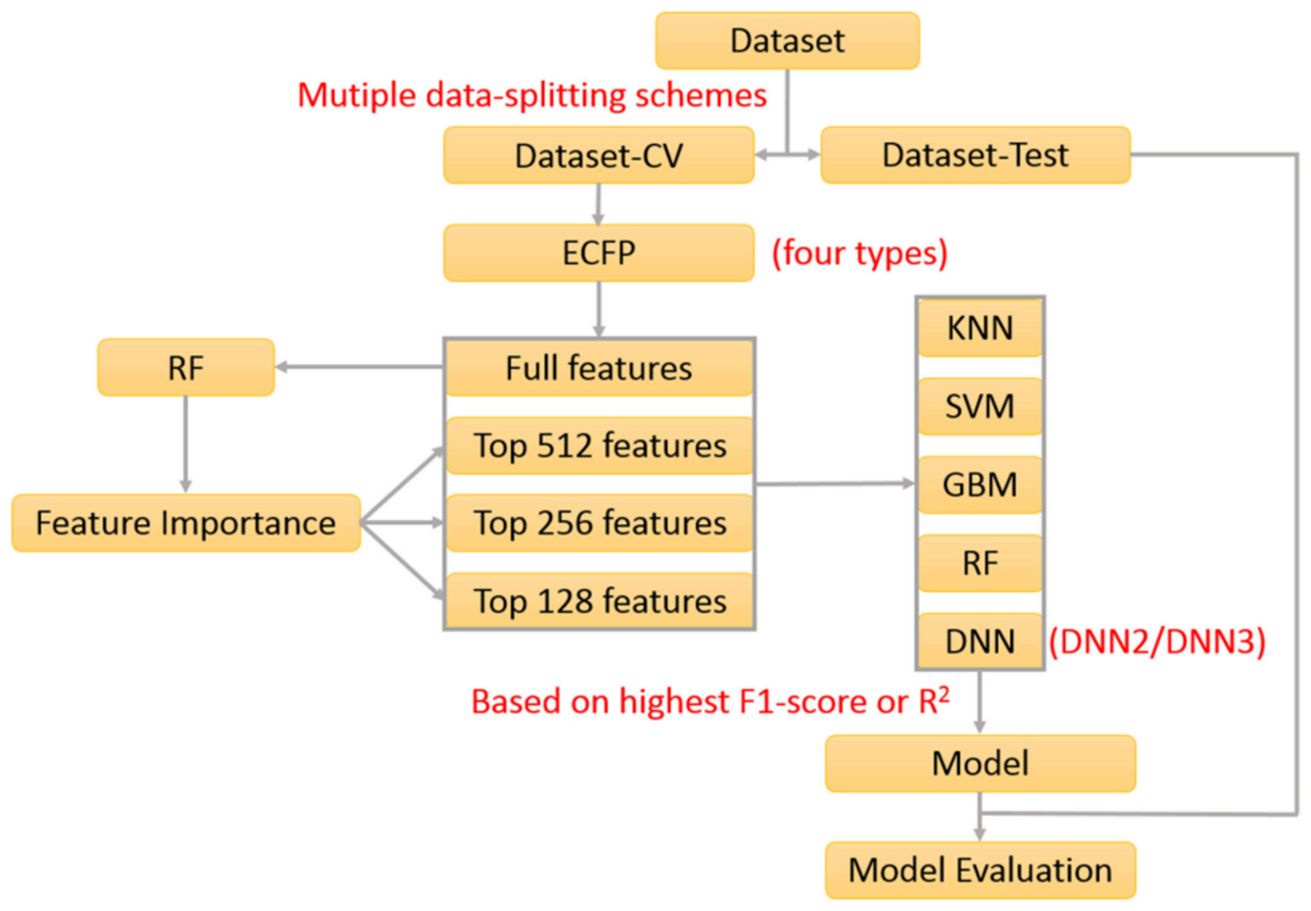
The protocol to derive the classification and regression model used in this work.

In our previous work about the bitterant prediction (Zheng et al., 2018), we develop a systematic and general protocol to build the classification model, which makes full use of the multiple machine-learning methods (KNN, SVM, GBM, RF, and DNN) by the consensus voting and adopts the Extended-connectivity Fingerprint (ECFP) (Rogers and Hahn, 2010) as the molecular descriptor. In practice, this protocol can be further adapted to generate the regression model. In this work, we will exploit this protocol (Figure 1) to derive the machine-learning based models for the prediction of sweeteners and its RS.

In our work, 530 sweeteners are curated from SuperSweet (Ahmed et al., 2011) and SweetenersDB (Chéron et al., 2017) and additionally gathered from the literature (Rojas et al., 2016a; Banerjee and Preissner, 2018), while 850 non-sweeteners consist of 718 bitter compounds downloaded from BitterDB (Wiener et al., 2012) and 132 tasteless compounds retrieved from the literature (Rojas et al., 2016a). Four criteria are defined for the data curation above. (1) Only the larger fragment is kept for the disconnected structures such as salt. (2) Only the compounds containing the elements C, H, O, N, S, P, Si, F, Cl, Br, or I are considered. (3) The same compound with the different taste modalities is excluded. (4) The duplicated compounds from the different sources are eliminated. Based on these standards, all the compounds are finally saved as the Tripos mol2 files, which are integrated into e-Sweet platform for the public access.

In order to train and test the classification model, the whole dataset is randomly divided into two parts: the dataset for the cross-validation (Dataset-CV) and the hold-out test set for the independent validation (Dataset-test). The detailed data-splitting scheme is given as follows: 80% of sweeteners (339 compounds) and 80% of non-sweeteners (544 compounds) randomly selected from the whole dataset are adopted to train the model with the five-fold cross-validation, while the rest of them (221 compounds) are used as the hold-out test set. Finally, this whole data-splitting will be repeated for nineteen or three times to reduce the bias from the random data-splitting. Concretely, nineteen data-splitting schemes are performed for KNN, SVM, GBM, and RF, while three data-splitting schemes are carried out for deep neuron network (DNN) on account of its much higher computational burden.

Besides the indispensable dataset and its partition above, the molecular descriptors are also required for the machine-learning method. Extended-connectivity Fingerprint (ECFP), which is extensively used in the quantitative structure-activity relationship (QSAR) studies (Ekins et al., 2010; Chen et al., 2011; Hu et al., 2012; Koutsoukas et al., 2016; Braga et al., 2017; Rodríguez-Pérez et al., 2017), is adopted as the molecular descriptor in this work. Four ECFPs (1024bit-ECFP4, 2048bit-ECFP4, 1024bit-ECFP6, and 2048bit-ECFP6) are generated for all the curated compounds in the aforementioned dataset with our own implementation of ECFP in e-Bitter program (Zheng et al., 2018), which uniquely offers the intuitive visualization of each “1” bit of ECFP in the context of 3D structure and is also integrated into e-Sweet platform.

Furthermore, feature selection is generally applied in the machine-learning method. In this work, both full-feature without the feature selection and feature-subset with the feature selection are considered. Here the feature selection is performed according to the feature importance (Figure 1), which is derived from the model-training with the random forest (RF) method that will be described in the following paragraph about the model-training. In total, 76 runs of model-training with RF are conducted by considering the combination of four ECFP fingerprints and nineteen random data-splitting of the dataset, which will lead to 76 models and the attendant 76 sets of feature importance. Then the feature importance for all the bits in the ECFP fingerprint is sorted in the descending order and plotted in Figures S1–S4. Thus the top 512, 256, and 128 important features (Figures S1–S4) are selected, respectively as the typical feature subsets for the following model-training, since the exhaustive and systematic scan of feature-number ranging from 1 to fingerprint-length is really time-consuming especially for the training of deep neuron networks (DNN).

Five machine-learning methods (KNN, SVM, GBM, RF, and DNN) are utilized to train the model, which are minutely introduced in our previous work about the bitterant prediction (Zheng et al., 2018) and briefly summarized as follows. K-nearest neighbors (KNN) method conducts the classification and regression based on the closest instances in the training set. Support vector machine (SVM) performs the classification and regression via constructing the hyper-planes in the high-dimensional space. Random forest (RF) and gradient boosting machine (GBM) belong to the decision-tree based ensemble method. RF builds a multitude of decision trees by the bootstrap-sampling of training set and random-selection of feature-subset. GBM generates a series of decision trees in a step-wise manner, rather than in a random way as RF. Deep neuron network (DNN) is a neural network with more than one hidden layer between the input and output layers. Nowadays, thousands of neurons in each layer can be routinely adopted in DNN, which can combine the advanced regularization technique such as the dropout to avoid the overfitting. In this work, the deep neuron networks with two hidden layers (DNN2 in Figure S5) and three hidden layers (DNN3 in Figure S6) are employed. All the key parameters for each method are listed in Table S1, which will be fine-tuned in the five-fold cross-validation (**CV**) to achieve the optimal performance.

The performance of models on the training set and test set are evaluated by the following metrics: the accuracy, precision, specificity, sensitivity, Matthews correlation coefficient (MCC), non-error rate (NER) and F1-score (Equations 1–6). It should be noted that F1-score (Equation 1) is adopted as the criterion to select the best model, albeit F1-score, MCC, and NER are commonly used to measure the quality of the classification.

(1) $$\begin{array}{lll} \text{F}1\text{-score} & = & 2\times \text{TP}/\left(2\times \text{TP}+\text{FP}+\text{FN}\right) \end{array}$$

(2) $$\begin{array}{lll} \text{Accuracy} & = & \left(\text{TP}+\text{TN}\right)/\left(\text{TP}+\text{TN}+\text{FP}+\text{FN}\right) \end{array}$$

(3) $$\begin{array}{lll} \text{Precision} & = & \text{TP}/\left(\text{TP}+\text{FP}\right) \end{array}$$

(4) $$\begin{array}{lll} \text{Specificity} & = & \text{TN}/\left(\text{TN}+\text{FP}\right) \end{array}$$

(5) $$\begin{array}{lll} \text{Sensitivity} & = & \text{TP}/\left(\text{TP}+\text{FN}\right) \end{array}$$

(6) $$\begin{array}{lll} MCC & = & \frac{\left(\text{TP}\times \text{TN}-\text{FP}\times \text{FN}\right)}{\sqrt{\left(\text{TP}+\text{FP}\right)\;\left(\text{TP}+\text{FN}\right)\;\left(\text{TN}+\text{FP}\right)\;\left(\text{TN}+\text{FN}\right)}} \end{array}$$

(7) $$\begin{array}{l} \Delta \text{F}1\text{-}\text{score}=|\text{F}1\text{-score}(\text{cross-validation})\\ \;-\text{F}1\text{-score}\left(\text{test set}\right)| \end{array}$$

(8) $$\begin{array}{lll} \text{NER} & = & \left(\text{Sensitivity}+\text{Specificity}\right)/2 \end{array}$$

TP, TN, FP, and FN are the numbers of true sweeteners, true non-sweeteners, false sweeteners, and false non-sweeteners, respectively. NER is short for non-error rate and is the arithmetic mean of sensitivity and specificity in the binary classification. ΔF1-score is calculated to monitor the potential over-fitting or under-fitting.

Upon completion of the model-training with the five-fold cross-validation, totally 1312 models including 328 models without feature selection and 984 models with feature selection are harvested according to the highest F1-score, and are further gauged on the respective hold-out test sets with the evaluation metrics: accuracy, precision, specificity, sensitivity, F1-score, MCC, and NER, which are listed in Table S2. To reduce the bias from the random splitting of the whole dataset, 96 average models (AM) are derived from 1,312 individual models by averaging over the different data-splitting schemes and are tabulated in Table S3.

Following the guidelines of OECD, Y-randomization test for our models should be performed and the applicability-domain of our models should be also defined practically. To inspect the robustness of all the models, Y-randomization test is done with the following procedure. Firstly, the experimentally observed labels for Dataset-CV are randomly shuffled (Table S4). Subsequently, the five-fold cross-validation on this noisy dataset is performed with exactly the same molecular descriptors and the same protocol mentioned in the previous section about the model-training. The best models are also determined based on the highest F1-score assessed on the internal validation dataset during the cross-validation, and further evaluated on the hold-out test set (**Dataset-Test**) without any random shuffling. All the results are collected in Tables S5–S6. Meanwhile, with regard to the definition of the applicability domain, it is generally hypothesized that the compound, which is highly dissimilar to all the compounds used in the model-training, may not be predicted confidently (Tropsha, 2010). With this assumption in our mind, the applicability domain of our models is defined on the basis of the ECFP based Tanimoto-similarity between the compound of interest and its five closest neighboring compounds in our training set (Dataset-CV).

Finally, 1,312 individual models (M0001–M1312 in Table S2) and 96 average models (AM01–AM96 in Table S3) are obtained after the model training and validation. Based on these models, four consensus models are proposed according to the criteria such as the performance, speed and diversity of machine-learning based models, and are integrated into our e-Sweet platform. All the constitute models for each consensus model are provided in Tables S7–S10 and the performances of these four consensus models are given in Table 1. More specifically, Consensus model 1 (CM01) selects 19 best individual models (Table S7) with all the methods except DNN purely based on the highest F1-scores in each data-splitting scheme from the perspective of performance and speed. Consensus model 2 (CM02) selects 19 best individual models (Table S8) with all the methods including DNN solely based on the highest F1-scores in each data-splitting scheme according to the model performance. Consensus model 3 (CM03) considers the top five average models (Table S9) with the highest F1-scores. Consensus model 4 (CM04) chooses the five average models (Table S10) considering each machine-learning method with the highest F1-score to balance the performance and diversity of machine-learning based models. All the evaluation metrics for each consensus model (Table 1) are obtained by averaging over all the constituent models.

**Table 1:** The performance of four consensus models (CM01–CM04) for the sweetener/non-sweetener classification.

**Model**	**Accuracy** **(test set)**	**Precision** **(test set)**	**Specificity** **(test set)**	**Sensitivity** **(test set)**	**F1-score** **(test set)**	**MCC** **(test set)**	**NER** **(test set)**	**F1-score** **(CV)**	**NER** **(CV)**	**ΔF1-score**	**ΔNER**
**MEAN(STANDARD DEVIATION)**											
CM01	0.91 (0.01)	0.90 (0.03)	0.94 (0.02)	0.85 (0.02)	0.88 (0.02)	0.80 (0.02)	0.90 (0.01)	0.85 (0.01)	0.87 (0.01)	0.03 (0.02)	0.03 (0.01)
CM02	0.91 (0.01)	0.90 (0.03)	0.94 (0.02)	0.86 (0.03)	0.88 (0.02)	0.81 (0.03)	0.90 (0.01)	0.85 (0.01)	0.87 (0.01)	0.04 (0.02)	0.03 (0.02)
CM03	0.89 (0.00)	0.89 (0.01)	0.93 (0.01)	0.83 (0.00)	0.86 (0.00)	0.77 (0.01)	0.88 (0.00)	0.85 (0.01)	0.87 (0.00)	0.02 (0.01)	0.02 (0.01)
CM04	0.89 (0.01)	0.89 (0.02)	0.94 (0.01)	0.82 (0.01)	0.85 (0.01)	0.76 (0.01)	0.88 (0.01)	0.84 (0.00)	0.87 (0.00)	0.02 (0.00)	0.02 (0.00)
**95% CONFIDENCE INTERVAL: MEAN** ± **MARGIN OF ERROR**											
CM01	0.91 ± 0.01	0.90 ± 0.01	0.94 ± 0.01	0.85 ± 0.01	0.88 ± 0.01	0.80 ± 0.01	0.90 ± 0.01	0.85 ± 0.01	0.87 ± 0.01	0.03 ± 0.01	0.03 ± 0.01
CM02	0.91 ± 0.01	0.90 ± 0.01	0.94 ± 0.01	0.86 ± 0.01	0.88 ± 0.01	0.81 ± 0.01	0.90 ± 0.01	0.85 ± 0.01	0.87 ± 0.01	0.04 ± 0.01	0.03 ± 0.01
CM03	0.89 ± 0.00	0.89 ± 0.01	0.93 ± 0.01	0.83 ± 0.00	0.86 ± 0.00	0.77 ± 0.00	0.88 ± 0.00	0.85 ± 0.00	0.87 ± 0.00	0.02 ± 0.00	0.02 ± 0.00
CM04	0.89 ± 0.01	0.89 ± 0.02	0.94 ± 0.01	0.82 ± 0.01	0.85 ± 0.01	0.77 ± 0.01	0.88 ± 0.01	0.84 ± 0.00	0.87 ± 0.00	0.02 ± 0.00	0.02 ± 0.00

*(1) The number in each parenthesis is the standard deviation, which is derived based on the multiple random data-splitting schemes; (2) ΔF1-score and ΔNER refer to | F1-score (test set)–F1-score (cross-validation) | and | NER (test set)–NER (cross-validation) | respectively; (3) “MCC,” “CV,” and “NER” are short for “Matthews correlation coefficient,” “cross-validation,” and “Non-error Rate,” respectively*.

### Sweetness Prediction Based on Multiple Machine Learning Methods

In our work, all the sweeteners with RS are gathered from the literature (Iwamura, 1981; Drew et al., 1998; Kinghorn and Soejarto, 2002; Vepuri et al., 2007; Yang et al., 2011), and subjected to the filtering with the following criteria. (1) Only the larger fragment is saved for the disconnected structures such as salts. (2) Only the compounds containing the elements C, H, O, N, S, P, Si, F, Cl, Br, or I are considered. (3) Only one compound is kept for the duplicated compounds from the different sources. (4) Only the compound with the experimental RS, which is only measured relative to the 5% (w/v) sucrose, is taken account. After the filtering with these conditions, 352 sweeteners are curated for our subsequent training with the machine learning methods. All the structures with the Tripos mol2 files, and their corresponding log_10_RS (common logarithm of the relative sweetness) used as the dependent variable (Y) are publicly available in our e-Sweet platform. To train and validate the model, the whole dataset is sorted ascendingly according to log_10_RS. Twenty percent of the whole dataset (71 compounds) is randomly selected from every five compounds in the ascending order to form the hold-out test set (Dataset-Test) with the even distribution of log_10_RS. The rest of them (281 compounds) are adopted to train the model with the five-fold cross-validation (Dataset-CV). Similarly, the whole data-splitting is repeated for the multiple times as well.

To derive the regression model for RS, nearly the same protocol (Figure 1) as the sweetener/non-sweetener classification is adopted. According to this protocol, all the combination of the molecular fingerprints, feature selection, feature number, data-splitting schemes, and machine-learning methods is taken into account in the model-training, and thereby 1,312 best individual models are also achieved based on the highest R^2^ (square of the coefficient of determination) after the five-fold cross-validation, and are further assessed on the respective hold-out test sets with the evaluation metrics: R^2^, mean absolute error (MAE), mean squared error (MSE), and ΔR^2^ (referring to |R^2^(test set)–R^2^(cross-validation)|), which are summarized in Table S11. Subsequently, 96 average models are also obtained based on 1,312 individual models by averaging over the different data-splitting schemes, whose performances are given in Table S12. Furthermore, Y-randomization test (Tables S13–S15) and defining the applicability-domain for our models are also carried out with the similar protocol in the previous section about the classification model. Finally, three consensus models (CM01–CM03 in Tables S16–S18) are suggested on the basis of 1,312 individual models and 96 averages models and are embedded into our e-Sweet platform.

## Results and Discussion

### Overview of e-Sweet Platform

e-Sweet is a machine-learning based platform for the automatic prediction of the sweetener and its RS, which is developed based on our previous e-Bitter program (Zheng et al., 2018). This e-Sweet platform can be easily installed via the simple click of mouse and can smoothly run both in the modes of graphic user-interface and command-line, which are well tested on the Win7, Win8, and Win10. The whole program including the manual and tutorials can be freely from the link (https://www.dropbox.com/sh/1fmlv7nf6wofgcp/AADBJzFbbbiNRJUP0806wSyna?dl=0).

**Figure 2:**
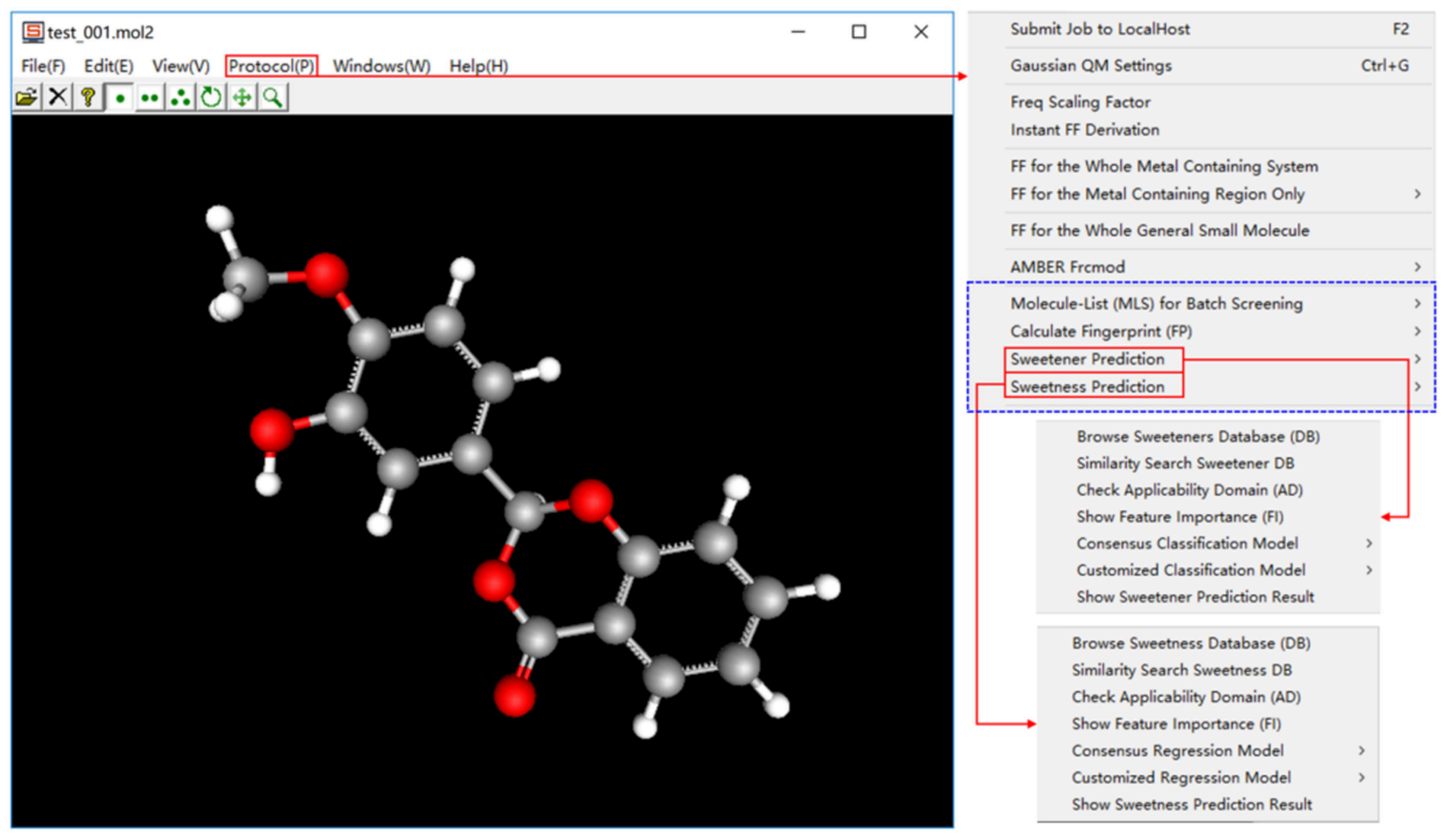
The main features of e-Sweet platform for the sweetener and sweetness prediction.

In the current version of e-Sweet, there are several major helpful functions for the food scientists. (1) Visualize and inquiry our curated datasets for the classification of sweetener/non-sweetener or the regression of RS. (2) Predict the sweetener and its RS with the multiple machine-learning methods by evoking the external scikit-learn (v0.19.1), Keras (v1.1.0), and Theano (v1.0.1) python libraries fully integrated in the free Anaconda (v2-5.2.0) that can also be handily installed on the windows in the simple way. (3) Virtual screening of database to enrich the possible sweetener candidates. (4) Generate and visualize the ECFP fingerprint, which is adopted as the molecular descriptor and is also natively implemented in this platform. (5) View the fingerprint bit in the context of 3D structure, and synchronously display the feature importance of fingerprint bit contributing to the classification of sweetener/non-sweetener or regression of RS. The detailed usage of all those functions is articulated in the manual and tutorials, while only the key functions (Figure 2) will be detailed as follows.

In a nutshell, e-Sweet is the first, free, and convenient standalone software for the experimental food scientists to automate the prediction of the sweetener and its corresponding RS with the machine-learning based classification and regression models, and also offers several key auxiliary functions relevant to the prediction.

### The Chemical Space of Our Curated Datasets Embedded in e-Sweet

Our curated datasets for the classification of sweetener/non-sweetener and the regression of RS are publicly available and fully integrated into our e-Sweet platform, with which users can simultaneously visualize the chemical structures and the corresponding labels (or log_10_RS) and can conveniently enquiry our datasets with the compounds of users' interests by Tanimoto-similarity based structure search (Figure S7).

Our dataset for the sweetener/non-sweetener classification consists of 530 sweeteners and 850 non-sweeteners, which is the largest dataset so far. In the latest sweetener/non-sweetener classification model from Rojas et al. 435 sweeteners and 214 non-sweeteners are utilized, which is much less than ours. To examine the difference of chemical spaces between the sweeteners and non-sweeteners in our dataset, the molecular weight (MW), logP, and the numbers of hydrogen-bond donor (N_HBD_) and hydrogen-bond acceptor (N_HBA_) are calculated by OpenBabel v2.4 (Oboyle et al., 2011). The scatter plots of logP vs. MW (Figure S8) and N_HBD_ vs. N_HBA_ (Figure S9) showcase that the distributions for the sweeteners are very similar to the counterpart for the non-sweeteners. Hence the intuitive discrimination between the sweeteners and non-sweeteners by the simple descriptors such as logP, MW, N_HBD_, and N_HBA_ is not effective. Furthermore, ECFP based similarity-matrix (Figure S10) illustrates that the overall pairwise Tanimoto-similarities between the sweeteners and non-sweeteners are quite low with the average value of 0.08 over the entire matrix, indicating that ECFP fingerprint may be a promising molecular descriptor for the classification of sweeteners and non-sweeteners.

In addition, our dataset for the regression of RS is composed of 352 sweeteners, and is larger than the datasets utilized in most of relevant works (Zhong et al., 2013; Rojas et al., 2016c; Chéron et al., 2017; Ojha and Roy, 2017), but is smaller than the dataset used in the work of Goel et al. which is made up of 455 sweeteners that is not directly accessible to the other researchers (Goel et al., 2018). It is worth mentioning that both works glean the sweeteners with RS from the same source (Iwamura, 1981; Drew et al., 1998; Kinghorn and Soejarto, 2002; Vepuri et al., 2007; Yang et al., 2011), thus the different number of sweeteners used in both works is presumably resulted from the distinct curation criteria. To check the conformational flexibility of the sweeteners in this dataset, the numbers of the freely rotatable bonds (N_FRB_) for all the sweeteners are computed by OpenBabel v2.4 and the histogram of N_FRB_ (Figure S11) demonstrates that most of the sweeteners are quite flexible and have many conformers, which may bring about the irreproducible result for the model prediction if the conformation-dependent 3D molecular descriptors are used to establish the model. Therefore, ECFP based 2D molecular descriptors are used in this work.

In a word, our dataset for the sweeteners/non-sweeteners classification is the largest and the dataset for the sweeteners with RS is the second largest, and both datasets are publicly available to other researchers. ECFP based similarity-matrix indicates that ECFP based 2D descriptor could be beneficial to the classification of sweeteners and non-sweeteners, and the analysis of conformational flexibility of the sweeteners in this dataset casts light on the potential weakness of the conformation-dependent 3D molecular descriptors.

### Prediction of Sweetener by the Classification Model in e-Sweet

**Figure 3:**
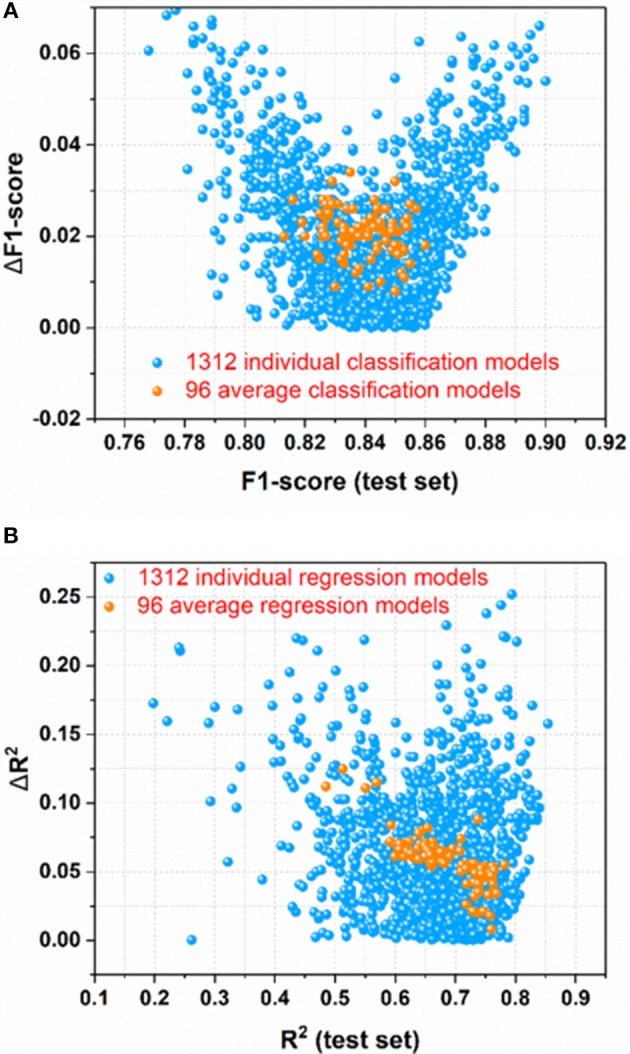
**(A)** the scatter-plot of ΔF1-score vs. F1-score for all the classification models; **(B)** The scatter plot of ΔR^2^ vs. R^2^(test set) for all the regression models. ΔF1-score [referring to |F1-score (test set)–F1-score(cross-validation)|] and ΔR^2^ [referring to |R^2^(test set)–R^2^(cross-validation)|] are used to monitor the potential overfitting or underfitting.

For the sweetener/non-sweetener classification, 1,312 individual classification models (M0001–M1312 in Table S2) and 96 average classification models (AM01–AM96 in Table S3) are harvested. The scatter-plot of ΔF1-score vs. F1-score for all the models is plotted in Figure 3A, since F1-score is the performance indicator of the classification model and ΔF1-score is used to examine the possible over-fitting or under-fitting of the classification model. Figure 3A demonstrates that ΔF1-score for most of individual and average classification models is lower than 0.04, suggesting that the model performance on the test set and in the cross-validation is quite similar. Thus, most of our models do not suffer from the obvious over-fitting or under-fitting from this perspective. Moreover, the orange dots (Figure 3A) standing for 96 average classification models based on the multiple data-splitting schemes have much narrower distribution than the blue dots (Figure 3A) denoting 1,312 individual classification models on the basis of the single data-splitting scheme, which provides an important clue that the different random data-splitting schemes have dramatic effects on the model performance. Therefore, it is a good practice for the machine-learning practitioners to repeat the data-splitting for the multiple times to gain more objective models.

To further inspect the robustness of all 1,312 individual and 96 average classification models, Y-randomization test is performed for all the classification models by the random shuffling of experimental labels in Dataset-CV (Table S4), and all the results are tabulated in Tables S5–S6. For the better illustration, the scatter plot of F1-score(test set) vs. MCC(test set) for all the models is plotted in Figure S12, which clearly demonstrates that the model performances after Y-randomization is drastically decreased relative to the models without Y-randomization. Accordingly, all our previous models without Y-randomization are quite robust and not obtained by chance.

However, it is not very efficient to harness all 1,312 individual and 96 average classification models simultaneously for the pragmatic prediction of sweeteners, consequently four typical consensus models (CM01–CM04 in Tables S7–S10) are suggested based on the performance, speed, and diversity of the models, and are incorporated into our e-Sweet platform. Observed from Table 1, the overall performances of all these consensus models on the test set (Table 1) are very promising, while the best model CM02 with the highest F1-score can achieve the 95% confidence intervals for the accuracy (0.91 ± 0.01), precision (0.90 ± 0.01), specificity (0.94 ± 0.01), sensitivity (0.86 ± 0.01), F1-score (0.88 ± 0.01), MCC (0.81 ± 0.01), and NER (0.90 ± 0.01) on the test set by averaging over the 19 data-splitting schemes.

To demonstrate the advantage of our models, CM01–CM04 in Tables S7–S10 are compared with the model in the work of Rojas et al. which is the only published work about the sweetener/non-sweeteners classification based on the relatively large dataset and affords the NER values of 0.85 and 0.83 on the test set and in the cross-validation, respectively, whereas the evaluation metrics such as F1-score and MCC are not reported in their work. The procedure for the statistical comparison is given as follows. (1) Bland-Altman analysis (Martin Bland and Altman, 1986) is firstly conducted to examine whether NER(test set) and NER (cross-validation) of the models from Rojas et al. match well within the limits of agreement (LoA) in the Bland-Altman plots based on our consensus classification models. (2) If NER(test set) and NER (cross-validation) of the models from Rojas et al. agree well, it indicates that their model probably does not suffer from the evident over-fitting or under-fitting. Subsequently, further comparison will be performed to check whether their model is within the 95% confidence intervals of ΔNER (referring to |NER(test set)–NER(cross-validation)|) and NER(test set), respectively.

According to this comparison protocol, Figure S14 clearly illustrates that NER(test set) and NER(cross-validation) of the model from Rojas et al. agree very well in all the Bland-Altman plots (Figure S14) based on CM01, CM02, CM03, and CM04. Subsequently, NER(test set) and ΔNER will be used as the performance indicators for the further comparisons. More specifically, ΔNER of the model from Rojas et al. is 0.02, and is within the 95% confidence intervals of ΔNER from our CM01, CM02, CM03, and CM04, which are 0.03 ± 0.01, 0.03 ± 0.01, 0.02 ± 0.00, and 0.02 ± 0.00, respectively. Meanwhile, NER(test set) of the model from Rojas et al. is 0.85, and is consistently lower than the 95% confidence intervals of NER (test set) from our CM01, CM02, CM03, and CM04, which are 0.90 ± 0.01, 0.90 ± 0.01, 0.88 ± 0.00, and 0.88 ± 0.01, respectively. Therefore, all four consensus sweetener/non-sweeteners classification models are better than the model from Rojas et al.

In short, the robust sweetener/non-sweetener classification models based on the largest dataset, multiple data-splitting schemes and manifold machine-learning methods are derived, and our proposed four consensus models are demonstrated to outperform the model from Rojas et al. that is based on the single data-splitting scheme.

### Prediction of Relative Sweetness by the Regression Model in e-Sweet

For the prediction of RS, 1,312 individual regression models (M0001–M1312 in Table S11) and 96 average regression models (AM01–AM96 in Table S12) are achieved. The scatter plot of ΔR^2^ [referring to |R^2^(test set)–R^2^(cross-validation)|] vs. R^2^(test set) for all the models is made for the assessment of overall performance, because R^2^(test set) is the performance indicator of regression model and ΔR^2^ is used to monitor the potential over-fitting or under-fitting of regression model. From Figure 3B, it illustrates that ΔR^2^ for most of the individual and average regression models is <0.10, implying that the models achieve the consistently similar performance on the hold-out test set and in the cross-validation, respectively. Thus, most of the models do not exhibit the noticeable over-fitting or under-fitting from this point of view. In addition, observed from Figure 3B, the more compact distribution of the average models relative to the individual models also emphasizes that the average models based on the multiple data-splitting schemes are more convergent than the individual models based on the single data-splitting scheme.

To further ensure the robustness of all the individual and average regression models, Y-randomization test is also conducted for all the regression models by the random shuffling of experimental ${\text{log}}_{10}^{\text{RS}}$ in Dataset-CV (Table S13), and all the outcomes are given in Tables S14, S15. For the sake of intuitive description, the scatter plot of R^2^(test set) vs. MAE(test set) for all the models before and after Y-randomization in Figure S13 unambiguously illustrates that our regression models without Y-randomization are reliable.

Nevertheless, it is not realistic to utilize all the 1,312 individual and 96 average regression models at the same time for the practical prediction of RS, hence three representative consensus models (CM01-CM03 in Tables S16–S18) are proposed and integrated into our e-Sweet platform. Table 2 illustrates that our consensus models (CM01–CM03) on the basis of the individual and average models afford R^2^(test set) ranging from 0.77 to 0.78. CM02 has the highest R^2^(test set) with the 95% confidence interval of 0.78 ± 0.02, while CM03 provides the lowest ΔR^2^ with the 95% confidence interval of 0.03 ± 0.01.

**Table 2:** The performance of three consensus models (CM01–CM03) for the regression of relative sweetness (RS).

**Model**	***R^**2**^*** **(test set)**	**MSE** **(test set)**	**MAE** **(test set)**	***R^**2**^*** **(CV)**	***ΔR^**2**^***
**MEAN(STANDARD DEVIATION)**					
CM01	0.77 (0.05)	0.27 (0.06)	0.39 (0.03)	0.72 (0.05)	0.07 (0.05)
CM02	0.78 (0.05)	0.28 (0.06)	0.40 (0.03)	0.71 (0.05)	0.07 (0.05)
CM03	0.77 (0.01)	0.58 (0.31)	0.58 (0.17)	0.74 (0.01)	0.03 (0.01)
**95% CONFIDENCE INTERVAL: MEAN** **±** **MARGIN OF ERROR**					
CM01	0.77 ± 0.02	0.27 ± 0.03	0.39 ± 0.01	0.72 ± 0.02	0.07 ± 0.02
CM02	0.78 ± 0.02	0.28 ± 0.03	0.40 ± 0.01	0.71 ± 0.02	0.07 ± 0.02
CM03	0.77 ± 0.01	0.58 ± 0.27	0.58 ± 0.15	0.74 ± 0.01	0.03 ± 0.01

*(1) The number in each parenthesis is the standard deviation, which is obtained on the basis of the multiple random data-splitting schemes; (2) ΔR^2^ referring to | R^2^(test set)–R^2^(cross-validation) | is employed to monitor the potential over-fitting/under-fitting; (3) “CV” is short for the cross-validation*.

For the sake of the easier comparison with the other works about the prediction of RS, R^2^(test set) and R^2^(cross-validation) are generally reported in the respective works and compiled in Table S19, which are all based on only one data-splitting scheme to prepare the hold-out test set and training set in the other works. The statistical comparison between ours and other models is very similar to the aforementioned comparisons between the classification models and will be carried out as follows: (1) Bland-Altman method is firstly adopted to check whether R^2^(test set) and R^2^(cross-validation) of the models from other works agree well within the limits of agreement in the Bland-Altman plots based on our consensus regression models. (2) If R^2^(test set) and R^2^(cross-validation) of the models from other works agree well, the 95% confidence intervals of |R^2^(test set)-R^2^(cross-validation)| and R^2^(test set) for our models are used for the further comparison with the models from other works. Otherwise, the model may suffer from the over-fitting or under-fitting due to the distinct difference between R^2^(test set) and R^2^(cross-validation) and thereby will be excluded in the subsequent comparison.

From Figure S15, all the models from other works exceed the upper or lower limits of agreement (LoA) and their 95% confident intervals, which reveals that the model performances of other models on the test set and in the cross-validation do not agree well compared to the counterpart of our consensus model CM03. Thus, CM03 is the best model in term of the agreement between R^2^(test set) and R^2^(cross-validation). However, all the constituent models in CM03 are derived from DNN method, which are much slower relative to the models derived from the other machine-learning methods such as KNN, SVM, GBM, and RF. Therefore, we proposed two other consensus models (CM01 and CM02). CM02 is constructed on 19 best constituent models in 19 data-splitting schemes. However, in CM02 there is still one constituent model that comes from the time-consuming DNN method. Thus, CM01 is suggested also based on 19 best constituent models by excluding the model from DNN method. As a result, CM01 has very similar constituent models relative to CM02, but is much faster than CM02 and thereby is suitable for the database screening. Thus, it is understandable that Bland-Altman plots (Figure S15) of CM01 and CM02 are very similar.

Therefore, the Bland-Altman plot (Figure S15A) based on CM01 is taken as an instance. Five 3D descriptors based models are very close to the limits of agreement (LoA), however, those models can be still assumed that R^2^(test set) and R^2^(cross-validation) of these five models are agreeable according to the Bland-Altman plot based on CM01 (Figure S15A), while only one 3D descriptors based model completely locates outside the upper and lower LoA and their 95% confident intervals. These five acceptable 3D descriptors based models in Bland-Altman plot (Figure S15A) are MLR model from Zhong et al. SVM model from Zhong et al. GFA model from Goel et al. ANN model from Goel. et al. and SVM model from Cheron et al. which afford ΔR^2^ with the values of 0.04, 0.05, 0.03, 0.06, and 0.16, respectively (Table S19). According to Table 2, the 95% confidence interval of ΔR^2^ for our CM01 is 0.07 ± 0.02. Consequently, the ΔR^2^ of SVM model from Cheron et al. is much larger than the 95% confidence interval (0.07 ± 0.02) from CM01. Finally, four remaining models will be further compared with our model CM01 based on R^2^(test set). MLR model with R^2^(test set) value of 0.77 and SVM model with R^2^(test set) value of 0.78 from Zhong et al. (Table S19) are still within the 95% confidence interval (0.77 ± 0.02) of R^2^(test set) for our CM01, while GFA model with R^2^(test set) value of 0.83 and ANN model with R^2^(test set) value of 0.83 from Goel et al. (Table S19) is higher than the 95% confidence interval (0.77 ± 0.02) of R^2^(test set) for our CM01 (Table 2). Therefore, our CM01 has a similar performance with the MLR and SVM models from Zhong et al. and shows the lower performance than the GFA and ANN models from Goel et al. It is worth mentioning that this conclusion also holds for CM02. Nevertheless, Goel et al. employed the conformation-dependent 3D molecular descriptors such as the LUMO eigenvalue, which requires the time-consuming quantum mechanical (QM) calculation particularly for the large molecules. Moreover, the flexible sweeteners usually possess very diverse conformations due to a number of freely rotatable bonds, which may provide the totally different molecular descriptors for the same compound and thereby may lead to the irreproducible result in the practical prediction. Actually the work of Rojas et al. also well addresses this issue and suggests to adopt the 2D molecular descriptors for the simplicity and the fast speed. Thus, ECFP based 2D molecular descriptors are adopted in our work.

As such, 2D descriptors based models including ours will be the main focus for the comparison of model performance. Two 2D descriptors based models from other works are very close to the limits of agreement (LoA), albeit they are still in the acceptable region. One model from Rojas et al. is trained with MLR and 2D Dragon descriptors, and gives R^2^(test set), R^2^(cross-validation), and ΔR^2^ of 0.70, 0.78, and 0.08, respectively, while the other from Cheron et al. is built with SVM and 2D Dragon descriptors, and offers R^2^(test set), R^2^(cross-validation), and ΔR^2^ of 0.83, 0.69, and 0.14, respectively. However, the 95% confidence interval of ΔR^2^ for our CM01 model is 0.07 ± 0.02. Hence only the model from Rojas et al. is within the 95% confidence interval (0.07 ± 0.02) of ΔR^2^. Finally, the model comparison based on R^2^(test set) illustrates that CM01 is better than the model from Rojas et al. since R^2^(test set) with the value of 0.70 from Rojas et al. is much lower than the 95% confidence interval (0.77 ± 0.02) of R^2^(test set) from CM01. It is noteworthy that this conclusion can also apply to CM02.

In sum, our consensus regression model CM03 is prominently promising than all the models from other works in term of agreement between R^2^(test set) and R^2^(cross-validation) based on the Bland-Altman plot of CM03, while CM01/CM02 remarkably outperforms the 2D descriptors based models from other works according to the full analysis of Bland-Altman plot and the 95% confident intervals of ΔR^2^ and R^2^(test set), but is inferior to the 3D descriptors based models from Goel et al. that are derived from the single data-splitting scheme. However, the 3D descriptors based models are not pragmatic for the prediction by other users. Furthermore, it still should be taken with caution that R^2^(test set) from the single data-splitting scheme is adopted to compare the model performance, since different data-splitting schemes have apparent effects on the model performance.

### Automatic Inspection of Applicability Domain in e-Sweet

**Figure 4:**
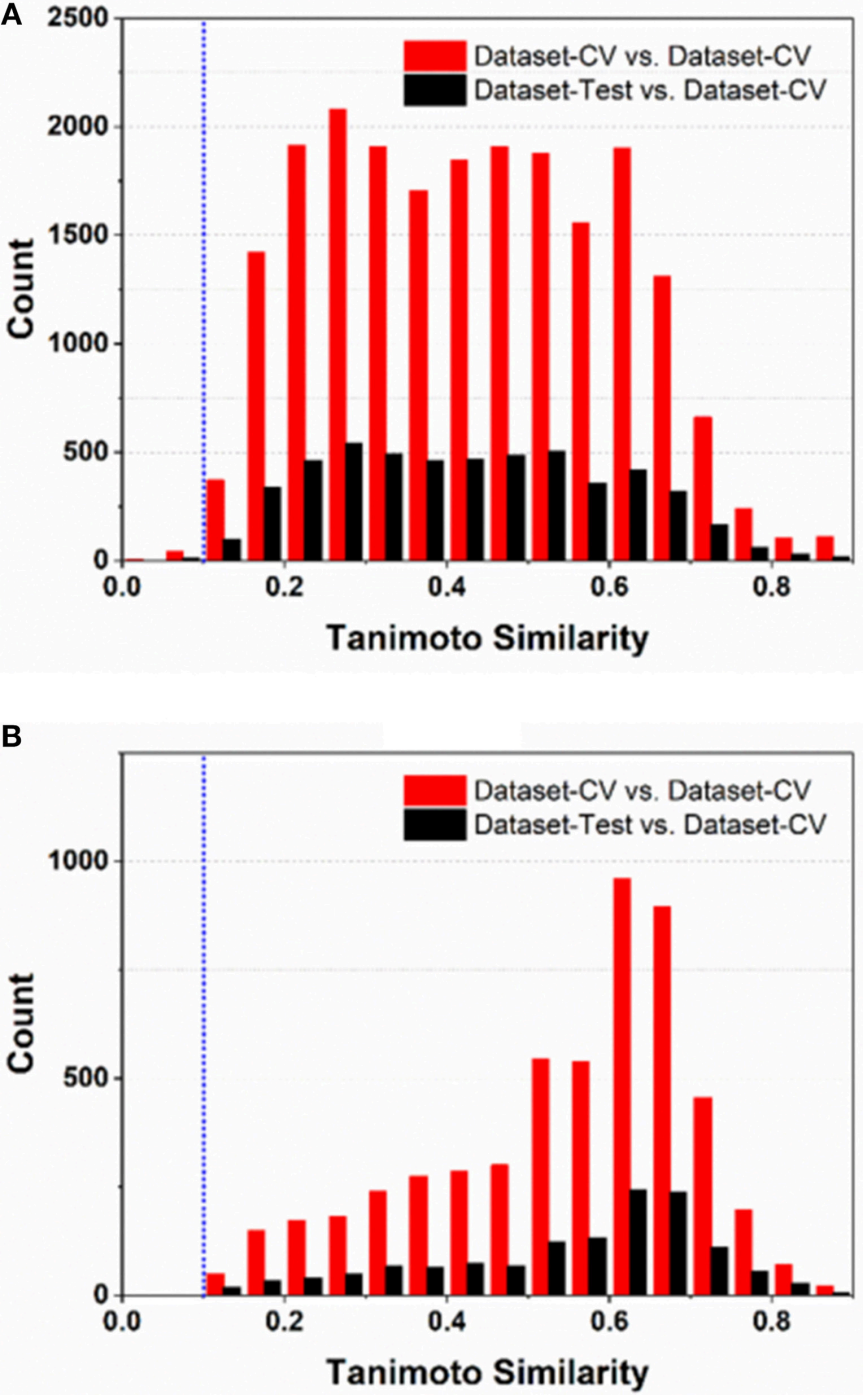
The histograms of average-similarity are utilized to define the applicability-domain of our classification **(A)** and regression models **(B)**. Both average-similarity thresholds of 0.1 are defined and implemented in our e-Sweet platform to automatically check whether the compound to be predicted is within the applicability domain of our models.

To comply the guideline of OECD, the applicability domain of the models should be defined appropriately. In this work, the applicability domain of our models is defined on the basis of the concept “average-similarity.” More Concretely , the automatic procedure implemented in our e-Sweet is given as follows: (1) each compound in the test set (Dataset-Test) is compared with all the compounds in the cross-validation dataset (Dataset-CV) according to the Tanimoto-similarity based on 2048bit-ECFP6; (2) five most similar compounds from Dataset-CV are retrieved and treated as five nearest neighbors for the given compound in Dataset-Test, and the average of five similarities is defined as the “average-similarity” between this given compound and these five nearest neighbors; (3) each compound in Dataset-Test retrieves five nearest neighbors in Dataset-CV to calculate the average-similarity. Similarly, each compound in Dataset-CV also finds five nearest neighbors in Dataset-CV to compute its corresponding average-similarity; (4) the histograms of the average-similarity for Dataset-Test and Dataset-CV are given in Figure 4 to address the applicability domain of our models.

For the classification model, Figure 4A shows that the average-similarity of 0.1 could be used as the threshold for the definition of the applicability domain of our classification models. If the average-similarity of the compound of interest is larger than this threshold (0.1), it means that this compound is located inside the applicability domain of our models, the prediction for this compound is a confident inference. Otherwise, this prediction may be a bold extrapolation. Similarly, for the regression model, Figure 4B reveals that the average-similarity of 0.1 can also serve as the threshold to define the applicability domain of our regression models. In order to automatically check whether the compound to be predicted is within the applicability domain of our classification and regression models, we have implemented a convenient function via simple clicking of the menu in our e-Sweet platform.

In brief, our classification and regression models for the prediction of sweetener and its RS have the pragmatically defined applicability domain, which is not commonly or explicitly mentioned in other relevant works and can be automatically inspected by our e-Sweet.

### Model Interpretation for our Classification and Regression Models in e-Sweet

Model interpretation suggested by OECD, will be considered based on the feature importance, which underscores the importance of each ECFP fingerprint bit contributing to the sweeteners/non-sweeteners classification or the regression of RS. Our e-Sweet platform can advantageously offer the appealing function to synchronously visualize the structural feature in the context of 3D structure and the associated feature importance for the ECFP fingerprint bit “1.”

In order to visualize the structural features and the corresponding feature importance for all the bits in ECFP, it would be better to adopt the model trained with the full features, since the feature selection will obviously lose some ECFP bits and hamper us to view the complete bits. Hence the average feature importance, which is from the average classification (AM22 in Table S3) and regression model (AM22 in Table S12) trained with RF and full features (2048bit-ECFP6), is embedded in our e-Sweet for the fully interactive visualization of ECFP fingerprint-bit, structural feature, and feature importance of ECFP bit.

For the purpose of concise demonstration, visualization of the feature importance (FI) contributing to the sweeteners/non-sweeteners classification is taken as an example and only the structural feature with the largest feature importance is considered here. In this case, the bit with the largest feature importance (FI = 0.019821) is 1138-bit (Figure S16). In our sweeteners/non-sweeteners dataset, the ECFPs of 228 sweeteners and 20 non-sweeteners contain the “1” in the 1138-bit. Here only one sweet molecule containing “1” in 1138-bit is taken as an instance for the better illustration (Figure S16). The structure feature for 1138-bit is highlighted with the yellow color in the 3D viewer window, the corresponding feature importance for 1138-bit is shown in the window titled “FI.” Based on the feature importance, it means that 1138-bit is very important for the sweeteners/non-sweeteners classification.

Concisely, our e-Sweet platform provide a convenient and intuitive visualization function for the model interpretation, which makes our classification and regression models fully conform to the OECD guidelines.

### The Limitation and Prospect of This Work

Admittedly, our work has some shortcomings. (1) Our curated dataset only considers the organic compounds, ignores the inorganic compounds and mixtures, and also neglects the effects of purity, moisture content, and temperature. In addition, the sweet taste assessment results given by the trained taste panelists have some inevitable noise, because the taste panelists possess some subjective factors (e.g., some mixed tastes that are very difficult to be clearly discriminated in qualitative or quantitative manner) and objective reasons (e.g., the individual gene-polymorphism of sweet taste receptor). (2) The consensus strategy is used to balance the pros and cons of each machine-learning method. However, it will bring some extra computational burden, because the final prediction is obtained by averaging over all the prediction results from each constituent model. (3) Applicability domain of the regression model for the relative sweetness is still limited, because the size of dataset for the regression model is relatively small compared to the size of sweetener/non-sweetener dataset for the classification and thereby needs further expansion.

In spite of these limitations, our work also possesses several advantages, which may provide some beneficial advice for the other researchers to develop more informative sweetener/sweetness prediction model. (1) Different data-splitting schemes have dramatic effects on the model training and model performance, which will be more obvious for the dataset with the limited size. Hence the multiple data-splitting schemes are highly recommended. (2) 2D descriptors based models are preferred over 3D descriptors based models for the practical prediction, because the sweeteners are usually very flexible molecules with diverse conformations that will cause the irreproducible outcome for the 3D descriptors based models. (3) The model evaluation solely based on R^2^(test set) or F1-score(test set) may be not convincing enough. Thus, it is suggested to consider both R^2^(test set) and |R^2^(test set)-R^2^(CV)| for the regression models and both F1-score(test set) and |F1-score(test set)-F1-score(CV)| for the classification model, since the model probably suffers from the over-fitting or under-fitting if |R^2^(test set)-R^2^(CV)| or |F1-score(test set)-F1-score(CV)| is large. (4) Deep neural network (DNN) method affords the consensus regression model **CM03** with the best agreement between R^2^(test set) and R^2^(CV) compared to all the models from other works. Thus, more exhaustive parameter optimization for DNN may offer a very good venue to further enhance the model performance, although there are so many hyper-parameters in DNN. (5) Consensus strategy is suggested to balance the pros and cons of each machine learning based model. (6) The full compliance with OECD guideline including the intuitive model interpretation and defined applicability domain of the model is strongly recommended. (7) Software development with the in-depth encapsulation of prediction model, fingerprint generation, and feature selection in the automatic manner is also very important for other users to apply the prediction model to their projects.

In the near future, we envision that the machine-learning based sweetener/sweetness prediction will become more and more effective and pragmatic, if it can be seamlessly fused with the other computational methods and experimental techniques. In our opinions, the performance of machine learning based model is heavily reliant on the initial high-quality dataset, which can be sustainably extended by the experimental high-throughput screening on the sweet taste receptor. Moreover, the machine-learning based sweetener/sweetness prediction belongs to the ligand-based approach and is expected to further combine with the structure-based sweetener prediction such as the molecular dynamics simulation, free energy calculation with the enhanced sampling or molecular docking methods on the basis of the modeled 3D structure of sweet taste receptor, although solving the crystal structure of the sweet taste receptor remains challenging so far. Thus, in the near future, the in-depth integration of machine-learning based sweetener/sweetness prediction, structure-based sweetener/sweetness prediction, and the experimental high-throughput screening based on the sweet taste receptor will provide a good paradigm for the discovery and development of novel sweeteners.

## Conclusion

In this work, we present a machine-learning based platform “e-Sweet,” which is developed for the experimental food scientists to automatically predict the sweetener and its corresponding RS. This platform provides several advantageous functions. (1) Users can visualize and inquiry our curated datasets that are all publicly available; (2) Four consensus sweetener/non-sweetener classification models in e-Sweet, derived from the largest dataset (530 sweeteners and 850 non-sweeteners), offer the best performance with the 95% confidence intervals for the accuracy (0.91 ± 0.01), precision (0.90 ± 0.01), specificity (0.94 ± 0.01), sensitivity (0.86 ± 0.01), F1-score (0.88 ± 0.01), MCC (0.81 ± 0.01), NER (0.90 ± 0.01), and ΔNER (0.03 ± 0.01), respectively on the test set, and prominently outperforms the results from the work of Rojas et al. (NER = 0.85); (3) The RS prediction model is harvested on the basis of the second largest dataset (352 sweeteners with the RS) and gives the robust outcome with the 95% confidence intervals for the R^2^(test set) and ΔR^2^ of 0.77 ± 0.01 and 0.03 ± 0.01, respectively, which is also better than other works based on the conformation-independent 2D molecular descriptors in terms of both R^2^(test set) and ΔR^2^. (4) Both the classification and regression models are trained with the multiple machine-learning methods and fully comply with the guidelines of OECD. (5) Interactive visualization of fingerprint bit, 3D structural feature, and feature importance. Therefore, we hope that this comprehensive platform can enable the experimental food scientists to exploit the machine-learning methods to boost the discovery and development of more novel sweeteners with the high potency.

## Author Contributions

SZ wrote the manuscript, SZ and WC performed the calculations and analysis. WC and WX curated the dataset and test the program, YX and FL revised the manuscript. SZ and FL conceived the workflow and developed the program.

### Conflict of Interest Statement

The authors declare that the research was conducted in the absence of any commercial or financial relationships that could be construed as a potential conflict of interest.
