# Supplementary material for: e-Sweet: A Machine-Learning Based Platform for the Prediction of Sweetener and Its Relative Sweetness
Source: Front Chem. 2019 Jan 30;7:35. doi: 10.3389/fchem.2019.00035 (PMC6363693; doi:10.3389/fchem.2019.00035)
Supplement: Supplementary file 1 [file Table_1.docx]

e-Sweet: a Machine-learning Based Platform for the Prediction of Sweetener and its Relative Sweetness

**Suqing Zheng**^1,2*^**, Wenping Chang**^1^**, Wenxin Xu**^1^**, Yong Xu**^3^**, and Fu Lin**^1*^

^1^: *School of Pharmaceutical Sciences, Wenzhou Medical University, Wenzhou, Zhejiang, P. R. China, 325035*

^2^: *Chemical Biology Research Center, Wenzhou Medical University, Wenzhou, Zhejiang, P. R. China, 325035*

^3^: *Center of Chemical Biology, Guangzhou Institutes of Biomedicine and Health, Chinese Academy of Sciences, Guangzhou, Guangdong, P. R. China, 510530*

*Corresponding authors:

Suqing Zheng (Email: [zsq_2016@126.com](mailto:zsq_2016@126.com))

Fu Lin (Email: [lin1449@126.com](mailto:lin1449@126.com) )

| **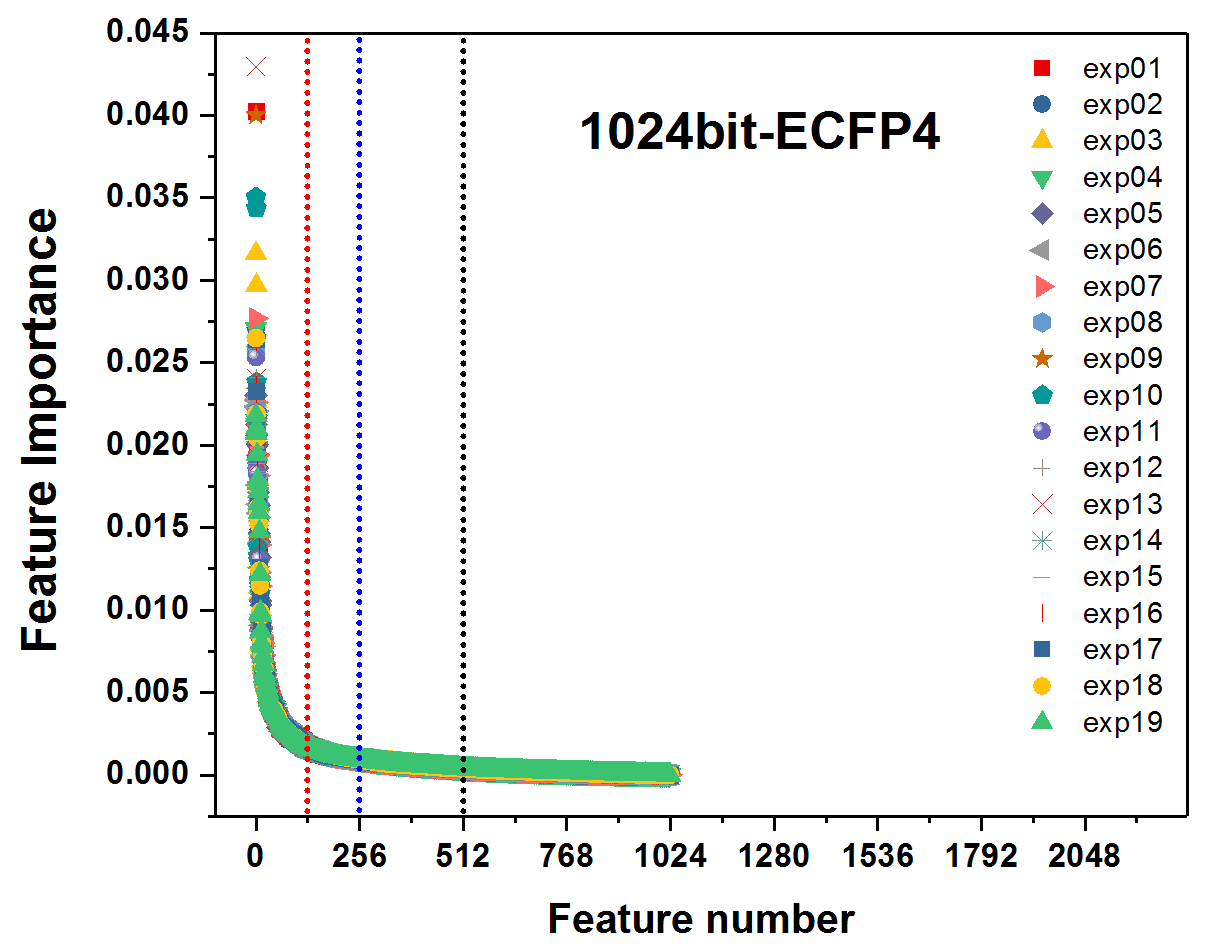** |
| --- |
| **Figure S1.** Nineteen sets of the feature importance derived from the random forest method with 1024bit-ECFP4. |

| **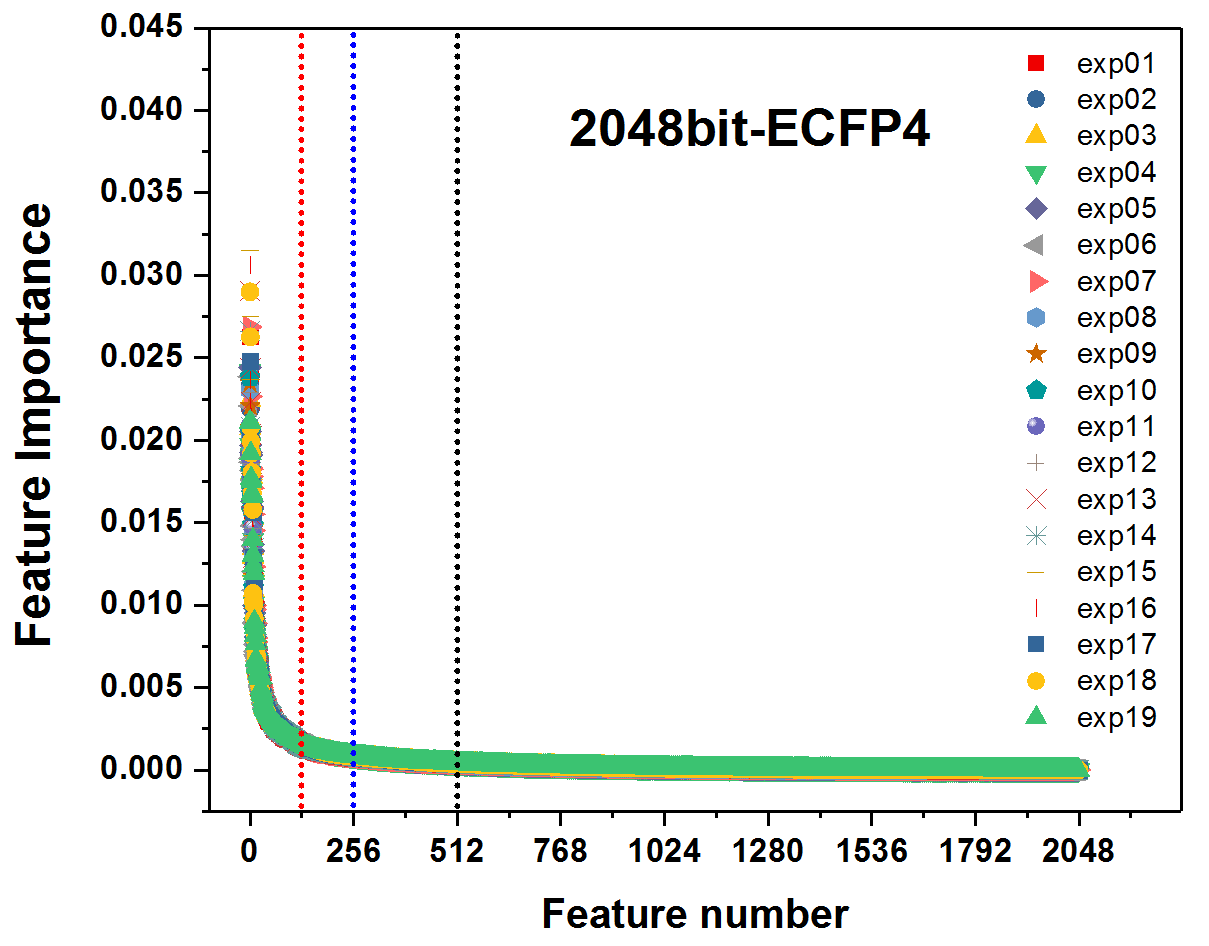** |
| --- |
| **Figure S2.** Nineteen sets of the feature importance derived from the random forest method with 2048bit-ECFP4. |

| **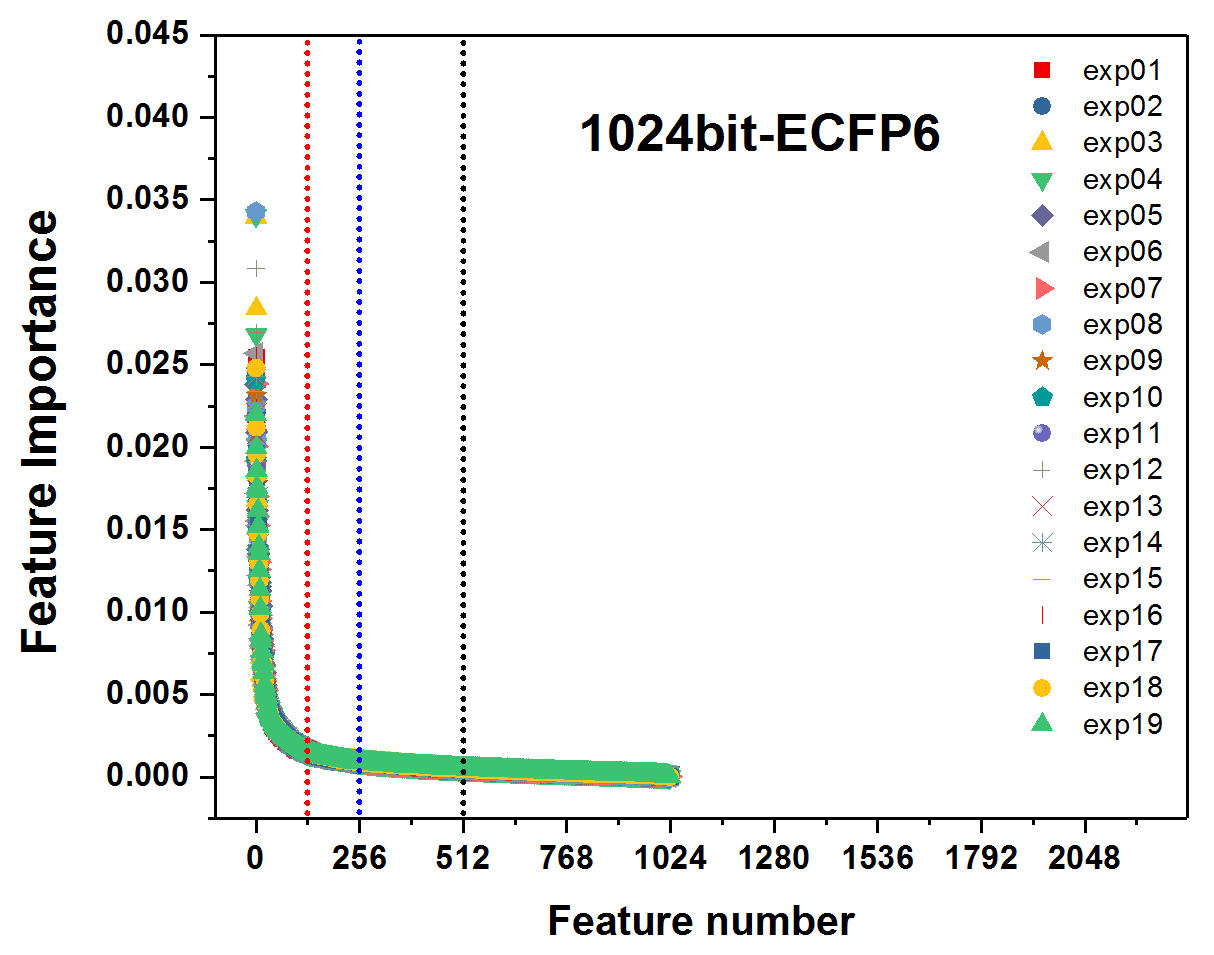** |
| --- |
| **Figure S3.** Nineteen sets of the feature importance derived from the random forest method with 1024bit-ECFP6. |

| **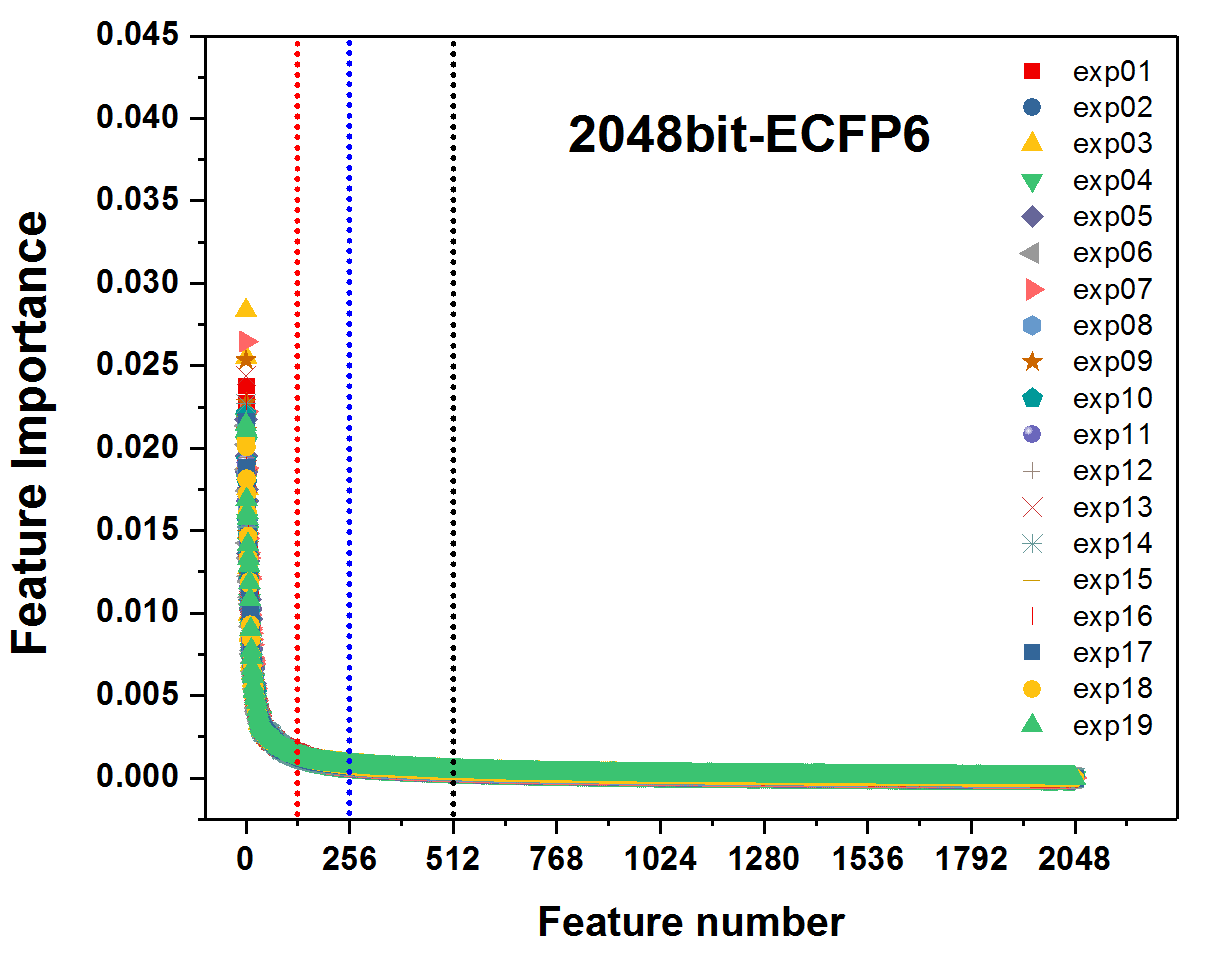** |
| --- |
| **Figure S4.** Nineteen sets of the feature importance derived from the random forest method with 2048bit-ECFP6. |

| **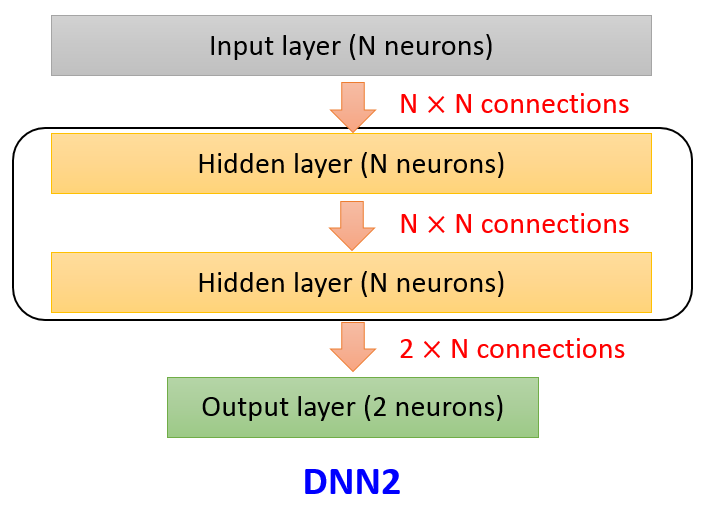** |
| --- |
| **Figure S5.** The configuration of DNN2 with two hidden layers. N in the figure refers to the number neurons and is set to the feature number in this work. |

| **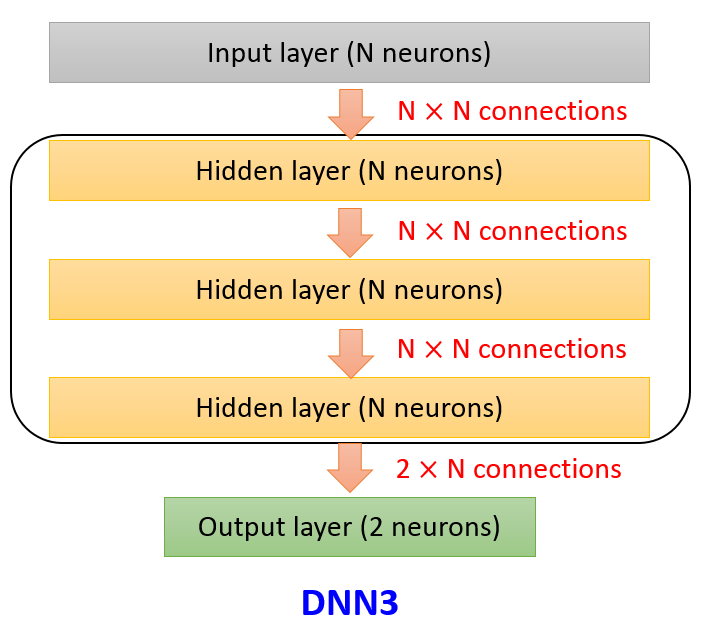** |
| --- |
| **Figure S6.** The configuration of DNN3 with three hidden layers. N in the figure refers to the number neurons and is set to the feature number in this work. |

| 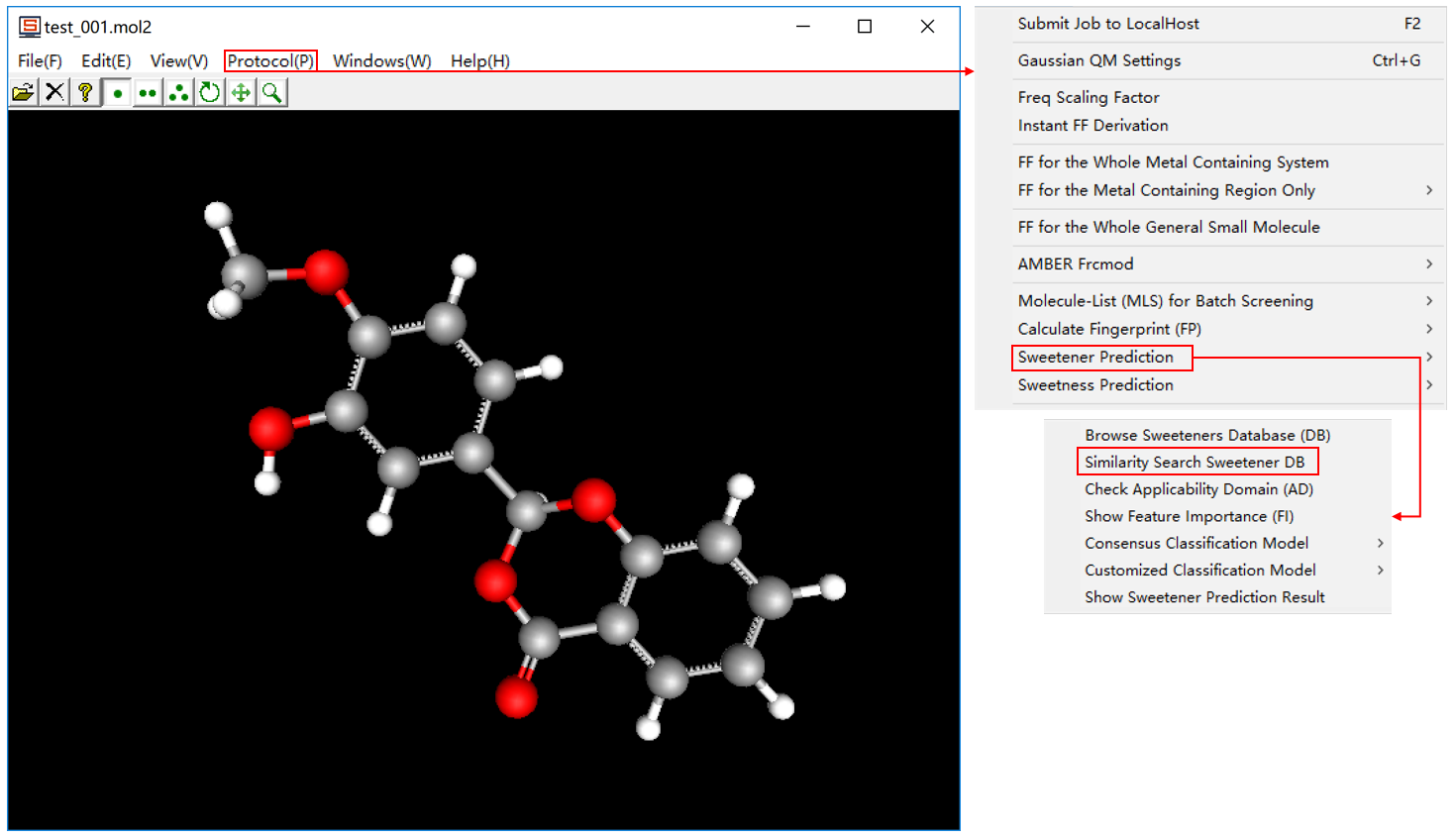 |
| --- |
| 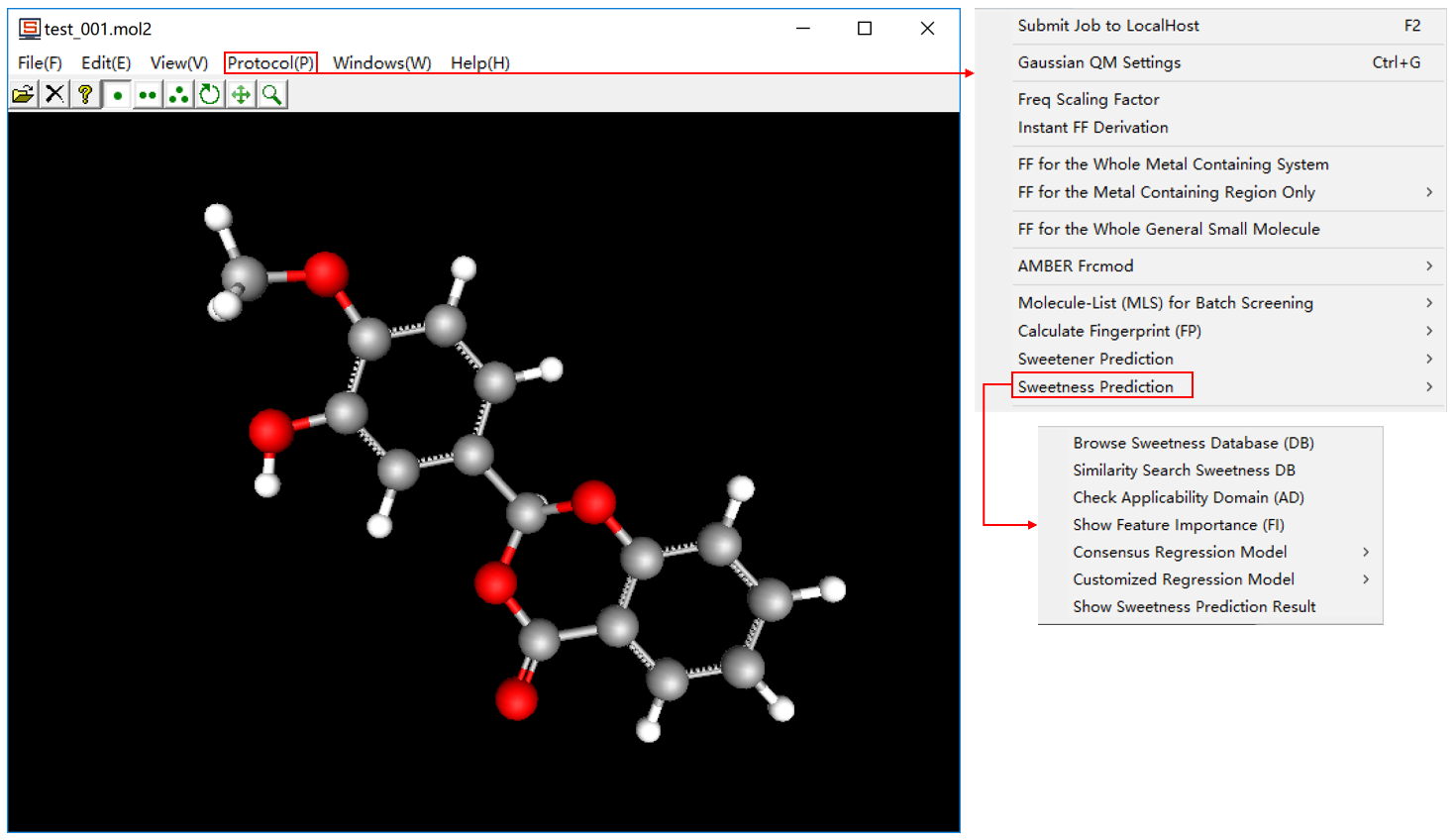 |
| **Figure S7.** The interface in e-Sweet platform used for visualizing and inquiring our curated datasets for the classification of sweetener/non-sweetener and the regression of relative sweetness. |

| **** |
| --- |
| **Figure S8.** The scatter plot for logP vs. MW for the dataset including 850 non-sweeteners and 530 sweeteners in the classification task. |

| **** |
| --- |
| **Figure S9.** The scatter plot for N_HBD_ vs. N_HBA_ for the dataset including 850 non-sweeteners and 530 sweeteners in the classification task. |

| **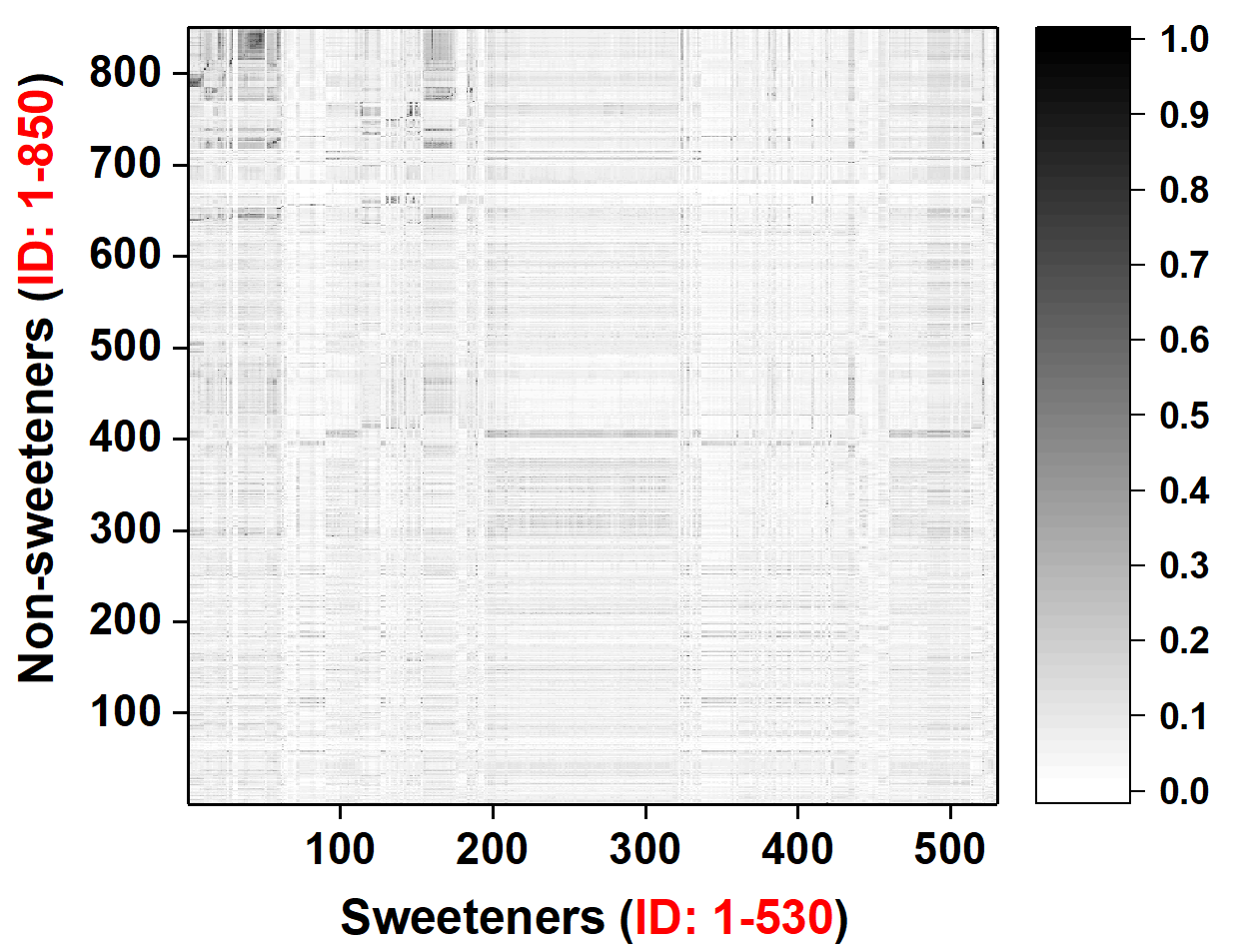** |
| --- |
| **Figure S10.** Tanimoto similarity matrix for the sweeteners vs. non-sweeteners. Similarity is calculated based on the 2048bit-ECFP6 fingerprint with our e-Sweet program. |

| **** |
| --- |
| **Figure S11.** The scatter plot of N_FRB_ vs. MW for the dataset including 352 sweeteners with relative sweetness in the regression task. Here N_FRB_ means that the number of freely rotatable bonds of sweetener. |

| **** |
| --- |
| **Figure S12.** The scatter plot of F1-score(test set) vs. MCC(test set) for all the classification models including all the individual and average models. |

| **** |
| --- |
| **Figure S13.** The scatter plot of R^2^(test set) vs. MSE(test set) for all the regression models including all the individual and average models. |

| 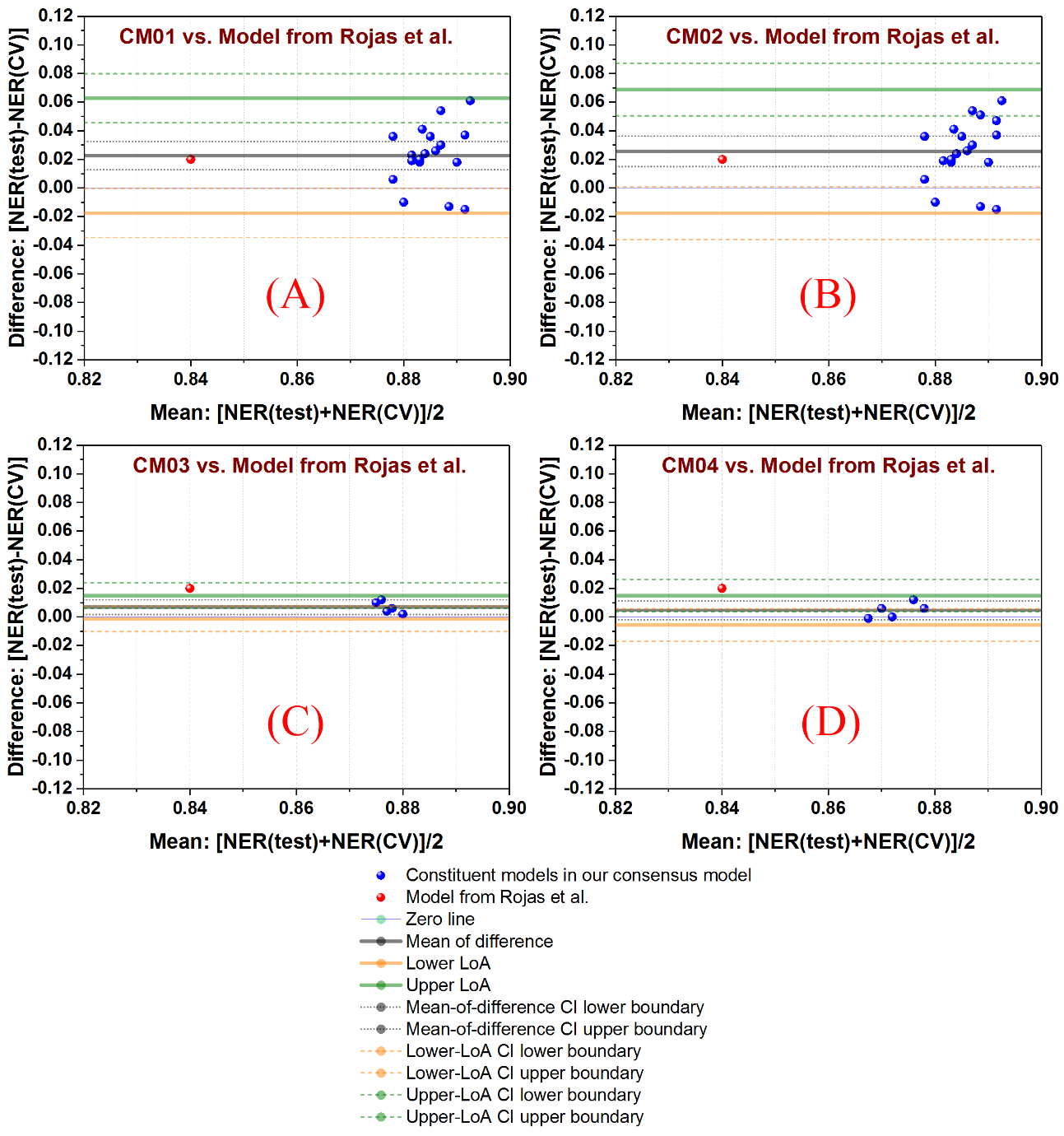 |
| --- |
| **Figure S14.** Bland-Altman plots of NER(test) and NER(CV) for our consensus classification models (**CM01**, **CM02**, **CM03** and **CM04**). NER(test) and NER(CV) are short for NER(test set) and NER(cross-validation) respectively; CI refers to the 95% confidence interval; LoA stands for the limit of agreement, which is defined by the Mean of difference plus/minus 1.96*SD (Standard Deviation) at the confidence level of 95%. |

| 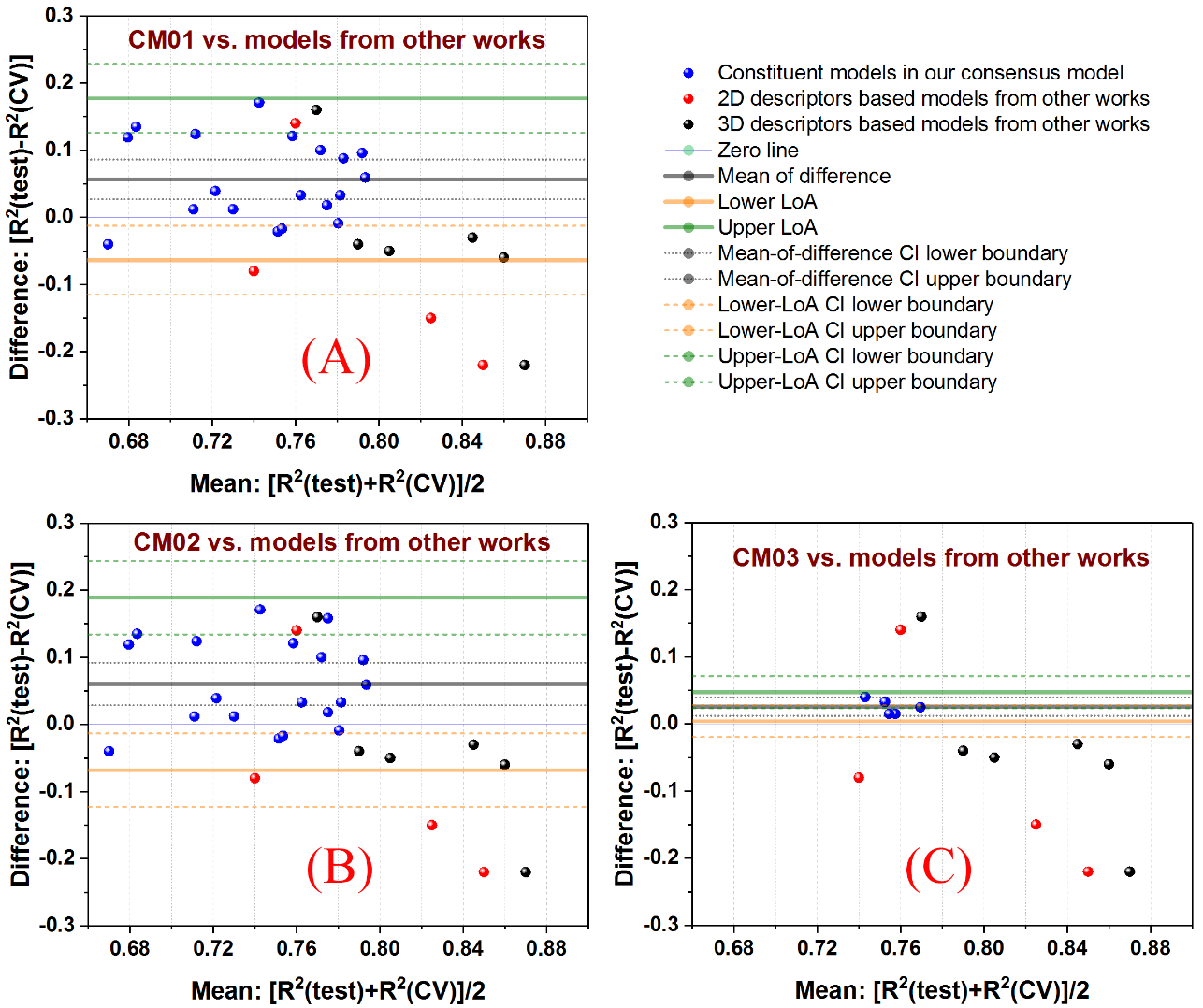 |
| --- |
| **Figure S15.** Bland-Altman plots of R^2^(test) and R^2^(CV) for our consensus regression models (**CM01**, **CM02**, and **CM03**). R^2^(test) and R^2^(CV) are short for R^2^(test set) and R^2^(cross-validation) respectively; CI refers to 95% confidence interval; LoA stands for the limit of agreement, which is defined by the mean of difference plus/minus 1.96*SD (Standard Deviation) at the confidence level of 95%. |

| 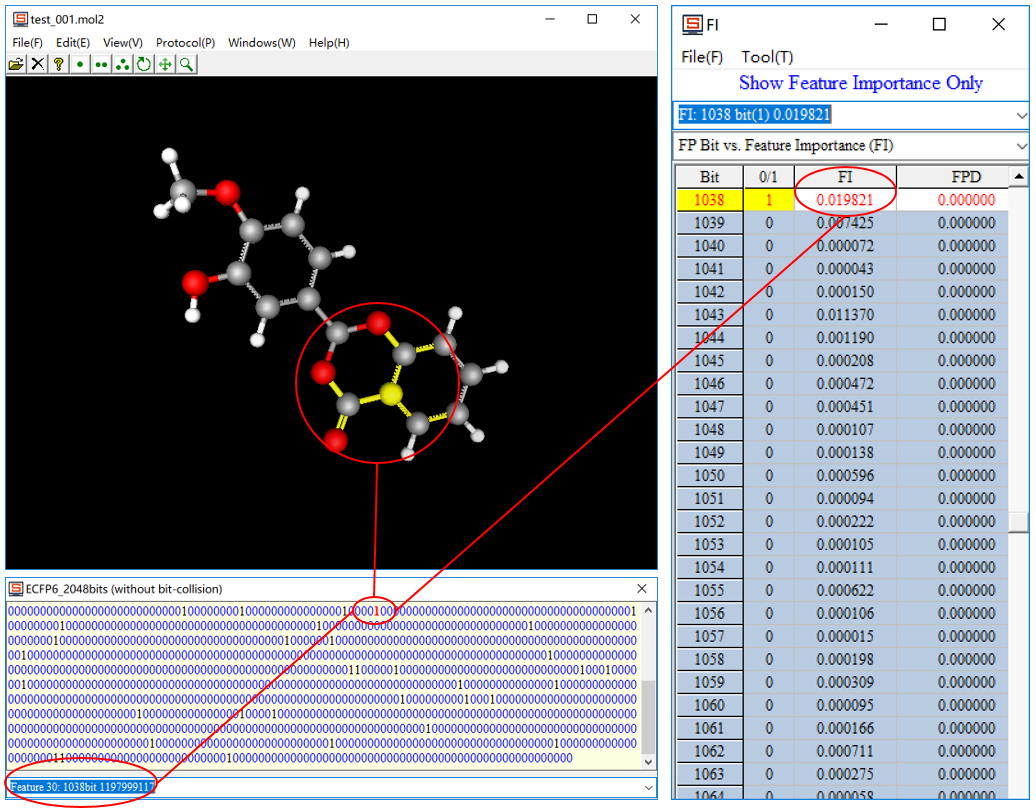 |
| --- |
| **Figure S16.** Interactive visualization of the feature importance (FI) in our e-Sweet platform. |

**Table S1.** The parameters for five machine-learning methods that need to be fine-tuned in the cross-validation

| **Machine-learning methods** | **Parameters** |
| --- | --- |
| KNN | Number of nearest neighbors (K): 1, 3, 5, 7, 9, 11, 13, and 15  Weighting schemes: uniform weight or distance-dependent weight |
| SVM | Kernel: RBF  Penalty parameter C: 1,000, 5,000, 10,000, 50,000, and 100,000  Kernel parameter gamma: 0.0001, 0.0005, 0.001, 0.005, 0.01, and 0.1 |
| RF | Number of decision trees: 10, 50, 100, 200, 300, 400, 500, 600, 700, 800, 900, and 1,000 |
| GBM | Number of decision trees: 10, 50, 100, 200, 300, 400, 500, 600, 700, 800, 900, and 1,000  Learning rate: 0.1, 0.2, 0.3, 0.4, 0.5, 0.6, 0.7, 0.8, and 0.9 |
| DNN | Activation function: rectified linear unit function (ReLU)  Optimizer: adam algorithm  Loss function: binary cross-entropy  Number of epochs: 100, 200, 300, 400, 500, and 600  Size of mini-batches: 60, 80, 100, 120, 140, and 160  Dropout rate: 0.1, 0.2, 0.3, 0.4, and 0.5 |

**Table S2.** All the individual classification models in this work

| **Model** | **FS** | **EXP** | **FP** | **Method** | **Accuracy**  **(test)** | **Precision**  **(test)** | **Specificity**  **(test)** | **Sensitivity**  **(test)** | **F1-score**  **(test)** | **MCC**  **(test)** | **NER**  **(test)** | **F1-score**  **(CV)** | **∆F1-score** | **Parameters from CV*** |
| --- | --- | --- | --- | --- | --- | --- | --- | --- | --- | --- | --- | --- | --- | --- |
| M0001 | full | exp01 | 1024bit-ECFP4 | KNN | 0.862 | 0.833 | 0.900 | 0.802 | 0.817 | 0.707 | 0.851 | 0.835 | 0.018 | 3 uniform |
| M0002 | full | exp02 | 1024bit-ECFP4 | KNN | 0.848 | 0.808 | 0.882 | 0.792 | 0.800 | 0.677 | 0.837 | 0.826 | 0.026 | 5 uniform |
| M0003 | full | exp03 | 1024bit-ECFP4 | KNN | 0.880 | 0.841 | 0.900 | 0.849 | 0.845 | 0.748 | 0.875 | 0.818 | 0.027 | 3 uniform |
| M0004 | full | exp04 | 1024bit-ECFP4 | KNN | 0.877 | 0.790 | 0.847 | 0.925 | 0.852 | 0.754 | 0.886 | 0.812 | 0.040 | 3 uniform |
| M0005 | full | exp05 | 1024bit-ECFP4 | KNN | 0.822 | 0.732 | 0.806 | 0.849 | 0.786 | 0.641 | 0.828 | 0.829 | 0.043 | 3 distance |
| M0006 | full | exp06 | 1024bit-ECFP4 | KNN | 0.888 | 0.832 | 0.888 | 0.887 | 0.858 | 0.767 | 0.887 | 0.812 | 0.046 | 3 distance |
| M0007 | full | exp07 | 1024bit-ECFP4 | KNN | 0.880 | 0.841 | 0.900 | 0.849 | 0.845 | 0.748 | 0.875 | 0.822 | 0.023 | 3 distance |
| M0008 | full | exp08 | 1024bit-ECFP4 | KNN | 0.899 | 0.868 | 0.918 | 0.868 | 0.868 | 0.786 | 0.893 | 0.813 | 0.055 | 5 distance |
| M0009 | full | exp09 | 1024bit-ECFP4 | KNN | 0.837 | 0.752 | 0.824 | 0.858 | 0.802 | 0.669 | 0.841 | 0.822 | 0.020 | 5 uniform |
| M0010 | full | exp10 | 1024bit-ECFP4 | KNN | 0.870 | 0.850 | 0.912 | 0.802 | 0.825 | 0.722 | 0.857 | 0.808 | 0.017 | 7 distance |
| M0011 | full | exp11 | 1024bit-ECFP4 | KNN | 0.844 | 0.779 | 0.853 | 0.830 | 0.804 | 0.676 | 0.841 | 0.842 | 0.038 | 3 distance |
| M0012 | full | exp12 | 1024bit-ECFP4 | KNN | 0.891 | 0.865 | 0.918 | 0.849 | 0.857 | 0.770 | 0.883 | 0.827 | 0.030 | 5 uniform |
| M0013 | full | exp13 | 1024bit-ECFP4 | KNN | 0.870 | 0.773 | 0.829 | 0.934 | 0.846 | 0.745 | 0.881 | 0.806 | 0.040 | 3 uniform |
| M0014 | full | exp14 | 1024bit-ECFP4 | KNN | 0.848 | 0.762 | 0.829 | 0.877 | 0.816 | 0.692 | 0.853 | 0.819 | 0.003 | 9 distance |
| M0015 | full | exp15 | 1024bit-ECFP4 | KNN | 0.841 | 0.758 | 0.829 | 0.858 | 0.805 | 0.675 | 0.843 | 0.817 | 0.012 | 1 uniform |
| M0016 | full | exp16 | 1024bit-ECFP4 | KNN | 0.844 | 0.812 | 0.888 | 0.774 | 0.792 | 0.668 | 0.831 | 0.824 | 0.032 | 5 uniform |
| M0017 | full | exp17 | 1024bit-ECFP4 | KNN | 0.855 | 0.784 | 0.853 | 0.858 | 0.820 | 0.701 | 0.855 | 0.817 | 0.003 | 3 distance |
| M0018 | full | exp18 | 1024bit-ECFP4 | KNN | 0.859 | 0.796 | 0.865 | 0.849 | 0.822 | 0.706 | 0.857 | 0.826 | 0.004 | 5 distance |
| M0019 | full | exp19 | 1024bit-ECFP4 | KNN | 0.899 | 0.861 | 0.912 | 0.877 | 0.869 | 0.786 | 0.895 | 0.811 | 0.058 | 5 distance |
| M0020 | full | exp01 | 1024bit-ECFP4 | SVM | 0.899 | 0.924 | 0.959 | 0.802 | 0.859 | 0.785 | 0.881 | 0.847 | 0.012 | 5 0.01 |
| M0021 | full | exp02 | 1024bit-ECFP4 | SVM | 0.884 | 0.885 | 0.935 | 0.802 | 0.842 | 0.753 | 0.869 | 0.847 | 0.005 | 500 0.05 |
| M0022 | full | exp03 | 1024bit-ECFP4 | SVM | 0.906 | 0.908 | 0.947 | 0.840 | 0.873 | 0.800 | 0.893 | 0.842 | 0.031 | 100 0.05 |
| M0023 | full | exp04 | 1024bit-ECFP4 | SVM | 0.902 | 0.869 | 0.918 | 0.877 | 0.873 | 0.794 | 0.897 | 0.833 | 0.040 | 10 0.05 |
| M0024 | full | exp05 | 1024bit-ECFP4 | SVM | 0.873 | 0.845 | 0.906 | 0.821 | 0.833 | 0.731 | 0.863 | 0.841 | 0.008 | 5 0.05 |
| M0025 | full | exp06 | 1024bit-ECFP4 | SVM | 0.880 | 0.876 | 0.929 | 0.802 | 0.837 | 0.745 | 0.866 | 0.849 | 0.012 | 1000.0 0.05 |
| M0026 | full | exp07 | 1024bit-ECFP4 | SVM | 0.902 | 0.916 | 0.953 | 0.821 | 0.866 | 0.792 | 0.887 | 0.848 | 0.018 | 50 0.05 |
| M0027 | full | exp08 | 1024bit-ECFP4 | SVM | 0.888 | 0.921 | 0.959 | 0.774 | 0.841 | 0.762 | 0.867 | 0.846 | 0.005 | 1 0.05 |
| M0028 | full | exp09 | 1024bit-ECFP4 | SVM | 0.862 | 0.833 | 0.900 | 0.802 | 0.817 | 0.707 | 0.851 | 0.843 | 0.026 | 100 0.05 |
| M0029 | full | exp10 | 1024bit-ECFP4 | SVM | 0.899 | 0.882 | 0.929 | 0.849 | 0.865 | 0.784 | 0.889 | 0.834 | 0.031 | 5 0.01 |
| M0030 | full | exp11 | 1024bit-ECFP4 | SVM | 0.880 | 0.876 | 0.929 | 0.802 | 0.837 | 0.745 | 0.866 | 0.851 | 0.014 | 1 0.05 |
| M0031 | full | exp12 | 1024bit-ECFP4 | SVM | 0.913 | 0.902 | 0.941 | 0.868 | 0.885 | 0.815 | 0.904 | 0.843 | 0.042 | 5 0.05 |
| M0032 | full | exp13 | 1024bit-ECFP4 | SVM | 0.884 | 0.849 | 0.906 | 0.849 | 0.849 | 0.755 | 0.877 | 0.834 | 0.015 | 5 0.01 |
| M0033 | full | exp14 | 1024bit-ECFP4 | SVM | 0.870 | 0.843 | 0.906 | 0.811 | 0.827 | 0.723 | 0.859 | 0.847 | 0.020 | 5000.0 0.05 |
| M0034 | full | exp15 | 1024bit-ECFP4 | SVM | 0.899 | 0.915 | 0.953 | 0.811 | 0.860 | 0.784 | 0.882 | 0.842 | 0.018 | 10 0.05 |
| M0035 | full | exp16 | 1024bit-ECFP4 | SVM | 0.862 | 0.886 | 0.941 | 0.736 | 0.804 | 0.707 | 0.839 | 0.839 | 0.035 | 50000.0 0.05 |
| M0036 | full | exp17 | 1024bit-ECFP4 | SVM | 0.888 | 0.879 | 0.929 | 0.821 | 0.849 | 0.761 | 0.875 | 0.831 | 0.018 | 100 0.05 |
| M0037 | full | exp18 | 1024bit-ECFP4 | SVM | 0.884 | 0.856 | 0.912 | 0.840 | 0.848 | 0.754 | 0.876 | 0.840 | 0.008 | 5 0.01 |
| M0038 | full | exp19 | 1024bit-ECFP4 | SVM | 0.913 | 0.927 | 0.959 | 0.840 | 0.881 | 0.815 | 0.899 | 0.838 | 0.043 | 5000.0 0.05 |
| M0039 | full | exp01 | 1024bit-ECFP4 | GBM | 0.873 | 0.908 | 0.953 | 0.745 | 0.819 | 0.731 | 0.849 | 0.842 | 0.023 | 300 0.2 |
| M0040 | full | exp02 | 1024bit-ECFP4 | GBM | 0.891 | 0.922 | 0.959 | 0.783 | 0.847 | 0.770 | 0.871 | 0.835 | 0.012 | 200 0.6 |
| M0041 | full | exp03 | 1024bit-ECFP4 | GBM | 0.880 | 0.876 | 0.929 | 0.802 | 0.837 | 0.745 | 0.866 | 0.831 | 0.006 | 400 0.3 |
| M0042 | full | exp04 | 1024bit-ECFP4 | GBM | 0.895 | 0.867 | 0.918 | 0.858 | 0.863 | 0.778 | 0.888 | 0.832 | 0.031 | 300 0.8 |
| M0043 | full | exp05 | 1024bit-ECFP4 | GBM | 0.880 | 0.869 | 0.924 | 0.811 | 0.839 | 0.745 | 0.868 | 0.843 | 0.004 | 500 0.1 |
| M0044 | full | exp06 | 1024bit-ECFP4 | GBM | 0.895 | 0.914 | 0.953 | 0.802 | 0.854 | 0.777 | 0.877 | 0.851 | 0.003 | 200 0.4 |
| M0045 | full | exp07 | 1024bit-ECFP4 | GBM | 0.891 | 0.896 | 0.941 | 0.811 | 0.851 | 0.768 | 0.876 | 0.827 | 0.024 | 500 0.5 |
| M0046 | full | exp08 | 1024bit-ECFP4 | GBM | 0.895 | 0.897 | 0.941 | 0.821 | 0.857 | 0.776 | 0.881 | 0.842 | 0.015 | 300 0.9 |
| M0047 | full | exp09 | 1024bit-ECFP4 | GBM | 0.884 | 0.843 | 0.900 | 0.858 | 0.850 | 0.756 | 0.879 | 0.832 | 0.018 | 300 0.6 |
| M0048 | full | exp10 | 1024bit-ECFP4 | GBM | 0.884 | 0.894 | 0.941 | 0.792 | 0.840 | 0.753 | 0.867 | 0.833 | 0.007 | 100 0.4 |
| M0049 | full | exp11 | 1024bit-ECFP4 | GBM | 0.870 | 0.880 | 0.935 | 0.764 | 0.818 | 0.722 | 0.850 | 0.837 | 0.019 | 300 0.1 |
| M0050 | full | exp12 | 1024bit-ECFP4 | GBM | 0.902 | 0.876 | 0.924 | 0.868 | 0.872 | 0.793 | 0.896 | 0.808 | 0.064 | 700 0.3 |
| M0051 | full | exp13 | 1024bit-ECFP4 | GBM | 0.855 | 0.811 | 0.882 | 0.811 | 0.811 | 0.694 | 0.847 | 0.837 | 0.026 | 300 0.2 |
| M0052 | full | exp14 | 1024bit-ECFP4 | GBM | 0.859 | 0.838 | 0.906 | 0.783 | 0.810 | 0.699 | 0.845 | 0.840 | 0.030 | 400 0.1 |
| M0053 | full | exp15 | 1024bit-ECFP4 | GBM | 0.891 | 0.904 | 0.947 | 0.802 | 0.850 | 0.769 | 0.875 | 0.825 | 0.025 | 400 0.8 |
| M0054 | full | exp16 | 1024bit-ECFP4 | GBM | 0.866 | 0.897 | 0.947 | 0.736 | 0.808 | 0.715 | 0.841 | 0.824 | 0.016 | 500 0.6 |
| M0055 | full | exp17 | 1024bit-ECFP4 | GBM | 0.884 | 0.894 | 0.941 | 0.792 | 0.840 | 0.753 | 0.867 | 0.816 | 0.024 | 300 0.5 |
| M0056 | full | exp18 | 1024bit-ECFP4 | GBM | 0.895 | 0.897 | 0.941 | 0.821 | 0.857 | 0.776 | 0.881 | 0.838 | 0.019 | 800 0.1 |
| M0057 | full | exp19 | 1024bit-ECFP4 | GBM | 0.891 | 0.896 | 0.941 | 0.811 | 0.851 | 0.768 | 0.876 | 0.826 | 0.025 | 200 0.1 |
| M0058 | full | exp01 | 1024bit-ECFP4 | RF | 0.866 | 0.888 | 0.941 | 0.745 | 0.810 | 0.714 | 0.843 | 0.838 | 0.027 | 50 |
| M0059 | full | exp02 | 1024bit-ECFP4 | RF | 0.880 | 0.929 | 0.965 | 0.745 | 0.827 | 0.748 | 0.855 | 0.837 | 0.010 | 100 |
| M0060 | full | exp03 | 1024bit-ECFP4 | RF | 0.895 | 0.933 | 0.965 | 0.783 | 0.851 | 0.778 | 0.874 | 0.841 | 0.010 | 50 |
| M0061 | full | exp04 | 1024bit-ECFP4 | RF | 0.895 | 0.889 | 0.935 | 0.830 | 0.859 | 0.776 | 0.883 | 0.831 | 0.028 | 900 |
| M0062 | full | exp05 | 1024bit-ECFP4 | RF | 0.884 | 0.885 | 0.935 | 0.802 | 0.842 | 0.753 | 0.869 | 0.848 | 0.006 | 300 |
| M0063 | full | exp06 | 1024bit-ECFP4 | RF | 0.899 | 0.953 | 0.976 | 0.774 | 0.854 | 0.788 | 0.875 | 0.841 | 0.013 | 200 |
| M0064 | full | exp07 | 1024bit-ECFP4 | RF | 0.906 | 0.955 | 0.976 | 0.792 | 0.866 | 0.802 | 0.884 | 0.847 | 0.019 | 800 |
| M0065 | full | exp08 | 1024bit-ECFP4 | RF | 0.902 | 0.934 | 0.965 | 0.802 | 0.863 | 0.793 | 0.883 | 0.834 | 0.029 | 1000 |
| M0066 | full | exp09 | 1024bit-ECFP4 | RF | 0.899 | 0.915 | 0.953 | 0.811 | 0.860 | 0.784 | 0.882 | 0.830 | 0.030 | 50 |
| M0067 | full | exp10 | 1024bit-ECFP4 | RF | 0.880 | 0.910 | 0.953 | 0.764 | 0.831 | 0.746 | 0.859 | 0.827 | 0.004 | 10 |
| M0068 | full | exp11 | 1024bit-ECFP4 | RF | 0.884 | 0.902 | 0.947 | 0.783 | 0.838 | 0.753 | 0.865 | 0.847 | 0.009 | 800 |
| M0069 | full | exp12 | 1024bit-ECFP4 | RF | 0.920 | 0.957 | 0.976 | 0.830 | 0.889 | 0.832 | 0.903 | 0.828 | 0.061 | 100 |
| M0070 | full | exp13 | 1024bit-ECFP4 | RF | 0.902 | 0.883 | 0.929 | 0.858 | 0.871 | 0.792 | 0.893 | 0.836 | 0.035 | 50 |
| M0071 | full | exp14 | 1024bit-ECFP4 | RF | 0.866 | 0.856 | 0.918 | 0.783 | 0.818 | 0.714 | 0.851 | 0.856 | 0.038 | 500 |
| M0072 | full | exp15 | 1024bit-ECFP4 | RF | 0.899 | 0.933 | 0.965 | 0.792 | 0.857 | 0.786 | 0.879 | 0.836 | 0.021 | 500 |
| M0073 | full | exp16 | 1024bit-ECFP4 | RF | 0.870 | 0.889 | 0.941 | 0.755 | 0.816 | 0.722 | 0.848 | 0.839 | 0.023 | 100 |
| M0074 | full | exp17 | 1024bit-ECFP4 | RF | 0.888 | 0.912 | 0.953 | 0.783 | 0.843 | 0.761 | 0.868 | 0.838 | 0.005 | 400 |
| M0075 | full | exp18 | 1024bit-ECFP4 | RF | 0.884 | 0.902 | 0.947 | 0.783 | 0.838 | 0.753 | 0.865 | 0.841 | 0.003 | 500 |
| M0076 | full | exp19 | 1024bit-ECFP4 | RF | 0.909 | 0.926 | 0.959 | 0.830 | 0.876 | 0.808 | 0.894 | 0.844 | 0.032 | 300 |
| M0077 | full | exp01 | 1024bit-ECFP4 | DNN2 | 0.888 | 0.912 | 0.953 | 0.783 | 0.843 | 0.761 | 0.868 | 0.847 | 0.004 | 300 80 0.5 |
| M0078 | full | exp02 | 1024bit-ECFP4 | DNN2 | 0.895 | 0.914 | 0.953 | 0.802 | 0.854 | 0.777 | 0.877 | 0.856 | 0.002 | 600 120 0.3 |
| M0079 | full | exp03 | 1024bit-ECFP4 | DNN2 | 0.873 | 0.874 | 0.929 | 0.783 | 0.826 | 0.729 | 0.856 | 0.848 | 0.022 | 100 80 0.1 |
| M0080 | full | exp01 | 1024bit-ECFP4 | DNN3 | 0.880 | 0.901 | 0.947 | 0.774 | 0.832 | 0.746 | 0.861 | 0.845 | 0.013 | 500 160 0.5 |
| M0081 | full | exp02 | 1024bit-ECFP4 | DNN3 | 0.888 | 0.895 | 0.941 | 0.802 | 0.846 | 0.761 | 0.871 | 0.852 | 0.006 | 300 100 0.1 |
| M0082 | full | exp03 | 1024bit-ECFP4 | DNN3 | 0.877 | 0.875 | 0.929 | 0.792 | 0.832 | 0.737 | 0.861 | 0.849 | 0.017 | 200 100 0.2 |
| M0083 | full | exp01 | 2048bit-ECFP4 | KNN | 0.862 | 0.840 | 0.906 | 0.792 | 0.816 | 0.707 | 0.849 | 0.846 | 0.030 | 3 uniform |
| M0084 | full | exp02 | 2048bit-ECFP4 | KNN | 0.870 | 0.824 | 0.888 | 0.840 | 0.832 | 0.725 | 0.864 | 0.826 | 0.006 | 3 distance |
| M0085 | full | exp03 | 2048bit-ECFP4 | KNN | 0.877 | 0.853 | 0.912 | 0.821 | 0.837 | 0.738 | 0.867 | 0.822 | 0.015 | 7 distance |
| M0086 | full | exp04 | 2048bit-ECFP4 | KNN | 0.891 | 0.833 | 0.888 | 0.896 | 0.864 | 0.775 | 0.892 | 0.813 | 0.051 | 7 distance |
| M0087 | full | exp05 | 2048bit-ECFP4 | KNN | 0.844 | 0.769 | 0.841 | 0.849 | 0.807 | 0.679 | 0.845 | 0.832 | 0.024 | 3 distance |
| M0088 | full | exp06 | 2048bit-ECFP4 | KNN | 0.891 | 0.839 | 0.894 | 0.887 | 0.862 | 0.773 | 0.891 | 0.813 | 0.049 | 3 distance |
| M0089 | full | exp07 | 2048bit-ECFP4 | KNN | 0.855 | 0.806 | 0.876 | 0.821 | 0.813 | 0.695 | 0.849 | 0.833 | 0.020 | 9 distance |
| M0090 | full | exp08 | 2048bit-ECFP4 | KNN | 0.884 | 0.894 | 0.941 | 0.792 | 0.840 | 0.753 | 0.867 | 0.812 | 0.028 | 9 distance |
| M0091 | full | exp09 | 2048bit-ECFP4 | KNN | 0.844 | 0.760 | 0.829 | 0.868 | 0.811 | 0.684 | 0.849 | 0.817 | 0.006 | 3 distance |
| M0092 | full | exp10 | 2048bit-ECFP4 | KNN | 0.859 | 0.860 | 0.924 | 0.755 | 0.804 | 0.698 | 0.840 | 0.808 | 0.004 | 9 distance |
| M0093 | full | exp11 | 2048bit-ECFP4 | KNN | 0.848 | 0.786 | 0.859 | 0.830 | 0.807 | 0.682 | 0.845 | 0.838 | 0.031 | 3 uniform |
| M0094 | full | exp12 | 2048bit-ECFP4 | KNN | 0.888 | 0.857 | 0.912 | 0.849 | 0.853 | 0.762 | 0.881 | 0.826 | 0.027 | 5 uniform |
| M0095 | full | exp13 | 2048bit-ECFP4 | KNN | 0.870 | 0.782 | 0.841 | 0.915 | 0.843 | 0.739 | 0.878 | 0.812 | 0.031 | 3 uniform |
| M0096 | full | exp14 | 2048bit-ECFP4 | KNN | 0.841 | 0.758 | 0.829 | 0.858 | 0.805 | 0.675 | 0.843 | 0.831 | 0.026 | 9 distance |
| M0097 | full | exp15 | 2048bit-ECFP4 | KNN | 0.866 | 0.795 | 0.859 | 0.877 | 0.834 | 0.725 | 0.868 | 0.822 | 0.012 | 3 uniform |
| M0098 | full | exp16 | 2048bit-ECFP4 | KNN | 0.844 | 0.806 | 0.882 | 0.783 | 0.794 | 0.669 | 0.833 | 0.828 | 0.034 | 5 uniform |
| M0099 | full | exp17 | 2048bit-ECFP4 | KNN | 0.851 | 0.788 | 0.859 | 0.840 | 0.813 | 0.691 | 0.849 | 0.818 | 0.005 | 3 distance |
| M0100 | full | exp18 | 2048bit-ECFP4 | KNN | 0.862 | 0.815 | 0.882 | 0.830 | 0.822 | 0.710 | 0.856 | 0.832 | 0.010 | 5 distance |
| M0101 | full | exp19 | 2048bit-ECFP4 | KNN | 0.888 | 0.844 | 0.900 | 0.868 | 0.856 | 0.764 | 0.884 | 0.813 | 0.043 | 5 distance |
| M0102 | full | exp01 | 2048bit-ECFP4 | SVM | 0.888 | 0.921 | 0.959 | 0.774 | 0.841 | 0.762 | 0.867 | 0.853 | 0.012 | 10 0.005 |
| M0103 | full | exp02 | 2048bit-ECFP4 | SVM | 0.877 | 0.867 | 0.924 | 0.802 | 0.833 | 0.737 | 0.863 | 0.848 | 0.015 | 10 0.05 |
| M0104 | full | exp03 | 2048bit-ECFP4 | SVM | 0.884 | 0.863 | 0.918 | 0.830 | 0.846 | 0.754 | 0.874 | 0.850 | 0.004 | 10 0.01 |
| M0105 | full | exp04 | 2048bit-ECFP4 | SVM | 0.906 | 0.892 | 0.935 | 0.858 | 0.875 | 0.800 | 0.897 | 0.833 | 0.042 | 10 0.005 |
| M0106 | full | exp05 | 2048bit-ECFP4 | SVM | 0.877 | 0.833 | 0.894 | 0.849 | 0.841 | 0.741 | 0.871 | 0.852 | 0.011 | 5 0.01 |
| M0107 | full | exp06 | 2048bit-ECFP4 | SVM | 0.895 | 0.905 | 0.947 | 0.811 | 0.856 | 0.776 | 0.879 | 0.858 | 0.002 | 10 0.005 |
| M0108 | full | exp07 | 2048bit-ECFP4 | SVM | 0.895 | 0.914 | 0.953 | 0.802 | 0.854 | 0.777 | 0.877 | 0.854 | 0.000 | 5 0.01 |
| M0109 | full | exp08 | 2048bit-ECFP4 | SVM | 0.888 | 0.921 | 0.959 | 0.774 | 0.841 | 0.762 | 0.867 | 0.844 | 0.003 | 1 0.05 |
| M0110 | full | exp09 | 2048bit-ECFP4 | SVM | 0.884 | 0.849 | 0.906 | 0.849 | 0.849 | 0.755 | 0.877 | 0.845 | 0.004 | 10 0.01 |
| M0111 | full | exp10 | 2048bit-ECFP4 | SVM | 0.906 | 0.900 | 0.941 | 0.849 | 0.874 | 0.800 | 0.895 | 0.843 | 0.031 | 5 0.01 |
| M0112 | full | exp11 | 2048bit-ECFP4 | SVM | 0.888 | 0.887 | 0.935 | 0.811 | 0.847 | 0.761 | 0.873 | 0.862 | 0.015 | 5 0.01 |
| M0113 | full | exp12 | 2048bit-ECFP4 | SVM | 0.913 | 0.902 | 0.941 | 0.868 | 0.885 | 0.815 | 0.904 | 0.841 | 0.044 | 10 0.01 |
| M0114 | full | exp13 | 2048bit-ECFP4 | SVM | 0.884 | 0.836 | 0.894 | 0.868 | 0.852 | 0.757 | 0.881 | 0.838 | 0.014 | 100 0.005 |
| M0115 | full | exp14 | 2048bit-ECFP4 | SVM | 0.873 | 0.838 | 0.900 | 0.830 | 0.834 | 0.732 | 0.865 | 0.860 | 0.026 | 5 0.01 |
| M0116 | full | exp15 | 2048bit-ECFP4 | SVM | 0.902 | 0.925 | 0.959 | 0.811 | 0.864 | 0.792 | 0.885 | 0.845 | 0.019 | 500 0.05 |
| M0117 | full | exp16 | 2048bit-ECFP4 | SVM | 0.877 | 0.891 | 0.941 | 0.774 | 0.828 | 0.737 | 0.857 | 0.845 | 0.017 | 500 0.05 |
| M0118 | full | exp17 | 2048bit-ECFP4 | SVM | 0.895 | 0.897 | 0.941 | 0.821 | 0.857 | 0.776 | 0.881 | 0.840 | 0.017 | 5000.0 0.05 |
| M0119 | full | exp18 | 2048bit-ECFP4 | SVM | 0.891 | 0.873 | 0.924 | 0.840 | 0.856 | 0.769 | 0.882 | 0.847 | 0.009 | 10 0.01 |
| M0120 | full | exp19 | 2048bit-ECFP4 | SVM | 0.913 | 0.936 | 0.965 | 0.830 | 0.880 | 0.816 | 0.897 | 0.840 | 0.040 | 1 0.05 |
| M0121 | full | exp01 | 2048bit-ECFP4 | GBM | 0.866 | 0.863 | 0.924 | 0.774 | 0.816 | 0.714 | 0.849 | 0.838 | 0.022 | 400 0.1 |
| M0122 | full | exp02 | 2048bit-ECFP4 | GBM | 0.880 | 0.910 | 0.953 | 0.764 | 0.831 | 0.746 | 0.859 | 0.834 | 0.003 | 300 0.1 |
| M0123 | full | exp03 | 2048bit-ECFP4 | GBM | 0.873 | 0.851 | 0.912 | 0.811 | 0.831 | 0.730 | 0.862 | 0.833 | 0.002 | 1000 0.3 |
| M0124 | full | exp04 | 2048bit-ECFP4 | GBM | 0.873 | 0.882 | 0.935 | 0.774 | 0.824 | 0.729 | 0.855 | 0.814 | 0.010 | 50 0.1 |
| M0125 | full | exp05 | 2048bit-ECFP4 | GBM | 0.862 | 0.847 | 0.912 | 0.783 | 0.814 | 0.706 | 0.848 | 0.831 | 0.017 | 100 0.4 |
| M0126 | full | exp06 | 2048bit-ECFP4 | GBM | 0.895 | 0.933 | 0.965 | 0.783 | 0.851 | 0.778 | 0.874 | 0.831 | 0.020 | 400 0.2 |
| M0127 | full | exp07 | 2048bit-ECFP4 | GBM | 0.870 | 0.865 | 0.924 | 0.783 | 0.822 | 0.722 | 0.854 | 0.829 | 0.007 | 100 0.1 |
| M0128 | full | exp08 | 2048bit-ECFP4 | GBM | 0.880 | 0.901 | 0.947 | 0.774 | 0.832 | 0.746 | 0.861 | 0.830 | 0.002 | 100 0.3 |
| M0129 | full | exp09 | 2048bit-ECFP4 | GBM | 0.888 | 0.879 | 0.929 | 0.821 | 0.849 | 0.761 | 0.875 | 0.830 | 0.019 | 200 0.3 |
| M0130 | full | exp10 | 2048bit-ECFP4 | GBM | 0.902 | 0.925 | 0.959 | 0.811 | 0.864 | 0.792 | 0.885 | 0.829 | 0.035 | 200 0.1 |
| M0131 | full | exp11 | 2048bit-ECFP4 | GBM | 0.859 | 0.860 | 0.924 | 0.755 | 0.804 | 0.698 | 0.840 | 0.830 | 0.026 | 400 0.4 |
| M0132 | full | exp12 | 2048bit-ECFP4 | GBM | 0.902 | 0.934 | 0.965 | 0.802 | 0.863 | 0.793 | 0.883 | 0.813 | 0.050 | 50 0.2 |
| M0133 | full | exp13 | 2048bit-ECFP4 | GBM | 0.859 | 0.807 | 0.876 | 0.830 | 0.819 | 0.703 | 0.853 | 0.836 | 0.017 | 200 0.3 |
| M0134 | full | exp14 | 2048bit-ECFP4 | GBM | 0.851 | 0.822 | 0.894 | 0.783 | 0.802 | 0.684 | 0.839 | 0.834 | 0.032 | 100 0.4 |
| M0135 | full | exp15 | 2048bit-ECFP4 | GBM | 0.884 | 0.885 | 0.935 | 0.802 | 0.842 | 0.753 | 0.869 | 0.816 | 0.026 | 300 0.1 |
| M0136 | full | exp16 | 2048bit-ECFP4 | GBM | 0.873 | 0.890 | 0.941 | 0.764 | 0.822 | 0.730 | 0.853 | 0.819 | 0.003 | 100 0.5 |
| M0137 | full | exp17 | 2048bit-ECFP4 | GBM | 0.877 | 0.875 | 0.929 | 0.792 | 0.832 | 0.737 | 0.861 | 0.810 | 0.022 | 100 0.3 |
| M0138 | full | exp18 | 2048bit-ECFP4 | GBM | 0.870 | 0.830 | 0.894 | 0.830 | 0.830 | 0.724 | 0.862 | 0.840 | 0.010 | 200 0.8 |
| M0139 | full | exp19 | 2048bit-ECFP4 | GBM | 0.895 | 0.889 | 0.935 | 0.830 | 0.859 | 0.776 | 0.883 | 0.824 | 0.035 | 500 0.1 |
| M0140 | full | exp01 | 2048bit-ECFP4 | RF | 0.866 | 0.906 | 0.953 | 0.726 | 0.806 | 0.716 | 0.839 | 0.843 | 0.037 | 300 |
| M0141 | full | exp02 | 2048bit-ECFP4 | RF | 0.877 | 0.900 | 0.947 | 0.764 | 0.827 | 0.738 | 0.855 | 0.834 | 0.007 | 200 |
| M0142 | full | exp03 | 2048bit-ECFP4 | RF | 0.895 | 0.923 | 0.959 | 0.792 | 0.853 | 0.777 | 0.875 | 0.842 | 0.011 | 1000 |
| M0143 | full | exp04 | 2048bit-ECFP4 | RF | 0.891 | 0.880 | 0.929 | 0.830 | 0.854 | 0.769 | 0.879 | 0.832 | 0.022 | 1000 |
| M0144 | full | exp05 | 2048bit-ECFP4 | RF | 0.873 | 0.866 | 0.924 | 0.792 | 0.828 | 0.729 | 0.858 | 0.855 | 0.027 | 500 |
| M0145 | full | exp06 | 2048bit-ECFP4 | RF | 0.902 | 0.954 | 0.976 | 0.783 | 0.860 | 0.795 | 0.879 | 0.847 | 0.013 | 500 |
| M0146 | full | exp07 | 2048bit-ECFP4 | RF | 0.899 | 0.933 | 0.965 | 0.792 | 0.857 | 0.786 | 0.879 | 0.848 | 0.009 | 300 |
| M0147 | full | exp08 | 2048bit-ECFP4 | RF | 0.906 | 0.935 | 0.965 | 0.811 | 0.869 | 0.801 | 0.888 | 0.842 | 0.027 | 1000 |
| M0148 | full | exp09 | 2048bit-ECFP4 | RF | 0.899 | 0.915 | 0.953 | 0.811 | 0.860 | 0.784 | 0.882 | 0.821 | 0.039 | 500 |
| M0149 | full | exp10 | 2048bit-ECFP4 | RF | 0.902 | 0.934 | 0.965 | 0.802 | 0.863 | 0.793 | 0.883 | 0.826 | 0.037 | 700 |
| M0150 | full | exp11 | 2048bit-ECFP4 | RF | 0.870 | 0.889 | 0.941 | 0.755 | 0.816 | 0.722 | 0.848 | 0.845 | 0.029 | 800 |
| M0151 | full | exp12 | 2048bit-ECFP4 | RF | 0.917 | 0.946 | 0.971 | 0.830 | 0.884 | 0.824 | 0.900 | 0.838 | 0.046 | 200 |
| M0152 | full | exp13 | 2048bit-ECFP4 | RF | 0.891 | 0.873 | 0.924 | 0.840 | 0.856 | 0.769 | 0.882 | 0.834 | 0.022 | 50 |
| M0153 | full | exp14 | 2048bit-ECFP4 | RF | 0.873 | 0.859 | 0.918 | 0.802 | 0.829 | 0.730 | 0.860 | 0.860 | 0.031 | 400 |
| M0154 | full | exp15 | 2048bit-ECFP4 | RF | 0.891 | 0.922 | 0.959 | 0.783 | 0.847 | 0.770 | 0.871 | 0.836 | 0.011 | 50 |
| M0155 | full | exp16 | 2048bit-ECFP4 | RF | 0.884 | 0.920 | 0.959 | 0.764 | 0.835 | 0.755 | 0.861 | 0.847 | 0.012 | 50 |
| M0156 | full | exp17 | 2048bit-ECFP4 | RF | 0.899 | 0.924 | 0.959 | 0.802 | 0.859 | 0.785 | 0.881 | 0.832 | 0.027 | 500 |
| M0157 | full | exp18 | 2048bit-ECFP4 | RF | 0.888 | 0.903 | 0.947 | 0.792 | 0.844 | 0.761 | 0.869 | 0.845 | 0.001 | 50 |
| M0158 | full | exp19 | 2048bit-ECFP4 | RF | 0.909 | 0.918 | 0.953 | 0.840 | 0.877 | 0.807 | 0.896 | 0.837 | 0.040 | 800 |
| M0159 | full | exp01 | 2048bit-ECFP4 | DNN2 | 0.884 | 0.902 | 0.947 | 0.783 | 0.838 | 0.753 | 0.865 | 0.847 | 0.009 | 300 80 0.2 |
| M0160 | full | exp02 | 2048bit-ECFP4 | DNN2 | 0.888 | 0.887 | 0.935 | 0.811 | 0.847 | 0.761 | 0.873 | 0.850 | 0.003 | 600 80 0.3 |
| M0161 | full | exp03 | 2048bit-ECFP4 | DNN2 | 0.902 | 0.891 | 0.935 | 0.849 | 0.870 | 0.792 | 0.892 | 0.846 | 0.024 | 600 140 0.5 |
| M0162 | full | exp01 | 2048bit-ECFP4 | DNN3 | 0.880 | 0.892 | 0.941 | 0.783 | 0.834 | 0.745 | 0.862 | 0.828 | 0.006 | 100 60 0.4 |
| M0163 | full | exp02 | 2048bit-ECFP4 | DNN3 | 0.877 | 0.883 | 0.935 | 0.783 | 0.830 | 0.737 | 0.859 | 0.664 | 0.166 | 200 60 0.5 |
| M0164 | full | exp03 | 2048bit-ECFP4 | DNN3 | 0.920 | 0.896 | 0.935 | 0.896 | 0.896 | 0.832 | 0.915 | 0.837 | 0.059 | 100 80 0.5 |
| M0165 | full | exp01 | 1024bit-ECFP6 | KNN | 0.862 | 0.862 | 0.924 | 0.764 | 0.810 | 0.706 | 0.844 | 0.813 | 0.003 | 5 distance |
| M0166 | full | exp02 | 1024bit-ECFP6 | KNN | 0.844 | 0.832 | 0.906 | 0.745 | 0.786 | 0.667 | 0.826 | 0.815 | 0.028 | 7 distance |
| M0167 | full | exp03 | 1024bit-ECFP6 | KNN | 0.877 | 0.840 | 0.900 | 0.840 | 0.840 | 0.740 | 0.870 | 0.814 | 0.026 | 3 uniform |
| M0168 | full | exp04 | 1024bit-ECFP6 | KNN | 0.877 | 0.800 | 0.859 | 0.906 | 0.850 | 0.750 | 0.883 | 0.795 | 0.055 | 5 distance |
| M0169 | full | exp05 | 1024bit-ECFP6 | KNN | 0.833 | 0.742 | 0.812 | 0.868 | 0.800 | 0.665 | 0.840 | 0.814 | 0.014 | 3 distance |
| M0170 | full | exp06 | 1024bit-ECFP6 | KNN | 0.884 | 0.843 | 0.900 | 0.858 | 0.850 | 0.756 | 0.879 | 0.812 | 0.038 | 3 uniform |
| M0171 | full | exp07 | 1024bit-ECFP6 | KNN | 0.870 | 0.824 | 0.888 | 0.840 | 0.832 | 0.725 | 0.864 | 0.801 | 0.031 | 5 distance |
| M0172 | full | exp08 | 1024bit-ECFP6 | KNN | 0.873 | 0.838 | 0.900 | 0.830 | 0.834 | 0.732 | 0.865 | 0.807 | 0.027 | 5 distance |
| M0173 | full | exp09 | 1024bit-ECFP6 | KNN | 0.833 | 0.763 | 0.841 | 0.821 | 0.791 | 0.654 | 0.831 | 0.784 | 0.007 | 11 distance |
| M0174 | full | exp10 | 1024bit-ECFP6 | KNN | 0.859 | 0.825 | 0.894 | 0.802 | 0.813 | 0.700 | 0.848 | 0.788 | 0.025 | 5 distance |
| M0175 | full | exp11 | 1024bit-ECFP6 | KNN | 0.848 | 0.786 | 0.859 | 0.830 | 0.807 | 0.682 | 0.845 | 0.827 | 0.020 | 3 uniform |
| M0176 | full | exp12 | 1024bit-ECFP6 | KNN | 0.888 | 0.838 | 0.894 | 0.877 | 0.857 | 0.765 | 0.885 | 0.814 | 0.043 | 5 uniform |
| M0177 | full | exp13 | 1024bit-ECFP6 | KNN | 0.844 | 0.756 | 0.824 | 0.877 | 0.812 | 0.686 | 0.851 | 0.798 | 0.015 | 5 distance |
| M0178 | full | exp14 | 1024bit-ECFP6 | KNN | 0.841 | 0.750 | 0.818 | 0.877 | 0.809 | 0.680 | 0.847 | 0.800 | 0.009 | 5 uniform |
| M0179 | full | exp15 | 1024bit-ECFP6 | KNN | 0.844 | 0.779 | 0.853 | 0.830 | 0.804 | 0.676 | 0.841 | 0.806 | 0.002 | 1 uniform |
| M0180 | full | exp16 | 1024bit-ECFP6 | KNN | 0.841 | 0.792 | 0.871 | 0.792 | 0.792 | 0.663 | 0.832 | 0.811 | 0.019 | 3 distance |
| M0181 | full | exp17 | 1024bit-ECFP6 | KNN | 0.837 | 0.775 | 0.853 | 0.811 | 0.793 | 0.659 | 0.832 | 0.804 | 0.011 | 3 uniform |
| M0182 | full | exp18 | 1024bit-ECFP6 | KNN | 0.862 | 0.821 | 0.888 | 0.821 | 0.821 | 0.709 | 0.855 | 0.816 | 0.005 | 5 distance |
| M0183 | full | exp19 | 1024bit-ECFP6 | KNN | 0.888 | 0.832 | 0.888 | 0.887 | 0.858 | 0.767 | 0.887 | 0.796 | 0.063 | 5 distance |
| M0184 | full | exp01 | 1024bit-ECFP6 | SVM | 0.873 | 0.890 | 0.941 | 0.764 | 0.822 | 0.730 | 0.853 | 0.836 | 0.014 | 5 0.01 |
| M0185 | full | exp02 | 1024bit-ECFP6 | SVM | 0.888 | 0.895 | 0.941 | 0.802 | 0.846 | 0.761 | 0.871 | 0.832 | 0.014 | 5 0.005 |
| M0186 | full | exp03 | 1024bit-ECFP6 | SVM | 0.870 | 0.865 | 0.924 | 0.783 | 0.822 | 0.722 | 0.854 | 0.834 | 0.012 | 10 0.01 |
| M0187 | full | exp04 | 1024bit-ECFP6 | SVM | 0.884 | 0.863 | 0.918 | 0.830 | 0.846 | 0.754 | 0.874 | 0.818 | 0.028 | 50 0.01 |
| M0188 | full | exp05 | 1024bit-ECFP6 | SVM | 0.877 | 0.846 | 0.906 | 0.830 | 0.838 | 0.739 | 0.868 | 0.833 | 0.005 | 5 0.01 |
| M0189 | full | exp06 | 1024bit-ECFP6 | SVM | 0.870 | 0.880 | 0.935 | 0.764 | 0.818 | 0.722 | 0.850 | 0.836 | 0.018 | 50000.0 0.01 |
| M0190 | full | exp07 | 1024bit-ECFP6 | SVM | 0.877 | 0.860 | 0.918 | 0.811 | 0.835 | 0.738 | 0.865 | 0.846 | 0.011 | 5 0.01 |
| M0191 | full | exp08 | 1024bit-ECFP6 | SVM | 0.880 | 0.892 | 0.941 | 0.783 | 0.834 | 0.745 | 0.862 | 0.825 | 0.009 | 5 0.01 |
| M0192 | full | exp09 | 1024bit-ECFP6 | SVM | 0.884 | 0.856 | 0.912 | 0.840 | 0.848 | 0.754 | 0.876 | 0.837 | 0.011 | 5 0.01 |
| M0193 | full | exp10 | 1024bit-ECFP6 | SVM | 0.870 | 0.857 | 0.918 | 0.792 | 0.824 | 0.722 | 0.855 | 0.838 | 0.014 | 5 0.005 |
| M0194 | full | exp11 | 1024bit-ECFP6 | SVM | 0.870 | 0.880 | 0.935 | 0.764 | 0.818 | 0.722 | 0.850 | 0.849 | 0.031 | 5 0.005 |
| M0195 | full | exp12 | 1024bit-ECFP6 | SVM | 0.888 | 0.879 | 0.929 | 0.821 | 0.849 | 0.761 | 0.875 | 0.828 | 0.021 | 5 0.005 |
| M0196 | full | exp13 | 1024bit-ECFP6 | SVM | 0.873 | 0.814 | 0.876 | 0.868 | 0.840 | 0.736 | 0.872 | 0.830 | 0.010 | 5 0.01 |
| M0197 | full | exp14 | 1024bit-ECFP6 | SVM | 0.851 | 0.788 | 0.859 | 0.840 | 0.813 | 0.691 | 0.849 | 0.836 | 0.023 | 5 0.01 |
| M0198 | full | exp15 | 1024bit-ECFP6 | SVM | 0.884 | 0.911 | 0.953 | 0.774 | 0.837 | 0.754 | 0.863 | 0.832 | 0.005 | 10 0.01 |
| M0199 | full | exp16 | 1024bit-ECFP6 | SVM | 0.862 | 0.878 | 0.935 | 0.745 | 0.806 | 0.706 | 0.840 | 0.842 | 0.036 | 1000.0 0.05 |
| M0200 | full | exp17 | 1024bit-ECFP6 | SVM | 0.877 | 0.867 | 0.924 | 0.802 | 0.833 | 0.737 | 0.863 | 0.835 | 0.002 | 10 0.01 |
| M0201 | full | exp18 | 1024bit-ECFP6 | SVM | 0.891 | 0.880 | 0.929 | 0.830 | 0.854 | 0.769 | 0.879 | 0.828 | 0.026 | 100 0.05 |
| M0202 | full | exp19 | 1024bit-ECFP6 | SVM | 0.913 | 0.910 | 0.947 | 0.858 | 0.883 | 0.815 | 0.902 | 0.831 | 0.052 | 5 0.01 |
| M0203 | full | exp01 | 1024bit-ECFP6 | GBM | 0.851 | 0.874 | 0.935 | 0.717 | 0.788 | 0.683 | 0.826 | 0.819 | 0.031 | 200 0.8 |
| M0204 | full | exp02 | 1024bit-ECFP6 | GBM | 0.880 | 0.884 | 0.935 | 0.792 | 0.836 | 0.745 | 0.864 | 0.818 | 0.018 | 200 0.9 |
| M0205 | full | exp03 | 1024bit-ECFP6 | GBM | 0.873 | 0.851 | 0.912 | 0.811 | 0.831 | 0.730 | 0.862 | 0.828 | 0.003 | 400 0.7 |
| M0206 | full | exp04 | 1024bit-ECFP6 | GBM | 0.888 | 0.879 | 0.929 | 0.821 | 0.849 | 0.761 | 0.875 | 0.828 | 0.021 | 700 0.1 |
| M0207 | full | exp05 | 1024bit-ECFP6 | GBM | 0.877 | 0.867 | 0.924 | 0.802 | 0.833 | 0.737 | 0.863 | 0.829 | 0.004 | 800 0.2 |
| M0208 | full | exp06 | 1024bit-ECFP6 | GBM | 0.880 | 0.876 | 0.929 | 0.802 | 0.837 | 0.745 | 0.866 | 0.847 | 0.010 | 500 0.1 |
| M0209 | full | exp07 | 1024bit-ECFP6 | GBM | 0.873 | 0.890 | 0.941 | 0.764 | 0.822 | 0.730 | 0.853 | 0.832 | 0.010 | 50 0.4 |
| M0210 | full | exp08 | 1024bit-ECFP6 | GBM | 0.906 | 0.944 | 0.971 | 0.802 | 0.867 | 0.801 | 0.887 | 0.830 | 0.037 | 400 0.1 |
| M0211 | full | exp09 | 1024bit-ECFP6 | GBM | 0.870 | 0.850 | 0.912 | 0.802 | 0.825 | 0.722 | 0.857 | 0.825 | 0.000 | 200 0.4 |
| M0212 | full | exp10 | 1024bit-ECFP6 | GBM | 0.891 | 0.913 | 0.953 | 0.792 | 0.848 | 0.769 | 0.873 | 0.820 | 0.028 | 800 0.1 |
| M0213 | full | exp11 | 1024bit-ECFP6 | GBM | 0.884 | 0.930 | 0.965 | 0.755 | 0.833 | 0.756 | 0.860 | 0.844 | 0.011 | 100 0.3 |
| M0214 | full | exp12 | 1024bit-ECFP6 | GBM | 0.899 | 0.924 | 0.959 | 0.802 | 0.859 | 0.785 | 0.881 | 0.823 | 0.036 | 500 0.1 |
| M0215 | full | exp13 | 1024bit-ECFP6 | GBM | 0.870 | 0.830 | 0.894 | 0.830 | 0.830 | 0.724 | 0.862 | 0.822 | 0.008 | 100 0.3 |
| M0216 | full | exp14 | 1024bit-ECFP6 | GBM | 0.859 | 0.853 | 0.918 | 0.764 | 0.806 | 0.698 | 0.841 | 0.834 | 0.027 | 200 0.4 |
| M0217 | full | exp15 | 1024bit-ECFP6 | GBM | 0.884 | 0.885 | 0.935 | 0.802 | 0.842 | 0.753 | 0.869 | 0.824 | 0.018 | 700 0.3 |
| M0218 | full | exp16 | 1024bit-ECFP6 | GBM | 0.866 | 0.879 | 0.935 | 0.755 | 0.812 | 0.714 | 0.845 | 0.818 | 0.006 | 200 0.5 |
| M0219 | full | exp17 | 1024bit-ECFP6 | GBM | 0.880 | 0.892 | 0.941 | 0.783 | 0.834 | 0.745 | 0.862 | 0.830 | 0.004 | 200 0.2 |
| M0220 | full | exp18 | 1024bit-ECFP6 | GBM | 0.870 | 0.865 | 0.924 | 0.783 | 0.822 | 0.722 | 0.854 | 0.831 | 0.009 | 400 0.1 |
| M0221 | full | exp19 | 1024bit-ECFP6 | GBM | 0.888 | 0.887 | 0.935 | 0.811 | 0.847 | 0.761 | 0.873 | 0.816 | 0.031 | 900 0.1 |
| M0222 | full | exp01 | 1024bit-ECFP6 | RF | 0.866 | 0.937 | 0.971 | 0.698 | 0.800 | 0.720 | 0.835 | 0.828 | 0.028 | 600 |
| M0223 | full | exp02 | 1024bit-ECFP6 | RF | 0.866 | 0.926 | 0.965 | 0.708 | 0.802 | 0.718 | 0.837 | 0.828 | 0.026 | 600 |
| M0224 | full | exp03 | 1024bit-ECFP6 | RF | 0.888 | 0.941 | 0.971 | 0.755 | 0.838 | 0.764 | 0.863 | 0.827 | 0.011 | 50 |
| M0225 | full | exp04 | 1024bit-ECFP6 | RF | 0.877 | 0.883 | 0.935 | 0.783 | 0.830 | 0.737 | 0.859 | 0.828 | 0.002 | 50 |
| M0226 | full | exp05 | 1024bit-ECFP6 | RF | 0.884 | 0.902 | 0.947 | 0.783 | 0.838 | 0.753 | 0.865 | 0.844 | 0.006 | 500 |
| M0227 | full | exp06 | 1024bit-ECFP6 | RF | 0.906 | 0.976 | 0.988 | 0.774 | 0.863 | 0.805 | 0.881 | 0.837 | 0.026 | 800 |
| M0228 | full | exp07 | 1024bit-ECFP6 | RF | 0.891 | 0.952 | 0.976 | 0.755 | 0.842 | 0.773 | 0.865 | 0.836 | 0.006 | 600 |
| M0229 | full | exp08 | 1024bit-ECFP6 | RF | 0.888 | 0.963 | 0.982 | 0.736 | 0.834 | 0.767 | 0.859 | 0.837 | 0.003 | 50 |
| M0230 | full | exp09 | 1024bit-ECFP6 | RF | 0.895 | 0.923 | 0.959 | 0.792 | 0.853 | 0.777 | 0.875 | 0.820 | 0.033 | 700 |
| M0231 | full | exp10 | 1024bit-ECFP6 | RF | 0.891 | 0.952 | 0.976 | 0.755 | 0.842 | 0.773 | 0.865 | 0.823 | 0.019 | 600 |
| M0232 | full | exp11 | 1024bit-ECFP6 | RF | 0.873 | 0.918 | 0.959 | 0.736 | 0.817 | 0.732 | 0.847 | 0.840 | 0.023 | 900 |
| M0233 | full | exp12 | 1024bit-ECFP6 | RF | 0.917 | 0.956 | 0.976 | 0.821 | 0.883 | 0.825 | 0.898 | 0.821 | 0.062 | 50 |
| M0234 | full | exp13 | 1024bit-ECFP6 | RF | 0.909 | 0.926 | 0.959 | 0.830 | 0.876 | 0.808 | 0.894 | 0.819 | 0.057 | 100 |
| M0235 | full | exp14 | 1024bit-ECFP6 | RF | 0.862 | 0.862 | 0.924 | 0.764 | 0.810 | 0.706 | 0.844 | 0.848 | 0.038 | 600 |
| M0236 | full | exp15 | 1024bit-ECFP6 | RF | 0.888 | 0.941 | 0.971 | 0.755 | 0.838 | 0.764 | 0.863 | 0.831 | 0.007 | 800 |
| M0237 | full | exp16 | 1024bit-ECFP6 | RF | 0.877 | 0.909 | 0.953 | 0.755 | 0.825 | 0.739 | 0.854 | 0.833 | 0.008 | 1000 |
| M0238 | full | exp17 | 1024bit-ECFP6 | RF | 0.888 | 0.921 | 0.959 | 0.774 | 0.841 | 0.762 | 0.867 | 0.822 | 0.019 | 300 |
| M0239 | full | exp18 | 1024bit-ECFP6 | RF | 0.891 | 0.913 | 0.953 | 0.792 | 0.848 | 0.769 | 0.873 | 0.824 | 0.024 | 1000 |
| M0240 | full | exp19 | 1024bit-ECFP6 | RF | 0.909 | 0.926 | 0.959 | 0.830 | 0.876 | 0.808 | 0.894 | 0.827 | 0.049 | 900 |
| M0241 | full | exp01 | 1024bit-ECFP6 | DNN2 | 0.866 | 0.906 | 0.953 | 0.726 | 0.806 | 0.716 | 0.839 | 0.837 | 0.031 | 200 140 0.2 |
| M0242 | full | exp02 | 1024bit-ECFP6 | DNN2 | 0.873 | 0.882 | 0.935 | 0.774 | 0.824 | 0.729 | 0.855 | 0.844 | 0.020 | 100 160 0.2 |
| M0243 | full | exp03 | 1024bit-ECFP6 | DNN2 | 0.880 | 0.910 | 0.953 | 0.764 | 0.831 | 0.746 | 0.859 | 0.841 | 0.010 | 400 80 0.1 |
| M0244 | full | exp01 | 1024bit-ECFP6 | DNN3 | 0.877 | 0.909 | 0.953 | 0.755 | 0.825 | 0.739 | 0.854 | 0.838 | 0.013 | 400 100 0.5 |
| M0245 | full | exp02 | 1024bit-ECFP6 | DNN3 | 0.880 | 0.892 | 0.941 | 0.783 | 0.834 | 0.745 | 0.862 | 0.839 | 0.005 | 200 120 0.3 |
| M0246 | full | exp03 | 1024bit-ECFP6 | DNN3 | 0.870 | 0.889 | 0.941 | 0.755 | 0.816 | 0.722 | 0.848 | 0.843 | 0.027 | 500 80 0.5 |
| M0247 | full | exp01 | 2048bit-ECFP6 | KNN | 0.873 | 0.890 | 0.941 | 0.764 | 0.822 | 0.730 | 0.853 | 0.818 | 0.004 | 5 distance |
| M0248 | full | exp02 | 2048bit-ECFP6 | KNN | 0.859 | 0.845 | 0.912 | 0.774 | 0.808 | 0.698 | 0.843 | 0.820 | 0.012 | 5 uniform |
| M0249 | full | exp03 | 2048bit-ECFP6 | KNN | 0.877 | 0.860 | 0.918 | 0.811 | 0.835 | 0.738 | 0.865 | 0.812 | 0.023 | 3 uniform |
| M0250 | full | exp04 | 2048bit-ECFP6 | KNN | 0.837 | 0.740 | 0.806 | 0.887 | 0.807 | 0.676 | 0.847 | 0.793 | 0.014 | 3 distance |
| M0251 | full | exp05 | 2048bit-ECFP6 | KNN | 0.851 | 0.773 | 0.841 | 0.868 | 0.818 | 0.696 | 0.855 | 0.811 | 0.007 | 3 uniform |
| M0252 | full | exp06 | 2048bit-ECFP6 | KNN | 0.888 | 0.850 | 0.906 | 0.858 | 0.854 | 0.763 | 0.882 | 0.809 | 0.045 | 3 uniform |
| M0253 | full | exp07 | 2048bit-ECFP6 | KNN | 0.866 | 0.829 | 0.894 | 0.821 | 0.825 | 0.716 | 0.857 | 0.815 | 0.010 | 5 distance |
| M0254 | full | exp08 | 2048bit-ECFP6 | KNN | 0.899 | 0.861 | 0.912 | 0.877 | 0.869 | 0.786 | 0.895 | 0.811 | 0.058 | 3 distance |
| M0255 | full | exp09 | 2048bit-ECFP6 | KNN | 0.830 | 0.752 | 0.829 | 0.830 | 0.789 | 0.649 | 0.829 | 0.801 | 0.012 | 5 distance |
| M0256 | full | exp10 | 2048bit-ECFP6 | KNN | 0.880 | 0.848 | 0.906 | 0.840 | 0.844 | 0.747 | 0.873 | 0.797 | 0.047 | 3 distance |
| M0257 | full | exp11 | 2048bit-ECFP6 | KNN | 0.855 | 0.800 | 0.871 | 0.830 | 0.815 | 0.696 | 0.851 | 0.831 | 0.016 | 3 uniform |
| M0258 | full | exp12 | 2048bit-ECFP6 | KNN | 0.906 | 0.885 | 0.929 | 0.868 | 0.876 | 0.800 | 0.899 | 0.818 | 0.058 | 5 uniform |
| M0259 | full | exp13 | 2048bit-ECFP6 | KNN | 0.833 | 0.738 | 0.806 | 0.877 | 0.802 | 0.667 | 0.842 | 0.798 | 0.004 | 3 distance |
| M0260 | full | exp14 | 2048bit-ECFP6 | KNN | 0.848 | 0.767 | 0.835 | 0.868 | 0.814 | 0.690 | 0.851 | 0.815 | 0.001 | 5 uniform |
| M0261 | full | exp15 | 2048bit-ECFP6 | KNN | 0.862 | 0.815 | 0.882 | 0.830 | 0.822 | 0.710 | 0.856 | 0.810 | 0.012 | 5 uniform |
| M0262 | full | exp16 | 2048bit-ECFP6 | KNN | 0.833 | 0.759 | 0.835 | 0.830 | 0.793 | 0.656 | 0.833 | 0.817 | 0.024 | 1 uniform |
| M0263 | full | exp17 | 2048bit-ECFP6 | KNN | 0.862 | 0.798 | 0.865 | 0.858 | 0.827 | 0.714 | 0.861 | 0.805 | 0.022 | 1 uniform |
| M0264 | full | exp18 | 2048bit-ECFP6 | KNN | 0.870 | 0.830 | 0.894 | 0.830 | 0.830 | 0.724 | 0.862 | 0.812 | 0.018 | 3 distance |
| M0265 | full | exp19 | 2048bit-ECFP6 | KNN | 0.870 | 0.807 | 0.871 | 0.868 | 0.836 | 0.730 | 0.869 | 0.797 | 0.039 | 3 distance |
| M0266 | full | exp01 | 2048bit-ECFP6 | SVM | 0.888 | 0.921 | 0.959 | 0.774 | 0.841 | 0.762 | 0.867 | 0.850 | 0.009 | 5 0.005 |
| M0267 | full | exp02 | 2048bit-ECFP6 | SVM | 0.895 | 0.905 | 0.947 | 0.811 | 0.856 | 0.776 | 0.879 | 0.856 | 0.000 | 5 0.005 |
| M0268 | full | exp03 | 2048bit-ECFP6 | SVM | 0.888 | 0.903 | 0.947 | 0.792 | 0.844 | 0.761 | 0.869 | 0.842 | 0.002 | 100 0.01 |
| M0269 | full | exp04 | 2048bit-ECFP6 | SVM | 0.909 | 0.901 | 0.941 | 0.858 | 0.879 | 0.807 | 0.899 | 0.833 | 0.046 | 5 0.005 |
| M0270 | full | exp05 | 2048bit-ECFP6 | SVM | 0.880 | 0.861 | 0.918 | 0.821 | 0.841 | 0.746 | 0.869 | 0.843 | 0.002 | 1000.0 0.01 |
| M0271 | full | exp06 | 2048bit-ECFP6 | SVM | 0.888 | 0.887 | 0.935 | 0.811 | 0.847 | 0.761 | 0.873 | 0.853 | 0.006 | 5 0.01 |
| M0272 | full | exp07 | 2048bit-ECFP6 | SVM | 0.899 | 0.906 | 0.947 | 0.821 | 0.861 | 0.784 | 0.884 | 0.851 | 0.010 | 5 0.005 |
| M0273 | full | exp08 | 2048bit-ECFP6 | SVM | 0.891 | 0.913 | 0.953 | 0.792 | 0.848 | 0.769 | 0.873 | 0.840 | 0.008 | 5 0.005 |
| M0274 | full | exp09 | 2048bit-ECFP6 | SVM | 0.888 | 0.864 | 0.918 | 0.840 | 0.852 | 0.762 | 0.879 | 0.850 | 0.002 | 5 0.01 |
| M0275 | full | exp10 | 2048bit-ECFP6 | SVM | 0.884 | 0.878 | 0.929 | 0.811 | 0.843 | 0.753 | 0.870 | 0.848 | 0.005 | 500 5e-05 |
| M0276 | full | exp11 | 2048bit-ECFP6 | SVM | 0.888 | 0.895 | 0.941 | 0.802 | 0.846 | 0.761 | 0.871 | 0.856 | 0.010 | 5 0.005 |
| M0277 | full | exp12 | 2048bit-ECFP6 | SVM | 0.888 | 0.879 | 0.929 | 0.821 | 0.849 | 0.761 | 0.875 | 0.826 | 0.023 | 100 0.01 |
| M0278 | full | exp13 | 2048bit-ECFP6 | SVM | 0.902 | 0.862 | 0.912 | 0.887 | 0.874 | 0.795 | 0.899 | 0.836 | 0.038 | 5 0.005 |
| M0279 | full | exp14 | 2048bit-ECFP6 | SVM | 0.866 | 0.829 | 0.894 | 0.821 | 0.825 | 0.716 | 0.857 | 0.849 | 0.024 | 5 0.01 |
| M0280 | full | exp15 | 2048bit-ECFP6 | SVM | 0.895 | 0.914 | 0.953 | 0.802 | 0.854 | 0.777 | 0.877 | 0.842 | 0.012 | 5000.0 0.01 |
| M0281 | full | exp16 | 2048bit-ECFP6 | SVM | 0.873 | 0.899 | 0.947 | 0.755 | 0.821 | 0.730 | 0.851 | 0.836 | 0.015 | 10 0.05 |
| M0282 | full | exp17 | 2048bit-ECFP6 | SVM | 0.899 | 0.898 | 0.941 | 0.830 | 0.863 | 0.784 | 0.885 | 0.838 | 0.025 | 50 0.0005 |
| M0283 | full | exp18 | 2048bit-ECFP6 | SVM | 0.888 | 0.857 | 0.912 | 0.849 | 0.853 | 0.762 | 0.881 | 0.845 | 0.008 | 50 0.0005 |
| M0284 | full | exp19 | 2048bit-ECFP6 | SVM | 0.931 | 0.931 | 0.959 | 0.887 | 0.908 | 0.854 | 0.923 | 0.832 | 0.076 | 5 0.01 |
| M0285 | full | exp01 | 2048bit-ECFP6 | GBM | 0.870 | 0.889 | 0.941 | 0.755 | 0.816 | 0.722 | 0.848 | 0.826 | 0.010 | 200 0.2 |
| M0286 | full | exp02 | 2048bit-ECFP6 | GBM | 0.895 | 0.943 | 0.971 | 0.774 | 0.850 | 0.779 | 0.873 | 0.822 | 0.028 | 300 0.2 |
| M0287 | full | exp03 | 2048bit-ECFP6 | GBM | 0.884 | 0.885 | 0.935 | 0.802 | 0.842 | 0.753 | 0.869 | 0.826 | 0.016 | 500 0.1 |
| M0288 | full | exp04 | 2048bit-ECFP6 | GBM | 0.873 | 0.845 | 0.906 | 0.821 | 0.833 | 0.731 | 0.863 | 0.815 | 0.018 | 500 0.1 |
| M0289 | full | exp05 | 2048bit-ECFP6 | GBM | 0.877 | 0.867 | 0.924 | 0.802 | 0.833 | 0.737 | 0.863 | 0.827 | 0.006 | 50 0.5 |
| M0290 | full | exp06 | 2048bit-ECFP6 | GBM | 0.895 | 0.914 | 0.953 | 0.802 | 0.854 | 0.777 | 0.877 | 0.834 | 0.020 | 400 0.3 |
| M0291 | full | exp07 | 2048bit-ECFP6 | GBM | 0.877 | 0.883 | 0.935 | 0.783 | 0.830 | 0.737 | 0.859 | 0.817 | 0.013 | 100 0.2 |
| M0292 | full | exp08 | 2048bit-ECFP6 | GBM | 0.880 | 0.920 | 0.959 | 0.755 | 0.829 | 0.747 | 0.857 | 0.832 | 0.003 | 100 0.2 |
| M0293 | full | exp09 | 2048bit-ECFP6 | GBM | 0.895 | 0.914 | 0.953 | 0.802 | 0.854 | 0.777 | 0.877 | 0.825 | 0.029 | 50 0.4 |
| M0294 | full | exp10 | 2048bit-ECFP6 | GBM | 0.873 | 0.874 | 0.929 | 0.783 | 0.826 | 0.729 | 0.856 | 0.813 | 0.013 | 700 0.1 |
| M0295 | full | exp11 | 2048bit-ECFP6 | GBM | 0.870 | 0.880 | 0.935 | 0.764 | 0.818 | 0.722 | 0.850 | 0.829 | 0.011 | 900 0.2 |
| M0296 | full | exp12 | 2048bit-ECFP6 | GBM | 0.902 | 0.934 | 0.965 | 0.802 | 0.863 | 0.793 | 0.883 | 0.818 | 0.045 | 200 0.1 |
| M0297 | full | exp13 | 2048bit-ECFP6 | GBM | 0.855 | 0.800 | 0.871 | 0.830 | 0.815 | 0.696 | 0.851 | 0.814 | 0.001 | 500 0.2 |
| M0298 | full | exp14 | 2048bit-ECFP6 | GBM | 0.873 | 0.874 | 0.929 | 0.783 | 0.826 | 0.729 | 0.856 | 0.827 | 0.001 | 300 0.6 |
| M0299 | full | exp15 | 2048bit-ECFP6 | GBM | 0.877 | 0.900 | 0.947 | 0.764 | 0.827 | 0.738 | 0.855 | 0.816 | 0.011 | 500 0.5 |
| M0300 | full | exp16 | 2048bit-ECFP6 | GBM | 0.848 | 0.840 | 0.912 | 0.745 | 0.790 | 0.674 | 0.829 | 0.811 | 0.021 | 400 0.4 |
| M0301 | full | exp17 | 2048bit-ECFP6 | GBM | 0.880 | 0.869 | 0.924 | 0.811 | 0.839 | 0.745 | 0.868 | 0.805 | 0.034 | 200 0.3 |
| M0302 | full | exp18 | 2048bit-ECFP6 | GBM | 0.888 | 0.903 | 0.947 | 0.792 | 0.844 | 0.761 | 0.869 | 0.822 | 0.022 | 400 0.6 |
| M0303 | full | exp19 | 2048bit-ECFP6 | GBM | 0.902 | 0.925 | 0.959 | 0.811 | 0.864 | 0.792 | 0.885 | 0.815 | 0.049 | 100 0.1 |
| M0304 | full | exp01 | 2048bit-ECFP6 | RF | 0.855 | 0.893 | 0.947 | 0.708 | 0.789 | 0.692 | 0.827 | 0.839 | 0.050 | 100 |
| M0305 | full | exp02 | 2048bit-ECFP6 | RF | 0.877 | 0.929 | 0.965 | 0.736 | 0.821 | 0.741 | 0.851 | 0.830 | 0.009 | 600 |
| M0306 | full | exp03 | 2048bit-ECFP6 | RF | 0.884 | 0.930 | 0.965 | 0.755 | 0.833 | 0.756 | 0.860 | 0.831 | 0.002 | 50 |
| M0307 | full | exp04 | 2048bit-ECFP6 | RF | 0.895 | 0.905 | 0.947 | 0.811 | 0.856 | 0.776 | 0.879 | 0.830 | 0.026 | 400 |
| M0308 | full | exp05 | 2048bit-ECFP6 | RF | 0.880 | 0.884 | 0.935 | 0.792 | 0.836 | 0.745 | 0.864 | 0.842 | 0.006 | 900 |
| M0309 | full | exp06 | 2048bit-ECFP6 | RF | 0.902 | 0.965 | 0.982 | 0.774 | 0.859 | 0.796 | 0.878 | 0.830 | 0.029 | 700 |
| M0310 | full | exp07 | 2048bit-ECFP6 | RF | 0.891 | 0.942 | 0.971 | 0.764 | 0.844 | 0.772 | 0.867 | 0.840 | 0.004 | 50 |
| M0311 | full | exp08 | 2048bit-ECFP6 | RF | 0.895 | 0.943 | 0.971 | 0.774 | 0.850 | 0.779 | 0.873 | 0.838 | 0.012 | 500 |
| M0312 | full | exp09 | 2048bit-ECFP6 | RF | 0.891 | 0.922 | 0.959 | 0.783 | 0.847 | 0.770 | 0.871 | 0.825 | 0.022 | 50 |
| M0313 | full | exp10 | 2048bit-ECFP6 | RF | 0.895 | 0.953 | 0.976 | 0.764 | 0.848 | 0.780 | 0.870 | 0.829 | 0.019 | 200 |
| M0314 | full | exp11 | 2048bit-ECFP6 | RF | 0.880 | 0.929 | 0.965 | 0.745 | 0.827 | 0.748 | 0.855 | 0.839 | 0.012 | 200 |
| M0315 | full | exp12 | 2048bit-ECFP6 | RF | 0.906 | 0.955 | 0.976 | 0.792 | 0.866 | 0.802 | 0.884 | 0.827 | 0.039 | 400 |
| M0316 | full | exp13 | 2048bit-ECFP6 | RF | 0.909 | 0.918 | 0.953 | 0.840 | 0.877 | 0.807 | 0.896 | 0.818 | 0.059 | 50 |
| M0317 | full | exp14 | 2048bit-ECFP6 | RF | 0.862 | 0.862 | 0.924 | 0.764 | 0.810 | 0.706 | 0.844 | 0.850 | 0.040 | 400 |
| M0318 | full | exp15 | 2048bit-ECFP6 | RF | 0.891 | 0.942 | 0.971 | 0.764 | 0.844 | 0.772 | 0.867 | 0.836 | 0.008 | 100 |
| M0319 | full | exp16 | 2048bit-ECFP6 | RF | 0.884 | 0.920 | 0.959 | 0.764 | 0.835 | 0.755 | 0.861 | 0.836 | 0.001 | 800 |
| M0320 | full | exp17 | 2048bit-ECFP6 | RF | 0.891 | 0.932 | 0.965 | 0.774 | 0.845 | 0.771 | 0.869 | 0.825 | 0.020 | 800 |
| M0321 | full | exp18 | 2048bit-ECFP6 | RF | 0.895 | 0.923 | 0.959 | 0.792 | 0.853 | 0.777 | 0.875 | 0.828 | 0.025 | 300 |
| M0322 | full | exp19 | 2048bit-ECFP6 | RF | 0.906 | 0.908 | 0.947 | 0.840 | 0.873 | 0.800 | 0.893 | 0.842 | 0.031 | 100 |
| M0323 | full | exp01 | 2048bit-ECFP6 | DNN2 | 0.888 | 0.921 | 0.959 | 0.774 | 0.841 | 0.762 | 0.867 | 0.846 | 0.005 | 500 100 0.5 |
| M0324 | full | exp02 | 2048bit-ECFP6 | DNN2 | 0.873 | 0.866 | 0.924 | 0.792 | 0.828 | 0.729 | 0.858 | 0.850 | 0.022 | 100 60 0.4 |
| M0325 | full | exp03 | 2048bit-ECFP6 | DNN2 | 0.877 | 0.883 | 0.935 | 0.783 | 0.830 | 0.737 | 0.859 | 0.844 | 0.014 | 400 100 0.5 |
| M0326 | full | exp01 | 2048bit-ECFP6 | DNN3 | 0.877 | 0.900 | 0.947 | 0.764 | 0.827 | 0.738 | 0.855 | 0.812 | 0.015 | 100 80 0.5 |
| M0327 | full | exp02 | 2048bit-ECFP6 | DNN3 | 0.870 | 0.961 | 0.982 | 0.689 | 0.802 | 0.731 | 0.835 | 0.767 | 0.035 | 100 60 0.5 |
| M0328 | full | exp03 | 2048bit-ECFP6 | DNN3 | 0.870 | 0.927 | 0.965 | 0.717 | 0.809 | 0.726 | 0.841 | 0.799 | 0.010 | 100 60 0.5 |
| M0329 | 512 | exp01 | 1024bit-ECFP4 | KNN | 0.870 | 0.872 | 0.929 | 0.774 | 0.820 | 0.721 | 0.852 | 0.847 | 0.027 | 3 distance |
| M0330 | 512 | exp02 | 1024bit-ECFP4 | KNN | 0.877 | 0.846 | 0.906 | 0.830 | 0.838 | 0.739 | 0.868 | 0.841 | 0.003 | 3 uniform |
| M0331 | 512 | exp03 | 1024bit-ECFP4 | KNN | 0.870 | 0.850 | 0.912 | 0.802 | 0.825 | 0.722 | 0.857 | 0.832 | 0.007 | 5 distance |
| M0332 | 512 | exp04 | 1024bit-ECFP4 | KNN | 0.873 | 0.789 | 0.847 | 0.915 | 0.847 | 0.746 | 0.881 | 0.827 | 0.020 | 1 uniform |
| M0333 | 512 | exp05 | 1024bit-ECFP4 | KNN | 0.833 | 0.759 | 0.835 | 0.830 | 0.793 | 0.656 | 0.833 | 0.842 | 0.049 | 3 distance |
| M0334 | 512 | exp06 | 1024bit-ECFP4 | KNN | 0.888 | 0.857 | 0.912 | 0.849 | 0.853 | 0.762 | 0.881 | 0.836 | 0.017 | 3 uniform |
| M0335 | 512 | exp07 | 1024bit-ECFP4 | KNN | 0.902 | 0.899 | 0.941 | 0.840 | 0.868 | 0.792 | 0.890 | 0.831 | 0.037 | 3 distance |
| M0336 | 512 | exp08 | 1024bit-ECFP4 | KNN | 0.895 | 0.860 | 0.912 | 0.868 | 0.864 | 0.778 | 0.890 | 0.822 | 0.042 | 3 uniform |
| M0337 | 512 | exp09 | 1024bit-ECFP4 | KNN | 0.880 | 0.841 | 0.900 | 0.849 | 0.845 | 0.748 | 0.875 | 0.838 | 0.007 | 3 distance |
| M0338 | 512 | exp10 | 1024bit-ECFP4 | KNN | 0.880 | 0.884 | 0.935 | 0.792 | 0.836 | 0.745 | 0.864 | 0.822 | 0.014 | 7 distance |
| M0339 | 512 | exp11 | 1024bit-ECFP4 | KNN | 0.870 | 0.837 | 0.900 | 0.821 | 0.829 | 0.723 | 0.861 | 0.850 | 0.021 | 3 distance |
| M0340 | 512 | exp12 | 1024bit-ECFP4 | KNN | 0.895 | 0.897 | 0.941 | 0.821 | 0.857 | 0.776 | 0.881 | 0.838 | 0.019 | 5 uniform |
| M0341 | 512 | exp13 | 1024bit-ECFP4 | KNN | 0.841 | 0.731 | 0.788 | 0.925 | 0.817 | 0.694 | 0.857 | 0.822 | 0.005 | 1 uniform |
| M0342 | 512 | exp14 | 1024bit-ECFP4 | KNN | 0.873 | 0.789 | 0.847 | 0.915 | 0.847 | 0.746 | 0.881 | 0.838 | 0.009 | 3 distance |
| M0343 | 512 | exp15 | 1024bit-ECFP4 | KNN | 0.862 | 0.809 | 0.876 | 0.840 | 0.824 | 0.711 | 0.858 | 0.830 | 0.006 | 3 uniform |
| M0344 | 512 | exp16 | 1024bit-ECFP4 | KNN | 0.855 | 0.800 | 0.871 | 0.830 | 0.815 | 0.696 | 0.851 | 0.834 | 0.019 | 1 uniform |
| M0345 | 512 | exp17 | 1024bit-ECFP4 | KNN | 0.859 | 0.807 | 0.876 | 0.830 | 0.819 | 0.703 | 0.853 | 0.827 | 0.008 | 3 distance |
| M0346 | 512 | exp18 | 1024bit-ECFP4 | KNN | 0.866 | 0.848 | 0.912 | 0.792 | 0.820 | 0.714 | 0.852 | 0.836 | 0.016 | 5 distance |
| M0347 | 512 | exp19 | 1024bit-ECFP4 | KNN | 0.888 | 0.871 | 0.924 | 0.830 | 0.850 | 0.761 | 0.877 | 0.809 | 0.041 | 7 distance |
| M0348 | 512 | exp01 | 1024bit-ECFP4 | SVM | 0.873 | 0.890 | 0.941 | 0.764 | 0.822 | 0.730 | 0.853 | 0.853 | 0.031 | 1000.0 0.05 |
| M0349 | 512 | exp02 | 1024bit-ECFP4 | SVM | 0.902 | 0.891 | 0.935 | 0.849 | 0.870 | 0.792 | 0.892 | 0.848 | 0.022 | 10000.0 0.05 |
| M0350 | 512 | exp03 | 1024bit-ECFP4 | SVM | 0.906 | 0.917 | 0.953 | 0.830 | 0.871 | 0.800 | 0.891 | 0.845 | 0.026 | 1000.0 0.05 |
| M0351 | 512 | exp04 | 1024bit-ECFP4 | SVM | 0.899 | 0.861 | 0.912 | 0.877 | 0.869 | 0.786 | 0.895 | 0.843 | 0.026 | 1000.0 0.05 |
| M0352 | 512 | exp05 | 1024bit-ECFP4 | SVM | 0.862 | 0.833 | 0.900 | 0.802 | 0.817 | 0.707 | 0.851 | 0.861 | 0.044 | 50 0.05 |
| M0353 | 512 | exp06 | 1024bit-ECFP4 | SVM | 0.884 | 0.878 | 0.929 | 0.811 | 0.843 | 0.753 | 0.870 | 0.858 | 0.015 | 5 0.05 |
| M0354 | 512 | exp07 | 1024bit-ECFP4 | SVM | 0.909 | 0.926 | 0.959 | 0.830 | 0.876 | 0.808 | 0.894 | 0.855 | 0.021 | 10 0.05 |
| M0355 | 512 | exp08 | 1024bit-ECFP4 | SVM | 0.895 | 0.881 | 0.929 | 0.840 | 0.860 | 0.777 | 0.885 | 0.843 | 0.017 | 10000.0 0.05 |
| M0356 | 512 | exp09 | 1024bit-ECFP4 | SVM | 0.899 | 0.882 | 0.929 | 0.849 | 0.865 | 0.784 | 0.889 | 0.844 | 0.021 | 10000.0 0.05 |
| M0357 | 512 | exp10 | 1024bit-ECFP4 | SVM | 0.917 | 0.937 | 0.965 | 0.840 | 0.886 | 0.823 | 0.902 | 0.844 | 0.042 | 50000.0 0.05 |
| M0358 | 512 | exp11 | 1024bit-ECFP4 | SVM | 0.877 | 0.883 | 0.935 | 0.783 | 0.830 | 0.737 | 0.859 | 0.856 | 0.026 | 1 0.05 |
| M0359 | 512 | exp12 | 1024bit-ECFP4 | SVM | 0.917 | 0.903 | 0.941 | 0.877 | 0.890 | 0.823 | 0.909 | 0.852 | 0.038 | 500 0.05 |
| M0360 | 512 | exp13 | 1024bit-ECFP4 | SVM | 0.899 | 0.868 | 0.918 | 0.868 | 0.868 | 0.786 | 0.893 | 0.842 | 0.026 | 1000.0 0.05 |
| M0361 | 512 | exp14 | 1024bit-ECFP4 | SVM | 0.866 | 0.822 | 0.888 | 0.830 | 0.826 | 0.717 | 0.859 | 0.852 | 0.026 | 1 0.05 |
| M0362 | 512 | exp15 | 1024bit-ECFP4 | SVM | 0.913 | 0.946 | 0.971 | 0.821 | 0.879 | 0.816 | 0.896 | 0.846 | 0.033 | 50 0.05 |
| M0363 | 512 | exp16 | 1024bit-ECFP4 | SVM | 0.873 | 0.882 | 0.935 | 0.774 | 0.824 | 0.729 | 0.855 | 0.855 | 0.031 | 50000.0 0.05 |
| M0364 | 512 | exp17 | 1024bit-ECFP4 | SVM | 0.884 | 0.863 | 0.918 | 0.830 | 0.846 | 0.754 | 0.874 | 0.839 | 0.007 | 10 0.05 |
| M0365 | 512 | exp18 | 1024bit-ECFP4 | SVM | 0.884 | 0.863 | 0.918 | 0.830 | 0.846 | 0.754 | 0.874 | 0.846 | 0.000 | 5 0.05 |
| M0366 | 512 | exp19 | 1024bit-ECFP4 | SVM | 0.917 | 0.919 | 0.953 | 0.858 | 0.888 | 0.823 | 0.905 | 0.844 | 0.044 | 1 0.05 |
| M0367 | 512 | exp01 | 1024bit-ECFP4 | GBM | 0.888 | 0.941 | 0.971 | 0.755 | 0.838 | 0.764 | 0.863 | 0.846 | 0.008 | 300 0.4 |
| M0368 | 512 | exp02 | 1024bit-ECFP4 | GBM | 0.895 | 0.933 | 0.965 | 0.783 | 0.851 | 0.778 | 0.874 | 0.831 | 0.020 | 500 0.2 |
| M0369 | 512 | exp03 | 1024bit-ECFP4 | GBM | 0.873 | 0.882 | 0.935 | 0.774 | 0.824 | 0.729 | 0.855 | 0.840 | 0.016 | 100 0.7 |
| M0370 | 512 | exp04 | 1024bit-ECFP4 | GBM | 0.902 | 0.883 | 0.929 | 0.858 | 0.871 | 0.792 | 0.893 | 0.833 | 0.038 | 100 0.2 |
| M0371 | 512 | exp05 | 1024bit-ECFP4 | GBM | 0.855 | 0.830 | 0.900 | 0.783 | 0.806 | 0.691 | 0.842 | 0.843 | 0.037 | 600 0.1 |
| M0372 | 512 | exp06 | 1024bit-ECFP4 | GBM | 0.891 | 0.913 | 0.953 | 0.792 | 0.848 | 0.769 | 0.873 | 0.842 | 0.006 | 700 0.1 |
| M0373 | 512 | exp07 | 1024bit-ECFP4 | GBM | 0.884 | 0.911 | 0.953 | 0.774 | 0.837 | 0.754 | 0.863 | 0.835 | 0.002 | 200 0.4 |
| M0374 | 512 | exp08 | 1024bit-ECFP4 | GBM | 0.877 | 0.867 | 0.924 | 0.802 | 0.833 | 0.737 | 0.863 | 0.839 | 0.006 | 400 0.2 |
| M0375 | 512 | exp09 | 1024bit-ECFP4 | GBM | 0.870 | 0.830 | 0.894 | 0.830 | 0.830 | 0.724 | 0.862 | 0.828 | 0.002 | 400 0.7 |
| M0376 | 512 | exp10 | 1024bit-ECFP4 | GBM | 0.873 | 0.899 | 0.947 | 0.755 | 0.821 | 0.730 | 0.851 | 0.837 | 0.016 | 50 0.4 |
| M0377 | 512 | exp11 | 1024bit-ECFP4 | GBM | 0.866 | 0.871 | 0.929 | 0.764 | 0.814 | 0.714 | 0.847 | 0.847 | 0.033 | 600 0.4 |
| M0378 | 512 | exp12 | 1024bit-ECFP4 | GBM | 0.909 | 0.926 | 0.959 | 0.830 | 0.876 | 0.808 | 0.894 | 0.815 | 0.061 | 400 0.2 |
| M0379 | 512 | exp13 | 1024bit-ECFP4 | GBM | 0.873 | 0.845 | 0.906 | 0.821 | 0.833 | 0.731 | 0.863 | 0.847 | 0.014 | 200 0.2 |
| M0380 | 512 | exp14 | 1024bit-ECFP4 | GBM | 0.859 | 0.838 | 0.906 | 0.783 | 0.810 | 0.699 | 0.845 | 0.841 | 0.031 | 400 0.1 |
| M0381 | 512 | exp15 | 1024bit-ECFP4 | GBM | 0.888 | 0.903 | 0.947 | 0.792 | 0.844 | 0.761 | 0.869 | 0.831 | 0.013 | 200 0.3 |
| M0382 | 512 | exp16 | 1024bit-ECFP4 | GBM | 0.855 | 0.875 | 0.935 | 0.726 | 0.794 | 0.691 | 0.831 | 0.825 | 0.030 | 400 0.5 |
| M0383 | 512 | exp17 | 1024bit-ECFP4 | GBM | 0.884 | 0.940 | 0.971 | 0.745 | 0.832 | 0.757 | 0.858 | 0.813 | 0.019 | 50 0.2 |
| M0384 | 512 | exp18 | 1024bit-ECFP4 | GBM | 0.873 | 0.851 | 0.912 | 0.811 | 0.831 | 0.730 | 0.862 | 0.842 | 0.011 | 300 0.8 |
| M0385 | 512 | exp19 | 1024bit-ECFP4 | GBM | 0.880 | 0.876 | 0.929 | 0.802 | 0.837 | 0.745 | 0.866 | 0.818 | 0.019 | 300 0.1 |
| M0386 | 512 | exp01 | 1024bit-ECFP4 | RF | 0.873 | 0.928 | 0.965 | 0.726 | 0.815 | 0.733 | 0.845 | 0.840 | 0.025 | 200 |
| M0387 | 512 | exp02 | 1024bit-ECFP4 | RF | 0.877 | 0.919 | 0.959 | 0.745 | 0.823 | 0.739 | 0.852 | 0.840 | 0.017 | 500 |
| M0388 | 512 | exp03 | 1024bit-ECFP4 | RF | 0.891 | 0.922 | 0.959 | 0.783 | 0.847 | 0.770 | 0.871 | 0.837 | 0.010 | 600 |
| M0389 | 512 | exp04 | 1024bit-ECFP4 | RF | 0.902 | 0.899 | 0.941 | 0.840 | 0.868 | 0.792 | 0.890 | 0.840 | 0.028 | 200 |
| M0390 | 512 | exp05 | 1024bit-ECFP4 | RF | 0.877 | 0.875 | 0.929 | 0.792 | 0.832 | 0.737 | 0.861 | 0.847 | 0.015 | 400 |
| M0391 | 512 | exp06 | 1024bit-ECFP4 | RF | 0.906 | 0.965 | 0.982 | 0.783 | 0.865 | 0.804 | 0.883 | 0.850 | 0.015 | 100 |
| M0392 | 512 | exp07 | 1024bit-ECFP4 | RF | 0.895 | 0.933 | 0.965 | 0.783 | 0.851 | 0.778 | 0.874 | 0.851 | 0.000 | 100 |
| M0393 | 512 | exp08 | 1024bit-ECFP4 | RF | 0.895 | 0.933 | 0.965 | 0.783 | 0.851 | 0.778 | 0.874 | 0.839 | 0.012 | 400 |
| M0394 | 512 | exp09 | 1024bit-ECFP4 | RF | 0.902 | 0.916 | 0.953 | 0.821 | 0.866 | 0.792 | 0.887 | 0.833 | 0.033 | 700 |
| M0395 | 512 | exp10 | 1024bit-ECFP4 | RF | 0.891 | 0.932 | 0.965 | 0.774 | 0.845 | 0.771 | 0.869 | 0.835 | 0.010 | 100 |
| M0396 | 512 | exp11 | 1024bit-ECFP4 | RF | 0.884 | 0.885 | 0.935 | 0.802 | 0.842 | 0.753 | 0.869 | 0.850 | 0.008 | 50 |
| M0397 | 512 | exp12 | 1024bit-ECFP4 | RF | 0.917 | 0.946 | 0.971 | 0.830 | 0.884 | 0.824 | 0.900 | 0.829 | 0.055 | 100 |
| M0398 | 512 | exp13 | 1024bit-ECFP4 | RF | 0.913 | 0.910 | 0.947 | 0.858 | 0.883 | 0.815 | 0.902 | 0.832 | 0.051 | 50 |
| M0399 | 512 | exp14 | 1024bit-ECFP4 | RF | 0.866 | 0.856 | 0.918 | 0.783 | 0.818 | 0.714 | 0.851 | 0.857 | 0.039 | 300 |
| M0400 | 512 | exp15 | 1024bit-ECFP4 | RF | 0.902 | 0.954 | 0.976 | 0.783 | 0.860 | 0.795 | 0.879 | 0.834 | 0.026 | 900 |
| M0401 | 512 | exp16 | 1024bit-ECFP4 | RF | 0.873 | 0.908 | 0.953 | 0.745 | 0.819 | 0.731 | 0.849 | 0.843 | 0.024 | 200 |
| M0402 | 512 | exp17 | 1024bit-ECFP4 | RF | 0.884 | 0.911 | 0.953 | 0.774 | 0.837 | 0.754 | 0.863 | 0.839 | 0.002 | 400 |
| M0403 | 512 | exp18 | 1024bit-ECFP4 | RF | 0.888 | 0.912 | 0.953 | 0.783 | 0.843 | 0.761 | 0.868 | 0.842 | 0.001 | 200 |
| M0404 | 512 | exp19 | 1024bit-ECFP4 | RF | 0.913 | 0.927 | 0.959 | 0.840 | 0.881 | 0.815 | 0.899 | 0.838 | 0.043 | 600 |
| M0405 | 512 | exp01 | 1024bit-ECFP4 | DNN2 | 0.895 | 0.923 | 0.959 | 0.792 | 0.853 | 0.777 | 0.875 | 0.839 | 0.014 | 100 80 0.3 |
| M0406 | 512 | exp02 | 1024bit-ECFP4 | DNN2 | 0.906 | 0.908 | 0.947 | 0.840 | 0.873 | 0.800 | 0.893 | 0.842 | 0.031 | 600 80 0.2 |
| M0407 | 512 | exp03 | 1024bit-ECFP4 | DNN2 | 0.891 | 0.880 | 0.929 | 0.830 | 0.854 | 0.769 | 0.879 | 0.846 | 0.008 | 100 140 0.5 |
| M0408 | 512 | exp01 | 1024bit-ECFP4 | DNN3 | 0.895 | 0.905 | 0.947 | 0.811 | 0.856 | 0.776 | 0.879 | 0.851 | 0.005 | 500 120 0.4 |
| M0409 | 512 | exp02 | 1024bit-ECFP4 | DNN3 | 0.899 | 0.906 | 0.947 | 0.821 | 0.861 | 0.784 | 0.884 | 0.843 | 0.018 | 100 120 0.5 |
| M0410 | 512 | exp03 | 1024bit-ECFP4 | DNN3 | 0.880 | 0.861 | 0.918 | 0.821 | 0.841 | 0.746 | 0.869 | 0.850 | 0.009 | 500 60 0.3 |
| M0411 | 512 | exp01 | 2048bit-ECFP4 | KNN | 0.877 | 0.900 | 0.947 | 0.764 | 0.827 | 0.738 | 0.855 | 0.847 | 0.020 | 3 distance |
| M0412 | 512 | exp02 | 2048bit-ECFP4 | KNN | 0.870 | 0.850 | 0.912 | 0.802 | 0.825 | 0.722 | 0.857 | 0.847 | 0.022 | 3 uniform |
| M0413 | 512 | exp03 | 2048bit-ECFP4 | KNN | 0.880 | 0.854 | 0.912 | 0.830 | 0.842 | 0.746 | 0.871 | 0.842 | 0.000 | 3 distance |
| M0414 | 512 | exp04 | 2048bit-ECFP4 | KNN | 0.866 | 0.790 | 0.853 | 0.887 | 0.836 | 0.726 | 0.870 | 0.832 | 0.004 | 1 uniform |
| M0415 | 512 | exp05 | 2048bit-ECFP4 | KNN | 0.851 | 0.793 | 0.865 | 0.830 | 0.811 | 0.689 | 0.847 | 0.842 | 0.030 | 1 uniform |
| M0416 | 512 | exp06 | 2048bit-ECFP4 | KNN | 0.870 | 0.850 | 0.912 | 0.802 | 0.825 | 0.722 | 0.857 | 0.840 | 0.015 | 3 uniform |
| M0417 | 512 | exp07 | 2048bit-ECFP4 | KNN | 0.895 | 0.881 | 0.929 | 0.840 | 0.860 | 0.777 | 0.885 | 0.832 | 0.028 | 3 distance |
| M0418 | 512 | exp08 | 2048bit-ECFP4 | KNN | 0.888 | 0.844 | 0.900 | 0.868 | 0.856 | 0.764 | 0.884 | 0.835 | 0.021 | 1 uniform |
| M0419 | 512 | exp09 | 2048bit-ECFP4 | KNN | 0.859 | 0.802 | 0.871 | 0.840 | 0.820 | 0.704 | 0.855 | 0.828 | 0.008 | 3 distance |
| M0420 | 512 | exp10 | 2048bit-ECFP4 | KNN | 0.891 | 0.896 | 0.941 | 0.811 | 0.851 | 0.768 | 0.876 | 0.823 | 0.028 | 1 uniform |
| M0421 | 512 | exp11 | 2048bit-ECFP4 | KNN | 0.873 | 0.859 | 0.918 | 0.802 | 0.829 | 0.730 | 0.860 | 0.853 | 0.024 | 3 distance |
| M0422 | 512 | exp12 | 2048bit-ECFP4 | KNN | 0.906 | 0.926 | 0.959 | 0.821 | 0.870 | 0.800 | 0.890 | 0.837 | 0.033 | 5 uniform |
| M0423 | 512 | exp13 | 2048bit-ECFP4 | KNN | 0.873 | 0.798 | 0.859 | 0.896 | 0.844 | 0.742 | 0.877 | 0.834 | 0.010 | 1 uniform |
| M0424 | 512 | exp14 | 2048bit-ECFP4 | KNN | 0.859 | 0.782 | 0.847 | 0.877 | 0.827 | 0.711 | 0.862 | 0.850 | 0.023 | 3 distance |
| M0425 | 512 | exp15 | 2048bit-ECFP4 | KNN | 0.851 | 0.828 | 0.900 | 0.774 | 0.800 | 0.683 | 0.837 | 0.833 | 0.033 | 1 uniform |
| M0426 | 512 | exp16 | 2048bit-ECFP4 | KNN | 0.848 | 0.827 | 0.900 | 0.764 | 0.794 | 0.675 | 0.832 | 0.838 | 0.044 | 3 uniform |
| M0427 | 512 | exp17 | 2048bit-ECFP4 | KNN | 0.877 | 0.840 | 0.900 | 0.840 | 0.840 | 0.740 | 0.870 | 0.833 | 0.007 | 1 uniform |
| M0428 | 512 | exp18 | 2048bit-ECFP4 | KNN | 0.877 | 0.860 | 0.918 | 0.811 | 0.835 | 0.738 | 0.865 | 0.835 | 0.000 | 3 uniform |
| M0429 | 512 | exp19 | 2048bit-ECFP4 | KNN | 0.899 | 0.890 | 0.935 | 0.840 | 0.864 | 0.784 | 0.887 | 0.833 | 0.031 | 3 uniform |
| M0430 | 512 | exp01 | 2048bit-ECFP4 | SVM | 0.862 | 0.878 | 0.935 | 0.745 | 0.806 | 0.706 | 0.840 | 0.867 | 0.061 | 1000.0 0.05 |
| M0431 | 512 | exp02 | 2048bit-ECFP4 | SVM | 0.888 | 0.895 | 0.941 | 0.802 | 0.846 | 0.761 | 0.871 | 0.856 | 0.010 | 1 0.05 |
| M0432 | 512 | exp03 | 2048bit-ECFP4 | SVM | 0.884 | 0.894 | 0.941 | 0.792 | 0.840 | 0.753 | 0.867 | 0.853 | 0.013 | 1 0.05 |
| M0433 | 512 | exp04 | 2048bit-ECFP4 | SVM | 0.899 | 0.861 | 0.912 | 0.877 | 0.869 | 0.786 | 0.895 | 0.855 | 0.014 | 5 0.05 |
| M0434 | 512 | exp05 | 2048bit-ECFP4 | SVM | 0.866 | 0.842 | 0.906 | 0.802 | 0.821 | 0.715 | 0.854 | 0.867 | 0.046 | 5 0.1 |
| M0435 | 512 | exp06 | 2048bit-ECFP4 | SVM | 0.888 | 0.895 | 0.941 | 0.802 | 0.846 | 0.761 | 0.871 | 0.862 | 0.016 | 500 0.05 |
| M0436 | 512 | exp07 | 2048bit-ECFP4 | SVM | 0.899 | 0.915 | 0.953 | 0.811 | 0.860 | 0.784 | 0.882 | 0.863 | 0.003 | 1 0.1 |
| M0437 | 512 | exp08 | 2048bit-ECFP4 | SVM | 0.895 | 0.881 | 0.929 | 0.840 | 0.860 | 0.777 | 0.885 | 0.851 | 0.009 | 10 0.1 |
| M0438 | 512 | exp09 | 2048bit-ECFP4 | SVM | 0.902 | 0.883 | 0.929 | 0.858 | 0.871 | 0.792 | 0.893 | 0.854 | 0.017 | 5000.0 0.05 |
| M0439 | 512 | exp10 | 2048bit-ECFP4 | SVM | 0.913 | 0.936 | 0.965 | 0.830 | 0.880 | 0.816 | 0.897 | 0.854 | 0.026 | 5 0.1 |
| M0440 | 512 | exp11 | 2048bit-ECFP4 | SVM | 0.870 | 0.865 | 0.924 | 0.783 | 0.822 | 0.722 | 0.854 | 0.860 | 0.038 | 10 0.01 |
| M0441 | 512 | exp12 | 2048bit-ECFP4 | SVM | 0.920 | 0.920 | 0.953 | 0.868 | 0.893 | 0.831 | 0.910 | 0.847 | 0.046 | 5000.0 0.05 |
| M0442 | 512 | exp13 | 2048bit-ECFP4 | SVM | 0.895 | 0.853 | 0.906 | 0.877 | 0.865 | 0.779 | 0.891 | 0.851 | 0.014 | 50 0.05 |
| M0443 | 512 | exp14 | 2048bit-ECFP4 | SVM | 0.862 | 0.827 | 0.894 | 0.811 | 0.819 | 0.708 | 0.853 | 0.868 | 0.049 | 50000.0 0.05 |
| M0444 | 512 | exp15 | 2048bit-ECFP4 | SVM | 0.902 | 0.934 | 0.965 | 0.802 | 0.863 | 0.793 | 0.883 | 0.850 | 0.013 | 10 0.05 |
| M0445 | 512 | exp16 | 2048bit-ECFP4 | SVM | 0.891 | 0.904 | 0.947 | 0.802 | 0.850 | 0.769 | 0.875 | 0.862 | 0.012 | 1000.0 0.1 |
| M0446 | 512 | exp17 | 2048bit-ECFP4 | SVM | 0.899 | 0.890 | 0.935 | 0.840 | 0.864 | 0.784 | 0.887 | 0.853 | 0.011 | 10 0.1 |
| M0447 | 512 | exp18 | 2048bit-ECFP4 | SVM | 0.888 | 0.871 | 0.924 | 0.830 | 0.850 | 0.761 | 0.877 | 0.862 | 0.012 | 10 0.01 |
| M0448 | 512 | exp19 | 2048bit-ECFP4 | SVM | 0.909 | 0.901 | 0.941 | 0.858 | 0.879 | 0.807 | 0.899 | 0.848 | 0.031 | 10 0.005 |
| M0449 | 512 | exp01 | 2048bit-ECFP4 | GBM | 0.873 | 0.890 | 0.941 | 0.764 | 0.822 | 0.730 | 0.853 | 0.845 | 0.023 | 300 0.3 |
| M0450 | 512 | exp02 | 2048bit-ECFP4 | GBM | 0.888 | 0.912 | 0.953 | 0.783 | 0.843 | 0.761 | 0.868 | 0.844 | 0.001 | 100 0.3 |
| M0451 | 512 | exp03 | 2048bit-ECFP4 | GBM | 0.884 | 0.894 | 0.941 | 0.792 | 0.840 | 0.753 | 0.867 | 0.841 | 0.001 | 100 0.8 |
| M0452 | 512 | exp04 | 2048bit-ECFP4 | GBM | 0.891 | 0.880 | 0.929 | 0.830 | 0.854 | 0.769 | 0.879 | 0.823 | 0.031 | 200 0.3 |
| M0453 | 512 | exp05 | 2048bit-ECFP4 | GBM | 0.870 | 0.837 | 0.900 | 0.821 | 0.829 | 0.723 | 0.861 | 0.844 | 0.015 | 300 0.6 |
| M0454 | 512 | exp06 | 2048bit-ECFP4 | GBM | 0.891 | 0.932 | 0.965 | 0.774 | 0.845 | 0.771 | 0.869 | 0.851 | 0.006 | 300 0.4 |
| M0455 | 512 | exp07 | 2048bit-ECFP4 | GBM | 0.891 | 0.896 | 0.941 | 0.811 | 0.851 | 0.768 | 0.876 | 0.842 | 0.009 | 50 0.7 |
| M0456 | 512 | exp08 | 2048bit-ECFP4 | GBM | 0.880 | 0.892 | 0.941 | 0.783 | 0.834 | 0.745 | 0.862 | 0.846 | 0.012 | 50 0.3 |
| M0457 | 512 | exp09 | 2048bit-ECFP4 | GBM | 0.880 | 0.884 | 0.935 | 0.792 | 0.836 | 0.745 | 0.864 | 0.831 | 0.005 | 500 0.2 |
| M0458 | 512 | exp10 | 2048bit-ECFP4 | GBM | 0.906 | 0.926 | 0.959 | 0.821 | 0.870 | 0.800 | 0.890 | 0.838 | 0.032 | 50 0.5 |
| M0459 | 512 | exp11 | 2048bit-ECFP4 | GBM | 0.851 | 0.874 | 0.935 | 0.717 | 0.788 | 0.683 | 0.826 | 0.843 | 0.055 | 400 0.4 |
| M0460 | 512 | exp12 | 2048bit-ECFP4 | GBM | 0.906 | 0.917 | 0.953 | 0.830 | 0.871 | 0.800 | 0.891 | 0.824 | 0.047 | 400 0.5 |
| M0461 | 512 | exp13 | 2048bit-ECFP4 | GBM | 0.873 | 0.826 | 0.888 | 0.849 | 0.837 | 0.734 | 0.869 | 0.845 | 0.008 | 100 0.8 |
| M0462 | 512 | exp14 | 2048bit-ECFP4 | GBM | 0.844 | 0.806 | 0.882 | 0.783 | 0.794 | 0.669 | 0.833 | 0.845 | 0.051 | 200 0.9 |
| M0463 | 512 | exp15 | 2048bit-ECFP4 | GBM | 0.891 | 0.913 | 0.953 | 0.792 | 0.848 | 0.769 | 0.873 | 0.821 | 0.027 | 100 0.6 |
| M0464 | 512 | exp16 | 2048bit-ECFP4 | GBM | 0.873 | 0.908 | 0.953 | 0.745 | 0.819 | 0.731 | 0.849 | 0.834 | 0.015 | 50 0.5 |
| M0465 | 512 | exp17 | 2048bit-ECFP4 | GBM | 0.880 | 0.892 | 0.941 | 0.783 | 0.834 | 0.745 | 0.862 | 0.829 | 0.005 | 300 0.9 |
| M0466 | 512 | exp18 | 2048bit-ECFP4 | GBM | 0.877 | 0.853 | 0.912 | 0.821 | 0.837 | 0.738 | 0.867 | 0.854 | 0.017 | 100 0.5 |
| M0467 | 512 | exp19 | 2048bit-ECFP4 | GBM | 0.913 | 0.936 | 0.965 | 0.830 | 0.880 | 0.816 | 0.897 | 0.832 | 0.048 | 200 0.2 |
| M0468 | 512 | exp01 | 2048bit-ECFP4 | RF | 0.870 | 0.927 | 0.965 | 0.717 | 0.809 | 0.726 | 0.841 | 0.854 | 0.044 | 400 |
| M0469 | 512 | exp02 | 2048bit-ECFP4 | RF | 0.870 | 0.898 | 0.947 | 0.745 | 0.814 | 0.723 | 0.846 | 0.845 | 0.031 | 50 |
| M0470 | 512 | exp03 | 2048bit-ECFP4 | RF | 0.884 | 0.902 | 0.947 | 0.783 | 0.838 | 0.753 | 0.865 | 0.845 | 0.007 | 400 |
| M0471 | 512 | exp04 | 2048bit-ECFP4 | RF | 0.895 | 0.897 | 0.941 | 0.821 | 0.857 | 0.776 | 0.881 | 0.842 | 0.015 | 500 |
| M0472 | 512 | exp05 | 2048bit-ECFP4 | RF | 0.866 | 0.863 | 0.924 | 0.774 | 0.816 | 0.714 | 0.849 | 0.852 | 0.036 | 400 |
| M0473 | 512 | exp06 | 2048bit-ECFP4 | RF | 0.902 | 0.954 | 0.976 | 0.783 | 0.860 | 0.795 | 0.879 | 0.852 | 0.008 | 200 |
| M0474 | 512 | exp07 | 2048bit-ECFP4 | RF | 0.906 | 0.965 | 0.982 | 0.783 | 0.865 | 0.804 | 0.883 | 0.849 | 0.016 | 300 |
| M0475 | 512 | exp08 | 2048bit-ECFP4 | RF | 0.899 | 0.933 | 0.965 | 0.792 | 0.857 | 0.786 | 0.879 | 0.851 | 0.006 | 400 |
| M0476 | 512 | exp09 | 2048bit-ECFP4 | RF | 0.899 | 0.906 | 0.947 | 0.821 | 0.861 | 0.784 | 0.884 | 0.835 | 0.026 | 50 |
| M0477 | 512 | exp10 | 2048bit-ECFP4 | RF | 0.909 | 0.955 | 0.976 | 0.802 | 0.872 | 0.810 | 0.889 | 0.838 | 0.034 | 200 |
| M0478 | 512 | exp11 | 2048bit-ECFP4 | RF | 0.880 | 0.910 | 0.953 | 0.764 | 0.831 | 0.746 | 0.859 | 0.846 | 0.015 | 400 |
| M0479 | 512 | exp12 | 2048bit-ECFP4 | RF | 0.913 | 0.936 | 0.965 | 0.830 | 0.880 | 0.816 | 0.897 | 0.840 | 0.040 | 300 |
| M0480 | 512 | exp13 | 2048bit-ECFP4 | RF | 0.895 | 0.881 | 0.929 | 0.840 | 0.860 | 0.777 | 0.885 | 0.839 | 0.021 | 300 |
| M0481 | 512 | exp14 | 2048bit-ECFP4 | RF | 0.880 | 0.884 | 0.935 | 0.792 | 0.836 | 0.745 | 0.864 | 0.858 | 0.022 | 1000 |
| M0482 | 512 | exp15 | 2048bit-ECFP4 | RF | 0.899 | 0.943 | 0.971 | 0.783 | 0.856 | 0.787 | 0.877 | 0.832 | 0.024 | 50 |
| M0483 | 512 | exp16 | 2048bit-ECFP4 | RF | 0.877 | 0.909 | 0.953 | 0.755 | 0.825 | 0.739 | 0.854 | 0.853 | 0.028 | 100 |
| M0484 | 512 | exp17 | 2048bit-ECFP4 | RF | 0.888 | 0.921 | 0.959 | 0.774 | 0.841 | 0.762 | 0.867 | 0.843 | 0.002 | 800 |
| M0485 | 512 | exp18 | 2048bit-ECFP4 | RF | 0.891 | 0.922 | 0.959 | 0.783 | 0.847 | 0.770 | 0.871 | 0.850 | 0.003 | 300 |
| M0486 | 512 | exp19 | 2048bit-ECFP4 | RF | 0.902 | 0.925 | 0.959 | 0.811 | 0.864 | 0.792 | 0.885 | 0.843 | 0.021 | 50 |
| M0487 | 512 | exp01 | 2048bit-ECFP4 | DNN2 | 0.895 | 0.943 | 0.971 | 0.774 | 0.850 | 0.779 | 0.873 | 0.857 | 0.007 | 200 100 0.5 |
| M0488 | 512 | exp02 | 2048bit-ECFP4 | DNN2 | 0.895 | 0.897 | 0.941 | 0.821 | 0.857 | 0.776 | 0.881 | 0.857 | 0.000 | 200 140 0.1 |
| M0489 | 512 | exp03 | 2048bit-ECFP4 | DNN2 | 0.884 | 0.885 | 0.935 | 0.802 | 0.842 | 0.753 | 0.869 | 0.858 | 0.016 | 200 160 0.3 |
| M0490 | 512 | exp01 | 2048bit-ECFP4 | DNN3 | 0.880 | 0.901 | 0.947 | 0.774 | 0.832 | 0.746 | 0.861 | 0.853 | 0.021 | 400 140 0.5 |
| M0491 | 512 | exp02 | 2048bit-ECFP4 | DNN3 | 0.895 | 0.905 | 0.947 | 0.811 | 0.856 | 0.776 | 0.879 | 0.854 | 0.002 | 200 80 0.5 |
| M0492 | 512 | exp03 | 2048bit-ECFP4 | DNN3 | 0.888 | 0.887 | 0.935 | 0.811 | 0.847 | 0.761 | 0.873 | 0.855 | 0.008 | 500 80 0.4 |
| M0493 | 512 | exp01 | 1024bit-ECFP6 | KNN | 0.880 | 0.884 | 0.935 | 0.792 | 0.836 | 0.745 | 0.864 | 0.827 | 0.009 | 3 uniform |
| M0494 | 512 | exp02 | 1024bit-ECFP6 | KNN | 0.848 | 0.864 | 0.929 | 0.717 | 0.784 | 0.675 | 0.823 | 0.836 | 0.052 | 7 distance |
| M0495 | 512 | exp03 | 1024bit-ECFP6 | KNN | 0.877 | 0.853 | 0.912 | 0.821 | 0.837 | 0.738 | 0.867 | 0.827 | 0.010 | 3 uniform |
| M0496 | 512 | exp04 | 1024bit-ECFP6 | KNN | 0.862 | 0.788 | 0.853 | 0.877 | 0.830 | 0.718 | 0.865 | 0.819 | 0.011 | 1 uniform |
| M0497 | 512 | exp05 | 1024bit-ECFP6 | KNN | 0.873 | 0.820 | 0.882 | 0.858 | 0.839 | 0.735 | 0.870 | 0.834 | 0.005 | 1 uniform |
| M0498 | 512 | exp06 | 1024bit-ECFP6 | KNN | 0.880 | 0.869 | 0.924 | 0.811 | 0.839 | 0.745 | 0.868 | 0.823 | 0.016 | 5 uniform |
| M0499 | 512 | exp07 | 1024bit-ECFP6 | KNN | 0.870 | 0.872 | 0.929 | 0.774 | 0.820 | 0.721 | 0.852 | 0.816 | 0.004 | 7 distance |
| M0500 | 512 | exp08 | 1024bit-ECFP6 | KNN | 0.884 | 0.902 | 0.947 | 0.783 | 0.838 | 0.753 | 0.865 | 0.817 | 0.021 | 5 distance |
| M0501 | 512 | exp09 | 1024bit-ECFP6 | KNN | 0.862 | 0.809 | 0.876 | 0.840 | 0.824 | 0.711 | 0.858 | 0.828 | 0.004 | 5 uniform |
| M0502 | 512 | exp10 | 1024bit-ECFP6 | KNN | 0.884 | 0.863 | 0.918 | 0.830 | 0.846 | 0.754 | 0.874 | 0.807 | 0.039 | 3 distance |
| M0503 | 512 | exp11 | 1024bit-ECFP6 | KNN | 0.859 | 0.825 | 0.894 | 0.802 | 0.813 | 0.700 | 0.848 | 0.837 | 0.024 | 3 distance |
| M0504 | 512 | exp12 | 1024bit-ECFP6 | KNN | 0.906 | 0.900 | 0.941 | 0.849 | 0.874 | 0.800 | 0.895 | 0.835 | 0.039 | 5 uniform |
| M0505 | 512 | exp13 | 1024bit-ECFP6 | KNN | 0.833 | 0.731 | 0.794 | 0.896 | 0.805 | 0.673 | 0.845 | 0.825 | 0.020 | 1 uniform |
| M0506 | 512 | exp14 | 1024bit-ECFP6 | KNN | 0.844 | 0.769 | 0.841 | 0.849 | 0.807 | 0.679 | 0.845 | 0.834 | 0.027 | 3 uniform |
| M0507 | 512 | exp15 | 1024bit-ECFP6 | KNN | 0.859 | 0.813 | 0.882 | 0.821 | 0.817 | 0.702 | 0.851 | 0.833 | 0.016 | 1 uniform |
| M0508 | 512 | exp16 | 1024bit-ECFP6 | KNN | 0.855 | 0.824 | 0.894 | 0.792 | 0.808 | 0.692 | 0.843 | 0.828 | 0.020 | 3 uniform |
| M0509 | 512 | exp17 | 1024bit-ECFP6 | KNN | 0.855 | 0.800 | 0.871 | 0.830 | 0.815 | 0.696 | 0.851 | 0.831 | 0.016 | 1 uniform |
| M0510 | 512 | exp18 | 1024bit-ECFP6 | KNN | 0.859 | 0.838 | 0.906 | 0.783 | 0.810 | 0.699 | 0.845 | 0.829 | 0.019 | 5 uniform |
| M0511 | 512 | exp19 | 1024bit-ECFP6 | KNN | 0.884 | 0.878 | 0.929 | 0.811 | 0.843 | 0.753 | 0.870 | 0.809 | 0.034 | 5 uniform |
| M0512 | 512 | exp01 | 1024bit-ECFP6 | SVM | 0.884 | 0.911 | 0.953 | 0.774 | 0.837 | 0.754 | 0.863 | 0.852 | 0.015 | 5000.0 0.05 |
| M0513 | 512 | exp02 | 1024bit-ECFP6 | SVM | 0.884 | 0.885 | 0.935 | 0.802 | 0.842 | 0.753 | 0.869 | 0.843 | 0.001 | 5000.0 0.05 |
| M0514 | 512 | exp03 | 1024bit-ECFP6 | SVM | 0.880 | 0.876 | 0.929 | 0.802 | 0.837 | 0.745 | 0.866 | 0.837 | 0.000 | 100000.0 0.05 |
| M0515 | 512 | exp04 | 1024bit-ECFP6 | SVM | 0.906 | 0.885 | 0.929 | 0.868 | 0.876 | 0.800 | 0.899 | 0.850 | 0.026 | 50000.0 0.05 |
| M0516 | 512 | exp05 | 1024bit-ECFP6 | SVM | 0.859 | 0.825 | 0.894 | 0.802 | 0.813 | 0.700 | 0.848 | 0.854 | 0.041 | 10000.0 0.05 |
| M0517 | 512 | exp06 | 1024bit-ECFP6 | SVM | 0.884 | 0.894 | 0.941 | 0.792 | 0.840 | 0.753 | 0.867 | 0.847 | 0.007 | 5 0.05 |
| M0518 | 512 | exp07 | 1024bit-ECFP6 | SVM | 0.895 | 0.914 | 0.953 | 0.802 | 0.854 | 0.777 | 0.877 | 0.853 | 0.001 | 500 0.05 |
| M0519 | 512 | exp08 | 1024bit-ECFP6 | SVM | 0.895 | 0.933 | 0.965 | 0.783 | 0.851 | 0.778 | 0.874 | 0.845 | 0.006 | 1 0.05 |
| M0520 | 512 | exp09 | 1024bit-ECFP6 | SVM | 0.899 | 0.890 | 0.935 | 0.840 | 0.864 | 0.784 | 0.887 | 0.843 | 0.021 | 1000.0 0.05 |
| M0521 | 512 | exp10 | 1024bit-ECFP6 | SVM | 0.888 | 0.931 | 0.965 | 0.764 | 0.839 | 0.763 | 0.865 | 0.842 | 0.003 | 1 0.05 |
| M0522 | 512 | exp11 | 1024bit-ECFP6 | SVM | 0.884 | 0.894 | 0.941 | 0.792 | 0.840 | 0.753 | 0.867 | 0.841 | 0.001 | 1 0.05 |
| M0523 | 512 | exp12 | 1024bit-ECFP6 | SVM | 0.913 | 0.936 | 0.965 | 0.830 | 0.880 | 0.816 | 0.897 | 0.839 | 0.041 | 1 0.05 |
| M0524 | 512 | exp13 | 1024bit-ECFP6 | SVM | 0.906 | 0.877 | 0.924 | 0.877 | 0.877 | 0.801 | 0.901 | 0.836 | 0.041 | 1 0.05 |
| M0525 | 512 | exp14 | 1024bit-ECFP6 | SVM | 0.859 | 0.825 | 0.894 | 0.802 | 0.813 | 0.700 | 0.848 | 0.854 | 0.041 | 50 0.05 |
| M0526 | 512 | exp15 | 1024bit-ECFP6 | SVM | 0.888 | 0.912 | 0.953 | 0.783 | 0.843 | 0.761 | 0.868 | 0.846 | 0.003 | 10000.0 0.05 |
| M0527 | 512 | exp16 | 1024bit-ECFP6 | SVM | 0.891 | 0.913 | 0.953 | 0.792 | 0.848 | 0.769 | 0.873 | 0.855 | 0.007 | 50000.0 0.05 |
| M0528 | 512 | exp17 | 1024bit-ECFP6 | SVM | 0.888 | 0.864 | 0.918 | 0.840 | 0.852 | 0.762 | 0.879 | 0.840 | 0.012 | 500 0.05 |
| M0529 | 512 | exp18 | 1024bit-ECFP6 | SVM | 0.880 | 0.861 | 0.918 | 0.821 | 0.841 | 0.746 | 0.869 | 0.842 | 0.001 | 1 0.05 |
| M0530 | 512 | exp19 | 1024bit-ECFP6 | SVM | 0.920 | 0.938 | 0.965 | 0.849 | 0.891 | 0.831 | 0.907 | 0.837 | 0.054 | 10000.0 0.05 |
| M0531 | 512 | exp01 | 1024bit-ECFP6 | GBM | 0.859 | 0.904 | 0.953 | 0.708 | 0.794 | 0.701 | 0.831 | 0.823 | 0.029 | 500 0.4 |
| M0532 | 512 | exp02 | 1024bit-ECFP6 | GBM | 0.877 | 0.891 | 0.941 | 0.774 | 0.828 | 0.737 | 0.857 | 0.820 | 0.008 | 200 0.7 |
| M0533 | 512 | exp03 | 1024bit-ECFP6 | GBM | 0.877 | 0.860 | 0.918 | 0.811 | 0.835 | 0.738 | 0.865 | 0.823 | 0.012 | 300 0.9 |
| M0534 | 512 | exp04 | 1024bit-ECFP6 | GBM | 0.866 | 0.848 | 0.912 | 0.792 | 0.820 | 0.714 | 0.852 | 0.837 | 0.017 | 100 0.4 |
| M0535 | 512 | exp05 | 1024bit-ECFP6 | GBM | 0.877 | 0.867 | 0.924 | 0.802 | 0.833 | 0.737 | 0.863 | 0.836 | 0.003 | 50 0.5 |
| M0536 | 512 | exp06 | 1024bit-ECFP6 | GBM | 0.877 | 0.883 | 0.935 | 0.783 | 0.830 | 0.737 | 0.859 | 0.844 | 0.014 | 200 0.2 |
| M0537 | 512 | exp07 | 1024bit-ECFP6 | GBM | 0.873 | 0.899 | 0.947 | 0.755 | 0.821 | 0.730 | 0.851 | 0.839 | 0.018 | 50 0.4 |
| M0538 | 512 | exp08 | 1024bit-ECFP6 | GBM | 0.884 | 0.951 | 0.976 | 0.736 | 0.830 | 0.758 | 0.856 | 0.826 | 0.004 | 200 0.1 |
| M0539 | 512 | exp09 | 1024bit-ECFP6 | GBM | 0.899 | 0.906 | 0.947 | 0.821 | 0.861 | 0.784 | 0.884 | 0.823 | 0.038 | 400 0.2 |
| M0540 | 512 | exp10 | 1024bit-ECFP6 | GBM | 0.877 | 0.891 | 0.941 | 0.774 | 0.828 | 0.737 | 0.857 | 0.816 | 0.012 | 200 0.5 |
| M0541 | 512 | exp11 | 1024bit-ECFP6 | GBM | 0.851 | 0.874 | 0.935 | 0.717 | 0.788 | 0.683 | 0.826 | 0.844 | 0.055 | 400 0.1 |
| M0542 | 512 | exp12 | 1024bit-ECFP6 | GBM | 0.891 | 0.913 | 0.953 | 0.792 | 0.848 | 0.769 | 0.873 | 0.820 | 0.028 | 300 0.2 |
| M0543 | 512 | exp13 | 1024bit-ECFP6 | GBM | 0.877 | 0.875 | 0.929 | 0.792 | 0.832 | 0.737 | 0.861 | 0.816 | 0.016 | 100 0.2 |
| M0544 | 512 | exp14 | 1024bit-ECFP6 | GBM | 0.848 | 0.833 | 0.906 | 0.755 | 0.792 | 0.675 | 0.831 | 0.832 | 0.040 | 50 0.4 |
| M0545 | 512 | exp15 | 1024bit-ECFP6 | GBM | 0.877 | 0.950 | 0.976 | 0.717 | 0.817 | 0.743 | 0.847 | 0.820 | 0.003 | 50 0.3 |
| M0546 | 512 | exp16 | 1024bit-ECFP6 | GBM | 0.862 | 0.905 | 0.953 | 0.717 | 0.800 | 0.708 | 0.835 | 0.817 | 0.017 | 200 0.5 |
| M0547 | 512 | exp17 | 1024bit-ECFP6 | GBM | 0.895 | 0.943 | 0.971 | 0.774 | 0.850 | 0.779 | 0.873 | 0.826 | 0.024 | 100 0.2 |
| M0548 | 512 | exp18 | 1024bit-ECFP6 | GBM | 0.873 | 0.882 | 0.935 | 0.774 | 0.824 | 0.729 | 0.855 | 0.836 | 0.012 | 200 0.2 |
| M0549 | 512 | exp19 | 1024bit-ECFP6 | GBM | 0.895 | 0.905 | 0.947 | 0.811 | 0.856 | 0.776 | 0.879 | 0.814 | 0.042 | 200 0.1 |
| M0550 | 512 | exp01 | 1024bit-ECFP6 | RF | 0.870 | 0.938 | 0.971 | 0.708 | 0.806 | 0.727 | 0.839 | 0.837 | 0.031 | 700 |
| M0551 | 512 | exp02 | 1024bit-ECFP6 | RF | 0.877 | 0.939 | 0.971 | 0.726 | 0.819 | 0.742 | 0.849 | 0.833 | 0.014 | 700 |
| M0552 | 512 | exp03 | 1024bit-ECFP6 | RF | 0.877 | 0.929 | 0.965 | 0.736 | 0.821 | 0.741 | 0.851 | 0.825 | 0.004 | 50 |
| M0553 | 512 | exp04 | 1024bit-ECFP6 | RF | 0.880 | 0.892 | 0.941 | 0.783 | 0.834 | 0.745 | 0.862 | 0.826 | 0.008 | 100 |
| M0554 | 512 | exp05 | 1024bit-ECFP6 | RF | 0.877 | 0.883 | 0.935 | 0.783 | 0.830 | 0.737 | 0.859 | 0.852 | 0.022 | 300 |
| M0555 | 512 | exp06 | 1024bit-ECFP6 | RF | 0.899 | 0.964 | 0.982 | 0.764 | 0.853 | 0.789 | 0.873 | 0.842 | 0.011 | 300 |
| M0556 | 512 | exp07 | 1024bit-ECFP6 | RF | 0.884 | 0.940 | 0.971 | 0.745 | 0.832 | 0.757 | 0.858 | 0.838 | 0.006 | 200 |
| M0557 | 512 | exp08 | 1024bit-ECFP6 | RF | 0.895 | 0.964 | 0.982 | 0.755 | 0.847 | 0.782 | 0.869 | 0.827 | 0.020 | 1000 |
| M0558 | 512 | exp09 | 1024bit-ECFP6 | RF | 0.895 | 0.933 | 0.965 | 0.783 | 0.851 | 0.778 | 0.874 | 0.830 | 0.021 | 1000 |
| M0559 | 512 | exp10 | 1024bit-ECFP6 | RF | 0.891 | 0.952 | 0.976 | 0.755 | 0.842 | 0.773 | 0.865 | 0.823 | 0.019 | 200 |
| M0560 | 512 | exp11 | 1024bit-ECFP6 | RF | 0.866 | 0.906 | 0.953 | 0.726 | 0.806 | 0.716 | 0.839 | 0.842 | 0.036 | 900 |
| M0561 | 512 | exp12 | 1024bit-ECFP6 | RF | 0.906 | 0.955 | 0.976 | 0.792 | 0.866 | 0.802 | 0.884 | 0.823 | 0.043 | 400 |
| M0562 | 512 | exp13 | 1024bit-ECFP6 | RF | 0.899 | 0.906 | 0.947 | 0.821 | 0.861 | 0.784 | 0.884 | 0.821 | 0.040 | 50 |
| M0563 | 512 | exp14 | 1024bit-ECFP6 | RF | 0.866 | 0.871 | 0.929 | 0.764 | 0.814 | 0.714 | 0.847 | 0.850 | 0.036 | 100 |
| M0564 | 512 | exp15 | 1024bit-ECFP6 | RF | 0.891 | 0.942 | 0.971 | 0.764 | 0.844 | 0.772 | 0.867 | 0.836 | 0.008 | 900 |
| M0565 | 512 | exp16 | 1024bit-ECFP6 | RF | 0.866 | 0.906 | 0.953 | 0.726 | 0.806 | 0.716 | 0.839 | 0.840 | 0.034 | 1000 |
| M0566 | 512 | exp17 | 1024bit-ECFP6 | RF | 0.877 | 0.909 | 0.953 | 0.755 | 0.825 | 0.739 | 0.854 | 0.829 | 0.004 | 100 |
| M0567 | 512 | exp18 | 1024bit-ECFP6 | RF | 0.891 | 0.932 | 0.965 | 0.774 | 0.845 | 0.771 | 0.869 | 0.834 | 0.011 | 100 |
| M0568 | 512 | exp19 | 1024bit-ECFP6 | RF | 0.902 | 0.891 | 0.935 | 0.849 | 0.870 | 0.792 | 0.892 | 0.832 | 0.038 | 10 |
| M0569 | 512 | exp01 | 1024bit-ECFP6 | DNN2 | 0.841 | 0.869 | 0.935 | 0.689 | 0.768 | 0.660 | 0.812 | 0.829 | 0.061 | 100 140 0.2 |
| M0570 | 512 | exp02 | 1024bit-ECFP6 | DNN2 | 0.891 | 0.896 | 0.941 | 0.811 | 0.851 | 0.768 | 0.876 | 0.830 | 0.021 | 100 100 0.1 |
| M0571 | 512 | exp03 | 1024bit-ECFP6 | DNN2 | 0.873 | 0.859 | 0.918 | 0.802 | 0.829 | 0.730 | 0.860 | 0.827 | 0.002 | 400 120 0.4 |
| M0572 | 512 | exp01 | 1024bit-ECFP6 | DNN3 | 0.848 | 0.872 | 0.935 | 0.708 | 0.781 | 0.675 | 0.822 | 0.837 | 0.056 | 400 160 0.5 |
| M0573 | 512 | exp02 | 1024bit-ECFP6 | DNN3 | 0.891 | 0.888 | 0.935 | 0.821 | 0.853 | 0.768 | 0.878 | 0.833 | 0.020 | 600 80 0.2 |
| M0574 | 512 | exp03 | 1024bit-ECFP6 | DNN3 | 0.891 | 0.888 | 0.935 | 0.821 | 0.853 | 0.768 | 0.878 | 0.831 | 0.022 | 400 80 0.2 |
| M0575 | 512 | exp01 | 2048bit-ECFP6 | KNN | 0.859 | 0.838 | 0.906 | 0.783 | 0.810 | 0.699 | 0.845 | 0.855 | 0.045 | 1 uniform |
| M0576 | 512 | exp02 | 2048bit-ECFP6 | KNN | 0.873 | 0.882 | 0.935 | 0.774 | 0.824 | 0.729 | 0.855 | 0.850 | 0.026 | 3 uniform |
| M0577 | 512 | exp03 | 2048bit-ECFP6 | KNN | 0.848 | 0.814 | 0.888 | 0.783 | 0.798 | 0.676 | 0.836 | 0.841 | 0.042 | 1 uniform |
| M0578 | 512 | exp04 | 2048bit-ECFP6 | KNN | 0.880 | 0.854 | 0.912 | 0.830 | 0.842 | 0.746 | 0.871 | 0.830 | 0.012 | 3 distance |
| M0579 | 512 | exp05 | 2048bit-ECFP6 | KNN | 0.877 | 0.827 | 0.888 | 0.858 | 0.843 | 0.742 | 0.873 | 0.850 | 0.007 | 1 uniform |
| M0580 | 512 | exp06 | 2048bit-ECFP6 | KNN | 0.891 | 0.865 | 0.918 | 0.849 | 0.857 | 0.770 | 0.883 | 0.841 | 0.016 | 1 uniform |
| M0581 | 512 | exp07 | 2048bit-ECFP6 | KNN | 0.870 | 0.880 | 0.935 | 0.764 | 0.818 | 0.722 | 0.850 | 0.833 | 0.015 | 7 uniform |
| M0582 | 512 | exp08 | 2048bit-ECFP6 | KNN | 0.902 | 0.862 | 0.912 | 0.887 | 0.874 | 0.795 | 0.899 | 0.843 | 0.031 | 1 uniform |
| M0583 | 512 | exp09 | 2048bit-ECFP6 | KNN | 0.873 | 0.851 | 0.912 | 0.811 | 0.831 | 0.730 | 0.862 | 0.830 | 0.001 | 3 distance |
| M0584 | 512 | exp10 | 2048bit-ECFP6 | KNN | 0.891 | 0.896 | 0.941 | 0.811 | 0.851 | 0.768 | 0.876 | 0.829 | 0.022 | 1 uniform |
| M0585 | 512 | exp11 | 2048bit-ECFP6 | KNN | 0.873 | 0.859 | 0.918 | 0.802 | 0.829 | 0.730 | 0.860 | 0.854 | 0.025 | 3 distance |
| M0586 | 512 | exp12 | 2048bit-ECFP6 | KNN | 0.906 | 0.955 | 0.976 | 0.792 | 0.866 | 0.802 | 0.884 | 0.840 | 0.026 | 5 distance |
| M0587 | 512 | exp13 | 2048bit-ECFP6 | KNN | 0.862 | 0.779 | 0.841 | 0.896 | 0.833 | 0.722 | 0.869 | 0.840 | 0.007 | 1 uniform |
| M0588 | 512 | exp14 | 2048bit-ECFP6 | KNN | 0.862 | 0.809 | 0.876 | 0.840 | 0.824 | 0.711 | 0.858 | 0.843 | 0.019 | 3 distance |
| M0589 | 512 | exp15 | 2048bit-ECFP6 | KNN | 0.873 | 0.874 | 0.929 | 0.783 | 0.826 | 0.729 | 0.856 | 0.835 | 0.009 | 1 uniform |
| M0590 | 512 | exp16 | 2048bit-ECFP6 | KNN | 0.873 | 0.845 | 0.906 | 0.821 | 0.833 | 0.731 | 0.863 | 0.863 | 0.030 | 1 uniform |
| M0591 | 512 | exp17 | 2048bit-ECFP6 | KNN | 0.880 | 0.861 | 0.918 | 0.821 | 0.841 | 0.746 | 0.869 | 0.846 | 0.005 | 1 uniform |
| M0592 | 512 | exp18 | 2048bit-ECFP6 | KNN | 0.859 | 0.845 | 0.912 | 0.774 | 0.808 | 0.698 | 0.843 | 0.845 | 0.036 | 5 uniform |
| M0593 | 512 | exp19 | 2048bit-ECFP6 | KNN | 0.888 | 0.838 | 0.894 | 0.877 | 0.857 | 0.765 | 0.885 | 0.831 | 0.026 | 1 uniform |
| M0594 | 512 | exp01 | 2048bit-ECFP6 | SVM | 0.870 | 0.889 | 0.941 | 0.755 | 0.816 | 0.722 | 0.848 | 0.859 | 0.043 | 100 0.05 |
| M0595 | 512 | exp02 | 2048bit-ECFP6 | SVM | 0.884 | 0.885 | 0.935 | 0.802 | 0.842 | 0.753 | 0.869 | 0.858 | 0.016 | 50 0.05 |
| M0596 | 512 | exp03 | 2048bit-ECFP6 | SVM | 0.877 | 0.875 | 0.929 | 0.792 | 0.832 | 0.737 | 0.861 | 0.857 | 0.025 | 1 0.05 |
| M0597 | 512 | exp04 | 2048bit-ECFP6 | SVM | 0.899 | 0.898 | 0.941 | 0.830 | 0.863 | 0.784 | 0.885 | 0.855 | 0.008 | 10 0.1 |
| M0598 | 512 | exp05 | 2048bit-ECFP6 | SVM | 0.888 | 0.871 | 0.924 | 0.830 | 0.850 | 0.761 | 0.877 | 0.870 | 0.020 | 10000.0 0.05 |
| M0599 | 512 | exp06 | 2048bit-ECFP6 | SVM | 0.895 | 0.914 | 0.953 | 0.802 | 0.854 | 0.777 | 0.877 | 0.868 | 0.014 | 1000.0 0.05 |
| M0600 | 512 | exp07 | 2048bit-ECFP6 | SVM | 0.888 | 0.912 | 0.953 | 0.783 | 0.843 | 0.761 | 0.868 | 0.862 | 0.019 | 10 0.1 |
| M0601 | 512 | exp08 | 2048bit-ECFP6 | SVM | 0.891 | 0.904 | 0.947 | 0.802 | 0.850 | 0.769 | 0.875 | 0.865 | 0.015 | 10000.0 0.05 |
| M0602 | 512 | exp09 | 2048bit-ECFP6 | SVM | 0.899 | 0.906 | 0.947 | 0.821 | 0.861 | 0.784 | 0.884 | 0.844 | 0.017 | 50000.0 0.05 |
| M0603 | 512 | exp10 | 2048bit-ECFP6 | SVM | 0.906 | 0.944 | 0.971 | 0.802 | 0.867 | 0.801 | 0.887 | 0.858 | 0.009 | 50 0.05 |
| M0604 | 512 | exp11 | 2048bit-ECFP6 | SVM | 0.862 | 0.854 | 0.918 | 0.774 | 0.812 | 0.706 | 0.846 | 0.868 | 0.056 | 5 0.05 |
| M0605 | 512 | exp12 | 2048bit-ECFP6 | SVM | 0.920 | 0.947 | 0.971 | 0.840 | 0.890 | 0.832 | 0.905 | 0.852 | 0.038 | 500 0.05 |
| M0606 | 512 | exp13 | 2048bit-ECFP6 | SVM | 0.895 | 0.853 | 0.906 | 0.877 | 0.865 | 0.779 | 0.891 | 0.850 | 0.015 | 100000.0 0.05 |
| M0607 | 512 | exp14 | 2048bit-ECFP6 | SVM | 0.873 | 0.845 | 0.906 | 0.821 | 0.833 | 0.731 | 0.863 | 0.868 | 0.035 | 1000.0 0.1 |
| M0608 | 512 | exp15 | 2048bit-ECFP6 | SVM | 0.902 | 0.944 | 0.971 | 0.792 | 0.862 | 0.794 | 0.881 | 0.856 | 0.006 | 50 0.1 |
| M0609 | 512 | exp16 | 2048bit-ECFP6 | SVM | 0.888 | 0.903 | 0.947 | 0.792 | 0.844 | 0.761 | 0.869 | 0.869 | 0.025 | 50000.0 0.05 |
| M0610 | 512 | exp17 | 2048bit-ECFP6 | SVM | 0.895 | 0.889 | 0.935 | 0.830 | 0.859 | 0.776 | 0.883 | 0.856 | 0.003 | 50000.0 0.1 |
| M0611 | 512 | exp18 | 2048bit-ECFP6 | SVM | 0.899 | 0.924 | 0.959 | 0.802 | 0.859 | 0.785 | 0.881 | 0.860 | 0.001 | 50000.0 0.1 |
| M0612 | 512 | exp19 | 2048bit-ECFP6 | SVM | 0.906 | 0.908 | 0.947 | 0.840 | 0.873 | 0.800 | 0.893 | 0.844 | 0.029 | 1 0.05 |
| M0613 | 512 | exp01 | 2048bit-ECFP6 | GBM | 0.873 | 0.918 | 0.959 | 0.736 | 0.817 | 0.732 | 0.847 | 0.841 | 0.024 | 100 0.3 |
| M0614 | 512 | exp02 | 2048bit-ECFP6 | GBM | 0.870 | 0.898 | 0.947 | 0.745 | 0.814 | 0.723 | 0.846 | 0.838 | 0.024 | 600 0.3 |
| M0615 | 512 | exp03 | 2048bit-ECFP6 | GBM | 0.862 | 0.862 | 0.924 | 0.764 | 0.810 | 0.706 | 0.844 | 0.836 | 0.025 | 200 0.9 |
| M0616 | 512 | exp04 | 2048bit-ECFP6 | GBM | 0.870 | 0.865 | 0.924 | 0.783 | 0.822 | 0.722 | 0.854 | 0.815 | 0.007 | 700 0.3 |
| M0617 | 512 | exp05 | 2048bit-ECFP6 | GBM | 0.877 | 0.867 | 0.924 | 0.802 | 0.833 | 0.737 | 0.863 | 0.841 | 0.008 | 200 0.5 |
| M0618 | 512 | exp06 | 2048bit-ECFP6 | GBM | 0.884 | 0.902 | 0.947 | 0.783 | 0.838 | 0.753 | 0.865 | 0.835 | 0.003 | 200 0.5 |
| M0619 | 512 | exp07 | 2048bit-ECFP6 | GBM | 0.895 | 0.943 | 0.971 | 0.774 | 0.850 | 0.779 | 0.873 | 0.833 | 0.017 | 100 0.5 |
| M0620 | 512 | exp08 | 2048bit-ECFP6 | GBM | 0.884 | 0.930 | 0.965 | 0.755 | 0.833 | 0.756 | 0.860 | 0.840 | 0.007 | 300 0.1 |
| M0621 | 512 | exp09 | 2048bit-ECFP6 | GBM | 0.888 | 0.903 | 0.947 | 0.792 | 0.844 | 0.761 | 0.869 | 0.826 | 0.018 | 400 0.1 |
| M0622 | 512 | exp10 | 2048bit-ECFP6 | GBM | 0.895 | 0.923 | 0.959 | 0.792 | 0.853 | 0.777 | 0.875 | 0.828 | 0.025 | 1000 0.1 |
| M0623 | 512 | exp11 | 2048bit-ECFP6 | GBM | 0.851 | 0.865 | 0.929 | 0.726 | 0.790 | 0.682 | 0.828 | 0.842 | 0.051 | 300 0.5 |
| M0624 | 512 | exp12 | 2048bit-ECFP6 | GBM | 0.895 | 0.914 | 0.953 | 0.802 | 0.854 | 0.777 | 0.877 | 0.821 | 0.033 | 200 0.6 |
| M0625 | 512 | exp13 | 2048bit-ECFP6 | GBM | 0.855 | 0.811 | 0.882 | 0.811 | 0.811 | 0.694 | 0.847 | 0.826 | 0.015 | 200 0.6 |
| M0626 | 512 | exp14 | 2048bit-ECFP6 | GBM | 0.873 | 0.890 | 0.941 | 0.764 | 0.822 | 0.730 | 0.853 | 0.838 | 0.016 | 400 0.7 |
| M0627 | 512 | exp15 | 2048bit-ECFP6 | GBM | 0.862 | 0.878 | 0.935 | 0.745 | 0.806 | 0.706 | 0.840 | 0.831 | 0.024 | 50 0.7 |
| M0628 | 512 | exp16 | 2048bit-ECFP6 | GBM | 0.859 | 0.860 | 0.924 | 0.755 | 0.804 | 0.698 | 0.840 | 0.835 | 0.031 | 300 0.9 |
| M0629 | 512 | exp17 | 2048bit-ECFP6 | GBM | 0.866 | 0.856 | 0.918 | 0.783 | 0.818 | 0.714 | 0.851 | 0.815 | 0.003 | 300 0.7 |
| M0630 | 512 | exp18 | 2048bit-ECFP6 | GBM | 0.880 | 0.884 | 0.935 | 0.792 | 0.836 | 0.745 | 0.864 | 0.832 | 0.004 | 100 0.5 |
| M0631 | 512 | exp19 | 2048bit-ECFP6 | GBM | 0.906 | 0.944 | 0.971 | 0.802 | 0.867 | 0.801 | 0.887 | 0.824 | 0.043 | 200 0.2 |
| M0632 | 512 | exp01 | 2048bit-ECFP6 | RF | 0.851 | 0.892 | 0.947 | 0.698 | 0.783 | 0.684 | 0.823 | 0.849 | 0.066 | 10 |
| M0633 | 512 | exp02 | 2048bit-ECFP6 | RF | 0.873 | 0.928 | 0.965 | 0.726 | 0.815 | 0.733 | 0.845 | 0.840 | 0.025 | 600 |
| M0634 | 512 | exp03 | 2048bit-ECFP6 | RF | 0.899 | 0.933 | 0.965 | 0.792 | 0.857 | 0.786 | 0.879 | 0.845 | 0.012 | 400 |
| M0635 | 512 | exp04 | 2048bit-ECFP6 | RF | 0.888 | 0.895 | 0.941 | 0.802 | 0.846 | 0.761 | 0.871 | 0.845 | 0.001 | 100 |
| M0636 | 512 | exp05 | 2048bit-ECFP6 | RF | 0.877 | 0.867 | 0.924 | 0.802 | 0.833 | 0.737 | 0.863 | 0.851 | 0.018 | 800 |
| M0637 | 512 | exp06 | 2048bit-ECFP6 | RF | 0.909 | 0.976 | 0.988 | 0.783 | 0.869 | 0.813 | 0.885 | 0.843 | 0.026 | 100 |
| M0638 | 512 | exp07 | 2048bit-ECFP6 | RF | 0.902 | 0.976 | 0.988 | 0.764 | 0.857 | 0.798 | 0.876 | 0.842 | 0.015 | 300 |
| M0639 | 512 | exp08 | 2048bit-ECFP6 | RF | 0.899 | 0.943 | 0.971 | 0.783 | 0.856 | 0.787 | 0.877 | 0.850 | 0.006 | 900 |
| M0640 | 512 | exp09 | 2048bit-ECFP6 | RF | 0.877 | 0.883 | 0.935 | 0.783 | 0.830 | 0.737 | 0.859 | 0.838 | 0.008 | 50 |
| M0641 | 512 | exp10 | 2048bit-ECFP6 | RF | 0.906 | 0.965 | 0.982 | 0.783 | 0.865 | 0.804 | 0.883 | 0.835 | 0.030 | 50 |
| M0642 | 512 | exp11 | 2048bit-ECFP6 | RF | 0.873 | 0.882 | 0.935 | 0.774 | 0.824 | 0.729 | 0.855 | 0.851 | 0.027 | 10 |
| M0643 | 512 | exp12 | 2048bit-ECFP6 | RF | 0.913 | 0.956 | 0.976 | 0.811 | 0.878 | 0.817 | 0.893 | 0.842 | 0.036 | 100 |
| M0644 | 512 | exp13 | 2048bit-ECFP6 | RF | 0.895 | 0.897 | 0.941 | 0.821 | 0.857 | 0.776 | 0.881 | 0.826 | 0.031 | 300 |
| M0645 | 512 | exp14 | 2048bit-ECFP6 | RF | 0.873 | 0.882 | 0.935 | 0.774 | 0.824 | 0.729 | 0.855 | 0.858 | 0.034 | 800 |
| M0646 | 512 | exp15 | 2048bit-ECFP6 | RF | 0.891 | 0.952 | 0.976 | 0.755 | 0.842 | 0.773 | 0.865 | 0.831 | 0.011 | 400 |
| M0647 | 512 | exp16 | 2048bit-ECFP6 | RF | 0.873 | 0.918 | 0.959 | 0.736 | 0.817 | 0.732 | 0.847 | 0.845 | 0.028 | 200 |
| M0648 | 512 | exp17 | 2048bit-ECFP6 | RF | 0.899 | 0.943 | 0.971 | 0.783 | 0.856 | 0.787 | 0.877 | 0.844 | 0.012 | 1000 |
| M0649 | 512 | exp18 | 2048bit-ECFP6 | RF | 0.895 | 0.943 | 0.971 | 0.774 | 0.850 | 0.779 | 0.873 | 0.850 | 0.000 | 800 |
| M0650 | 512 | exp19 | 2048bit-ECFP6 | RF | 0.902 | 0.916 | 0.953 | 0.821 | 0.866 | 0.792 | 0.887 | 0.841 | 0.025 | 400 |
| M0651 | 512 | exp01 | 2048bit-ECFP6 | DNN2 | 0.873 | 0.908 | 0.953 | 0.745 | 0.819 | 0.731 | 0.849 | 0.847 | 0.028 | 300 100 0.2 |
| M0652 | 512 | exp02 | 2048bit-ECFP6 | DNN2 | 0.884 | 0.911 | 0.953 | 0.774 | 0.837 | 0.754 | 0.863 | 0.849 | 0.012 | 500 120 0.3 |
| M0653 | 512 | exp03 | 2048bit-ECFP6 | DNN2 | 0.884 | 0.894 | 0.941 | 0.792 | 0.840 | 0.753 | 0.867 | 0.845 | 0.005 | 400 160 0.4 |
| M0654 | 512 | exp01 | 2048bit-ECFP6 | DNN3 | 0.873 | 0.882 | 0.935 | 0.774 | 0.824 | 0.729 | 0.855 | 0.852 | 0.028 | 400 60 0.5 |
| M0655 | 512 | exp02 | 2048bit-ECFP6 | DNN3 | 0.884 | 0.894 | 0.941 | 0.792 | 0.840 | 0.753 | 0.867 | 0.855 | 0.015 | 200 80 0.3 |
| M0656 | 512 | exp03 | 2048bit-ECFP6 | DNN3 | 0.880 | 0.876 | 0.929 | 0.802 | 0.837 | 0.745 | 0.866 | 0.849 | 0.012 | 300 140 0.1 |
| M0657 | 256 | exp01 | 1024bit-ECFP4 | KNN | 0.862 | 0.833 | 0.900 | 0.802 | 0.817 | 0.707 | 0.851 | 0.840 | 0.023 | 1 uniform |
| M0658 | 256 | exp02 | 1024bit-ECFP4 | KNN | 0.873 | 0.845 | 0.906 | 0.821 | 0.833 | 0.731 | 0.863 | 0.837 | 0.004 | 3 uniform |
| M0659 | 256 | exp03 | 1024bit-ECFP4 | KNN | 0.880 | 0.869 | 0.924 | 0.811 | 0.839 | 0.745 | 0.868 | 0.836 | 0.003 | 5 distance |
| M0660 | 256 | exp04 | 1024bit-ECFP4 | KNN | 0.880 | 0.812 | 0.871 | 0.896 | 0.852 | 0.755 | 0.883 | 0.824 | 0.028 | 5 uniform |
| M0661 | 256 | exp05 | 1024bit-ECFP4 | KNN | 0.837 | 0.785 | 0.865 | 0.792 | 0.789 | 0.656 | 0.829 | 0.855 | 0.066 | 3 distance |
| M0662 | 256 | exp06 | 1024bit-ECFP4 | KNN | 0.895 | 0.889 | 0.935 | 0.830 | 0.859 | 0.776 | 0.883 | 0.843 | 0.016 | 5 distance |
| M0663 | 256 | exp07 | 1024bit-ECFP4 | KNN | 0.873 | 0.866 | 0.924 | 0.792 | 0.828 | 0.729 | 0.858 | 0.832 | 0.004 | 7 distance |
| M0664 | 256 | exp08 | 1024bit-ECFP4 | KNN | 0.906 | 0.908 | 0.947 | 0.840 | 0.873 | 0.800 | 0.893 | 0.837 | 0.036 | 5 distance |
| M0665 | 256 | exp09 | 1024bit-ECFP4 | KNN | 0.891 | 0.852 | 0.906 | 0.868 | 0.860 | 0.771 | 0.887 | 0.834 | 0.026 | 3 distance |
| M0666 | 256 | exp10 | 1024bit-ECFP4 | KNN | 0.899 | 0.898 | 0.941 | 0.830 | 0.863 | 0.784 | 0.885 | 0.825 | 0.038 | 5 distance |
| M0667 | 256 | exp11 | 1024bit-ECFP4 | KNN | 0.880 | 0.854 | 0.912 | 0.830 | 0.842 | 0.746 | 0.871 | 0.844 | 0.002 | 5 uniform |
| M0668 | 256 | exp12 | 1024bit-ECFP4 | KNN | 0.884 | 0.863 | 0.918 | 0.830 | 0.846 | 0.754 | 0.874 | 0.835 | 0.011 | 3 distance |
| M0669 | 256 | exp13 | 1024bit-ECFP4 | KNN | 0.884 | 0.814 | 0.871 | 0.906 | 0.857 | 0.763 | 0.889 | 0.822 | 0.035 | 3 uniform |
| M0670 | 256 | exp14 | 1024bit-ECFP4 | KNN | 0.833 | 0.738 | 0.806 | 0.877 | 0.802 | 0.667 | 0.842 | 0.839 | 0.037 | 3 distance |
| M0671 | 256 | exp15 | 1024bit-ECFP4 | KNN | 0.859 | 0.838 | 0.906 | 0.783 | 0.810 | 0.699 | 0.845 | 0.827 | 0.017 | 3 uniform |
| M0672 | 256 | exp16 | 1024bit-ECFP4 | KNN | 0.870 | 0.843 | 0.906 | 0.811 | 0.827 | 0.723 | 0.859 | 0.852 | 0.025 | 3 uniform |
| M0673 | 256 | exp17 | 1024bit-ECFP4 | KNN | 0.877 | 0.827 | 0.888 | 0.858 | 0.843 | 0.742 | 0.873 | 0.821 | 0.022 | 3 distance |
| M0674 | 256 | exp18 | 1024bit-ECFP4 | KNN | 0.837 | 0.802 | 0.882 | 0.764 | 0.783 | 0.653 | 0.823 | 0.845 | 0.062 | 7 uniform |
| M0675 | 256 | exp19 | 1024bit-ECFP4 | KNN | 0.902 | 0.891 | 0.935 | 0.849 | 0.870 | 0.792 | 0.892 | 0.830 | 0.040 | 7 distance |
| M0676 | 256 | exp01 | 1024bit-ECFP4 | SVM | 0.877 | 0.919 | 0.959 | 0.745 | 0.823 | 0.739 | 0.852 | 0.860 | 0.037 | 5000.0 0.1 |
| M0677 | 256 | exp02 | 1024bit-ECFP4 | SVM | 0.873 | 0.838 | 0.900 | 0.830 | 0.834 | 0.732 | 0.865 | 0.856 | 0.022 | 5 0.1 |
| M0678 | 256 | exp03 | 1024bit-ECFP4 | SVM | 0.895 | 0.897 | 0.941 | 0.821 | 0.857 | 0.776 | 0.881 | 0.851 | 0.006 | 5 0.1 |
| M0679 | 256 | exp04 | 1024bit-ECFP4 | SVM | 0.895 | 0.847 | 0.900 | 0.887 | 0.866 | 0.780 | 0.893 | 0.855 | 0.011 | 10 0.1 |
| M0680 | 256 | exp05 | 1024bit-ECFP4 | SVM | 0.877 | 0.846 | 0.906 | 0.830 | 0.838 | 0.739 | 0.868 | 0.865 | 0.027 | 5 0.1 |
| M0681 | 256 | exp06 | 1024bit-ECFP4 | SVM | 0.891 | 0.888 | 0.935 | 0.821 | 0.853 | 0.768 | 0.878 | 0.855 | 0.002 | 5 0.05 |
| M0682 | 256 | exp07 | 1024bit-ECFP4 | SVM | 0.884 | 0.870 | 0.924 | 0.821 | 0.845 | 0.753 | 0.873 | 0.847 | 0.002 | 1 0.05 |
| M0683 | 256 | exp08 | 1024bit-ECFP4 | SVM | 0.888 | 0.864 | 0.918 | 0.840 | 0.852 | 0.762 | 0.879 | 0.852 | 0.000 | 5 0.05 |
| M0684 | 256 | exp09 | 1024bit-ECFP4 | SVM | 0.899 | 0.890 | 0.935 | 0.840 | 0.864 | 0.784 | 0.887 | 0.855 | 0.009 | 10 0.05 |
| M0685 | 256 | exp10 | 1024bit-ECFP4 | SVM | 0.888 | 0.887 | 0.935 | 0.811 | 0.847 | 0.761 | 0.873 | 0.843 | 0.004 | 5 0.05 |
| M0686 | 256 | exp11 | 1024bit-ECFP4 | SVM | 0.895 | 0.881 | 0.929 | 0.840 | 0.860 | 0.777 | 0.885 | 0.862 | 0.002 | 1 0.1 |
| M0687 | 256 | exp12 | 1024bit-ECFP4 | SVM | 0.917 | 0.919 | 0.953 | 0.858 | 0.888 | 0.823 | 0.905 | 0.848 | 0.040 | 5 0.05 |
| M0688 | 256 | exp13 | 1024bit-ECFP4 | SVM | 0.891 | 0.845 | 0.900 | 0.877 | 0.861 | 0.772 | 0.889 | 0.849 | 0.012 | 50 0.1 |
| M0689 | 256 | exp14 | 1024bit-ECFP4 | SVM | 0.888 | 0.850 | 0.906 | 0.858 | 0.854 | 0.763 | 0.882 | 0.861 | 0.007 | 5 0.05 |
| M0690 | 256 | exp15 | 1024bit-ECFP4 | SVM | 0.895 | 0.923 | 0.959 | 0.792 | 0.853 | 0.777 | 0.875 | 0.850 | 0.003 | 100000.0 0.1 |
| M0691 | 256 | exp16 | 1024bit-ECFP4 | SVM | 0.888 | 0.895 | 0.941 | 0.802 | 0.846 | 0.761 | 0.871 | 0.860 | 0.014 | 100000.0 0.1 |
| M0692 | 256 | exp17 | 1024bit-ECFP4 | SVM | 0.888 | 0.879 | 0.929 | 0.821 | 0.849 | 0.761 | 0.875 | 0.846 | 0.003 | 10000.0 0.01 |
| M0693 | 256 | exp18 | 1024bit-ECFP4 | SVM | 0.895 | 0.889 | 0.935 | 0.830 | 0.859 | 0.776 | 0.883 | 0.853 | 0.006 | 5 0.05 |
| M0694 | 256 | exp19 | 1024bit-ECFP4 | SVM | 0.924 | 0.913 | 0.947 | 0.887 | 0.900 | 0.839 | 0.917 | 0.846 | 0.054 | 5 0.05 |
| M0695 | 256 | exp01 | 1024bit-ECFP4 | GBM | 0.855 | 0.884 | 0.941 | 0.717 | 0.792 | 0.691 | 0.829 | 0.852 | 0.060 | 200 0.4 |
| M0696 | 256 | exp02 | 1024bit-ECFP4 | GBM | 0.888 | 0.895 | 0.941 | 0.802 | 0.846 | 0.761 | 0.871 | 0.826 | 0.020 | 200 0.8 |
| M0697 | 256 | exp03 | 1024bit-ECFP4 | GBM | 0.888 | 0.895 | 0.941 | 0.802 | 0.846 | 0.761 | 0.871 | 0.838 | 0.008 | 400 0.1 |
| M0698 | 256 | exp04 | 1024bit-ECFP4 | GBM | 0.891 | 0.858 | 0.912 | 0.858 | 0.858 | 0.770 | 0.885 | 0.830 | 0.028 | 600 0.6 |
| M0699 | 256 | exp05 | 1024bit-ECFP4 | GBM | 0.848 | 0.808 | 0.882 | 0.792 | 0.800 | 0.677 | 0.837 | 0.862 | 0.062 | 200 0.5 |
| M0700 | 256 | exp06 | 1024bit-ECFP4 | GBM | 0.880 | 0.901 | 0.947 | 0.774 | 0.832 | 0.746 | 0.861 | 0.848 | 0.016 | 500 0.1 |
| M0701 | 256 | exp07 | 1024bit-ECFP4 | GBM | 0.888 | 0.903 | 0.947 | 0.792 | 0.844 | 0.761 | 0.869 | 0.837 | 0.007 | 200 0.7 |
| M0702 | 256 | exp08 | 1024bit-ECFP4 | GBM | 0.888 | 0.871 | 0.924 | 0.830 | 0.850 | 0.761 | 0.877 | 0.844 | 0.006 | 200 0.7 |
| M0703 | 256 | exp09 | 1024bit-ECFP4 | GBM | 0.891 | 0.896 | 0.941 | 0.811 | 0.851 | 0.768 | 0.876 | 0.841 | 0.010 | 50 0.9 |
| M0704 | 256 | exp10 | 1024bit-ECFP4 | GBM | 0.880 | 0.884 | 0.935 | 0.792 | 0.836 | 0.745 | 0.864 | 0.832 | 0.004 | 500 0.2 |
| M0705 | 256 | exp11 | 1024bit-ECFP4 | GBM | 0.866 | 0.871 | 0.929 | 0.764 | 0.814 | 0.714 | 0.847 | 0.846 | 0.032 | 500 0.1 |
| M0706 | 256 | exp12 | 1024bit-ECFP4 | GBM | 0.913 | 0.910 | 0.947 | 0.858 | 0.883 | 0.815 | 0.902 | 0.835 | 0.048 | 900 0.8 |
| M0707 | 256 | exp13 | 1024bit-ECFP4 | GBM | 0.870 | 0.837 | 0.900 | 0.821 | 0.829 | 0.723 | 0.861 | 0.839 | 0.010 | 300 0.2 |
| M0708 | 256 | exp14 | 1024bit-ECFP4 | GBM | 0.844 | 0.806 | 0.882 | 0.783 | 0.794 | 0.669 | 0.833 | 0.849 | 0.055 | 1000 0.1 |
| M0709 | 256 | exp15 | 1024bit-ECFP4 | GBM | 0.880 | 0.876 | 0.929 | 0.802 | 0.837 | 0.745 | 0.866 | 0.831 | 0.006 | 300 0.6 |
| M0710 | 256 | exp16 | 1024bit-ECFP4 | GBM | 0.848 | 0.856 | 0.924 | 0.726 | 0.786 | 0.674 | 0.825 | 0.834 | 0.048 | 200 0.8 |
| M0711 | 256 | exp17 | 1024bit-ECFP4 | GBM | 0.902 | 0.916 | 0.953 | 0.821 | 0.866 | 0.792 | 0.887 | 0.817 | 0.049 | 200 0.9 |
| M0712 | 256 | exp18 | 1024bit-ECFP4 | GBM | 0.891 | 0.888 | 0.935 | 0.821 | 0.853 | 0.768 | 0.878 | 0.836 | 0.017 | 800 0.2 |
| M0713 | 256 | exp19 | 1024bit-ECFP4 | GBM | 0.880 | 0.901 | 0.947 | 0.774 | 0.832 | 0.746 | 0.861 | 0.820 | 0.012 | 50 0.2 |
| M0714 | 256 | exp01 | 1024bit-ECFP4 | RF | 0.884 | 0.940 | 0.971 | 0.745 | 0.832 | 0.757 | 0.858 | 0.850 | 0.018 | 200 |
| M0715 | 256 | exp02 | 1024bit-ECFP4 | RF | 0.877 | 0.900 | 0.947 | 0.764 | 0.827 | 0.738 | 0.855 | 0.846 | 0.019 | 100 |
| M0716 | 256 | exp03 | 1024bit-ECFP4 | RF | 0.888 | 0.903 | 0.947 | 0.792 | 0.844 | 0.761 | 0.869 | 0.842 | 0.002 | 100 |
| M0717 | 256 | exp04 | 1024bit-ECFP4 | RF | 0.884 | 0.863 | 0.918 | 0.830 | 0.846 | 0.754 | 0.874 | 0.842 | 0.004 | 100 |
| M0718 | 256 | exp05 | 1024bit-ECFP4 | RF | 0.873 | 0.874 | 0.929 | 0.783 | 0.826 | 0.729 | 0.856 | 0.846 | 0.020 | 200 |
| M0719 | 256 | exp06 | 1024bit-ECFP4 | RF | 0.909 | 0.966 | 0.982 | 0.792 | 0.870 | 0.811 | 0.887 | 0.843 | 0.027 | 900 |
| M0720 | 256 | exp07 | 1024bit-ECFP4 | RF | 0.895 | 0.943 | 0.971 | 0.774 | 0.850 | 0.779 | 0.873 | 0.844 | 0.006 | 500 |
| M0721 | 256 | exp08 | 1024bit-ECFP4 | RF | 0.902 | 0.891 | 0.935 | 0.849 | 0.870 | 0.792 | 0.892 | 0.836 | 0.034 | 400 |
| M0722 | 256 | exp09 | 1024bit-ECFP4 | RF | 0.902 | 0.916 | 0.953 | 0.821 | 0.866 | 0.792 | 0.887 | 0.841 | 0.025 | 100 |
| M0723 | 256 | exp10 | 1024bit-ECFP4 | RF | 0.888 | 0.921 | 0.959 | 0.774 | 0.841 | 0.762 | 0.867 | 0.832 | 0.009 | 200 |
| M0724 | 256 | exp11 | 1024bit-ECFP4 | RF | 0.888 | 0.903 | 0.947 | 0.792 | 0.844 | 0.761 | 0.869 | 0.841 | 0.003 | 200 |
| M0725 | 256 | exp12 | 1024bit-ECFP4 | RF | 0.895 | 0.889 | 0.935 | 0.830 | 0.859 | 0.776 | 0.883 | 0.830 | 0.029 | 10 |
| M0726 | 256 | exp13 | 1024bit-ECFP4 | RF | 0.913 | 0.902 | 0.941 | 0.868 | 0.885 | 0.815 | 0.904 | 0.835 | 0.050 | 50 |
| M0727 | 256 | exp14 | 1024bit-ECFP4 | RF | 0.873 | 0.859 | 0.918 | 0.802 | 0.829 | 0.730 | 0.860 | 0.856 | 0.027 | 200 |
| M0728 | 256 | exp15 | 1024bit-ECFP4 | RF | 0.895 | 0.923 | 0.959 | 0.792 | 0.853 | 0.777 | 0.875 | 0.835 | 0.018 | 50 |
| M0729 | 256 | exp16 | 1024bit-ECFP4 | RF | 0.870 | 0.907 | 0.953 | 0.736 | 0.812 | 0.723 | 0.845 | 0.850 | 0.037 | 500 |
| M0730 | 256 | exp17 | 1024bit-ECFP4 | RF | 0.884 | 0.902 | 0.947 | 0.783 | 0.838 | 0.753 | 0.865 | 0.831 | 0.007 | 600 |
| M0731 | 256 | exp18 | 1024bit-ECFP4 | RF | 0.888 | 0.921 | 0.959 | 0.774 | 0.841 | 0.762 | 0.867 | 0.840 | 0.001 | 1000 |
| M0732 | 256 | exp19 | 1024bit-ECFP4 | RF | 0.899 | 0.898 | 0.941 | 0.830 | 0.863 | 0.784 | 0.885 | 0.829 | 0.034 | 200 |
| M0733 | 256 | exp01 | 1024bit-ECFP4 | DNN2 | 0.873 | 0.908 | 0.953 | 0.745 | 0.819 | 0.731 | 0.849 | 0.859 | 0.040 | 500 100 0.3 |
| M0734 | 256 | exp02 | 1024bit-ECFP4 | DNN2 | 0.895 | 0.881 | 0.929 | 0.840 | 0.860 | 0.777 | 0.885 | 0.840 | 0.020 | 300 80 0.4 |
| M0735 | 256 | exp03 | 1024bit-ECFP4 | DNN2 | 0.895 | 0.889 | 0.935 | 0.830 | 0.859 | 0.776 | 0.883 | 0.841 | 0.018 | 600 160 0.5 |
| M0736 | 256 | exp01 | 1024bit-ECFP4 | DNN3 | 0.870 | 0.898 | 0.947 | 0.745 | 0.814 | 0.723 | 0.846 | 0.859 | 0.045 | 100 140 0.2 |
| M0737 | 256 | exp02 | 1024bit-ECFP4 | DNN3 | 0.902 | 0.899 | 0.941 | 0.840 | 0.868 | 0.792 | 0.890 | 0.840 | 0.028 | 100 160 0.2 |
| M0738 | 256 | exp03 | 1024bit-ECFP4 | DNN3 | 0.902 | 0.907 | 0.947 | 0.830 | 0.867 | 0.792 | 0.888 | 0.844 | 0.023 | 100 80 0.2 |
| M0739 | 256 | exp01 | 2048bit-ECFP4 | KNN | 0.877 | 0.891 | 0.941 | 0.774 | 0.828 | 0.737 | 0.857 | 0.843 | 0.015 | 3 distance |
| M0740 | 256 | exp02 | 2048bit-ECFP4 | KNN | 0.873 | 0.874 | 0.929 | 0.783 | 0.826 | 0.729 | 0.856 | 0.843 | 0.017 | 5 distance |
| M0741 | 256 | exp03 | 2048bit-ECFP4 | KNN | 0.866 | 0.829 | 0.894 | 0.821 | 0.825 | 0.716 | 0.857 | 0.840 | 0.015 | 3 distance |
| M0742 | 256 | exp04 | 2048bit-ECFP4 | KNN | 0.851 | 0.783 | 0.853 | 0.849 | 0.814 | 0.693 | 0.851 | 0.834 | 0.020 | 5 distance |
| M0743 | 256 | exp05 | 2048bit-ECFP4 | KNN | 0.855 | 0.806 | 0.876 | 0.821 | 0.813 | 0.695 | 0.849 | 0.842 | 0.029 | 7 distance |
| M0744 | 256 | exp06 | 2048bit-ECFP4 | KNN | 0.902 | 0.899 | 0.941 | 0.840 | 0.868 | 0.792 | 0.890 | 0.844 | 0.024 | 5 uniform |
| M0745 | 256 | exp07 | 2048bit-ECFP4 | KNN | 0.884 | 0.902 | 0.947 | 0.783 | 0.838 | 0.753 | 0.865 | 0.834 | 0.004 | 7 distance |
| M0746 | 256 | exp08 | 2048bit-ECFP4 | KNN | 0.891 | 0.896 | 0.941 | 0.811 | 0.851 | 0.768 | 0.876 | 0.841 | 0.010 | 5 distance |
| M0747 | 256 | exp09 | 2048bit-ECFP4 | KNN | 0.859 | 0.819 | 0.888 | 0.811 | 0.815 | 0.701 | 0.850 | 0.835 | 0.020 | 5 distance |
| M0748 | 256 | exp10 | 2048bit-ECFP4 | KNN | 0.902 | 0.925 | 0.959 | 0.811 | 0.864 | 0.792 | 0.885 | 0.829 | 0.035 | 3 uniform |
| M0749 | 256 | exp11 | 2048bit-ECFP4 | KNN | 0.851 | 0.804 | 0.876 | 0.811 | 0.808 | 0.687 | 0.844 | 0.849 | 0.041 | 1 uniform |
| M0750 | 256 | exp12 | 2048bit-ECFP4 | KNN | 0.888 | 0.871 | 0.924 | 0.830 | 0.850 | 0.761 | 0.877 | 0.839 | 0.011 | 3 distance |
| M0751 | 256 | exp13 | 2048bit-ECFP4 | KNN | 0.851 | 0.788 | 0.859 | 0.840 | 0.813 | 0.691 | 0.849 | 0.837 | 0.024 | 3 uniform |
| M0752 | 256 | exp14 | 2048bit-ECFP4 | KNN | 0.841 | 0.772 | 0.847 | 0.830 | 0.800 | 0.669 | 0.839 | 0.852 | 0.052 | 5 distance |
| M0753 | 256 | exp15 | 2048bit-ECFP4 | KNN | 0.873 | 0.874 | 0.929 | 0.783 | 0.826 | 0.729 | 0.856 | 0.838 | 0.012 | 3 distance |
| M0754 | 256 | exp16 | 2048bit-ECFP4 | KNN | 0.866 | 0.842 | 0.906 | 0.802 | 0.821 | 0.715 | 0.854 | 0.852 | 0.031 | 3 distance |
| M0755 | 256 | exp17 | 2048bit-ECFP4 | KNN | 0.888 | 0.857 | 0.912 | 0.849 | 0.853 | 0.762 | 0.881 | 0.836 | 0.017 | 1 uniform |
| M0756 | 256 | exp18 | 2048bit-ECFP4 | KNN | 0.862 | 0.840 | 0.906 | 0.792 | 0.816 | 0.707 | 0.849 | 0.854 | 0.038 | 5 uniform |
| M0757 | 256 | exp19 | 2048bit-ECFP4 | KNN | 0.895 | 0.889 | 0.935 | 0.830 | 0.859 | 0.776 | 0.883 | 0.835 | 0.024 | 7 distance |
| M0758 | 256 | exp01 | 2048bit-ECFP4 | SVM | 0.873 | 0.899 | 0.947 | 0.755 | 0.821 | 0.730 | 0.851 | 0.861 | 0.040 | 1 0.1 |
| M0759 | 256 | exp02 | 2048bit-ECFP4 | SVM | 0.877 | 0.853 | 0.912 | 0.821 | 0.837 | 0.738 | 0.867 | 0.857 | 0.020 | 5 0.1 |
| M0760 | 256 | exp03 | 2048bit-ECFP4 | SVM | 0.884 | 0.863 | 0.918 | 0.830 | 0.846 | 0.754 | 0.874 | 0.855 | 0.009 | 10 0.01 |
| M0761 | 256 | exp04 | 2048bit-ECFP4 | SVM | 0.888 | 0.850 | 0.906 | 0.858 | 0.854 | 0.763 | 0.882 | 0.860 | 0.006 | 50000.0 0.1 |
| M0762 | 256 | exp05 | 2048bit-ECFP4 | SVM | 0.888 | 0.850 | 0.906 | 0.858 | 0.854 | 0.763 | 0.882 | 0.873 | 0.019 | 100 0.1 |
| M0763 | 256 | exp06 | 2048bit-ECFP4 | SVM | 0.899 | 0.915 | 0.953 | 0.811 | 0.860 | 0.784 | 0.882 | 0.861 | 0.001 | 50 0.1 |
| M0764 | 256 | exp07 | 2048bit-ECFP4 | SVM | 0.902 | 0.899 | 0.941 | 0.840 | 0.868 | 0.792 | 0.890 | 0.855 | 0.013 | 5 0.1 |
| M0765 | 256 | exp08 | 2048bit-ECFP4 | SVM | 0.891 | 0.845 | 0.900 | 0.877 | 0.861 | 0.772 | 0.889 | 0.858 | 0.003 | 50000.0 0.05 |
| M0766 | 256 | exp09 | 2048bit-ECFP4 | SVM | 0.888 | 0.871 | 0.924 | 0.830 | 0.850 | 0.761 | 0.877 | 0.860 | 0.010 | 5 0.05 |
| M0767 | 256 | exp10 | 2048bit-ECFP4 | SVM | 0.906 | 0.908 | 0.947 | 0.840 | 0.873 | 0.800 | 0.893 | 0.839 | 0.034 | 5 0.05 |
| M0768 | 256 | exp11 | 2048bit-ECFP4 | SVM | 0.866 | 0.856 | 0.918 | 0.783 | 0.818 | 0.714 | 0.851 | 0.858 | 0.040 | 1 0.05 |
| M0769 | 256 | exp12 | 2048bit-ECFP4 | SVM | 0.895 | 0.881 | 0.929 | 0.840 | 0.860 | 0.777 | 0.885 | 0.851 | 0.009 | 5 0.1 |
| M0770 | 256 | exp13 | 2048bit-ECFP4 | SVM | 0.873 | 0.814 | 0.876 | 0.868 | 0.840 | 0.736 | 0.872 | 0.859 | 0.019 | 1000.0 0.1 |
| M0771 | 256 | exp14 | 2048bit-ECFP4 | SVM | 0.873 | 0.832 | 0.894 | 0.840 | 0.836 | 0.732 | 0.867 | 0.875 | 0.039 | 5 0.05 |
| M0772 | 256 | exp15 | 2048bit-ECFP4 | SVM | 0.899 | 0.898 | 0.941 | 0.830 | 0.863 | 0.784 | 0.885 | 0.844 | 0.019 | 5 0.05 |
| M0773 | 256 | exp16 | 2048bit-ECFP4 | SVM | 0.891 | 0.904 | 0.947 | 0.802 | 0.850 | 0.769 | 0.875 | 0.862 | 0.012 | 5 0.1 |
| M0774 | 256 | exp17 | 2048bit-ECFP4 | SVM | 0.880 | 0.861 | 0.918 | 0.821 | 0.841 | 0.746 | 0.869 | 0.848 | 0.007 | 500 0.05 |
| M0775 | 256 | exp18 | 2048bit-ECFP4 | SVM | 0.891 | 0.880 | 0.929 | 0.830 | 0.854 | 0.769 | 0.879 | 0.862 | 0.008 | 5 0.1 |
| M0776 | 256 | exp19 | 2048bit-ECFP4 | SVM | 0.909 | 0.909 | 0.947 | 0.849 | 0.878 | 0.807 | 0.898 | 0.840 | 0.038 | 1 0.1 |
| M0777 | 256 | exp01 | 2048bit-ECFP4 | GBM | 0.866 | 0.879 | 0.935 | 0.755 | 0.812 | 0.714 | 0.845 | 0.862 | 0.050 | 600 0.1 |
| M0778 | 256 | exp02 | 2048bit-ECFP4 | GBM | 0.888 | 0.921 | 0.959 | 0.774 | 0.841 | 0.762 | 0.867 | 0.842 | 0.001 | 700 0.1 |
| M0779 | 256 | exp03 | 2048bit-ECFP4 | GBM | 0.880 | 0.854 | 0.912 | 0.830 | 0.842 | 0.746 | 0.871 | 0.850 | 0.008 | 300 0.5 |
| M0780 | 256 | exp04 | 2048bit-ECFP4 | GBM | 0.877 | 0.840 | 0.900 | 0.840 | 0.840 | 0.740 | 0.870 | 0.841 | 0.001 | 50 0.4 |
| M0781 | 256 | exp05 | 2048bit-ECFP4 | GBM | 0.884 | 0.843 | 0.900 | 0.858 | 0.850 | 0.756 | 0.879 | 0.862 | 0.012 | 100 0.5 |
| M0782 | 256 | exp06 | 2048bit-ECFP4 | GBM | 0.895 | 0.914 | 0.953 | 0.802 | 0.854 | 0.777 | 0.877 | 0.846 | 0.008 | 300 0.1 |
| M0783 | 256 | exp07 | 2048bit-ECFP4 | GBM | 0.891 | 0.904 | 0.947 | 0.802 | 0.850 | 0.769 | 0.875 | 0.841 | 0.009 | 100 0.7 |
| M0784 | 256 | exp08 | 2048bit-ECFP4 | GBM | 0.895 | 0.897 | 0.941 | 0.821 | 0.857 | 0.776 | 0.881 | 0.850 | 0.007 | 300 0.3 |
| M0785 | 256 | exp09 | 2048bit-ECFP4 | GBM | 0.891 | 0.880 | 0.929 | 0.830 | 0.854 | 0.769 | 0.879 | 0.857 | 0.003 | 100 0.6 |
| M0786 | 256 | exp10 | 2048bit-ECFP4 | GBM | 0.906 | 0.926 | 0.959 | 0.821 | 0.870 | 0.800 | 0.890 | 0.850 | 0.020 | 300 0.1 |
| M0787 | 256 | exp11 | 2048bit-ECFP4 | GBM | 0.855 | 0.867 | 0.929 | 0.736 | 0.796 | 0.690 | 0.833 | 0.854 | 0.058 | 100 0.5 |
| M0788 | 256 | exp12 | 2048bit-ECFP4 | GBM | 0.902 | 0.883 | 0.929 | 0.858 | 0.871 | 0.792 | 0.893 | 0.840 | 0.031 | 50 0.7 |
| M0789 | 256 | exp13 | 2048bit-ECFP4 | GBM | 0.870 | 0.824 | 0.888 | 0.840 | 0.832 | 0.725 | 0.864 | 0.848 | 0.016 | 300 0.4 |
| M0790 | 256 | exp14 | 2048bit-ECFP4 | GBM | 0.866 | 0.842 | 0.906 | 0.802 | 0.821 | 0.715 | 0.854 | 0.848 | 0.027 | 100 0.6 |
| M0791 | 256 | exp15 | 2048bit-ECFP4 | GBM | 0.902 | 0.916 | 0.953 | 0.821 | 0.866 | 0.792 | 0.887 | 0.844 | 0.022 | 50 0.9 |
| M0792 | 256 | exp16 | 2048bit-ECFP4 | GBM | 0.859 | 0.860 | 0.924 | 0.755 | 0.804 | 0.698 | 0.840 | 0.846 | 0.042 | 400 0.2 |
| M0793 | 256 | exp17 | 2048bit-ECFP4 | GBM | 0.870 | 0.865 | 0.924 | 0.783 | 0.822 | 0.722 | 0.854 | 0.833 | 0.011 | 200 0.3 |
| M0794 | 256 | exp18 | 2048bit-ECFP4 | GBM | 0.877 | 0.867 | 0.924 | 0.802 | 0.833 | 0.737 | 0.863 | 0.856 | 0.023 | 400 0.7 |
| M0795 | 256 | exp19 | 2048bit-ECFP4 | GBM | 0.906 | 0.926 | 0.959 | 0.821 | 0.870 | 0.800 | 0.890 | 0.837 | 0.033 | 50 0.2 |
| M0796 | 256 | exp01 | 2048bit-ECFP4 | RF | 0.873 | 0.918 | 0.959 | 0.736 | 0.817 | 0.732 | 0.847 | 0.850 | 0.033 | 1000 |
| M0797 | 256 | exp02 | 2048bit-ECFP4 | RF | 0.877 | 0.900 | 0.947 | 0.764 | 0.827 | 0.738 | 0.855 | 0.841 | 0.014 | 700 |
| M0798 | 256 | exp03 | 2048bit-ECFP4 | RF | 0.884 | 0.885 | 0.935 | 0.802 | 0.842 | 0.753 | 0.869 | 0.850 | 0.008 | 1000 |
| M0799 | 256 | exp04 | 2048bit-ECFP4 | RF | 0.880 | 0.861 | 0.918 | 0.821 | 0.841 | 0.746 | 0.869 | 0.850 | 0.009 | 800 |
| M0800 | 256 | exp05 | 2048bit-ECFP4 | RF | 0.862 | 0.827 | 0.894 | 0.811 | 0.819 | 0.708 | 0.853 | 0.860 | 0.041 | 100 |
| M0801 | 256 | exp06 | 2048bit-ECFP4 | RF | 0.891 | 0.913 | 0.953 | 0.792 | 0.848 | 0.769 | 0.873 | 0.849 | 0.001 | 200 |
| M0802 | 256 | exp07 | 2048bit-ECFP4 | RF | 0.888 | 0.912 | 0.953 | 0.783 | 0.843 | 0.761 | 0.868 | 0.841 | 0.002 | 50 |
| M0803 | 256 | exp08 | 2048bit-ECFP4 | RF | 0.895 | 0.874 | 0.924 | 0.849 | 0.861 | 0.777 | 0.887 | 0.848 | 0.013 | 100 |
| M0804 | 256 | exp09 | 2048bit-ECFP4 | RF | 0.895 | 0.897 | 0.941 | 0.821 | 0.857 | 0.776 | 0.881 | 0.835 | 0.022 | 800 |
| M0805 | 256 | exp10 | 2048bit-ECFP4 | RF | 0.888 | 0.895 | 0.941 | 0.802 | 0.846 | 0.761 | 0.871 | 0.830 | 0.016 | 10 |
| M0806 | 256 | exp11 | 2048bit-ECFP4 | RF | 0.884 | 0.911 | 0.953 | 0.774 | 0.837 | 0.754 | 0.863 | 0.849 | 0.012 | 900 |
| M0807 | 256 | exp12 | 2048bit-ECFP4 | RF | 0.902 | 0.925 | 0.959 | 0.811 | 0.864 | 0.792 | 0.885 | 0.835 | 0.029 | 300 |
| M0808 | 256 | exp13 | 2048bit-ECFP4 | RF | 0.891 | 0.865 | 0.918 | 0.849 | 0.857 | 0.770 | 0.883 | 0.840 | 0.017 | 300 |
| M0809 | 256 | exp14 | 2048bit-ECFP4 | RF | 0.880 | 0.876 | 0.929 | 0.802 | 0.837 | 0.745 | 0.866 | 0.859 | 0.022 | 500 |
| M0810 | 256 | exp15 | 2048bit-ECFP4 | RF | 0.899 | 0.943 | 0.971 | 0.783 | 0.856 | 0.787 | 0.877 | 0.841 | 0.015 | 50 |
| M0811 | 256 | exp16 | 2048bit-ECFP4 | RF | 0.873 | 0.908 | 0.953 | 0.745 | 0.819 | 0.731 | 0.849 | 0.845 | 0.026 | 100 |
| M0812 | 256 | exp17 | 2048bit-ECFP4 | RF | 0.870 | 0.830 | 0.894 | 0.830 | 0.830 | 0.724 | 0.862 | 0.841 | 0.011 | 10 |
| M0813 | 256 | exp18 | 2048bit-ECFP4 | RF | 0.902 | 0.944 | 0.971 | 0.792 | 0.862 | 0.794 | 0.881 | 0.853 | 0.009 | 700 |
| M0814 | 256 | exp19 | 2048bit-ECFP4 | RF | 0.909 | 0.918 | 0.953 | 0.840 | 0.877 | 0.807 | 0.896 | 0.832 | 0.045 | 300 |
| M0815 | 256 | exp01 | 2048bit-ECFP4 | DNN2 | 0.888 | 0.912 | 0.953 | 0.783 | 0.843 | 0.761 | 0.868 | 0.859 | 0.016 | 600 160 0.4 |
| M0816 | 256 | exp02 | 2048bit-ECFP4 | DNN2 | 0.870 | 0.837 | 0.900 | 0.821 | 0.829 | 0.723 | 0.861 | 0.857 | 0.028 | 400 140 0.3 |
| M0817 | 256 | exp03 | 2048bit-ECFP4 | DNN2 | 0.866 | 0.842 | 0.906 | 0.802 | 0.821 | 0.715 | 0.854 | 0.856 | 0.035 | 600 140 0.1 |
| M0818 | 256 | exp01 | 2048bit-ECFP4 | DNN3 | 0.873 | 0.882 | 0.935 | 0.774 | 0.824 | 0.729 | 0.855 | 0.856 | 0.032 | 400 60 0.3 |
| M0819 | 256 | exp02 | 2048bit-ECFP4 | DNN3 | 0.880 | 0.854 | 0.912 | 0.830 | 0.842 | 0.746 | 0.871 | 0.854 | 0.012 | 300 160 0.3 |
| M0820 | 256 | exp03 | 2048bit-ECFP4 | DNN3 | 0.880 | 0.861 | 0.918 | 0.821 | 0.841 | 0.746 | 0.869 | 0.860 | 0.019 | 100 160 0.5 |
| M0821 | 256 | exp01 | 1024bit-ECFP6 | KNN | 0.862 | 0.854 | 0.918 | 0.774 | 0.812 | 0.706 | 0.846 | 0.843 | 0.031 | 3 distance |
| M0822 | 256 | exp02 | 1024bit-ECFP6 | KNN | 0.888 | 0.879 | 0.929 | 0.821 | 0.849 | 0.761 | 0.875 | 0.835 | 0.014 | 3 uniform |
| M0823 | 256 | exp03 | 1024bit-ECFP6 | KNN | 0.870 | 0.850 | 0.912 | 0.802 | 0.825 | 0.722 | 0.857 | 0.841 | 0.016 | 5 distance |
| M0824 | 256 | exp04 | 1024bit-ECFP6 | KNN | 0.848 | 0.758 | 0.824 | 0.887 | 0.817 | 0.695 | 0.855 | 0.830 | 0.013 | 1 uniform |
| M0825 | 256 | exp05 | 1024bit-ECFP6 | KNN | 0.822 | 0.757 | 0.841 | 0.792 | 0.774 | 0.629 | 0.817 | 0.842 | 0.068 | 3 distance |
| M0826 | 256 | exp06 | 1024bit-ECFP6 | KNN | 0.906 | 0.908 | 0.947 | 0.840 | 0.873 | 0.800 | 0.893 | 0.829 | 0.044 | 5 distance |
| M0827 | 256 | exp07 | 1024bit-ECFP6 | KNN | 0.873 | 0.874 | 0.929 | 0.783 | 0.826 | 0.729 | 0.856 | 0.833 | 0.007 | 7 distance |
| M0828 | 256 | exp08 | 1024bit-ECFP6 | KNN | 0.884 | 0.819 | 0.876 | 0.896 | 0.856 | 0.761 | 0.886 | 0.829 | 0.027 | 1 uniform |
| M0829 | 256 | exp09 | 1024bit-ECFP6 | KNN | 0.855 | 0.817 | 0.888 | 0.802 | 0.810 | 0.693 | 0.845 | 0.843 | 0.033 | 5 uniform |
| M0830 | 256 | exp10 | 1024bit-ECFP6 | KNN | 0.899 | 0.898 | 0.941 | 0.830 | 0.863 | 0.784 | 0.885 | 0.816 | 0.047 | 7 distance |
| M0831 | 256 | exp11 | 1024bit-ECFP6 | KNN | 0.888 | 0.887 | 0.935 | 0.811 | 0.847 | 0.761 | 0.873 | 0.843 | 0.004 | 5 uniform |
| M0832 | 256 | exp12 | 1024bit-ECFP6 | KNN | 0.895 | 0.867 | 0.918 | 0.858 | 0.863 | 0.778 | 0.888 | 0.837 | 0.026 | 3 distance |
| M0833 | 256 | exp13 | 1024bit-ECFP6 | KNN | 0.848 | 0.776 | 0.847 | 0.849 | 0.811 | 0.686 | 0.848 | 0.825 | 0.014 | 3 distance |
| M0834 | 256 | exp14 | 1024bit-ECFP6 | KNN | 0.862 | 0.779 | 0.841 | 0.896 | 0.833 | 0.722 | 0.869 | 0.841 | 0.008 | 3 uniform |
| M0835 | 256 | exp15 | 1024bit-ECFP6 | KNN | 0.866 | 0.856 | 0.918 | 0.783 | 0.818 | 0.714 | 0.851 | 0.836 | 0.018 | 3 distance |
| M0836 | 256 | exp16 | 1024bit-ECFP6 | KNN | 0.848 | 0.827 | 0.900 | 0.764 | 0.794 | 0.675 | 0.832 | 0.837 | 0.042 | 3 uniform |
| M0837 | 256 | exp17 | 1024bit-ECFP6 | KNN | 0.837 | 0.770 | 0.847 | 0.821 | 0.795 | 0.661 | 0.834 | 0.834 | 0.039 | 1 uniform |
| M0838 | 256 | exp18 | 1024bit-ECFP6 | KNN | 0.855 | 0.837 | 0.906 | 0.774 | 0.804 | 0.691 | 0.840 | 0.844 | 0.039 | 5 uniform |
| M0839 | 256 | exp19 | 1024bit-ECFP6 | KNN | 0.891 | 0.858 | 0.912 | 0.858 | 0.858 | 0.770 | 0.885 | 0.820 | 0.038 | 3 uniform |
| M0840 | 256 | exp01 | 1024bit-ECFP6 | SVM | 0.862 | 0.878 | 0.935 | 0.745 | 0.806 | 0.706 | 0.840 | 0.862 | 0.056 | 50 0.1 |
| M0841 | 256 | exp02 | 1024bit-ECFP6 | SVM | 0.880 | 0.869 | 0.924 | 0.811 | 0.839 | 0.745 | 0.868 | 0.848 | 0.009 | 10 0.1 |
| M0842 | 256 | exp03 | 1024bit-ECFP6 | SVM | 0.888 | 0.887 | 0.935 | 0.811 | 0.847 | 0.761 | 0.873 | 0.845 | 0.002 | 5 0.1 |
| M0843 | 256 | exp04 | 1024bit-ECFP6 | SVM | 0.888 | 0.850 | 0.906 | 0.858 | 0.854 | 0.763 | 0.882 | 0.852 | 0.002 | 10 0.1 |
| M0844 | 256 | exp05 | 1024bit-ECFP6 | SVM | 0.862 | 0.840 | 0.906 | 0.792 | 0.816 | 0.707 | 0.849 | 0.861 | 0.045 | 5 0.1 |
| M0845 | 256 | exp06 | 1024bit-ECFP6 | SVM | 0.891 | 0.922 | 0.959 | 0.783 | 0.847 | 0.770 | 0.871 | 0.848 | 0.001 | 50000.0 0.1 |
| M0846 | 256 | exp07 | 1024bit-ECFP6 | SVM | 0.884 | 0.902 | 0.947 | 0.783 | 0.838 | 0.753 | 0.865 | 0.853 | 0.015 | 5 0.05 |
| M0847 | 256 | exp08 | 1024bit-ECFP6 | SVM | 0.899 | 0.924 | 0.959 | 0.802 | 0.859 | 0.785 | 0.881 | 0.847 | 0.012 | 1 0.1 |
| M0848 | 256 | exp09 | 1024bit-ECFP6 | SVM | 0.895 | 0.881 | 0.929 | 0.840 | 0.860 | 0.777 | 0.885 | 0.839 | 0.021 | 500 0.05 |
| M0849 | 256 | exp10 | 1024bit-ECFP6 | SVM | 0.899 | 0.924 | 0.959 | 0.802 | 0.859 | 0.785 | 0.881 | 0.856 | 0.003 | 1 0.1 |
| M0850 | 256 | exp11 | 1024bit-ECFP6 | SVM | 0.877 | 0.875 | 0.929 | 0.792 | 0.832 | 0.737 | 0.861 | 0.851 | 0.019 | 50000.0 0.05 |
| M0851 | 256 | exp12 | 1024bit-ECFP6 | SVM | 0.906 | 0.892 | 0.935 | 0.858 | 0.875 | 0.800 | 0.897 | 0.847 | 0.028 | 500 0.1 |
| M0852 | 256 | exp13 | 1024bit-ECFP6 | SVM | 0.899 | 0.855 | 0.906 | 0.887 | 0.870 | 0.787 | 0.897 | 0.838 | 0.032 | 50000.0 0.1 |
| M0853 | 256 | exp14 | 1024bit-ECFP6 | SVM | 0.873 | 0.832 | 0.894 | 0.840 | 0.836 | 0.732 | 0.867 | 0.863 | 0.027 | 500 0.1 |
| M0854 | 256 | exp15 | 1024bit-ECFP6 | SVM | 0.880 | 0.920 | 0.959 | 0.755 | 0.829 | 0.747 | 0.857 | 0.849 | 0.020 | 5000.0 0.1 |
| M0855 | 256 | exp16 | 1024bit-ECFP6 | SVM | 0.877 | 0.891 | 0.941 | 0.774 | 0.828 | 0.737 | 0.857 | 0.855 | 0.027 | 5000.0 0.1 |
| M0856 | 256 | exp17 | 1024bit-ECFP6 | SVM | 0.888 | 0.857 | 0.912 | 0.849 | 0.853 | 0.762 | 0.881 | 0.849 | 0.004 | 10 0.1 |
| M0857 | 256 | exp18 | 1024bit-ECFP6 | SVM | 0.873 | 0.851 | 0.912 | 0.811 | 0.831 | 0.730 | 0.862 | 0.854 | 0.023 | 1 0.05 |
| M0858 | 256 | exp19 | 1024bit-ECFP6 | SVM | 0.920 | 0.920 | 0.953 | 0.868 | 0.893 | 0.831 | 0.910 | 0.836 | 0.057 | 100 0.1 |
| M0859 | 256 | exp01 | 1024bit-ECFP6 | GBM | 0.848 | 0.864 | 0.929 | 0.717 | 0.784 | 0.675 | 0.823 | 0.832 | 0.048 | 300 0.6 |
| M0860 | 256 | exp02 | 1024bit-ECFP6 | GBM | 0.884 | 0.911 | 0.953 | 0.774 | 0.837 | 0.754 | 0.863 | 0.822 | 0.015 | 200 0.2 |
| M0861 | 256 | exp03 | 1024bit-ECFP6 | GBM | 0.866 | 0.829 | 0.894 | 0.821 | 0.825 | 0.716 | 0.857 | 0.836 | 0.011 | 200 0.5 |
| M0862 | 256 | exp04 | 1024bit-ECFP6 | GBM | 0.891 | 0.913 | 0.953 | 0.792 | 0.848 | 0.769 | 0.873 | 0.822 | 0.026 | 200 0.1 |
| M0863 | 256 | exp05 | 1024bit-ECFP6 | GBM | 0.859 | 0.825 | 0.894 | 0.802 | 0.813 | 0.700 | 0.848 | 0.841 | 0.028 | 200 0.4 |
| M0864 | 256 | exp06 | 1024bit-ECFP6 | GBM | 0.877 | 0.883 | 0.935 | 0.783 | 0.830 | 0.737 | 0.859 | 0.834 | 0.004 | 100 0.6 |
| M0865 | 256 | exp07 | 1024bit-ECFP6 | GBM | 0.859 | 0.885 | 0.941 | 0.726 | 0.798 | 0.699 | 0.833 | 0.822 | 0.024 | 100 0.9 |
| M0866 | 256 | exp08 | 1024bit-ECFP6 | GBM | 0.873 | 0.859 | 0.918 | 0.802 | 0.829 | 0.730 | 0.860 | 0.835 | 0.006 | 200 0.9 |
| M0867 | 256 | exp09 | 1024bit-ECFP6 | GBM | 0.902 | 0.916 | 0.953 | 0.821 | 0.866 | 0.792 | 0.887 | 0.816 | 0.050 | 500 0.5 |
| M0868 | 256 | exp10 | 1024bit-ECFP6 | GBM | 0.895 | 0.905 | 0.947 | 0.811 | 0.856 | 0.776 | 0.879 | 0.833 | 0.023 | 500 0.2 |
| M0869 | 256 | exp11 | 1024bit-ECFP6 | GBM | 0.851 | 0.874 | 0.935 | 0.717 | 0.788 | 0.683 | 0.826 | 0.844 | 0.056 | 200 0.1 |
| M0870 | 256 | exp12 | 1024bit-ECFP6 | GBM | 0.891 | 0.913 | 0.953 | 0.792 | 0.848 | 0.769 | 0.873 | 0.832 | 0.016 | 50 0.5 |
| M0871 | 256 | exp13 | 1024bit-ECFP6 | GBM | 0.873 | 0.814 | 0.876 | 0.868 | 0.840 | 0.736 | 0.872 | 0.821 | 0.019 | 100 0.5 |
| M0872 | 256 | exp14 | 1024bit-ECFP6 | GBM | 0.837 | 0.802 | 0.882 | 0.764 | 0.783 | 0.653 | 0.823 | 0.846 | 0.063 | 300 0.1 |
| M0873 | 256 | exp15 | 1024bit-ECFP6 | GBM | 0.877 | 0.919 | 0.959 | 0.745 | 0.823 | 0.739 | 0.852 | 0.821 | 0.002 | 300 0.8 |
| M0874 | 256 | exp16 | 1024bit-ECFP6 | GBM | 0.873 | 0.949 | 0.976 | 0.708 | 0.811 | 0.736 | 0.842 | 0.825 | 0.014 | 200 0.1 |
| M0875 | 256 | exp17 | 1024bit-ECFP6 | GBM | 0.888 | 0.895 | 0.941 | 0.802 | 0.846 | 0.761 | 0.871 | 0.824 | 0.022 | 200 0.1 |
| M0876 | 256 | exp18 | 1024bit-ECFP6 | GBM | 0.884 | 0.920 | 0.959 | 0.764 | 0.835 | 0.755 | 0.861 | 0.835 | 0.000 | 400 0.1 |
| M0877 | 256 | exp19 | 1024bit-ECFP6 | GBM | 0.899 | 0.924 | 0.959 | 0.802 | 0.859 | 0.785 | 0.881 | 0.819 | 0.040 | 50 0.4 |
| M0878 | 256 | exp01 | 1024bit-ECFP6 | RF | 0.859 | 0.924 | 0.965 | 0.689 | 0.789 | 0.703 | 0.827 | 0.850 | 0.061 | 50 |
| M0879 | 256 | exp02 | 1024bit-ECFP6 | RF | 0.895 | 0.953 | 0.976 | 0.764 | 0.848 | 0.780 | 0.870 | 0.830 | 0.018 | 100 |
| M0880 | 256 | exp03 | 1024bit-ECFP6 | RF | 0.895 | 0.905 | 0.947 | 0.811 | 0.856 | 0.776 | 0.879 | 0.835 | 0.021 | 100 |
| M0881 | 256 | exp04 | 1024bit-ECFP6 | RF | 0.899 | 0.915 | 0.953 | 0.811 | 0.860 | 0.784 | 0.882 | 0.831 | 0.029 | 500 |
| M0882 | 256 | exp05 | 1024bit-ECFP6 | RF | 0.859 | 0.838 | 0.906 | 0.783 | 0.810 | 0.699 | 0.845 | 0.842 | 0.032 | 800 |
| M0883 | 256 | exp06 | 1024bit-ECFP6 | RF | 0.902 | 0.965 | 0.982 | 0.774 | 0.859 | 0.796 | 0.878 | 0.840 | 0.019 | 300 |
| M0884 | 256 | exp07 | 1024bit-ECFP6 | RF | 0.891 | 0.952 | 0.976 | 0.755 | 0.842 | 0.773 | 0.865 | 0.844 | 0.002 | 100 |
| M0885 | 256 | exp08 | 1024bit-ECFP6 | RF | 0.895 | 0.943 | 0.971 | 0.774 | 0.850 | 0.779 | 0.873 | 0.841 | 0.009 | 300 |
| M0886 | 256 | exp09 | 1024bit-ECFP6 | RF | 0.902 | 0.925 | 0.959 | 0.811 | 0.864 | 0.792 | 0.885 | 0.833 | 0.031 | 200 |
| M0887 | 256 | exp10 | 1024bit-ECFP6 | RF | 0.902 | 0.954 | 0.976 | 0.783 | 0.860 | 0.795 | 0.879 | 0.833 | 0.027 | 900 |
| M0888 | 256 | exp11 | 1024bit-ECFP6 | RF | 0.870 | 0.907 | 0.953 | 0.736 | 0.812 | 0.723 | 0.845 | 0.842 | 0.030 | 300 |
| M0889 | 256 | exp12 | 1024bit-ECFP6 | RF | 0.909 | 0.909 | 0.947 | 0.849 | 0.878 | 0.807 | 0.898 | 0.841 | 0.037 | 50 |
| M0890 | 256 | exp13 | 1024bit-ECFP6 | RF | 0.909 | 0.901 | 0.941 | 0.858 | 0.879 | 0.807 | 0.899 | 0.831 | 0.048 | 50 |
| M0891 | 256 | exp14 | 1024bit-ECFP6 | RF | 0.880 | 0.876 | 0.929 | 0.802 | 0.837 | 0.745 | 0.866 | 0.855 | 0.018 | 50 |
| M0892 | 256 | exp15 | 1024bit-ECFP6 | RF | 0.873 | 0.918 | 0.959 | 0.736 | 0.817 | 0.732 | 0.847 | 0.832 | 0.015 | 100 |
| M0893 | 256 | exp16 | 1024bit-ECFP6 | RF | 0.877 | 0.909 | 0.953 | 0.755 | 0.825 | 0.739 | 0.854 | 0.851 | 0.026 | 900 |
| M0894 | 256 | exp17 | 1024bit-ECFP6 | RF | 0.888 | 0.912 | 0.953 | 0.783 | 0.843 | 0.761 | 0.868 | 0.840 | 0.003 | 600 |
| M0895 | 256 | exp18 | 1024bit-ECFP6 | RF | 0.888 | 0.941 | 0.971 | 0.755 | 0.838 | 0.764 | 0.863 | 0.843 | 0.005 | 500 |
| M0896 | 256 | exp19 | 1024bit-ECFP6 | RF | 0.913 | 0.936 | 0.965 | 0.830 | 0.880 | 0.816 | 0.897 | 0.823 | 0.057 | 700 |
| M0897 | 256 | exp01 | 1024bit-ECFP6 | DNN2 | 0.855 | 0.875 | 0.935 | 0.726 | 0.794 | 0.691 | 0.831 | 0.846 | 0.052 | 300 60 0.2 |
| M0898 | 256 | exp02 | 1024bit-ECFP6 | DNN2 | 0.877 | 0.860 | 0.918 | 0.811 | 0.835 | 0.738 | 0.865 | 0.825 | 0.010 | 500 160 0.5 |
| M0899 | 256 | exp03 | 1024bit-ECFP6 | DNN2 | 0.891 | 0.880 | 0.929 | 0.830 | 0.854 | 0.769 | 0.879 | 0.842 | 0.012 | 500 80 0.5 |
| M0900 | 256 | exp01 | 1024bit-ECFP6 | DNN3 | 0.855 | 0.884 | 0.941 | 0.717 | 0.792 | 0.691 | 0.829 | 0.845 | 0.053 | 500 120 0.1 |
| M0901 | 256 | exp02 | 1024bit-ECFP6 | DNN3 | 0.877 | 0.883 | 0.935 | 0.783 | 0.830 | 0.737 | 0.859 | 0.820 | 0.010 | 100 160 0.2 |
| M0902 | 256 | exp03 | 1024bit-ECFP6 | DNN3 | 0.895 | 0.897 | 0.941 | 0.821 | 0.857 | 0.776 | 0.881 | 0.836 | 0.021 | 200 100 0.4 |
| M0903 | 256 | exp01 | 2048bit-ECFP6 | KNN | 0.855 | 0.830 | 0.900 | 0.783 | 0.806 | 0.691 | 0.842 | 0.848 | 0.042 | 1 uniform |
| M0904 | 256 | exp02 | 2048bit-ECFP6 | KNN | 0.880 | 0.869 | 0.924 | 0.811 | 0.839 | 0.745 | 0.868 | 0.850 | 0.011 | 3 uniform |
| M0905 | 256 | exp03 | 2048bit-ECFP6 | KNN | 0.884 | 0.856 | 0.912 | 0.840 | 0.848 | 0.754 | 0.876 | 0.835 | 0.013 | 3 distance |
| M0906 | 256 | exp04 | 2048bit-ECFP6 | KNN | 0.891 | 0.858 | 0.912 | 0.858 | 0.858 | 0.770 | 0.885 | 0.833 | 0.025 | 5 distance |
| M0907 | 256 | exp05 | 2048bit-ECFP6 | KNN | 0.851 | 0.804 | 0.876 | 0.811 | 0.808 | 0.687 | 0.844 | 0.845 | 0.037 | 5 uniform |
| M0908 | 256 | exp06 | 2048bit-ECFP6 | KNN | 0.884 | 0.878 | 0.929 | 0.811 | 0.843 | 0.753 | 0.870 | 0.836 | 0.007 | 3 uniform |
| M0909 | 256 | exp07 | 2048bit-ECFP6 | KNN | 0.888 | 0.912 | 0.953 | 0.783 | 0.843 | 0.761 | 0.868 | 0.843 | 0.000 | 3 distance |
| M0910 | 256 | exp08 | 2048bit-ECFP6 | KNN | 0.895 | 0.874 | 0.924 | 0.849 | 0.861 | 0.777 | 0.887 | 0.843 | 0.018 | 3 distance |
| M0911 | 256 | exp09 | 2048bit-ECFP6 | KNN | 0.873 | 0.882 | 0.935 | 0.774 | 0.824 | 0.729 | 0.855 | 0.828 | 0.004 | 9 distance |
| M0912 | 256 | exp10 | 2048bit-ECFP6 | KNN | 0.902 | 0.916 | 0.953 | 0.821 | 0.866 | 0.792 | 0.887 | 0.826 | 0.040 | 5 uniform |
| M0913 | 256 | exp11 | 2048bit-ECFP6 | KNN | 0.902 | 0.891 | 0.935 | 0.849 | 0.870 | 0.792 | 0.892 | 0.847 | 0.023 | 3 distance |
| M0914 | 256 | exp12 | 2048bit-ECFP6 | KNN | 0.899 | 0.924 | 0.959 | 0.802 | 0.859 | 0.785 | 0.881 | 0.831 | 0.028 | 7 distance |
| M0915 | 256 | exp13 | 2048bit-ECFP6 | KNN | 0.848 | 0.796 | 0.871 | 0.811 | 0.804 | 0.680 | 0.841 | 0.842 | 0.038 | 3 uniform |
| M0916 | 256 | exp14 | 2048bit-ECFP6 | KNN | 0.866 | 0.795 | 0.859 | 0.877 | 0.834 | 0.725 | 0.868 | 0.848 | 0.014 | 1 uniform |
| M0917 | 256 | exp15 | 2048bit-ECFP6 | KNN | 0.891 | 0.922 | 0.959 | 0.783 | 0.847 | 0.770 | 0.871 | 0.827 | 0.020 | 7 distance |
| M0918 | 256 | exp16 | 2048bit-ECFP6 | KNN | 0.855 | 0.824 | 0.894 | 0.792 | 0.808 | 0.692 | 0.843 | 0.843 | 0.035 | 3 uniform |
| M0919 | 256 | exp17 | 2048bit-ECFP6 | KNN | 0.873 | 0.838 | 0.900 | 0.830 | 0.834 | 0.732 | 0.865 | 0.841 | 0.007 | 3 distance |
| M0920 | 256 | exp18 | 2048bit-ECFP6 | KNN | 0.866 | 0.856 | 0.918 | 0.783 | 0.818 | 0.714 | 0.851 | 0.851 | 0.033 | 3 uniform |
| M0921 | 256 | exp19 | 2048bit-ECFP6 | KNN | 0.888 | 0.871 | 0.924 | 0.830 | 0.850 | 0.761 | 0.877 | 0.832 | 0.018 | 7 distance |
| M0922 | 256 | exp01 | 2048bit-ECFP6 | SVM | 0.877 | 0.900 | 0.947 | 0.764 | 0.827 | 0.738 | 0.855 | 0.866 | 0.039 | 5 0.1 |
| M0923 | 256 | exp02 | 2048bit-ECFP6 | SVM | 0.891 | 0.873 | 0.924 | 0.840 | 0.856 | 0.769 | 0.882 | 0.857 | 0.001 | 5 0.05 |
| M0924 | 256 | exp03 | 2048bit-ECFP6 | SVM | 0.884 | 0.870 | 0.924 | 0.821 | 0.845 | 0.753 | 0.873 | 0.856 | 0.011 | 5 0.05 |
| M0925 | 256 | exp04 | 2048bit-ECFP6 | SVM | 0.891 | 0.865 | 0.918 | 0.849 | 0.857 | 0.770 | 0.883 | 0.866 | 0.009 | 5 0.1 |
| M0926 | 256 | exp05 | 2048bit-ECFP6 | SVM | 0.873 | 0.838 | 0.900 | 0.830 | 0.834 | 0.732 | 0.865 | 0.877 | 0.043 | 100000.0 0.1 |
| M0927 | 256 | exp06 | 2048bit-ECFP6 | SVM | 0.888 | 0.895 | 0.941 | 0.802 | 0.846 | 0.761 | 0.871 | 0.869 | 0.023 | 50000.0 0.1 |
| M0928 | 256 | exp07 | 2048bit-ECFP6 | SVM | 0.888 | 0.887 | 0.935 | 0.811 | 0.847 | 0.761 | 0.873 | 0.860 | 0.013 | 10 0.1 |
| M0929 | 256 | exp08 | 2048bit-ECFP6 | SVM | 0.891 | 0.873 | 0.924 | 0.840 | 0.856 | 0.769 | 0.882 | 0.871 | 0.015 | 5 0.1 |
| M0930 | 256 | exp09 | 2048bit-ECFP6 | SVM | 0.895 | 0.889 | 0.935 | 0.830 | 0.859 | 0.776 | 0.883 | 0.850 | 0.009 | 100 0.05 |
| M0931 | 256 | exp10 | 2048bit-ECFP6 | SVM | 0.913 | 0.936 | 0.965 | 0.830 | 0.880 | 0.816 | 0.897 | 0.859 | 0.021 | 5 0.1 |
| M0932 | 256 | exp11 | 2048bit-ECFP6 | SVM | 0.870 | 0.857 | 0.918 | 0.792 | 0.824 | 0.722 | 0.855 | 0.870 | 0.046 | 5 0.05 |
| M0933 | 256 | exp12 | 2048bit-ECFP6 | SVM | 0.920 | 0.920 | 0.953 | 0.868 | 0.893 | 0.831 | 0.910 | 0.848 | 0.045 | 5 0.1 |
| M0934 | 256 | exp13 | 2048bit-ECFP6 | SVM | 0.895 | 0.853 | 0.906 | 0.877 | 0.865 | 0.779 | 0.891 | 0.848 | 0.017 | 100 0.1 |
| M0935 | 256 | exp14 | 2048bit-ECFP6 | SVM | 0.888 | 0.844 | 0.900 | 0.868 | 0.856 | 0.764 | 0.884 | 0.878 | 0.022 | 100 0.1 |
| M0936 | 256 | exp15 | 2048bit-ECFP6 | SVM | 0.891 | 0.922 | 0.959 | 0.783 | 0.847 | 0.770 | 0.871 | 0.852 | 0.005 | 5 0.1 |
| M0937 | 256 | exp16 | 2048bit-ECFP6 | SVM | 0.884 | 0.870 | 0.924 | 0.821 | 0.845 | 0.753 | 0.873 | 0.858 | 0.013 | 10000.0 0.1 |
| M0938 | 256 | exp17 | 2048bit-ECFP6 | SVM | 0.888 | 0.857 | 0.912 | 0.849 | 0.853 | 0.762 | 0.881 | 0.861 | 0.008 | 10 0.1 |
| M0939 | 256 | exp18 | 2048bit-ECFP6 | SVM | 0.877 | 0.846 | 0.906 | 0.830 | 0.838 | 0.739 | 0.868 | 0.866 | 0.028 | 1 0.1 |
| M0940 | 256 | exp19 | 2048bit-ECFP6 | SVM | 0.917 | 0.911 | 0.947 | 0.868 | 0.889 | 0.823 | 0.907 | 0.844 | 0.045 | 1 0.1 |
| M0941 | 256 | exp01 | 2048bit-ECFP6 | GBM | 0.848 | 0.890 | 0.947 | 0.689 | 0.777 | 0.677 | 0.818 | 0.846 | 0.069 | 700 0.1 |
| M0942 | 256 | exp02 | 2048bit-ECFP6 | GBM | 0.884 | 0.902 | 0.947 | 0.783 | 0.838 | 0.753 | 0.865 | 0.830 | 0.008 | 100 0.6 |
| M0943 | 256 | exp03 | 2048bit-ECFP6 | GBM | 0.859 | 0.838 | 0.906 | 0.783 | 0.810 | 0.699 | 0.845 | 0.845 | 0.035 | 300 0.9 |
| M0944 | 256 | exp04 | 2048bit-ECFP6 | GBM | 0.884 | 0.885 | 0.935 | 0.802 | 0.842 | 0.753 | 0.869 | 0.842 | 0.000 | 300 0.3 |
| M0945 | 256 | exp05 | 2048bit-ECFP6 | GBM | 0.873 | 0.838 | 0.900 | 0.830 | 0.834 | 0.732 | 0.865 | 0.855 | 0.021 | 200 0.9 |
| M0946 | 256 | exp06 | 2048bit-ECFP6 | GBM | 0.884 | 0.878 | 0.929 | 0.811 | 0.843 | 0.753 | 0.870 | 0.836 | 0.007 | 100 0.9 |
| M0947 | 256 | exp07 | 2048bit-ECFP6 | GBM | 0.877 | 0.891 | 0.941 | 0.774 | 0.828 | 0.737 | 0.857 | 0.832 | 0.004 | 200 0.1 |
| M0948 | 256 | exp08 | 2048bit-ECFP6 | GBM | 0.877 | 0.909 | 0.953 | 0.755 | 0.825 | 0.739 | 0.854 | 0.850 | 0.025 | 300 0.1 |
| M0949 | 256 | exp09 | 2048bit-ECFP6 | GBM | 0.873 | 0.851 | 0.912 | 0.811 | 0.831 | 0.730 | 0.862 | 0.829 | 0.002 | 500 0.4 |
| M0950 | 256 | exp10 | 2048bit-ECFP6 | GBM | 0.888 | 0.887 | 0.935 | 0.811 | 0.847 | 0.761 | 0.873 | 0.833 | 0.014 | 800 0.6 |
| M0951 | 256 | exp11 | 2048bit-ECFP6 | GBM | 0.862 | 0.870 | 0.929 | 0.755 | 0.808 | 0.706 | 0.842 | 0.857 | 0.049 | 300 0.7 |
| M0952 | 256 | exp12 | 2048bit-ECFP6 | GBM | 0.917 | 0.919 | 0.953 | 0.858 | 0.888 | 0.823 | 0.905 | 0.831 | 0.057 | 400 0.4 |
| M0953 | 256 | exp13 | 2048bit-ECFP6 | GBM | 0.884 | 0.863 | 0.918 | 0.830 | 0.846 | 0.754 | 0.874 | 0.843 | 0.003 | 400 0.4 |
| M0954 | 256 | exp14 | 2048bit-ECFP6 | GBM | 0.873 | 0.866 | 0.924 | 0.792 | 0.828 | 0.729 | 0.858 | 0.849 | 0.021 | 100 0.3 |
| M0955 | 256 | exp15 | 2048bit-ECFP6 | GBM | 0.880 | 0.901 | 0.947 | 0.774 | 0.832 | 0.746 | 0.861 | 0.836 | 0.004 | 50 0.6 |
| M0956 | 256 | exp16 | 2048bit-ECFP6 | GBM | 0.866 | 0.879 | 0.935 | 0.755 | 0.812 | 0.714 | 0.845 | 0.842 | 0.030 | 500 0.8 |
| M0957 | 256 | exp17 | 2048bit-ECFP6 | GBM | 0.870 | 0.850 | 0.912 | 0.802 | 0.825 | 0.722 | 0.857 | 0.838 | 0.013 | 700 0.9 |
| M0958 | 256 | exp18 | 2048bit-ECFP6 | GBM | 0.877 | 0.846 | 0.906 | 0.830 | 0.838 | 0.739 | 0.868 | 0.844 | 0.006 | 800 0.6 |
| M0959 | 256 | exp19 | 2048bit-ECFP6 | GBM | 0.909 | 0.926 | 0.959 | 0.830 | 0.876 | 0.808 | 0.894 | 0.837 | 0.039 | 100 0.2 |
| M0960 | 256 | exp01 | 2048bit-ECFP6 | RF | 0.855 | 0.893 | 0.947 | 0.708 | 0.789 | 0.692 | 0.827 | 0.856 | 0.067 | 50 |
| M0961 | 256 | exp02 | 2048bit-ECFP6 | RF | 0.899 | 0.953 | 0.976 | 0.774 | 0.854 | 0.788 | 0.875 | 0.841 | 0.013 | 200 |
| M0962 | 256 | exp03 | 2048bit-ECFP6 | RF | 0.873 | 0.859 | 0.918 | 0.802 | 0.829 | 0.730 | 0.860 | 0.849 | 0.020 | 10 |
| M0963 | 256 | exp04 | 2048bit-ECFP6 | RF | 0.895 | 0.905 | 0.947 | 0.811 | 0.856 | 0.776 | 0.879 | 0.845 | 0.011 | 300 |
| M0964 | 256 | exp05 | 2048bit-ECFP6 | RF | 0.859 | 0.832 | 0.900 | 0.792 | 0.812 | 0.699 | 0.846 | 0.854 | 0.041 | 50 |
| M0965 | 256 | exp06 | 2048bit-ECFP6 | RF | 0.895 | 0.943 | 0.971 | 0.774 | 0.850 | 0.779 | 0.873 | 0.842 | 0.008 | 400 |
| M0966 | 256 | exp07 | 2048bit-ECFP6 | RF | 0.895 | 0.943 | 0.971 | 0.774 | 0.850 | 0.779 | 0.873 | 0.844 | 0.006 | 600 |
| M0967 | 256 | exp08 | 2048bit-ECFP6 | RF | 0.888 | 0.895 | 0.941 | 0.802 | 0.846 | 0.761 | 0.871 | 0.856 | 0.010 | 500 |
| M0968 | 256 | exp09 | 2048bit-ECFP6 | RF | 0.891 | 0.913 | 0.953 | 0.792 | 0.848 | 0.769 | 0.873 | 0.838 | 0.010 | 50 |
| M0969 | 256 | exp10 | 2048bit-ECFP6 | RF | 0.895 | 0.923 | 0.959 | 0.792 | 0.853 | 0.777 | 0.875 | 0.841 | 0.012 | 1000 |
| M0970 | 256 | exp11 | 2048bit-ECFP6 | RF | 0.870 | 0.907 | 0.953 | 0.736 | 0.812 | 0.723 | 0.845 | 0.855 | 0.043 | 50 |
| M0971 | 256 | exp12 | 2048bit-ECFP6 | RF | 0.924 | 0.967 | 0.982 | 0.830 | 0.893 | 0.841 | 0.906 | 0.836 | 0.057 | 200 |
| M0972 | 256 | exp13 | 2048bit-ECFP6 | RF | 0.899 | 0.898 | 0.941 | 0.830 | 0.863 | 0.784 | 0.885 | 0.840 | 0.023 | 50 |
| M0973 | 256 | exp14 | 2048bit-ECFP6 | RF | 0.873 | 0.866 | 0.924 | 0.792 | 0.828 | 0.729 | 0.858 | 0.862 | 0.034 | 100 |
| M0974 | 256 | exp15 | 2048bit-ECFP6 | RF | 0.899 | 0.943 | 0.971 | 0.783 | 0.856 | 0.787 | 0.877 | 0.832 | 0.024 | 800 |
| M0975 | 256 | exp16 | 2048bit-ECFP6 | RF | 0.873 | 0.918 | 0.959 | 0.736 | 0.817 | 0.732 | 0.847 | 0.852 | 0.035 | 50 |
| M0976 | 256 | exp17 | 2048bit-ECFP6 | RF | 0.891 | 0.913 | 0.953 | 0.792 | 0.848 | 0.769 | 0.873 | 0.845 | 0.003 | 700 |
| M0977 | 256 | exp18 | 2048bit-ECFP6 | RF | 0.895 | 0.923 | 0.959 | 0.792 | 0.853 | 0.777 | 0.875 | 0.850 | 0.003 | 200 |
| M0978 | 256 | exp19 | 2048bit-ECFP6 | RF | 0.902 | 0.925 | 0.959 | 0.811 | 0.864 | 0.792 | 0.885 | 0.838 | 0.026 | 600 |
| M0979 | 256 | exp01 | 2048bit-ECFP6 | DNN2 | 0.891 | 0.932 | 0.965 | 0.774 | 0.845 | 0.771 | 0.869 | 0.854 | 0.009 | 300 140 0.1 |
| M0980 | 256 | exp02 | 2048bit-ECFP6 | DNN2 | 0.880 | 0.869 | 0.924 | 0.811 | 0.839 | 0.745 | 0.868 | 0.850 | 0.011 | 500 120 0.5 |
| M0981 | 256 | exp03 | 2048bit-ECFP6 | DNN2 | 0.873 | 0.851 | 0.912 | 0.811 | 0.831 | 0.730 | 0.862 | 0.852 | 0.021 | 100 80 0.2 |
| M0982 | 256 | exp01 | 2048bit-ECFP6 | DNN3 | 0.877 | 0.909 | 0.953 | 0.755 | 0.825 | 0.739 | 0.854 | 0.858 | 0.033 | 500 140 0.4 |
| M0983 | 256 | exp02 | 2048bit-ECFP6 | DNN3 | 0.902 | 0.899 | 0.941 | 0.840 | 0.868 | 0.792 | 0.890 | 0.850 | 0.018 | 100 80 0.5 |
| M0984 | 256 | exp03 | 2048bit-ECFP6 | DNN3 | 0.895 | 0.897 | 0.941 | 0.821 | 0.857 | 0.776 | 0.881 | 0.853 | 0.004 | 300 160 0.3 |
| M0985 | 128 | exp01 | 1024bit-ECFP4 | KNN | 0.866 | 0.879 | 0.935 | 0.755 | 0.812 | 0.714 | 0.845 | 0.836 | 0.023 | 5 distance |
| M0986 | 128 | exp02 | 1024bit-ECFP4 | KNN | 0.880 | 0.876 | 0.929 | 0.802 | 0.837 | 0.745 | 0.866 | 0.828 | 0.009 | 7 distance |
| M0987 | 128 | exp03 | 1024bit-ECFP4 | KNN | 0.855 | 0.780 | 0.847 | 0.868 | 0.821 | 0.703 | 0.857 | 0.843 | 0.022 | 3 distance |
| M0988 | 128 | exp04 | 1024bit-ECFP4 | KNN | 0.870 | 0.792 | 0.853 | 0.896 | 0.841 | 0.735 | 0.875 | 0.832 | 0.009 | 5 distance |
| M0989 | 128 | exp05 | 1024bit-ECFP4 | KNN | 0.844 | 0.769 | 0.841 | 0.849 | 0.807 | 0.679 | 0.845 | 0.845 | 0.038 | 3 distance |
| M0990 | 128 | exp06 | 1024bit-ECFP4 | KNN | 0.884 | 0.849 | 0.906 | 0.849 | 0.849 | 0.755 | 0.877 | 0.847 | 0.002 | 5 distance |
| M0991 | 128 | exp07 | 1024bit-ECFP4 | KNN | 0.895 | 0.897 | 0.941 | 0.821 | 0.857 | 0.776 | 0.881 | 0.830 | 0.027 | 5 uniform |
| M0992 | 128 | exp08 | 1024bit-ECFP4 | KNN | 0.880 | 0.848 | 0.906 | 0.840 | 0.844 | 0.747 | 0.873 | 0.841 | 0.003 | 5 distance |
| M0993 | 128 | exp09 | 1024bit-ECFP4 | KNN | 0.870 | 0.802 | 0.865 | 0.877 | 0.838 | 0.731 | 0.871 | 0.844 | 0.006 | 5 distance |
| M0994 | 128 | exp10 | 1024bit-ECFP4 | KNN | 0.891 | 0.896 | 0.941 | 0.811 | 0.851 | 0.768 | 0.876 | 0.829 | 0.022 | 7 uniform |
| M0995 | 128 | exp11 | 1024bit-ECFP4 | KNN | 0.870 | 0.830 | 0.894 | 0.830 | 0.830 | 0.724 | 0.862 | 0.847 | 0.017 | 3 distance |
| M0996 | 128 | exp12 | 1024bit-ECFP4 | KNN | 0.884 | 0.843 | 0.900 | 0.858 | 0.850 | 0.756 | 0.879 | 0.832 | 0.018 | 3 distance |
| M0997 | 128 | exp13 | 1024bit-ECFP4 | KNN | 0.895 | 0.841 | 0.894 | 0.896 | 0.868 | 0.782 | 0.895 | 0.818 | 0.050 | 5 distance |
| M0998 | 128 | exp14 | 1024bit-ECFP4 | KNN | 0.851 | 0.783 | 0.853 | 0.849 | 0.814 | 0.693 | 0.851 | 0.850 | 0.036 | 5 distance |
| M0999 | 128 | exp15 | 1024bit-ECFP4 | KNN | 0.884 | 0.863 | 0.918 | 0.830 | 0.846 | 0.754 | 0.874 | 0.825 | 0.021 | 3 uniform |
| M1000 | 128 | exp16 | 1024bit-ECFP4 | KNN | 0.873 | 0.866 | 0.924 | 0.792 | 0.828 | 0.729 | 0.858 | 0.839 | 0.011 | 5 uniform |
| M1001 | 128 | exp17 | 1024bit-ECFP4 | KNN | 0.851 | 0.810 | 0.882 | 0.802 | 0.806 | 0.685 | 0.842 | 0.837 | 0.030 | 7 distance |
| M1002 | 128 | exp18 | 1024bit-ECFP4 | KNN | 0.841 | 0.798 | 0.876 | 0.783 | 0.790 | 0.662 | 0.830 | 0.840 | 0.050 | 7 distance |
| M1003 | 128 | exp19 | 1024bit-ECFP4 | KNN | 0.877 | 0.853 | 0.912 | 0.821 | 0.837 | 0.738 | 0.867 | 0.822 | 0.015 | 7 distance |
| M1004 | 128 | exp01 | 1024bit-ECFP4 | SVM | 0.870 | 0.880 | 0.935 | 0.764 | 0.818 | 0.722 | 0.850 | 0.851 | 0.033 | 1 0.1 |
| M1005 | 128 | exp02 | 1024bit-ECFP4 | SVM | 0.902 | 0.934 | 0.965 | 0.802 | 0.863 | 0.793 | 0.883 | 0.849 | 0.014 | 1 0.1 |
| M1006 | 128 | exp03 | 1024bit-ECFP4 | SVM | 0.880 | 0.869 | 0.924 | 0.811 | 0.839 | 0.745 | 0.868 | 0.852 | 0.013 | 10 0.1 |
| M1007 | 128 | exp04 | 1024bit-ECFP4 | SVM | 0.877 | 0.827 | 0.888 | 0.858 | 0.843 | 0.742 | 0.873 | 0.854 | 0.011 | 10 0.1 |
| M1008 | 128 | exp05 | 1024bit-ECFP4 | SVM | 0.859 | 0.813 | 0.882 | 0.821 | 0.817 | 0.702 | 0.851 | 0.849 | 0.032 | 1000.0 0.1 |
| M1009 | 128 | exp06 | 1024bit-ECFP4 | SVM | 0.906 | 0.917 | 0.953 | 0.830 | 0.871 | 0.800 | 0.891 | 0.854 | 0.017 | 5 0.1 |
| M1010 | 128 | exp07 | 1024bit-ECFP4 | SVM | 0.895 | 0.897 | 0.941 | 0.821 | 0.857 | 0.776 | 0.881 | 0.845 | 0.012 | 1 0.1 |
| M1011 | 128 | exp08 | 1024bit-ECFP4 | SVM | 0.891 | 0.852 | 0.906 | 0.868 | 0.860 | 0.771 | 0.887 | 0.856 | 0.004 | 100 0.1 |
| M1012 | 128 | exp09 | 1024bit-ECFP4 | SVM | 0.902 | 0.876 | 0.924 | 0.868 | 0.872 | 0.793 | 0.896 | 0.856 | 0.016 | 5 0.1 |
| M1013 | 128 | exp10 | 1024bit-ECFP4 | SVM | 0.888 | 0.871 | 0.924 | 0.830 | 0.850 | 0.761 | 0.877 | 0.844 | 0.006 | 50000.0 0.1 |
| M1014 | 128 | exp11 | 1024bit-ECFP4 | SVM | 0.884 | 0.878 | 0.929 | 0.811 | 0.843 | 0.753 | 0.870 | 0.865 | 0.022 | 5 0.1 |
| M1015 | 128 | exp12 | 1024bit-ECFP4 | SVM | 0.902 | 0.876 | 0.924 | 0.868 | 0.872 | 0.793 | 0.896 | 0.845 | 0.027 | 5 0.1 |
| M1016 | 128 | exp13 | 1024bit-ECFP4 | SVM | 0.891 | 0.852 | 0.906 | 0.868 | 0.860 | 0.771 | 0.887 | 0.843 | 0.017 | 5 0.05 |
| M1017 | 128 | exp14 | 1024bit-ECFP4 | SVM | 0.884 | 0.849 | 0.906 | 0.849 | 0.849 | 0.755 | 0.877 | 0.874 | 0.025 | 5 0.1 |
| M1018 | 128 | exp15 | 1024bit-ECFP4 | SVM | 0.877 | 0.883 | 0.935 | 0.783 | 0.830 | 0.737 | 0.859 | 0.848 | 0.018 | 5 0.1 |
| M1019 | 128 | exp16 | 1024bit-ECFP4 | SVM | 0.866 | 0.856 | 0.918 | 0.783 | 0.818 | 0.714 | 0.851 | 0.869 | 0.051 | 5 0.1 |
| M1020 | 128 | exp17 | 1024bit-ECFP4 | SVM | 0.899 | 0.868 | 0.918 | 0.868 | 0.868 | 0.786 | 0.893 | 0.845 | 0.023 | 5 0.1 |
| M1021 | 128 | exp18 | 1024bit-ECFP4 | SVM | 0.873 | 0.838 | 0.900 | 0.830 | 0.834 | 0.732 | 0.865 | 0.855 | 0.021 | 10 0.1 |
| M1022 | 128 | exp19 | 1024bit-ECFP4 | SVM | 0.909 | 0.901 | 0.941 | 0.858 | 0.879 | 0.807 | 0.899 | 0.848 | 0.031 | 5 0.05 |
| M1023 | 128 | exp01 | 1024bit-ECFP4 | GBM | 0.870 | 0.872 | 0.929 | 0.774 | 0.820 | 0.721 | 0.852 | 0.849 | 0.029 | 100 0.5 |
| M1024 | 128 | exp02 | 1024bit-ECFP4 | GBM | 0.895 | 0.889 | 0.935 | 0.830 | 0.859 | 0.776 | 0.883 | 0.843 | 0.016 | 200 0.2 |
| M1025 | 128 | exp03 | 1024bit-ECFP4 | GBM | 0.880 | 0.841 | 0.900 | 0.849 | 0.845 | 0.748 | 0.875 | 0.847 | 0.002 | 1000 0.8 |
| M1026 | 128 | exp04 | 1024bit-ECFP4 | GBM | 0.895 | 0.867 | 0.918 | 0.858 | 0.863 | 0.778 | 0.888 | 0.840 | 0.023 | 300 0.2 |
| M1027 | 128 | exp05 | 1024bit-ECFP4 | GBM | 0.859 | 0.825 | 0.894 | 0.802 | 0.813 | 0.700 | 0.848 | 0.840 | 0.027 | 300 0.2 |
| M1028 | 128 | exp06 | 1024bit-ECFP4 | GBM | 0.895 | 0.923 | 0.959 | 0.792 | 0.853 | 0.777 | 0.875 | 0.840 | 0.013 | 50 0.7 |
| M1029 | 128 | exp07 | 1024bit-ECFP4 | GBM | 0.899 | 0.915 | 0.953 | 0.811 | 0.860 | 0.784 | 0.882 | 0.831 | 0.029 | 1000 0.1 |
| M1030 | 128 | exp08 | 1024bit-ECFP4 | GBM | 0.884 | 0.843 | 0.900 | 0.858 | 0.850 | 0.756 | 0.879 | 0.845 | 0.005 | 800 0.1 |
| M1031 | 128 | exp09 | 1024bit-ECFP4 | GBM | 0.920 | 0.920 | 0.953 | 0.868 | 0.893 | 0.831 | 0.910 | 0.845 | 0.048 | 300 0.1 |
| M1032 | 128 | exp10 | 1024bit-ECFP4 | GBM | 0.866 | 0.856 | 0.918 | 0.783 | 0.818 | 0.714 | 0.851 | 0.845 | 0.027 | 50 0.5 |
| M1033 | 128 | exp11 | 1024bit-ECFP4 | GBM | 0.884 | 0.870 | 0.924 | 0.821 | 0.845 | 0.753 | 0.873 | 0.858 | 0.013 | 800 0.2 |
| M1034 | 128 | exp12 | 1024bit-ECFP4 | GBM | 0.920 | 0.912 | 0.947 | 0.877 | 0.894 | 0.831 | 0.912 | 0.833 | 0.061 | 500 0.1 |
| M1035 | 128 | exp13 | 1024bit-ECFP4 | GBM | 0.873 | 0.814 | 0.876 | 0.868 | 0.840 | 0.736 | 0.872 | 0.848 | 0.008 | 800 0.6 |
| M1036 | 128 | exp14 | 1024bit-ECFP4 | GBM | 0.859 | 0.819 | 0.888 | 0.811 | 0.815 | 0.701 | 0.850 | 0.852 | 0.037 | 100 0.4 |
| M1037 | 128 | exp15 | 1024bit-ECFP4 | GBM | 0.859 | 0.853 | 0.918 | 0.764 | 0.806 | 0.698 | 0.841 | 0.844 | 0.038 | 300 0.5 |
| M1038 | 128 | exp16 | 1024bit-ECFP4 | GBM | 0.862 | 0.878 | 0.935 | 0.745 | 0.806 | 0.706 | 0.840 | 0.842 | 0.036 | 100 0.5 |
| M1039 | 128 | exp17 | 1024bit-ECFP4 | GBM | 0.899 | 0.898 | 0.941 | 0.830 | 0.863 | 0.784 | 0.885 | 0.835 | 0.028 | 50 0.8 |
| M1040 | 128 | exp18 | 1024bit-ECFP4 | GBM | 0.873 | 0.826 | 0.888 | 0.849 | 0.837 | 0.734 | 0.869 | 0.855 | 0.018 | 900 0.2 |
| M1041 | 128 | exp19 | 1024bit-ECFP4 | GBM | 0.899 | 0.898 | 0.941 | 0.830 | 0.863 | 0.784 | 0.885 | 0.823 | 0.040 | 100 0.2 |
| M1042 | 128 | exp01 | 1024bit-ECFP4 | RF | 0.870 | 0.907 | 0.953 | 0.736 | 0.812 | 0.723 | 0.845 | 0.848 | 0.036 | 700 |
| M1043 | 128 | exp02 | 1024bit-ECFP4 | RF | 0.891 | 0.913 | 0.953 | 0.792 | 0.848 | 0.769 | 0.873 | 0.838 | 0.010 | 100 |
| M1044 | 128 | exp03 | 1024bit-ECFP4 | RF | 0.891 | 0.904 | 0.947 | 0.802 | 0.850 | 0.769 | 0.875 | 0.844 | 0.006 | 1000 |
| M1045 | 128 | exp04 | 1024bit-ECFP4 | RF | 0.877 | 0.840 | 0.900 | 0.840 | 0.840 | 0.740 | 0.870 | 0.843 | 0.003 | 100 |
| M1046 | 128 | exp05 | 1024bit-ECFP4 | RF | 0.870 | 0.843 | 0.906 | 0.811 | 0.827 | 0.723 | 0.859 | 0.858 | 0.031 | 50 |
| M1047 | 128 | exp06 | 1024bit-ECFP4 | RF | 0.888 | 0.903 | 0.947 | 0.792 | 0.844 | 0.761 | 0.869 | 0.841 | 0.003 | 200 |
| M1048 | 128 | exp07 | 1024bit-ECFP4 | RF | 0.902 | 0.934 | 0.965 | 0.802 | 0.863 | 0.793 | 0.883 | 0.834 | 0.029 | 400 |
| M1049 | 128 | exp08 | 1024bit-ECFP4 | RF | 0.899 | 0.890 | 0.935 | 0.840 | 0.864 | 0.784 | 0.887 | 0.844 | 0.020 | 600 |
| M1050 | 128 | exp09 | 1024bit-ECFP4 | RF | 0.902 | 0.907 | 0.947 | 0.830 | 0.867 | 0.792 | 0.888 | 0.845 | 0.022 | 100 |
| M1051 | 128 | exp10 | 1024bit-ECFP4 | RF | 0.906 | 0.926 | 0.959 | 0.821 | 0.870 | 0.800 | 0.890 | 0.835 | 0.035 | 500 |
| M1052 | 128 | exp11 | 1024bit-ECFP4 | RF | 0.884 | 0.911 | 0.953 | 0.774 | 0.837 | 0.754 | 0.863 | 0.853 | 0.016 | 100 |
| M1053 | 128 | exp12 | 1024bit-ECFP4 | RF | 0.902 | 0.899 | 0.941 | 0.840 | 0.868 | 0.792 | 0.890 | 0.831 | 0.037 | 50 |
| M1054 | 128 | exp13 | 1024bit-ECFP4 | RF | 0.884 | 0.856 | 0.912 | 0.840 | 0.848 | 0.754 | 0.876 | 0.837 | 0.011 | 100 |
| M1055 | 128 | exp14 | 1024bit-ECFP4 | RF | 0.873 | 0.859 | 0.918 | 0.802 | 0.829 | 0.730 | 0.860 | 0.847 | 0.018 | 200 |
| M1056 | 128 | exp15 | 1024bit-ECFP4 | RF | 0.870 | 0.880 | 0.935 | 0.764 | 0.818 | 0.722 | 0.850 | 0.834 | 0.016 | 700 |
| M1057 | 128 | exp16 | 1024bit-ECFP4 | RF | 0.851 | 0.857 | 0.924 | 0.736 | 0.792 | 0.682 | 0.830 | 0.851 | 0.058 | 300 |
| M1058 | 128 | exp17 | 1024bit-ECFP4 | RF | 0.902 | 0.916 | 0.953 | 0.821 | 0.866 | 0.792 | 0.887 | 0.837 | 0.029 | 100 |
| M1059 | 128 | exp18 | 1024bit-ECFP4 | RF | 0.884 | 0.894 | 0.941 | 0.792 | 0.840 | 0.753 | 0.867 | 0.850 | 0.010 | 1000 |
| M1060 | 128 | exp19 | 1024bit-ECFP4 | RF | 0.902 | 0.916 | 0.953 | 0.821 | 0.866 | 0.792 | 0.887 | 0.838 | 0.028 | 50 |
| M1061 | 128 | exp01 | 1024bit-ECFP4 | DNN2 | 0.873 | 0.866 | 0.924 | 0.792 | 0.828 | 0.729 | 0.858 | 0.842 | 0.014 | 100 60 0.4 |
| M1062 | 128 | exp02 | 1024bit-ECFP4 | DNN2 | 0.920 | 0.904 | 0.941 | 0.887 | 0.895 | 0.831 | 0.914 | 0.831 | 0.064 | 200 60 0.2 |
| M1063 | 128 | exp03 | 1024bit-ECFP4 | DNN2 | 0.880 | 0.861 | 0.918 | 0.821 | 0.841 | 0.746 | 0.869 | 0.843 | 0.002 | 400 60 0.1 |
| M1064 | 128 | exp01 | 1024bit-ECFP4 | DNN3 | 0.870 | 0.857 | 0.918 | 0.792 | 0.824 | 0.722 | 0.855 | 0.842 | 0.018 | 100 140 0.5 |
| M1065 | 128 | exp02 | 1024bit-ECFP4 | DNN3 | 0.895 | 0.874 | 0.924 | 0.849 | 0.861 | 0.777 | 0.887 | 0.835 | 0.026 | 600 60 0.2 |
| M1066 | 128 | exp03 | 1024bit-ECFP4 | DNN3 | 0.880 | 0.854 | 0.912 | 0.830 | 0.842 | 0.746 | 0.871 | 0.843 | 0.001 | 200 160 0.1 |
| M1067 | 128 | exp01 | 2048bit-ECFP4 | KNN | 0.855 | 0.859 | 0.924 | 0.745 | 0.798 | 0.690 | 0.835 | 0.844 | 0.046 | 7 distance |
| M1068 | 128 | exp02 | 2048bit-ECFP4 | KNN | 0.873 | 0.890 | 0.941 | 0.764 | 0.822 | 0.730 | 0.853 | 0.836 | 0.014 | 7 uniform |
| M1069 | 128 | exp03 | 2048bit-ECFP4 | KNN | 0.888 | 0.864 | 0.918 | 0.840 | 0.852 | 0.762 | 0.879 | 0.848 | 0.004 | 5 distance |
| M1070 | 128 | exp04 | 2048bit-ECFP4 | KNN | 0.862 | 0.779 | 0.841 | 0.896 | 0.833 | 0.722 | 0.869 | 0.834 | 0.001 | 1 uniform |
| M1071 | 128 | exp05 | 2048bit-ECFP4 | KNN | 0.870 | 0.818 | 0.882 | 0.849 | 0.833 | 0.727 | 0.865 | 0.845 | 0.012 | 5 distance |
| M1072 | 128 | exp06 | 2048bit-ECFP4 | KNN | 0.877 | 0.827 | 0.888 | 0.858 | 0.843 | 0.742 | 0.873 | 0.835 | 0.008 | 3 distance |
| M1073 | 128 | exp07 | 2048bit-ECFP4 | KNN | 0.909 | 0.918 | 0.953 | 0.840 | 0.877 | 0.807 | 0.896 | 0.828 | 0.049 | 5 distance |
| M1074 | 128 | exp08 | 2048bit-ECFP4 | KNN | 0.888 | 0.887 | 0.935 | 0.811 | 0.847 | 0.761 | 0.873 | 0.844 | 0.003 | 9 distance |
| M1075 | 128 | exp09 | 2048bit-ECFP4 | KNN | 0.880 | 0.841 | 0.900 | 0.849 | 0.845 | 0.748 | 0.875 | 0.828 | 0.017 | 9 distance |
| M1076 | 128 | exp10 | 2048bit-ECFP4 | KNN | 0.902 | 0.907 | 0.947 | 0.830 | 0.867 | 0.792 | 0.888 | 0.826 | 0.041 | 5 uniform |
| M1077 | 128 | exp11 | 2048bit-ECFP4 | KNN | 0.866 | 0.811 | 0.876 | 0.849 | 0.829 | 0.720 | 0.863 | 0.853 | 0.024 | 1 uniform |
| M1078 | 128 | exp12 | 2048bit-ECFP4 | KNN | 0.909 | 0.886 | 0.929 | 0.877 | 0.882 | 0.808 | 0.903 | 0.824 | 0.058 | 3 distance |
| M1079 | 128 | exp13 | 2048bit-ECFP4 | KNN | 0.877 | 0.810 | 0.871 | 0.887 | 0.847 | 0.746 | 0.879 | 0.834 | 0.013 | 3 distance |
| M1080 | 128 | exp14 | 2048bit-ECFP4 | KNN | 0.870 | 0.807 | 0.871 | 0.868 | 0.836 | 0.730 | 0.869 | 0.847 | 0.011 | 5 distance |
| M1081 | 128 | exp15 | 2048bit-ECFP4 | KNN | 0.866 | 0.879 | 0.935 | 0.755 | 0.812 | 0.714 | 0.845 | 0.828 | 0.016 | 9 distance |
| M1082 | 128 | exp16 | 2048bit-ECFP4 | KNN | 0.855 | 0.844 | 0.912 | 0.764 | 0.802 | 0.690 | 0.838 | 0.843 | 0.041 | 5 distance |
| M1083 | 128 | exp17 | 2048bit-ECFP4 | KNN | 0.870 | 0.830 | 0.894 | 0.830 | 0.830 | 0.724 | 0.862 | 0.835 | 0.005 | 3 distance |
| M1084 | 128 | exp18 | 2048bit-ECFP4 | KNN | 0.873 | 0.838 | 0.900 | 0.830 | 0.834 | 0.732 | 0.865 | 0.843 | 0.009 | 1 uniform |
| M1085 | 128 | exp19 | 2048bit-ECFP4 | KNN | 0.873 | 0.826 | 0.888 | 0.849 | 0.837 | 0.734 | 0.869 | 0.837 | 0.000 | 5 distance |
| M1086 | 128 | exp01 | 2048bit-ECFP4 | SVM | 0.866 | 0.897 | 0.947 | 0.736 | 0.808 | 0.715 | 0.841 | 0.858 | 0.049 | 100 0.005 |
| M1087 | 128 | exp02 | 2048bit-ECFP4 | SVM | 0.895 | 0.914 | 0.953 | 0.802 | 0.854 | 0.777 | 0.877 | 0.853 | 0.001 | 1 0.1 |
| M1088 | 128 | exp03 | 2048bit-ECFP4 | SVM | 0.880 | 0.854 | 0.912 | 0.830 | 0.842 | 0.746 | 0.871 | 0.848 | 0.006 | 10 0.1 |
| M1089 | 128 | exp04 | 2048bit-ECFP4 | SVM | 0.884 | 0.814 | 0.871 | 0.906 | 0.857 | 0.763 | 0.889 | 0.849 | 0.008 | 10 0.1 |
| M1090 | 128 | exp05 | 2048bit-ECFP4 | SVM | 0.870 | 0.818 | 0.882 | 0.849 | 0.833 | 0.727 | 0.865 | 0.857 | 0.024 | 5 0.1 |
| M1091 | 128 | exp06 | 2048bit-ECFP4 | SVM | 0.906 | 0.908 | 0.947 | 0.840 | 0.873 | 0.800 | 0.893 | 0.849 | 0.024 | 5 0.1 |
| M1092 | 128 | exp07 | 2048bit-ECFP4 | SVM | 0.891 | 0.904 | 0.947 | 0.802 | 0.850 | 0.769 | 0.875 | 0.845 | 0.005 | 1 0.1 |
| M1093 | 128 | exp08 | 2048bit-ECFP4 | SVM | 0.884 | 0.836 | 0.894 | 0.868 | 0.852 | 0.757 | 0.881 | 0.849 | 0.003 | 5000.0 0.1 |
| M1094 | 128 | exp09 | 2048bit-ECFP4 | SVM | 0.888 | 0.850 | 0.906 | 0.858 | 0.854 | 0.763 | 0.882 | 0.852 | 0.002 | 5 0.1 |
| M1095 | 128 | exp10 | 2048bit-ECFP4 | SVM | 0.899 | 0.906 | 0.947 | 0.821 | 0.861 | 0.784 | 0.884 | 0.856 | 0.005 | 5 0.1 |
| M1096 | 128 | exp11 | 2048bit-ECFP4 | SVM | 0.884 | 0.878 | 0.929 | 0.811 | 0.843 | 0.753 | 0.870 | 0.858 | 0.015 | 5 0.1 |
| M1097 | 128 | exp12 | 2048bit-ECFP4 | SVM | 0.920 | 0.912 | 0.947 | 0.877 | 0.894 | 0.831 | 0.912 | 0.841 | 0.053 | 10 0.1 |
| M1098 | 128 | exp13 | 2048bit-ECFP4 | SVM | 0.880 | 0.829 | 0.888 | 0.868 | 0.848 | 0.750 | 0.878 | 0.842 | 0.006 | 500 0.1 |
| M1099 | 128 | exp14 | 2048bit-ECFP4 | SVM | 0.866 | 0.805 | 0.871 | 0.858 | 0.831 | 0.721 | 0.865 | 0.866 | 0.035 | 5 0.1 |
| M1100 | 128 | exp15 | 2048bit-ECFP4 | SVM | 0.873 | 0.874 | 0.929 | 0.783 | 0.826 | 0.729 | 0.856 | 0.853 | 0.027 | 10 0.1 |
| M1101 | 128 | exp16 | 2048bit-ECFP4 | SVM | 0.866 | 0.863 | 0.924 | 0.774 | 0.816 | 0.714 | 0.849 | 0.847 | 0.031 | 5 0.05 |
| M1102 | 128 | exp17 | 2048bit-ECFP4 | SVM | 0.899 | 0.868 | 0.918 | 0.868 | 0.868 | 0.786 | 0.893 | 0.839 | 0.029 | 5 0.1 |
| M1103 | 128 | exp18 | 2048bit-ECFP4 | SVM | 0.877 | 0.846 | 0.906 | 0.830 | 0.838 | 0.739 | 0.868 | 0.864 | 0.026 | 100000.0 0.1 |
| M1104 | 128 | exp19 | 2048bit-ECFP4 | SVM | 0.913 | 0.902 | 0.941 | 0.868 | 0.885 | 0.815 | 0.904 | 0.836 | 0.049 | 1 0.1 |
| M1105 | 128 | exp01 | 2048bit-ECFP4 | GBM | 0.841 | 0.844 | 0.918 | 0.717 | 0.776 | 0.658 | 0.818 | 0.855 | 0.079 | 200 0.1 |
| M1106 | 128 | exp02 | 2048bit-ECFP4 | GBM | 0.884 | 0.870 | 0.924 | 0.821 | 0.845 | 0.753 | 0.873 | 0.847 | 0.002 | 100 0.5 |
| M1107 | 128 | exp03 | 2048bit-ECFP4 | GBM | 0.870 | 0.850 | 0.912 | 0.802 | 0.825 | 0.722 | 0.857 | 0.844 | 0.019 | 200 0.5 |
| M1108 | 128 | exp04 | 2048bit-ECFP4 | GBM | 0.909 | 0.886 | 0.929 | 0.877 | 0.882 | 0.808 | 0.903 | 0.839 | 0.043 | 50 0.8 |
| M1109 | 128 | exp05 | 2048bit-ECFP4 | GBM | 0.866 | 0.822 | 0.888 | 0.830 | 0.826 | 0.717 | 0.859 | 0.851 | 0.025 | 1000 0.4 |
| M1110 | 128 | exp06 | 2048bit-ECFP4 | GBM | 0.884 | 0.870 | 0.924 | 0.821 | 0.845 | 0.753 | 0.873 | 0.843 | 0.002 | 600 0.6 |
| M1111 | 128 | exp07 | 2048bit-ECFP4 | GBM | 0.877 | 0.883 | 0.935 | 0.783 | 0.830 | 0.737 | 0.859 | 0.845 | 0.015 | 100 0.1 |
| M1112 | 128 | exp08 | 2048bit-ECFP4 | GBM | 0.888 | 0.864 | 0.918 | 0.840 | 0.852 | 0.762 | 0.879 | 0.858 | 0.006 | 400 0.1 |
| M1113 | 128 | exp09 | 2048bit-ECFP4 | GBM | 0.895 | 0.867 | 0.918 | 0.858 | 0.863 | 0.778 | 0.888 | 0.837 | 0.026 | 200 0.4 |
| M1114 | 128 | exp10 | 2048bit-ECFP4 | GBM | 0.899 | 0.906 | 0.947 | 0.821 | 0.861 | 0.784 | 0.884 | 0.845 | 0.016 | 50 0.6 |
| M1115 | 128 | exp11 | 2048bit-ECFP4 | GBM | 0.895 | 0.881 | 0.929 | 0.840 | 0.860 | 0.777 | 0.885 | 0.866 | 0.006 | 100 0.7 |
| M1116 | 128 | exp12 | 2048bit-ECFP4 | GBM | 0.906 | 0.892 | 0.935 | 0.858 | 0.875 | 0.800 | 0.897 | 0.838 | 0.037 | 900 0.1 |
| M1117 | 128 | exp13 | 2048bit-ECFP4 | GBM | 0.880 | 0.841 | 0.900 | 0.849 | 0.845 | 0.748 | 0.875 | 0.856 | 0.011 | 800 0.1 |
| M1118 | 128 | exp14 | 2048bit-ECFP4 | GBM | 0.862 | 0.804 | 0.871 | 0.849 | 0.826 | 0.713 | 0.860 | 0.865 | 0.039 | 200 0.8 |
| M1119 | 128 | exp15 | 2048bit-ECFP4 | GBM | 0.891 | 0.922 | 0.959 | 0.783 | 0.847 | 0.770 | 0.871 | 0.849 | 0.002 | 300 0.2 |
| M1120 | 128 | exp16 | 2048bit-ECFP4 | GBM | 0.844 | 0.846 | 0.918 | 0.726 | 0.782 | 0.666 | 0.822 | 0.859 | 0.077 | 200 0.3 |
| M1121 | 128 | exp17 | 2048bit-ECFP4 | GBM | 0.895 | 0.889 | 0.935 | 0.830 | 0.859 | 0.776 | 0.883 | 0.837 | 0.022 | 200 0.2 |
| M1122 | 128 | exp18 | 2048bit-ECFP4 | GBM | 0.884 | 0.863 | 0.918 | 0.830 | 0.846 | 0.754 | 0.874 | 0.854 | 0.008 | 1000 0.2 |
| M1123 | 128 | exp19 | 2048bit-ECFP4 | GBM | 0.888 | 0.871 | 0.924 | 0.830 | 0.850 | 0.761 | 0.877 | 0.830 | 0.020 | 10 0.8 |
| M1124 | 128 | exp01 | 2048bit-ECFP4 | RF | 0.859 | 0.868 | 0.929 | 0.745 | 0.802 | 0.698 | 0.837 | 0.858 | 0.056 | 700 |
| M1125 | 128 | exp02 | 2048bit-ECFP4 | RF | 0.873 | 0.874 | 0.929 | 0.783 | 0.826 | 0.729 | 0.856 | 0.841 | 0.015 | 300 |
| M1126 | 128 | exp03 | 2048bit-ECFP4 | RF | 0.873 | 0.859 | 0.918 | 0.802 | 0.829 | 0.730 | 0.860 | 0.846 | 0.017 | 1000 |
| M1127 | 128 | exp04 | 2048bit-ECFP4 | RF | 0.880 | 0.835 | 0.894 | 0.858 | 0.847 | 0.749 | 0.876 | 0.849 | 0.002 | 800 |
| M1128 | 128 | exp05 | 2048bit-ECFP4 | RF | 0.862 | 0.827 | 0.894 | 0.811 | 0.819 | 0.708 | 0.853 | 0.868 | 0.049 | 50 |
| M1129 | 128 | exp06 | 2048bit-ECFP4 | RF | 0.891 | 0.896 | 0.941 | 0.811 | 0.851 | 0.768 | 0.876 | 0.843 | 0.008 | 100 |
| M1130 | 128 | exp07 | 2048bit-ECFP4 | RF | 0.899 | 0.906 | 0.947 | 0.821 | 0.861 | 0.784 | 0.884 | 0.834 | 0.027 | 800 |
| M1131 | 128 | exp08 | 2048bit-ECFP4 | RF | 0.891 | 0.888 | 0.935 | 0.821 | 0.853 | 0.768 | 0.878 | 0.849 | 0.004 | 500 |
| M1132 | 128 | exp09 | 2048bit-ECFP4 | RF | 0.895 | 0.867 | 0.918 | 0.858 | 0.863 | 0.778 | 0.888 | 0.840 | 0.023 | 100 |
| M1133 | 128 | exp10 | 2048bit-ECFP4 | RF | 0.899 | 0.915 | 0.953 | 0.811 | 0.860 | 0.784 | 0.882 | 0.833 | 0.027 | 50 |
| M1134 | 128 | exp11 | 2048bit-ECFP4 | RF | 0.888 | 0.912 | 0.953 | 0.783 | 0.843 | 0.761 | 0.868 | 0.856 | 0.013 | 700 |
| M1135 | 128 | exp12 | 2048bit-ECFP4 | RF | 0.909 | 0.918 | 0.953 | 0.840 | 0.877 | 0.807 | 0.896 | 0.824 | 0.053 | 700 |
| M1136 | 128 | exp13 | 2048bit-ECFP4 | RF | 0.909 | 0.879 | 0.924 | 0.887 | 0.883 | 0.809 | 0.905 | 0.852 | 0.031 | 10 |
| M1137 | 128 | exp14 | 2048bit-ECFP4 | RF | 0.851 | 0.810 | 0.882 | 0.802 | 0.806 | 0.685 | 0.842 | 0.853 | 0.047 | 100 |
| M1138 | 128 | exp15 | 2048bit-ECFP4 | RF | 0.870 | 0.889 | 0.941 | 0.755 | 0.816 | 0.722 | 0.848 | 0.847 | 0.031 | 50 |
| M1139 | 128 | exp16 | 2048bit-ECFP4 | RF | 0.848 | 0.856 | 0.924 | 0.726 | 0.786 | 0.674 | 0.825 | 0.849 | 0.063 | 600 |
| M1140 | 128 | exp17 | 2048bit-ECFP4 | RF | 0.888 | 0.895 | 0.941 | 0.802 | 0.846 | 0.761 | 0.871 | 0.843 | 0.003 | 1000 |
| M1141 | 128 | exp18 | 2048bit-ECFP4 | RF | 0.906 | 0.917 | 0.953 | 0.830 | 0.871 | 0.800 | 0.891 | 0.847 | 0.024 | 200 |
| M1142 | 128 | exp19 | 2048bit-ECFP4 | RF | 0.909 | 0.909 | 0.947 | 0.849 | 0.878 | 0.807 | 0.898 | 0.830 | 0.048 | 50 |
| M1143 | 128 | exp01 | 2048bit-ECFP4 | DNN2 | 0.862 | 0.854 | 0.918 | 0.774 | 0.812 | 0.706 | 0.846 | 0.858 | 0.046 | 200 140 0.3 |
| M1144 | 128 | exp02 | 2048bit-ECFP4 | DNN2 | 0.884 | 0.870 | 0.924 | 0.821 | 0.845 | 0.753 | 0.873 | 0.843 | 0.002 | 200 80 0.3 |
| M1145 | 128 | exp03 | 2048bit-ECFP4 | DNN2 | 0.884 | 0.878 | 0.929 | 0.811 | 0.843 | 0.753 | 0.870 | 0.844 | 0.001 | 500 120 0.3 |
| M1146 | 128 | exp01 | 2048bit-ECFP4 | DNN3 | 0.848 | 0.814 | 0.888 | 0.783 | 0.798 | 0.676 | 0.836 | 0.858 | 0.060 | 600 140 0.2 |
| M1147 | 128 | exp02 | 2048bit-ECFP4 | DNN3 | 0.902 | 0.883 | 0.929 | 0.858 | 0.871 | 0.792 | 0.893 | 0.840 | 0.031 | 100 160 0.1 |
| M1148 | 128 | exp03 | 2048bit-ECFP4 | DNN3 | 0.880 | 0.876 | 0.929 | 0.802 | 0.837 | 0.745 | 0.866 | 0.847 | 0.010 | 200 80 0.1 |
| M1149 | 128 | exp01 | 1024bit-ECFP6 | KNN | 0.855 | 0.884 | 0.941 | 0.717 | 0.792 | 0.691 | 0.829 | 0.841 | 0.049 | 9 distance |
| M1150 | 128 | exp02 | 1024bit-ECFP6 | KNN | 0.862 | 0.886 | 0.941 | 0.736 | 0.804 | 0.707 | 0.839 | 0.830 | 0.026 | 13 distance |
| M1151 | 128 | exp03 | 1024bit-ECFP6 | KNN | 0.848 | 0.762 | 0.829 | 0.877 | 0.816 | 0.692 | 0.853 | 0.827 | 0.011 | 1 uniform |
| M1152 | 128 | exp04 | 1024bit-ECFP6 | KNN | 0.877 | 0.790 | 0.847 | 0.925 | 0.852 | 0.754 | 0.886 | 0.828 | 0.024 | 5 distance |
| M1153 | 128 | exp05 | 1024bit-ECFP6 | KNN | 0.837 | 0.761 | 0.835 | 0.840 | 0.798 | 0.664 | 0.837 | 0.844 | 0.046 | 3 uniform |
| M1154 | 128 | exp06 | 1024bit-ECFP6 | KNN | 0.891 | 0.865 | 0.918 | 0.849 | 0.857 | 0.770 | 0.883 | 0.830 | 0.027 | 5 uniform |
| M1155 | 128 | exp07 | 1024bit-ECFP6 | KNN | 0.895 | 0.874 | 0.924 | 0.849 | 0.861 | 0.777 | 0.887 | 0.827 | 0.034 | 5 distance |
| M1156 | 128 | exp08 | 1024bit-ECFP6 | KNN | 0.877 | 0.875 | 0.929 | 0.792 | 0.832 | 0.737 | 0.861 | 0.829 | 0.003 | 7 distance |
| M1157 | 128 | exp09 | 1024bit-ECFP6 | KNN | 0.866 | 0.811 | 0.876 | 0.849 | 0.829 | 0.720 | 0.863 | 0.824 | 0.005 | 5 uniform |
| M1158 | 128 | exp10 | 1024bit-ECFP6 | KNN | 0.891 | 0.904 | 0.947 | 0.802 | 0.850 | 0.769 | 0.875 | 0.826 | 0.024 | 7 uniform |
| M1159 | 128 | exp11 | 1024bit-ECFP6 | KNN | 0.873 | 0.826 | 0.888 | 0.849 | 0.837 | 0.734 | 0.869 | 0.835 | 0.002 | 1 uniform |
| M1160 | 128 | exp12 | 1024bit-ECFP6 | KNN | 0.888 | 0.844 | 0.900 | 0.868 | 0.856 | 0.764 | 0.884 | 0.816 | 0.040 | 3 uniform |
| M1161 | 128 | exp13 | 1024bit-ECFP6 | KNN | 0.855 | 0.766 | 0.829 | 0.896 | 0.826 | 0.710 | 0.863 | 0.808 | 0.018 | 3 distance |
| M1162 | 128 | exp14 | 1024bit-ECFP6 | KNN | 0.844 | 0.774 | 0.847 | 0.840 | 0.805 | 0.677 | 0.843 | 0.831 | 0.026 | 5 uniform |
| M1163 | 128 | exp15 | 1024bit-ECFP6 | KNN | 0.830 | 0.771 | 0.853 | 0.792 | 0.781 | 0.642 | 0.823 | 0.816 | 0.035 | 1 uniform |
| M1164 | 128 | exp16 | 1024bit-ECFP6 | KNN | 0.855 | 0.837 | 0.906 | 0.774 | 0.804 | 0.691 | 0.840 | 0.836 | 0.032 | 5 distance |
| M1165 | 128 | exp17 | 1024bit-ECFP6 | KNN | 0.888 | 0.838 | 0.894 | 0.877 | 0.857 | 0.765 | 0.885 | 0.818 | 0.039 | 1 uniform |
| M1166 | 128 | exp18 | 1024bit-ECFP6 | KNN | 0.844 | 0.784 | 0.859 | 0.821 | 0.802 | 0.674 | 0.840 | 0.839 | 0.037 | 5 uniform |
| M1167 | 128 | exp19 | 1024bit-ECFP6 | KNN | 0.888 | 0.864 | 0.918 | 0.840 | 0.852 | 0.762 | 0.879 | 0.816 | 0.036 | 7 distance |
| M1168 | 128 | exp01 | 1024bit-ECFP6 | SVM | 0.877 | 0.883 | 0.935 | 0.783 | 0.830 | 0.737 | 0.859 | 0.852 | 0.022 | 1 0.1 |
| M1169 | 128 | exp02 | 1024bit-ECFP6 | SVM | 0.891 | 0.852 | 0.906 | 0.868 | 0.860 | 0.771 | 0.887 | 0.846 | 0.014 | 5 0.1 |
| M1170 | 128 | exp03 | 1024bit-ECFP6 | SVM | 0.873 | 0.866 | 0.924 | 0.792 | 0.828 | 0.729 | 0.858 | 0.850 | 0.022 | 5 0.1 |
| M1171 | 128 | exp04 | 1024bit-ECFP6 | SVM | 0.895 | 0.841 | 0.894 | 0.896 | 0.868 | 0.782 | 0.895 | 0.850 | 0.018 | 5 0.1 |
| M1172 | 128 | exp05 | 1024bit-ECFP6 | SVM | 0.877 | 0.846 | 0.906 | 0.830 | 0.838 | 0.739 | 0.868 | 0.866 | 0.028 | 5 0.1 |
| M1173 | 128 | exp06 | 1024bit-ECFP6 | SVM | 0.891 | 0.888 | 0.935 | 0.821 | 0.853 | 0.768 | 0.878 | 0.847 | 0.006 | 5 0.1 |
| M1174 | 128 | exp07 | 1024bit-ECFP6 | SVM | 0.888 | 0.895 | 0.941 | 0.802 | 0.846 | 0.761 | 0.871 | 0.846 | 0.000 | 5 0.05 |
| M1175 | 128 | exp08 | 1024bit-ECFP6 | SVM | 0.895 | 0.867 | 0.918 | 0.858 | 0.863 | 0.778 | 0.888 | 0.845 | 0.018 | 10000.0 0.1 |
| M1176 | 128 | exp09 | 1024bit-ECFP6 | SVM | 0.902 | 0.907 | 0.947 | 0.830 | 0.867 | 0.792 | 0.888 | 0.856 | 0.011 | 10 0.1 |
| M1177 | 128 | exp10 | 1024bit-ECFP6 | SVM | 0.906 | 0.926 | 0.959 | 0.821 | 0.870 | 0.800 | 0.890 | 0.846 | 0.024 | 5 0.1 |
| M1178 | 128 | exp11 | 1024bit-ECFP6 | SVM | 0.877 | 0.875 | 0.929 | 0.792 | 0.832 | 0.737 | 0.861 | 0.850 | 0.018 | 5 0.1 |
| M1179 | 128 | exp12 | 1024bit-ECFP6 | SVM | 0.928 | 0.948 | 0.971 | 0.858 | 0.901 | 0.847 | 0.914 | 0.830 | 0.071 | 5 0.1 |
| M1180 | 128 | exp13 | 1024bit-ECFP6 | SVM | 0.906 | 0.864 | 0.912 | 0.896 | 0.880 | 0.803 | 0.904 | 0.829 | 0.051 | 5 0.05 |
| M1181 | 128 | exp14 | 1024bit-ECFP6 | SVM | 0.859 | 0.802 | 0.871 | 0.840 | 0.820 | 0.704 | 0.855 | 0.860 | 0.040 | 5 0.1 |
| M1182 | 128 | exp15 | 1024bit-ECFP6 | SVM | 0.891 | 0.922 | 0.959 | 0.783 | 0.847 | 0.770 | 0.871 | 0.843 | 0.004 | 5 0.1 |
| M1183 | 128 | exp16 | 1024bit-ECFP6 | SVM | 0.880 | 0.892 | 0.941 | 0.783 | 0.834 | 0.745 | 0.862 | 0.858 | 0.024 | 5 0.1 |
| M1184 | 128 | exp17 | 1024bit-ECFP6 | SVM | 0.895 | 0.860 | 0.912 | 0.868 | 0.864 | 0.778 | 0.890 | 0.843 | 0.021 | 10 0.1 |
| M1185 | 128 | exp18 | 1024bit-ECFP6 | SVM | 0.884 | 0.885 | 0.935 | 0.802 | 0.842 | 0.753 | 0.869 | 0.855 | 0.013 | 1 0.1 |
| M1186 | 128 | exp19 | 1024bit-ECFP6 | SVM | 0.913 | 0.910 | 0.947 | 0.858 | 0.883 | 0.815 | 0.902 | 0.841 | 0.042 | 1 0.1 |
| M1187 | 128 | exp01 | 1024bit-ECFP6 | GBM | 0.862 | 0.878 | 0.935 | 0.745 | 0.806 | 0.706 | 0.840 | 0.849 | 0.043 | 400 0.2 |
| M1188 | 128 | exp02 | 1024bit-ECFP6 | GBM | 0.884 | 0.894 | 0.941 | 0.792 | 0.840 | 0.753 | 0.867 | 0.830 | 0.010 | 100 0.4 |
| M1189 | 128 | exp03 | 1024bit-ECFP6 | GBM | 0.899 | 0.906 | 0.947 | 0.821 | 0.861 | 0.784 | 0.884 | 0.838 | 0.023 | 1000 0.1 |
| M1190 | 128 | exp04 | 1024bit-ECFP6 | GBM | 0.899 | 0.868 | 0.918 | 0.868 | 0.868 | 0.786 | 0.893 | 0.840 | 0.028 | 200 0.4 |
| M1191 | 128 | exp05 | 1024bit-ECFP6 | GBM | 0.873 | 0.826 | 0.888 | 0.849 | 0.837 | 0.734 | 0.869 | 0.851 | 0.014 | 300 0.2 |
| M1192 | 128 | exp06 | 1024bit-ECFP6 | GBM | 0.873 | 0.859 | 0.918 | 0.802 | 0.829 | 0.730 | 0.860 | 0.833 | 0.004 | 700 0.1 |
| M1193 | 128 | exp07 | 1024bit-ECFP6 | GBM | 0.884 | 0.894 | 0.941 | 0.792 | 0.840 | 0.753 | 0.867 | 0.837 | 0.003 | 100 0.8 |
| M1194 | 128 | exp08 | 1024bit-ECFP6 | GBM | 0.888 | 0.879 | 0.929 | 0.821 | 0.849 | 0.761 | 0.875 | 0.844 | 0.005 | 200 0.2 |
| M1195 | 128 | exp09 | 1024bit-ECFP6 | GBM | 0.902 | 0.916 | 0.953 | 0.821 | 0.866 | 0.792 | 0.887 | 0.843 | 0.023 | 400 0.2 |
| M1196 | 128 | exp10 | 1024bit-ECFP6 | GBM | 0.877 | 0.867 | 0.924 | 0.802 | 0.833 | 0.737 | 0.863 | 0.832 | 0.001 | 100 0.4 |
| M1197 | 128 | exp11 | 1024bit-ECFP6 | GBM | 0.884 | 0.894 | 0.941 | 0.792 | 0.840 | 0.753 | 0.867 | 0.859 | 0.019 | 200 0.7 |
| M1198 | 128 | exp12 | 1024bit-ECFP6 | GBM | 0.909 | 0.945 | 0.971 | 0.811 | 0.873 | 0.809 | 0.891 | 0.831 | 0.042 | 200 0.2 |
| M1199 | 128 | exp13 | 1024bit-ECFP6 | GBM | 0.895 | 0.841 | 0.894 | 0.896 | 0.868 | 0.782 | 0.895 | 0.835 | 0.033 | 400 0.1 |
| M1200 | 128 | exp14 | 1024bit-ECFP6 | GBM | 0.870 | 0.843 | 0.906 | 0.811 | 0.827 | 0.723 | 0.859 | 0.849 | 0.022 | 100 0.5 |
| M1201 | 128 | exp15 | 1024bit-ECFP6 | GBM | 0.873 | 0.874 | 0.929 | 0.783 | 0.826 | 0.729 | 0.856 | 0.837 | 0.011 | 200 0.8 |
| M1202 | 128 | exp16 | 1024bit-ECFP6 | GBM | 0.870 | 0.872 | 0.929 | 0.774 | 0.820 | 0.721 | 0.852 | 0.834 | 0.014 | 200 0.5 |
| M1203 | 128 | exp17 | 1024bit-ECFP6 | GBM | 0.877 | 0.867 | 0.924 | 0.802 | 0.833 | 0.737 | 0.863 | 0.831 | 0.002 | 50 0.8 |
| M1204 | 128 | exp18 | 1024bit-ECFP6 | GBM | 0.891 | 0.896 | 0.941 | 0.811 | 0.851 | 0.768 | 0.876 | 0.839 | 0.012 | 50 0.5 |
| M1205 | 128 | exp19 | 1024bit-ECFP6 | GBM | 0.899 | 0.882 | 0.929 | 0.849 | 0.865 | 0.784 | 0.889 | 0.841 | 0.024 | 100 0.3 |
| M1206 | 128 | exp01 | 1024bit-ECFP6 | RF | 0.862 | 0.925 | 0.965 | 0.698 | 0.796 | 0.711 | 0.831 | 0.845 | 0.049 | 900 |
| M1207 | 128 | exp02 | 1024bit-ECFP6 | RF | 0.891 | 0.913 | 0.953 | 0.792 | 0.848 | 0.769 | 0.873 | 0.833 | 0.015 | 50 |
| M1208 | 128 | exp03 | 1024bit-ECFP6 | RF | 0.902 | 0.916 | 0.953 | 0.821 | 0.866 | 0.792 | 0.887 | 0.834 | 0.032 | 50 |
| M1209 | 128 | exp04 | 1024bit-ECFP6 | RF | 0.899 | 0.875 | 0.924 | 0.858 | 0.867 | 0.785 | 0.891 | 0.840 | 0.027 | 500 |
| M1210 | 128 | exp05 | 1024bit-ECFP6 | RF | 0.859 | 0.832 | 0.900 | 0.792 | 0.812 | 0.699 | 0.846 | 0.853 | 0.041 | 10 |
| M1211 | 128 | exp06 | 1024bit-ECFP6 | RF | 0.899 | 0.943 | 0.971 | 0.783 | 0.856 | 0.787 | 0.877 | 0.831 | 0.025 | 100 |
| M1212 | 128 | exp07 | 1024bit-ECFP6 | RF | 0.909 | 0.976 | 0.988 | 0.783 | 0.869 | 0.813 | 0.885 | 0.844 | 0.025 | 100 |
| M1213 | 128 | exp08 | 1024bit-ECFP6 | RF | 0.895 | 0.905 | 0.947 | 0.811 | 0.856 | 0.776 | 0.879 | 0.841 | 0.015 | 500 |
| M1214 | 128 | exp09 | 1024bit-ECFP6 | RF | 0.891 | 0.904 | 0.947 | 0.802 | 0.850 | 0.769 | 0.875 | 0.838 | 0.012 | 800 |
| M1215 | 128 | exp10 | 1024bit-ECFP6 | RF | 0.899 | 0.943 | 0.971 | 0.783 | 0.856 | 0.787 | 0.877 | 0.828 | 0.028 | 600 |
| M1216 | 128 | exp11 | 1024bit-ECFP6 | RF | 0.877 | 0.891 | 0.941 | 0.774 | 0.828 | 0.737 | 0.857 | 0.841 | 0.013 | 300 |
| M1217 | 128 | exp12 | 1024bit-ECFP6 | RF | 0.909 | 0.935 | 0.965 | 0.821 | 0.874 | 0.808 | 0.893 | 0.832 | 0.042 | 50 |
| M1218 | 128 | exp13 | 1024bit-ECFP6 | RF | 0.888 | 0.857 | 0.912 | 0.849 | 0.853 | 0.762 | 0.881 | 0.835 | 0.018 | 10 |
| M1219 | 128 | exp14 | 1024bit-ECFP6 | RF | 0.888 | 0.887 | 0.935 | 0.811 | 0.847 | 0.761 | 0.873 | 0.842 | 0.005 | 100 |
| M1220 | 128 | exp15 | 1024bit-ECFP6 | RF | 0.873 | 0.899 | 0.947 | 0.755 | 0.821 | 0.730 | 0.851 | 0.830 | 0.009 | 50 |
| M1221 | 128 | exp16 | 1024bit-ECFP6 | RF | 0.862 | 0.905 | 0.953 | 0.717 | 0.800 | 0.708 | 0.835 | 0.837 | 0.037 | 100 |
| M1222 | 128 | exp17 | 1024bit-ECFP6 | RF | 0.880 | 0.884 | 0.935 | 0.792 | 0.836 | 0.745 | 0.864 | 0.835 | 0.001 | 100 |
| M1223 | 128 | exp18 | 1024bit-ECFP6 | RF | 0.891 | 0.932 | 0.965 | 0.774 | 0.845 | 0.771 | 0.869 | 0.838 | 0.007 | 400 |
| M1224 | 128 | exp19 | 1024bit-ECFP6 | RF | 0.909 | 0.909 | 0.947 | 0.849 | 0.878 | 0.807 | 0.898 | 0.837 | 0.041 | 700 |
| M1225 | 128 | exp01 | 1024bit-ECFP6 | DNN2 | 0.877 | 0.883 | 0.935 | 0.783 | 0.830 | 0.737 | 0.859 | 0.844 | 0.014 | 100 160 0.2 |
| M1226 | 128 | exp02 | 1024bit-ECFP6 | DNN2 | 0.888 | 0.864 | 0.918 | 0.840 | 0.852 | 0.762 | 0.879 | 0.819 | 0.033 | 300 120 0.5 |
| M1227 | 128 | exp03 | 1024bit-ECFP6 | DNN2 | 0.851 | 0.810 | 0.882 | 0.802 | 0.806 | 0.685 | 0.842 | 0.840 | 0.034 | 500 80 0.1 |
| M1228 | 128 | exp01 | 1024bit-ECFP6 | DNN3 | 0.877 | 0.875 | 0.929 | 0.792 | 0.832 | 0.737 | 0.861 | 0.844 | 0.012 | 600 80 0.5 |
| M1229 | 128 | exp02 | 1024bit-ECFP6 | DNN3 | 0.873 | 0.874 | 0.929 | 0.783 | 0.826 | 0.729 | 0.856 | 0.821 | 0.005 | 100 80 0.1 |
| M1230 | 128 | exp03 | 1024bit-ECFP6 | DNN3 | 0.859 | 0.825 | 0.894 | 0.802 | 0.813 | 0.700 | 0.848 | 0.844 | 0.031 | 600 100 0.4 |
| M1231 | 128 | exp01 | 2048bit-ECFP6 | KNN | 0.855 | 0.893 | 0.947 | 0.708 | 0.789 | 0.692 | 0.827 | 0.843 | 0.054 | 5 uniform |
| M1232 | 128 | exp02 | 2048bit-ECFP6 | KNN | 0.862 | 0.878 | 0.935 | 0.745 | 0.806 | 0.706 | 0.840 | 0.841 | 0.035 | 7 distance |
| M1233 | 128 | exp03 | 2048bit-ECFP6 | KNN | 0.888 | 0.838 | 0.894 | 0.877 | 0.857 | 0.765 | 0.885 | 0.835 | 0.022 | 3 distance |
| M1234 | 128 | exp04 | 2048bit-ECFP6 | KNN | 0.848 | 0.758 | 0.824 | 0.887 | 0.817 | 0.695 | 0.855 | 0.829 | 0.012 | 3 distance |
| M1235 | 128 | exp05 | 2048bit-ECFP6 | KNN | 0.862 | 0.804 | 0.871 | 0.849 | 0.826 | 0.713 | 0.860 | 0.844 | 0.018 | 5 uniform |
| M1236 | 128 | exp06 | 2048bit-ECFP6 | KNN | 0.888 | 0.895 | 0.941 | 0.802 | 0.846 | 0.761 | 0.871 | 0.832 | 0.014 | 5 uniform |
| M1237 | 128 | exp07 | 2048bit-ECFP6 | KNN | 0.899 | 0.924 | 0.959 | 0.802 | 0.859 | 0.785 | 0.881 | 0.834 | 0.025 | 9 distance |
| M1238 | 128 | exp08 | 2048bit-ECFP6 | KNN | 0.877 | 0.860 | 0.918 | 0.811 | 0.835 | 0.738 | 0.865 | 0.839 | 0.004 | 5 distance |
| M1239 | 128 | exp09 | 2048bit-ECFP6 | KNN | 0.884 | 0.830 | 0.888 | 0.877 | 0.853 | 0.758 | 0.883 | 0.836 | 0.017 | 3 distance |
| M1240 | 128 | exp10 | 2048bit-ECFP6 | KNN | 0.880 | 0.861 | 0.918 | 0.821 | 0.841 | 0.746 | 0.869 | 0.809 | 0.032 | 3 distance |
| M1241 | 128 | exp11 | 2048bit-ECFP6 | KNN | 0.888 | 0.871 | 0.924 | 0.830 | 0.850 | 0.761 | 0.877 | 0.847 | 0.003 | 7 distance |
| M1242 | 128 | exp12 | 2048bit-ECFP6 | KNN | 0.891 | 0.858 | 0.912 | 0.858 | 0.858 | 0.770 | 0.885 | 0.833 | 0.025 | 3 distance |
| M1243 | 128 | exp13 | 2048bit-ECFP6 | KNN | 0.880 | 0.835 | 0.894 | 0.858 | 0.847 | 0.749 | 0.876 | 0.828 | 0.019 | 5 distance |
| M1244 | 128 | exp14 | 2048bit-ECFP6 | KNN | 0.859 | 0.786 | 0.853 | 0.868 | 0.825 | 0.709 | 0.861 | 0.845 | 0.020 | 5 distance |
| M1245 | 128 | exp15 | 2048bit-ECFP6 | KNN | 0.848 | 0.802 | 0.876 | 0.802 | 0.802 | 0.678 | 0.839 | 0.833 | 0.030 | 1 uniform |
| M1246 | 128 | exp16 | 2048bit-ECFP6 | KNN | 0.851 | 0.857 | 0.924 | 0.736 | 0.792 | 0.682 | 0.830 | 0.839 | 0.047 | 5 distance |
| M1247 | 128 | exp17 | 2048bit-ECFP6 | KNN | 0.877 | 0.846 | 0.906 | 0.830 | 0.838 | 0.739 | 0.868 | 0.841 | 0.003 | 3 distance |
| M1248 | 128 | exp18 | 2048bit-ECFP6 | KNN | 0.873 | 0.838 | 0.900 | 0.830 | 0.834 | 0.732 | 0.865 | 0.846 | 0.012 | 3 distance |
| M1249 | 128 | exp19 | 2048bit-ECFP6 | KNN | 0.884 | 0.843 | 0.900 | 0.858 | 0.850 | 0.756 | 0.879 | 0.839 | 0.011 | 3 distance |
| M1250 | 128 | exp01 | 2048bit-ECFP6 | SVM | 0.877 | 0.883 | 0.935 | 0.783 | 0.830 | 0.737 | 0.859 | 0.858 | 0.028 | 5 0.05 |
| M1251 | 128 | exp02 | 2048bit-ECFP6 | SVM | 0.888 | 0.850 | 0.906 | 0.858 | 0.854 | 0.763 | 0.882 | 0.840 | 0.014 | 5 0.1 |
| M1252 | 128 | exp03 | 2048bit-ECFP6 | SVM | 0.888 | 0.864 | 0.918 | 0.840 | 0.852 | 0.762 | 0.879 | 0.854 | 0.002 | 5 0.1 |
| M1253 | 128 | exp04 | 2048bit-ECFP6 | SVM | 0.880 | 0.812 | 0.871 | 0.896 | 0.852 | 0.755 | 0.883 | 0.858 | 0.006 | 10 0.1 |
| M1254 | 128 | exp05 | 2048bit-ECFP6 | SVM | 0.884 | 0.836 | 0.894 | 0.868 | 0.852 | 0.757 | 0.881 | 0.863 | 0.011 | 10 0.1 |
| M1255 | 128 | exp06 | 2048bit-ECFP6 | SVM | 0.909 | 0.901 | 0.941 | 0.858 | 0.879 | 0.807 | 0.899 | 0.855 | 0.024 | 5 0.1 |
| M1256 | 128 | exp07 | 2048bit-ECFP6 | SVM | 0.895 | 0.897 | 0.941 | 0.821 | 0.857 | 0.776 | 0.881 | 0.850 | 0.007 | 1 0.1 |
| M1257 | 128 | exp08 | 2048bit-ECFP6 | SVM | 0.866 | 0.817 | 0.882 | 0.840 | 0.828 | 0.718 | 0.861 | 0.854 | 0.026 | 5 0.1 |
| M1258 | 128 | exp09 | 2048bit-ECFP6 | SVM | 0.899 | 0.890 | 0.935 | 0.840 | 0.864 | 0.784 | 0.887 | 0.859 | 0.005 | 10 0.1 |
| M1259 | 128 | exp10 | 2048bit-ECFP6 | SVM | 0.877 | 0.867 | 0.924 | 0.802 | 0.833 | 0.737 | 0.863 | 0.841 | 0.008 | 5 0.05 |
| M1260 | 128 | exp11 | 2048bit-ECFP6 | SVM | 0.880 | 0.854 | 0.912 | 0.830 | 0.842 | 0.746 | 0.871 | 0.862 | 0.020 | 10 0.1 |
| M1261 | 128 | exp12 | 2048bit-ECFP6 | SVM | 0.906 | 0.926 | 0.959 | 0.821 | 0.870 | 0.800 | 0.890 | 0.833 | 0.037 | 5 0.05 |
| M1262 | 128 | exp13 | 2048bit-ECFP6 | SVM | 0.888 | 0.850 | 0.906 | 0.858 | 0.854 | 0.763 | 0.882 | 0.845 | 0.009 | 50000.0 0.1 |
| M1263 | 128 | exp14 | 2048bit-ECFP6 | SVM | 0.873 | 0.832 | 0.894 | 0.840 | 0.836 | 0.732 | 0.867 | 0.855 | 0.019 | 5 0.1 |
| M1264 | 128 | exp15 | 2048bit-ECFP6 | SVM | 0.877 | 0.875 | 0.929 | 0.792 | 0.832 | 0.737 | 0.861 | 0.844 | 0.012 | 10 0.1 |
| M1265 | 128 | exp16 | 2048bit-ECFP6 | SVM | 0.877 | 0.867 | 0.924 | 0.802 | 0.833 | 0.737 | 0.863 | 0.849 | 0.016 | 500 0.1 |
| M1266 | 128 | exp17 | 2048bit-ECFP6 | SVM | 0.891 | 0.880 | 0.929 | 0.830 | 0.854 | 0.769 | 0.879 | 0.847 | 0.007 | 10 0.1 |
| M1267 | 128 | exp18 | 2048bit-ECFP6 | SVM | 0.895 | 0.860 | 0.912 | 0.868 | 0.864 | 0.778 | 0.890 | 0.849 | 0.015 | 5 0.1 |
| M1268 | 128 | exp19 | 2048bit-ECFP6 | SVM | 0.909 | 0.909 | 0.947 | 0.849 | 0.878 | 0.807 | 0.898 | 0.836 | 0.042 | 1 0.1 |
| M1269 | 128 | exp01 | 2048bit-ECFP6 | GBM | 0.848 | 0.872 | 0.935 | 0.708 | 0.781 | 0.675 | 0.822 | 0.855 | 0.074 | 100 0.4 |
| M1270 | 128 | exp02 | 2048bit-ECFP6 | GBM | 0.891 | 0.880 | 0.929 | 0.830 | 0.854 | 0.769 | 0.879 | 0.830 | 0.024 | 400 0.5 |
| M1271 | 128 | exp03 | 2048bit-ECFP6 | GBM | 0.870 | 0.865 | 0.924 | 0.783 | 0.822 | 0.722 | 0.854 | 0.839 | 0.017 | 1000 0.1 |
| M1272 | 128 | exp04 | 2048bit-ECFP6 | GBM | 0.891 | 0.888 | 0.935 | 0.821 | 0.853 | 0.768 | 0.878 | 0.835 | 0.018 | 400 0.1 |
| M1273 | 128 | exp05 | 2048bit-ECFP6 | GBM | 0.877 | 0.840 | 0.900 | 0.840 | 0.840 | 0.740 | 0.870 | 0.835 | 0.005 | 300 0.6 |
| M1274 | 128 | exp06 | 2048bit-ECFP6 | GBM | 0.866 | 0.863 | 0.924 | 0.774 | 0.816 | 0.714 | 0.849 | 0.833 | 0.017 | 500 0.2 |
| M1275 | 128 | exp07 | 2048bit-ECFP6 | GBM | 0.877 | 0.867 | 0.924 | 0.802 | 0.833 | 0.737 | 0.863 | 0.843 | 0.010 | 300 0.3 |
| M1276 | 128 | exp08 | 2048bit-ECFP6 | GBM | 0.880 | 0.841 | 0.900 | 0.849 | 0.845 | 0.748 | 0.875 | 0.851 | 0.006 | 300 0.7 |
| M1277 | 128 | exp09 | 2048bit-ECFP6 | GBM | 0.888 | 0.887 | 0.935 | 0.811 | 0.847 | 0.761 | 0.873 | 0.852 | 0.005 | 300 0.3 |
| M1278 | 128 | exp10 | 2048bit-ECFP6 | GBM | 0.888 | 0.941 | 0.971 | 0.755 | 0.838 | 0.764 | 0.863 | 0.829 | 0.009 | 50 0.2 |
| M1279 | 128 | exp11 | 2048bit-ECFP6 | GBM | 0.855 | 0.837 | 0.906 | 0.774 | 0.804 | 0.691 | 0.840 | 0.845 | 0.041 | 200 0.2 |
| M1280 | 128 | exp12 | 2048bit-ECFP6 | GBM | 0.909 | 0.926 | 0.959 | 0.830 | 0.876 | 0.808 | 0.894 | 0.817 | 0.059 | 100 0.8 |
| M1281 | 128 | exp13 | 2048bit-ECFP6 | GBM | 0.902 | 0.876 | 0.924 | 0.868 | 0.872 | 0.793 | 0.896 | 0.839 | 0.033 | 300 0.2 |
| M1282 | 128 | exp14 | 2048bit-ECFP6 | GBM | 0.855 | 0.784 | 0.853 | 0.858 | 0.820 | 0.701 | 0.855 | 0.847 | 0.027 | 1000 0.7 |
| M1283 | 128 | exp15 | 2048bit-ECFP6 | GBM | 0.862 | 0.847 | 0.912 | 0.783 | 0.814 | 0.706 | 0.848 | 0.836 | 0.022 | 700 0.7 |
| M1284 | 128 | exp16 | 2048bit-ECFP6 | GBM | 0.862 | 0.847 | 0.912 | 0.783 | 0.814 | 0.706 | 0.848 | 0.844 | 0.030 | 100 0.6 |
| M1285 | 128 | exp17 | 2048bit-ECFP6 | GBM | 0.862 | 0.862 | 0.924 | 0.764 | 0.810 | 0.706 | 0.844 | 0.841 | 0.031 | 400 0.8 |
| M1286 | 128 | exp18 | 2048bit-ECFP6 | GBM | 0.899 | 0.933 | 0.965 | 0.792 | 0.857 | 0.786 | 0.879 | 0.843 | 0.014 | 200 0.1 |
| M1287 | 128 | exp19 | 2048bit-ECFP6 | GBM | 0.917 | 0.895 | 0.935 | 0.887 | 0.891 | 0.824 | 0.911 | 0.831 | 0.060 | 400 0.2 |
| M1288 | 128 | exp01 | 2048bit-ECFP6 | RF | 0.862 | 0.895 | 0.947 | 0.726 | 0.802 | 0.707 | 0.837 | 0.843 | 0.041 | 900 |
| M1289 | 128 | exp02 | 2048bit-ECFP6 | RF | 0.888 | 0.912 | 0.953 | 0.783 | 0.843 | 0.761 | 0.868 | 0.837 | 0.006 | 500 |
| M1290 | 128 | exp03 | 2048bit-ECFP6 | RF | 0.895 | 0.905 | 0.947 | 0.811 | 0.856 | 0.776 | 0.879 | 0.854 | 0.002 | 300 |
| M1291 | 128 | exp04 | 2048bit-ECFP6 | RF | 0.902 | 0.869 | 0.918 | 0.877 | 0.873 | 0.794 | 0.897 | 0.851 | 0.022 | 100 |
| M1292 | 128 | exp05 | 2048bit-ECFP6 | RF | 0.859 | 0.819 | 0.888 | 0.811 | 0.815 | 0.701 | 0.850 | 0.858 | 0.043 | 100 |
| M1293 | 128 | exp06 | 2048bit-ECFP6 | RF | 0.877 | 0.860 | 0.918 | 0.811 | 0.835 | 0.738 | 0.865 | 0.835 | 0.000 | 10 |
| M1294 | 128 | exp07 | 2048bit-ECFP6 | RF | 0.902 | 0.907 | 0.947 | 0.830 | 0.867 | 0.792 | 0.888 | 0.835 | 0.032 | 600 |
| M1295 | 128 | exp08 | 2048bit-ECFP6 | RF | 0.899 | 0.882 | 0.929 | 0.849 | 0.865 | 0.784 | 0.889 | 0.845 | 0.020 | 50 |
| M1296 | 128 | exp09 | 2048bit-ECFP6 | RF | 0.906 | 0.908 | 0.947 | 0.840 | 0.873 | 0.800 | 0.893 | 0.853 | 0.020 | 400 |
| M1297 | 128 | exp10 | 2048bit-ECFP6 | RF | 0.891 | 0.932 | 0.965 | 0.774 | 0.845 | 0.771 | 0.869 | 0.832 | 0.013 | 300 |
| M1298 | 128 | exp11 | 2048bit-ECFP6 | RF | 0.888 | 0.895 | 0.941 | 0.802 | 0.846 | 0.761 | 0.871 | 0.857 | 0.011 | 600 |
| M1299 | 128 | exp12 | 2048bit-ECFP6 | RF | 0.909 | 0.926 | 0.959 | 0.830 | 0.876 | 0.808 | 0.894 | 0.826 | 0.050 | 100 |
| M1300 | 128 | exp13 | 2048bit-ECFP6 | RF | 0.906 | 0.900 | 0.941 | 0.849 | 0.874 | 0.800 | 0.895 | 0.840 | 0.034 | 1000 |
| M1301 | 128 | exp14 | 2048bit-ECFP6 | RF | 0.859 | 0.825 | 0.894 | 0.802 | 0.813 | 0.700 | 0.848 | 0.849 | 0.036 | 900 |
| M1302 | 128 | exp15 | 2048bit-ECFP6 | RF | 0.873 | 0.882 | 0.935 | 0.774 | 0.824 | 0.729 | 0.855 | 0.837 | 0.013 | 50 |
| M1303 | 128 | exp16 | 2048bit-ECFP6 | RF | 0.859 | 0.860 | 0.924 | 0.755 | 0.804 | 0.698 | 0.840 | 0.857 | 0.053 | 100 |
| M1304 | 128 | exp17 | 2048bit-ECFP6 | RF | 0.877 | 0.891 | 0.941 | 0.774 | 0.828 | 0.737 | 0.857 | 0.851 | 0.023 | 700 |
| M1305 | 128 | exp18 | 2048bit-ECFP6 | RF | 0.895 | 0.905 | 0.947 | 0.811 | 0.856 | 0.776 | 0.879 | 0.841 | 0.015 | 700 |
| M1306 | 128 | exp19 | 2048bit-ECFP6 | RF | 0.924 | 0.929 | 0.959 | 0.868 | 0.898 | 0.838 | 0.913 | 0.832 | 0.066 | 1000 |
| M1307 | 128 | exp01 | 2048bit-ECFP6 | DNN2 | 0.870 | 0.865 | 0.924 | 0.783 | 0.822 | 0.722 | 0.854 | 0.834 | 0.012 | 400 160 0.3 |
| M1308 | 128 | exp02 | 2048bit-ECFP6 | DNN2 | 0.880 | 0.848 | 0.906 | 0.840 | 0.844 | 0.747 | 0.873 | 0.843 | 0.001 | 600 100 0.2 |
| M1309 | 128 | exp03 | 2048bit-ECFP6 | DNN2 | 0.866 | 0.829 | 0.894 | 0.821 | 0.825 | 0.716 | 0.857 | 0.839 | 0.014 | 400 120 0.5 |
| M1310 | 128 | exp01 | 2048bit-ECFP6 | DNN3 | 0.844 | 0.818 | 0.894 | 0.764 | 0.790 | 0.668 | 0.829 | 0.833 | 0.042 | 100 60 0.2 |
| M1311 | 128 | exp02 | 2048bit-ECFP6 | DNN3 | 0.891 | 0.873 | 0.924 | 0.840 | 0.856 | 0.769 | 0.882 | 0.840 | 0.016 | 600 60 0.1 |
| M1312 | 128 | exp03 | 2048bit-ECFP6 | DNN3 | 0.870 | 0.830 | 0.894 | 0.830 | 0.830 | 0.724 | 0.862 | 0.845 | 0.015 | 100 60 0.2 |

* Optimal parameters for each machine-learning methods as follows

(1) KNN: two parameters are the number of nearest neighbors (K) and weighting schemes (uniform weight or distance-dependent weight) respectively

(2) SVM: two parameters are the penalty parameter C and kernel parameter gamma respectively

(3) RF: one parameter is the number of decision trees

(4) GBM: two parameters are the number of decision trees and the learning rate

(5) DNN2 and DNN3: three parameters are number of epochs, the size of mini-batches, and the dropout rate

**Table S3.** All the average classification models by averaging over different data-splitting schemes.

| **Model** | **FS** | **FP** | **method** | **Accuracy**  **(test)** | **Precision**  **(test)** | **Specificity**  **(test)** | **Sensitivity**  **(test)** | **F1-score**  **(test)** | **MCC**  **(test)** | **NER**  **(test)** | **F1-score**  **(CV)** | $\boldsymbol{\Delta}$**F1-score** |
| --- | --- | --- | --- | --- | --- | --- | --- | --- | --- | --- | --- | --- |
| AM01 | full | 1024bit-ECFP4 | KNN | 0.864(0.022) | 0.807(0.041) | 0.871(0.036) | 0.852(0.040) | 0.828(0.026) | 0.718(0.043) | 0.862(0.021) | 0.820(0.009) | 0.028(0.016) |
| AM02 | full | 1024bit-ECFP4 | SVM | 0.889(0.015) | 0.884(0.029) | 0.932(0.019) | 0.819(0.032) | 0.850(0.021) | 0.764(0.032) | 0.876(0.017) | 0.842(0.006) | 0.021(0.012) |
| AM03 | full | 1024bit-ECFP4 | GBM | 0.883(0.013) | 0.883(0.027) | 0.933(0.019) | 0.804(0.034) | 0.841(0.019) | 0.752(0.027) | 0.868(0.015) | 0.833(0.010) | 0.021(0.013) |
| AM04 | full | 1024bit-ECFP4 | RF | 0.891(0.014) | 0.913(0.027) | 0.953(0.016) | 0.792(0.030) | 0.848(0.021) | 0.769(0.031) | 0.872(0.016) | 0.839(0.007) | 0.021(0.015) |
| AM05 | full | 1024bit-ECFP4 | DNN2 | 0.885(0.009) | 0.900(0.018) | 0.945(0.011) | 0.789(0.009) | 0.841(0.012) | 0.756(0.020) | 0.867(0.009) | 0.850(0.004) | 0.009(0.009) |
| AM06 | full | 1024bit-ECFP4 | DNN3 | 0.882(0.005) | 0.890(0.011) | 0.939(0.007) | 0.789(0.012) | 0.837(0.007) | 0.748(0.010) | 0.864(0.005) | 0.849(0.003) | 0.012(0.004) |
| AM07 | full | 2048bit-ECFP4 | KNN | 0.865(0.017) | 0.816(0.037) | 0.880(0.031) | 0.841(0.040) | 0.827(0.021) | 0.718(0.034) | 0.861(0.018) | 0.823(0.010) | 0.024(0.014) |
| AM08 | full | 2048bit-ECFP4 | SVM | 0.891(0.012) | 0.887(0.031) | 0.933(0.022) | 0.824(0.029) | 0.853(0.016) | 0.769(0.025) | 0.879(0.012) | 0.847(0.007) | 0.017(0.013) |
| AM09 | full | 2048bit-ECFP4 | GBM | 0.877(0.014) | 0.876(0.035) | 0.929(0.024) | 0.793(0.023) | 0.832(0.018) | 0.738(0.031) | 0.861(0.013) | 0.827(0.009) | 0.019(0.013) |
| AM10 | full | 2048bit-ECFP4 | RF | 0.891(0.014) | 0.911(0.026) | 0.951(0.016) | 0.795(0.029) | 0.849(0.020) | 0.769(0.030) | 0.873(0.016) | 0.840(0.009) | 0.023(0.013) |
| AM11 | full | 2048bit-ECFP4 | DNN2 | 0.891(0.008) | 0.893(0.006) | 0.939(0.006) | 0.814(0.027) | 0.852(0.013) | 0.769(0.017) | 0.877(0.011) | 0.848(0.002) | 0.012(0.009) |
| AM12 | full | 2048bit-ECFP4 | DNN3 | 0.892(0.020) | 0.890(0.005) | 0.937(0.003) | 0.821(0.053) | 0.853(0.030) | 0.771(0.043) | 0.879(0.026) | 0.777(0.080) | 0.077(0.067) |
| AM13 | full | 1024bit-ECFP6 | KNN | 0.858(0.019) | 0.805(0.035) | 0.872(0.031) | 0.836(0.041) | 0.819(0.023) | 0.704(0.037) | 0.854(0.019) | 0.806(0.010) | 0.023(0.017) |
| AM14 | full | 1024bit-ECFP6 | SVM | 0.878(0.013) | 0.869(0.029) | 0.923(0.023) | 0.805(0.034) | 0.835(0.017) | 0.741(0.026) | 0.864(0.014) | 0.834(0.007) | 0.018(0.012) |
| AM15 | full | 1024bit-ECFP6 | GBM | 0.878(0.013) | 0.883(0.028) | 0.934(0.018) | 0.789(0.027) | 0.833(0.018) | 0.741(0.028) | 0.862(0.014) | 0.827(0.008) | 0.016(0.012) |
| AM16 | full | 1024bit-ECFP6 | RF | 0.888(0.015) | 0.928(0.027) | 0.962(0.015) | 0.768(0.035) | 0.840(0.023) | 0.763(0.032) | 0.865(0.018) | 0.830(0.008) | 0.023(0.017) |
| AM17 | full | 1024bit-ECFP6 | DNN2 | 0.873(0.006) | 0.899(0.012) | 0.947(0.008) | 0.755(0.021) | 0.820(0.011) | 0.730(0.012) | 0.851(0.009) | 0.840(0.003) | 0.020(0.008) |
| AM18 | full | 1024bit-ECFP6 | DNN3 | 0.876(0.004) | 0.897(0.009) | 0.945(0.006) | 0.764(0.013) | 0.825(0.007) | 0.735(0.010) | 0.855(0.006) | 0.840(0.002) | 0.015(0.009) |
| AM19 | full | 2048bit-ECFP6 | KNN | 0.863(0.021) | 0.813(0.047) | 0.876(0.040) | 0.842(0.033) | 0.826(0.023) | 0.715(0.040) | 0.859(0.018) | 0.810(0.009) | 0.022(0.018) |
| AM20 | full | 2048bit-ECFP6 | SVM | 0.892(0.013) | 0.890(0.025) | 0.936(0.018) | 0.820(0.033) | 0.853(0.019) | 0.770(0.028) | 0.878(0.016) | 0.843(0.008) | 0.017(0.019) |
| AM21 | full | 2048bit-ECFP6 | GBM | 0.880(0.014) | 0.887(0.034) | 0.937(0.023) | 0.788(0.023) | 0.834(0.018) | 0.744(0.030) | 0.863(0.013) | 0.821(0.008) | 0.019(0.013) |
| AM22 | full | 2048bit-ECFP6 | RF | 0.889(0.014) | 0.924(0.025) | 0.960(0.014) | 0.776(0.031) | 0.843(0.021) | 0.766(0.029) | 0.868(0.016) | 0.833(0.008) | 0.022(0.016) |
| AM23 | full | 2048bit-ECFP6 | DNN2 | 0.879(0.006) | 0.890(0.023) | 0.939(0.015) | 0.783(0.007) | 0.833(0.006) | 0.743(0.014) | 0.861(0.004) | 0.847(0.002) | 0.014(0.007) |
| AM24 | full | 2048bit-ECFP6 | DNN3 | 0.872(0.003) | 0.929(0.025) | 0.965(0.014) | 0.723(0.031) | 0.813(0.011) | 0.732(0.005) | 0.844(0.008) | 0.793(0.019) | 0.020(0.010) |
| AM25 | 512 | 1024bit-ECFP4 | KNN | 0.872(0.017) | 0.834(0.045) | 0.893(0.040) | 0.840(0.040) | 0.835(0.019) | 0.733(0.033) | 0.866(0.015) | 0.833(0.010) | 0.019(0.014) |
| AM26 | 512 | 1024bit-ECFP4 | SVM | 0.893(0.017) | 0.887(0.032) | 0.933(0.021) | 0.830(0.031) | 0.857(0.023) | 0.774(0.037) | 0.881(0.018) | 0.849(0.006) | 0.026(0.011) |
| AM27 | 512 | 1024bit-ECFP4 | GBM | 0.879(0.014) | 0.885(0.035) | 0.935(0.023) | 0.788(0.032) | 0.833(0.020) | 0.742(0.032) | 0.862(0.015) | 0.834(0.010) | 0.020(0.015) |
| AM28 | 512 | 1024bit-ECFP4 | RF | 0.892(0.015) | 0.917(0.026) | 0.955(0.016) | 0.791(0.033) | 0.849(0.021) | 0.771(0.031) | 0.873(0.017) | 0.841(0.007) | 0.022(0.016) |
| AM29 | 512 | 1024bit-ECFP4 | DNN2 | 0.897(0.006) | 0.904(0.018) | 0.945(0.012) | 0.821(0.021) | 0.860(0.009) | 0.782(0.013) | 0.882(0.008) | 0.842(0.003) | 0.018(0.010) |
| AM30 | 512 | 1024bit-ECFP4 | DNN3 | 0.891(0.008) | 0.891(0.021) | 0.937(0.014) | 0.818(0.005) | 0.853(0.008) | 0.769(0.016) | 0.877(0.006) | 0.848(0.004) | 0.011(0.006) |
| AM31 | 512 | 2048bit-ECFP4 | KNN | 0.874(0.016) | 0.846(0.040) | 0.904(0.032) | 0.826(0.037) | 0.835(0.020) | 0.735(0.033) | 0.865(0.016) | 0.838(0.007) | 0.020(0.012) |
| AM32 | 512 | 2048bit-ECFP4 | SVM | 0.891(0.016) | 0.887(0.029) | 0.934(0.019) | 0.823(0.034) | 0.853(0.023) | 0.769(0.035) | 0.878(0.018) | 0.857(0.006) | 0.023(0.016) |
| AM33 | 512 | 2048bit-ECFP4 | GBM | 0.882(0.017) | 0.888(0.035) | 0.936(0.024) | 0.796(0.032) | 0.839(0.023) | 0.750(0.036) | 0.866(0.017) | 0.838(0.009) | 0.021(0.017) |
| AM34 | 512 | 2048bit-ECFP4 | RF | 0.891(0.014) | 0.917(0.026) | 0.955(0.016) | 0.787(0.029) | 0.847(0.020) | 0.769(0.030) | 0.871(0.015) | 0.846(0.007) | 0.021(0.012) |
| AM35 | 512 | 2048bit-ECFP4 | DNN2 | 0.891(0.005) | 0.908(0.025) | 0.949(0.016) | 0.799(0.019) | 0.850(0.006) | 0.769(0.012) | 0.874(0.005) | 0.857(0.000) | 0.008(0.006) |
| AM36 | 512 | 2048bit-ECFP4 | DNN3 | 0.888(0.006) | 0.898(0.008) | 0.943(0.006) | 0.799(0.017) | 0.845(0.010) | 0.761(0.012) | 0.871(0.007) | 0.854(0.001) | 0.010(0.008) |
| AM37 | 512 | 1024bit-ECFP6 | KNN | 0.867(0.017) | 0.837(0.044) | 0.898(0.038) | 0.818(0.040) | 0.826(0.020) | 0.720(0.032) | 0.858(0.015) | 0.826(0.009) | 0.020(0.013) |
| AM38 | 512 | 1024bit-ECFP6 | SVM | 0.890(0.015) | 0.893(0.033) | 0.938(0.022) | 0.811(0.031) | 0.849(0.020) | 0.766(0.032) | 0.875(0.016) | 0.845(0.006) | 0.017(0.017) |
| AM39 | 512 | 1024bit-ECFP6 | GBM | 0.876(0.014) | 0.894(0.031) | 0.942(0.019) | 0.769(0.034) | 0.826(0.020) | 0.735(0.029) | 0.856(0.016) | 0.827(0.009) | 0.021(0.014) |
| AM40 | 512 | 1024bit-ECFP6 | RF | 0.885(0.013) | 0.924(0.027) | 0.960(0.016) | 0.764(0.033) | 0.835(0.020) | 0.757(0.027) | 0.862(0.015) | 0.834(0.009) | 0.021(0.013) |
| AM41 | 512 | 1024bit-ECFP6 | DNN2 | 0.868(0.021) | 0.875(0.016) | 0.931(0.010) | 0.767(0.056) | 0.816(0.035) | 0.719(0.045) | 0.849(0.027) | 0.828(0.001) | 0.028(0.024) |
| AM42 | 512 | 1024bit-ECFP6 | DNN3 | 0.877(0.020) | 0.883(0.008) | 0.935(0.000) | 0.783(0.053) | 0.829(0.034) | 0.737(0.044) | 0.859(0.026) | 0.834(0.002) | 0.032(0.016) |
| AM43 | 512 | 2048bit-ECFP6 | KNN | 0.876(0.015) | 0.854(0.036) | 0.911(0.027) | 0.819(0.039) | 0.835(0.020) | 0.737(0.031) | 0.865(0.016) | 0.842(0.009) | 0.021(0.012) |
| AM44 | 512 | 2048bit-ECFP6 | SVM | 0.891(0.014) | 0.898(0.029) | 0.942(0.019) | 0.810(0.027) | 0.851(0.019) | 0.769(0.030) | 0.876(0.014) | 0.859(0.008) | 0.021(0.014) |
| AM45 | 512 | 2048bit-ECFP6 | GBM | 0.876(0.015) | 0.890(0.033) | 0.940(0.021) | 0.774(0.024) | 0.827(0.020) | 0.736(0.032) | 0.857(0.015) | 0.831(0.008) | 0.020(0.013) |
| AM46 | 512 | 2048bit-ECFP6 | RF | 0.889(0.016) | 0.924(0.033) | 0.959(0.019) | 0.777(0.031) | 0.843(0.023) | 0.766(0.035) | 0.868(0.017) | 0.844(0.007) | 0.022(0.015) |
| AM47 | 512 | 2048bit-ECFP6 | DNN2 | 0.880(0.005) | 0.904(0.007) | 0.949(0.006) | 0.770(0.019) | 0.832(0.009) | 0.746(0.011) | 0.860(0.008) | 0.847(0.002) | 0.015(0.010) |
| AM48 | 512 | 2048bit-ECFP6 | DNN3 | 0.879(0.005) | 0.884(0.007) | 0.935(0.005) | 0.789(0.012) | 0.834(0.007) | 0.742(0.010) | 0.863(0.005) | 0.852(0.002) | 0.018(0.007) |
| AM49 | 256 | 1024bit-ECFP4 | KNN | 0.875(0.021) | 0.844(0.041) | 0.902(0.033) | 0.831(0.037) | 0.836(0.026) | 0.736(0.043) | 0.867(0.021) | 0.836(0.009) | 0.026(0.018) |
| AM50 | 256 | 1024bit-ECFP4 | SVM | 0.892(0.012) | 0.881(0.026) | 0.929(0.019) | 0.832(0.033) | 0.855(0.017) | 0.771(0.025) | 0.881(0.014) | 0.853(0.006) | 0.014(0.015) |
| AM51 | 256 | 1024bit-ECFP4 | GBM | 0.878(0.018) | 0.877(0.031) | 0.929(0.021) | 0.797(0.036) | 0.834(0.026) | 0.741(0.039) | 0.863(0.020) | 0.838(0.011) | 0.026(0.020) |
| AM52 | 256 | 1024bit-ECFP4 | RF | 0.890(0.012) | 0.906(0.026) | 0.948(0.017) | 0.796(0.033) | 0.847(0.018) | 0.766(0.025) | 0.872(0.014) | 0.841(0.007) | 0.019(0.014) |
| AM53 | 256 | 1024bit-ECFP4 | DNN2 | 0.888(0.010) | 0.893(0.011) | 0.939(0.010) | 0.805(0.043) | 0.846(0.019) | 0.761(0.021) | 0.872(0.017) | 0.847(0.009) | 0.026(0.010) |
| AM54 | 256 | 1024bit-ECFP4 | DNN3 | 0.891(0.015) | 0.901(0.004) | 0.945(0.003) | 0.805(0.043) | 0.850(0.025) | 0.769(0.033) | 0.875(0.020) | 0.848(0.008) | 0.032(0.009) |
| AM55 | 256 | 2048bit-ECFP4 | KNN | 0.872(0.018) | 0.851(0.045) | 0.909(0.033) | 0.814(0.023) | 0.831(0.020) | 0.730(0.036) | 0.861(0.015) | 0.841(0.007) | 0.023(0.012) |
| AM56 | 256 | 2048bit-ECFP4 | SVM | 0.888(0.012) | 0.873(0.028) | 0.924(0.021) | 0.831(0.028) | 0.851(0.016) | 0.763(0.025) | 0.877(0.012) | 0.857(0.009) | 0.018(0.013) |
| AM57 | 256 | 2048bit-ECFP4 | GBM | 0.883(0.016) | 0.879(0.031) | 0.930(0.021) | 0.808(0.034) | 0.841(0.022) | 0.752(0.033) | 0.869(0.017) | 0.848(0.008) | 0.020(0.016) |
| AM58 | 256 | 2048bit-ECFP4 | RF | 0.886(0.012) | 0.895(0.032) | 0.940(0.022) | 0.800(0.031) | 0.844(0.017) | 0.759(0.026) | 0.870(0.013) | 0.845(0.008) | 0.018(0.012) |
| AM59 | 256 | 2048bit-ECFP4 | DNN2 | 0.875(0.010) | 0.864(0.034) | 0.920(0.024) | 0.802(0.016) | 0.831(0.009) | 0.733(0.020) | 0.861(0.006) | 0.858(0.001) | 0.027(0.008) |
| AM60 | 256 | 2048bit-ECFP4 | DNN3 | 0.878(0.003) | 0.866(0.012) | 0.922(0.010) | 0.808(0.025) | 0.836(0.008) | 0.740(0.008) | 0.865(0.007) | 0.857(0.002) | 0.021(0.008) |
| AM61 | 256 | 1024bit-ECFP6 | KNN | 0.868(0.022) | 0.835(0.047) | 0.896(0.038) | 0.823(0.041) | 0.828(0.027) | 0.723(0.045) | 0.860(0.021) | 0.835(0.008) | 0.028(0.016) |
| AM62 | 256 | 1024bit-ECFP6 | SVM | 0.886(0.014) | 0.883(0.029) | 0.932(0.021) | 0.814(0.038) | 0.846(0.021) | 0.759(0.031) | 0.873(0.017) | 0.850(0.007) | 0.021(0.017) |
| AM63 | 256 | 1024bit-ECFP6 | GBM | 0.875(0.018) | 0.884(0.041) | 0.935(0.028) | 0.780(0.041) | 0.827(0.025) | 0.735(0.038) | 0.857(0.019) | 0.829(0.009) | 0.025(0.018) |
| AM64 | 256 | 1024bit-ECFP6 | RF | 0.890(0.016) | 0.920(0.029) | 0.957(0.018) | 0.782(0.040) | 0.845(0.024) | 0.767(0.034) | 0.869(0.019) | 0.839(0.008) | 0.026(0.016) |
| AM65 | 256 | 1024bit-ECFP6 | DNN2 | 0.874(0.015) | 0.872(0.008) | 0.927(0.007) | 0.789(0.045) | 0.828(0.025) | 0.733(0.032) | 0.858(0.020) | 0.838(0.009) | 0.025(0.019) |
| AM66 | 256 | 1024bit-ECFP6 | DNN3 | 0.876(0.016) | 0.888(0.006) | 0.939(0.003) | 0.774(0.043) | 0.826(0.027) | 0.735(0.035) | 0.856(0.021) | 0.834(0.010) | 0.028(0.018) |
| AM67 | 256 | 2048bit-ECFP6 | KNN | 0.878(0.017) | 0.863(0.040) | 0.918(0.028) | 0.816(0.029) | 0.838(0.021) | 0.743(0.035) | 0.867(0.016) | 0.839(0.008) | 0.022(0.013) |
| AM68 | 256 | 2048bit-ECFP6 | SVM | 0.891(0.013) | 0.879(0.028) | 0.928(0.019) | 0.830(0.030) | 0.854(0.018) | 0.768(0.028) | 0.879(0.015) | 0.861(0.010) | 0.022(0.015) |
| AM69 | 256 | 2048bit-ECFP6 | GBM | 0.878(0.015) | 0.878(0.026) | 0.931(0.018) | 0.793(0.038) | 0.833(0.023) | 0.741(0.033) | 0.862(0.018) | 0.841(0.008) | 0.021(0.020) |
| AM70 | 256 | 2048bit-ECFP6 | RF | 0.888(0.016) | 0.912(0.033) | 0.952(0.020) | 0.785(0.030) | 0.843(0.023) | 0.762(0.035) | 0.869(0.017) | 0.846(0.008) | 0.024(0.018) |
| AM71 | 256 | 2048bit-ECFP6 | DNN2 | 0.881(0.007) | 0.884(0.035) | 0.934(0.023) | 0.799(0.017) | 0.838(0.006) | 0.749(0.017) | 0.866(0.003) | 0.852(0.002) | 0.013(0.005) |
| AM72 | 256 | 2048bit-ECFP6 | DNN3 | 0.891(0.011) | 0.902(0.005) | 0.945(0.006) | 0.805(0.036) | 0.850(0.018) | 0.769(0.022) | 0.875(0.015) | 0.854(0.003) | 0.018(0.012) |
| AM73 | 128 | 1024bit-ECFP4 | KNN | 0.872(0.016) | 0.836(0.039) | 0.896(0.032) | 0.833(0.037) | 0.833(0.020) | 0.730(0.033) | 0.864(0.016) | 0.836(0.009) | 0.022(0.014) |
| AM74 | 128 | 1024bit-ECFP4 | SVM | 0.887(0.014) | 0.870(0.029) | 0.922(0.020) | 0.831(0.032) | 0.850(0.019) | 0.761(0.030) | 0.877(0.016) | 0.853(0.008) | 0.021(0.011) |
| AM75 | 128 | 1024bit-ECFP4 | GBM | 0.884(0.019) | 0.869(0.035) | 0.922(0.024) | 0.822(0.037) | 0.844(0.026) | 0.753(0.040) | 0.872(0.020) | 0.843(0.008) | 0.026(0.015) |
| AM76 | 128 | 1024bit-ECFP4 | RF | 0.887(0.015) | 0.892(0.028) | 0.939(0.018) | 0.803(0.032) | 0.845(0.021) | 0.759(0.032) | 0.871(0.016) | 0.842(0.007) | 0.022(0.014) |
| AM77 | 128 | 1024bit-ECFP4 | DNN2 | 0.891(0.021) | 0.877(0.019) | 0.928(0.010) | 0.833(0.040) | 0.855(0.029) | 0.769(0.045) | 0.880(0.024) | 0.839(0.006) | 0.027(0.027) |
| AM78 | 128 | 1024bit-ECFP4 | DNN3 | 0.882(0.010) | 0.862(0.009) | 0.918(0.005) | 0.824(0.024) | 0.842(0.015) | 0.748(0.023) | 0.871(0.013) | 0.840(0.004) | 0.015(0.010) |
| AM79 | 128 | 2048bit-ECFP4 | KNN | 0.877(0.015) | 0.848(0.037) | 0.906(0.030) | 0.831(0.043) | 0.838(0.021) | 0.741(0.032) | 0.868(0.017) | 0.837(0.008) | 0.020(0.018) |
| AM80 | 128 | 2048bit-ECFP4 | SVM | 0.886(0.015) | 0.867(0.035) | 0.919(0.027) | 0.834(0.041) | 0.849(0.021) | 0.760(0.032) | 0.876(0.017) | 0.851(0.008) | 0.021(0.017) |
| AM81 | 128 | 2048bit-ECFP4 | GBM | 0.882(0.018) | 0.867(0.028) | 0.921(0.019) | 0.819(0.041) | 0.842(0.026) | 0.749(0.039) | 0.870(0.021) | 0.848(0.010) | 0.024(0.022) |
| AM82 | 128 | 2048bit-ECFP4 | RF | 0.884(0.019) | 0.880(0.031) | 0.930(0.021) | 0.810(0.039) | 0.843(0.027) | 0.754(0.041) | 0.870(0.022) | 0.845(0.010) | 0.028(0.019) |
| AM83 | 128 | 2048bit-ECFP4 | DNN2 | 0.877(0.010) | 0.867(0.010) | 0.924(0.004) | 0.802(0.020) | 0.833(0.015) | 0.737(0.022) | 0.863(0.012) | 0.848(0.007) | 0.016(0.021) |
| AM84 | 128 | 2048bit-ECFP4 | DNN3 | 0.877(0.022) | 0.858(0.031) | 0.915(0.019) | 0.814(0.032) | 0.835(0.030) | 0.738(0.048) | 0.865(0.023) | 0.848(0.008) | 0.034(0.021) |
| AM85 | 128 | 1024bit-ECFP6 | KNN | 0.867(0.020) | 0.827(0.047) | 0.888(0.040) | 0.831(0.051) | 0.827(0.025) | 0.721(0.041) | 0.860(0.021) | 0.827(0.009) | 0.027(0.014) |
| AM86 | 128 | 1024bit-ECFP6 | SVM | 0.891(0.016) | 0.880(0.033) | 0.929(0.024) | 0.831(0.036) | 0.854(0.021) | 0.769(0.033) | 0.879(0.017) | 0.848(0.009) | 0.024(0.017) |
| AM87 | 128 | 1024bit-ECFP6 | GBM | 0.885(0.013) | 0.879(0.027) | 0.929(0.019) | 0.813(0.034) | 0.844(0.018) | 0.755(0.028) | 0.871(0.015) | 0.840(0.008) | 0.017(0.013) |
| AM88 | 128 | 1024bit-ECFP6 | RF | 0.889(0.015) | 0.907(0.032) | 0.948(0.021) | 0.793(0.040) | 0.845(0.023) | 0.764(0.033) | 0.871(0.018) | 0.837(0.006) | 0.023(0.014) |
| AM89 | 128 | 1024bit-ECFP6 | DNN2 | 0.872(0.016) | 0.852(0.031) | 0.912(0.022) | 0.808(0.024) | 0.829(0.019) | 0.728(0.032) | 0.860(0.015) | 0.834(0.011) | 0.027(0.009) |
| AM90 | 128 | 1024bit-ECFP6 | DNN3 | 0.870(0.008) | 0.858(0.023) | 0.917(0.016) | 0.792(0.008) | 0.824(0.008) | 0.722(0.016) | 0.855(0.005) | 0.836(0.011) | 0.016(0.011) |
| AM91 | 128 | 2048bit-ECFP6 | KNN | 0.873(0.015) | 0.846(0.039) | 0.904(0.032) | 0.824(0.049) | 0.833(0.022) | 0.733(0.031) | 0.864(0.018) | 0.836(0.008) | 0.021(0.013) |
| AM92 | 128 | 2048bit-ECFP6 | SVM | 0.887(0.012) | 0.867(0.030) | 0.919(0.022) | 0.837(0.028) | 0.851(0.015) | 0.761(0.025) | 0.878(0.012) | 0.850(0.008) | 0.016(0.011) |
| AM93 | 128 | 2048bit-ECFP6 | GBM | 0.879(0.019) | 0.871(0.036) | 0.925(0.026) | 0.806(0.043) | 0.836(0.027) | 0.743(0.041) | 0.865(0.021) | 0.839(0.009) | 0.026(0.019) |
| AM94 | 128 | 2048bit-ECFP6 | RF | 0.888(0.019) | 0.890(0.031) | 0.937(0.020) | 0.809(0.038) | 0.847(0.027) | 0.762(0.040) | 0.873(0.021) | 0.844(0.010) | 0.026(0.018) |
| AM95 | 128 | 2048bit-ECFP6 | DNN2 | 0.872(0.006) | 0.847(0.015) | 0.908(0.012) | 0.815(0.024) | 0.830(0.010) | 0.728(0.013) | 0.861(0.008) | 0.839(0.004) | 0.009(0.006) |
| AM96 | 128 | 2048bit-ECFP6 | DNN3 | 0.868(0.019) | 0.840(0.024) | 0.904(0.014) | 0.811(0.034) | 0.825(0.027) | 0.720(0.041) | 0.858(0.022) | 0.839(0.005) | 0.025(0.013) |

**Table S4.** Evaluation metrics for **Dataset-CV** with the randomly shuffled labels before Y-randomization test of classification models

| **Exp** | **TP** | **TN** | **FP** | **FN** | **Accuracy** | **Precision** | **Specificity** | **Sensitivity** | **F1-score** | **MCC** |
| --- | --- | --- | --- | --- | --- | --- | --- | --- | --- | --- |
| exp01 | 169 | 425 | 255 | 255 | 0.538 | 0.399 | 0.625 | 0.399 | 0.399 | 0.024 |
| exp02 | 158 | 414 | 266 | 266 | 0.518 | 0.373 | 0.609 | 0.373 | 0.373 | -0.019 |
| exp03 | 157 | 413 | 267 | 267 | 0.516 | 0.370 | 0.607 | 0.370 | 0.370 | -0.022 |
| exp04 | 157 | 413 | 267 | 267 | 0.516 | 0.370 | 0.607 | 0.370 | 0.370 | -0.022 |
| exp05 | 162 | 418 | 262 | 262 | 0.525 | 0.382 | 0.615 | 0.382 | 0.382 | -0.003 |
| exp06 | 165 | 421 | 259 | 259 | 0.531 | 0.389 | 0.619 | 0.389 | 0.389 | 0.008 |
| exp07 | 171 | 427 | 253 | 253 | 0.542 | 0.403 | 0.628 | 0.403 | 0.403 | 0.031 |
| exp08 | 169 | 425 | 255 | 255 | 0.538 | 0.399 | 0.625 | 0.399 | 0.399 | 0.024 |
| exp09 | 181 | 437 | 243 | 243 | 0.56 | 0.427 | 0.643 | 0.427 | 0.427 | 0.070 |
| exp10 | 166 | 422 | 258 | 258 | 0.533 | 0.392 | 0.621 | 0.392 | 0.392 | 0.012 |
| exp11 | 164 | 420 | 260 | 260 | 0.529 | 0.387 | 0.618 | 0.387 | 0.387 | 0.004 |
| exp12 | 168 | 424 | 256 | 256 | 0.536 | 0.396 | 0.624 | 0.396 | 0.396 | 0.020 |
| exp13 | 155 | 411 | 269 | 269 | 0.513 | 0.366 | 0.604 | 0.366 | 0.366 | -0.030 |
| exp14 | 173 | 429 | 251 | 251 | 0.545 | 0.408 | 0.631 | 0.408 | 0.408 | 0.039 |
| exp15 | 154 | 410 | 270 | 270 | 0.511 | 0.363 | 0.603 | 0.363 | 0.363 | -0.034 |
| exp16 | 167 | 423 | 257 | 257 | 0.534 | 0.394 | 0.622 | 0.394 | 0.394 | 0.016 |
| exp17 | 154 | 410 | 270 | 270 | 0.511 | 0.363 | 0.603 | 0.363 | 0.363 | -0.034 |
| exp18 | 155 | 411 | 269 | 269 | 0.513 | 0.366 | 0.604 | 0.366 | 0.366 | -0.030 |
| exp19 | 160 | 416 | 264 | 264 | 0.522 | 0.377 | 0.612 | 0.377 | 0.377 | -0.011 |

Note: “Exp” refers to the random data-splitting scheme.

**Table S5.** All the individual classification models after Y-randomization test

| **Model** | **FS** | **EXP** | **FP** | **Method** | **Accuracy**  **(test)** | **Precision (test)** | **Specificity**  **(test)** | **Sensitivity**  **(test)** | **F1-score**  **(test)** | **MCC**  **(test)** | **NER**  **(test)** | **F1-score**  **(CV)** | **Parameters from CV*** |
| --- | --- | --- | --- | --- | --- | --- | --- | --- | --- | --- | --- | --- | --- |
| M0001 | full | exp01 | 1024bit-ECFP4 | KNN | 0.507 | 0.356 | 0.606 | 0.349 | 0.352 | -0.045 | 0.477 | 0.385 | 1 uniform |
| M0002 | full | exp02 | 1024bit-ECFP4 | KNN | 0.507 | 0.347 | 0.624 | 0.321 | 0.333 | -0.057 | 0.473 | 0.342 | 1 uniform |
| M0003 | full | exp03 | 1024bit-ECFP4 | KNN | 0.533 | 0.365 | 0.682 | 0.292 | 0.325 | -0.027 | 0.487 | 0.389 | 3 distance |
| M0004 | full | exp04 | 1024bit-ECFP4 | KNN | 0.551 | 0.413 | 0.641 | 0.406 | 0.410 | 0.047 | 0.524 | 0.409 | 1 uniform |
| M0005 | full | exp05 | 1024bit-ECFP4 | KNN | 0.533 | 0.393 | 0.618 | 0.396 | 0.394 | 0.014 | 0.507 | 0.379 | 1 uniform |
| M0006 | full | exp06 | 1024bit-ECFP4 | KNN | 0.551 | 0.427 | 0.582 | 0.500 | 0.461 | 0.081 | 0.541 | 0.369 | 1 uniform |
| M0007 | full | exp07 | 1024bit-ECFP4 | KNN | 0.482 | 0.354 | 0.518 | 0.425 | 0.386 | -0.056 | 0.472 | 0.386 | 1 uniform |
| M0008 | full | exp08 | 1024bit-ECFP4 | KNN | 0.500 | 0.352 | 0.588 | 0.358 | 0.355 | -0.053 | 0.473 | 0.368 | 1 uniform |
| M0009 | full | exp09 | 1024bit-ECFP4 | KNN | 0.576 | 0.450 | 0.647 | 0.462 | 0.456 | 0.109 | 0.554 | 0.439 | 1 uniform |
| M0010 | full | exp10 | 1024bit-ECFP4 | KNN | 0.482 | 0.333 | 0.565 | 0.349 | 0.341 | -0.086 | 0.457 | 0.409 | 1 uniform |
| M0011 | full | exp11 | 1024bit-ECFP4 | KNN | 0.482 | 0.350 | 0.529 | 0.406 | 0.376 | -0.064 | 0.468 | 0.428 | 1 uniform |
| M0012 | full | exp12 | 1024bit-ECFP4 | KNN | 0.525 | 0.379 | 0.624 | 0.368 | 0.373 | -0.009 | 0.496 | 0.404 | 1 uniform |
| M0013 | full | exp13 | 1024bit-ECFP4 | KNN | 0.536 | 0.380 | 0.665 | 0.330 | 0.354 | -0.005 | 0.498 | 0.372 | 1 uniform |
| M0014 | full | exp14 | 1024bit-ECFP4 | KNN | 0.569 | 0.438 | 0.653 | 0.434 | 0.436 | 0.087 | 0.543 | 0.376 | 1 uniform |
| M0015 | full | exp15 | 1024bit-ECFP4 | KNN | 0.518 | 0.345 | 0.665 | 0.283 | 0.311 | -0.055 | 0.474 | 0.358 | 3 uniform |
| M0016 | full | exp16 | 1024bit-ECFP4 | KNN | 0.533 | 0.381 | 0.647 | 0.349 | 0.365 | -0.004 | 0.498 | 0.415 | 1 uniform |
| M0017 | full | exp17 | 1024bit-ECFP4 | KNN | 0.536 | 0.394 | 0.629 | 0.387 | 0.390 | 0.016 | 0.508 | 0.393 | 1 uniform |
| M0018 | full | exp18 | 1024bit-ECFP4 | KNN | 0.478 | 0.336 | 0.547 | 0.368 | 0.351 | -0.084 | 0.458 | 0.434 | 1 uniform |
| M0019 | full | exp19 | 1024bit-ECFP4 | KNN | 0.565 | 0.426 | 0.682 | 0.377 | 0.400 | 0.061 | 0.530 | 0.382 | 1 uniform |
| M0020 | full | exp01 | 1024bit-ECFP4 | SVM | 0.616 | 0.000 | 1.000 | 0.000 | 0.000 | 0.000 | 0.500 | 0.013 | 10 0.1 |
| M0021 | full | exp02 | 1024bit-ECFP4 | SVM | 0.620 | 1.000 | 1.000 | 0.009 | 0.019 | 0.076 | 0.504 | 0.025 | 100000.0 0.0001 |
| M0022 | full | exp03 | 1024bit-ECFP4 | SVM | 0.616 | 0.000 | 1.000 | 0.000 | 0.000 | 0.000 | 0.500 | 0.000 | 5 5e-05 |
| M0023 | full | exp04 | 1024bit-ECFP4 | SVM | 0.616 | 0.000 | 1.000 | 0.000 | 0.000 | 0.000 | 0.500 | 0.005 | 100000.0 0.1 |
| M0024 | full | exp05 | 1024bit-ECFP4 | SVM | 0.616 | 0.000 | 1.000 | 0.000 | 0.000 | 0.000 | 0.500 | 0.000 | 1 0.005 |
| M0025 | full | exp06 | 1024bit-ECFP4 | SVM | 0.616 | 0.000 | 1.000 | 0.000 | 0.000 | 0.000 | 0.500 | 0.009 | 5000.0 0.0005 |
| M0026 | full | exp07 | 1024bit-ECFP4 | SVM | 0.616 | 0.000 | 1.000 | 0.000 | 0.000 | 0.000 | 0.500 | 0.028 | 10 0.1 |
| M0027 | full | exp08 | 1024bit-ECFP4 | SVM | 0.616 | 0.000 | 1.000 | 0.000 | 0.000 | 0.000 | 0.500 | 0.000 | 50000.0 0.0001 |
| M0028 | full | exp09 | 1024bit-ECFP4 | SVM | 0.616 | 0.000 | 1.000 | 0.000 | 0.000 | 0.000 | 0.500 | 0.005 | 500 1e-05 |
| M0029 | full | exp10 | 1024bit-ECFP4 | SVM | 0.616 | 0.000 | 1.000 | 0.000 | 0.000 | 0.000 | 0.500 | 0.017 | 5 0.1 |
| M0030 | full | exp11 | 1024bit-ECFP4 | SVM | 0.616 | 0.000 | 1.000 | 0.000 | 0.000 | 0.000 | 0.500 | 0.033 | 10 0.1 |
| M0031 | full | exp12 | 1024bit-ECFP4 | SVM | 0.616 | 0.000 | 1.000 | 0.000 | 0.000 | 0.000 | 0.500 | 0.000 | 500 0.1 |
| M0032 | full | exp13 | 1024bit-ECFP4 | SVM | 0.616 | 0.000 | 1.000 | 0.000 | 0.000 | 0.000 | 0.500 | 0.000 | 500 0.01 |
| M0033 | full | exp14 | 1024bit-ECFP4 | SVM | 0.616 | 0.000 | 1.000 | 0.000 | 0.000 | 0.000 | 0.500 | 0.000 | 100 0.0005 |
| M0034 | full | exp15 | 1024bit-ECFP4 | SVM | 0.616 | 0.000 | 1.000 | 0.000 | 0.000 | 0.000 | 0.500 | 0.000 | 5000.0 0.0005 |
| M0035 | full | exp16 | 1024bit-ECFP4 | SVM | 0.649 | 1.000 | 1.000 | 0.085 | 0.157 | 0.233 | 0.542 | 0.103 | 1 0.05 |
| M0036 | full | exp17 | 1024bit-ECFP4 | SVM | 0.616 | 0.000 | 1.000 | 0.000 | 0.000 | 0.000 | 0.500 | 0.000 | 100000.0 0.0001 |
| M0037 | full | exp18 | 1024bit-ECFP4 | SVM | 0.616 | 0.000 | 1.000 | 0.000 | 0.000 | 0.000 | 0.500 | 0.025 | 50000.0 0.1 |
| M0038 | full | exp19 | 1024bit-ECFP4 | SVM | 0.616 | 0.000 | 1.000 | 0.000 | 0.000 | 0.000 | 0.500 | 0.000 | 1000.0 5e-05 |
| M0039 | full | exp01 | 1024bit-ECFP4 | GBM | 0.558 | 0.409 | 0.694 | 0.340 | 0.371 | 0.035 | 0.517 | 0.373 | 100 0.6 |
| M0040 | full | exp02 | 1024bit-ECFP4 | GBM | 0.540 | 0.376 | 0.688 | 0.302 | 0.335 | -0.010 | 0.495 | 0.332 | 200 0.8 |
| M0041 | full | exp03 | 1024bit-ECFP4 | GBM | 0.525 | 0.324 | 0.718 | 0.217 | 0.260 | -0.073 | 0.467 | 0.398 | 50 0.6 |
| M0042 | full | exp04 | 1024bit-ECFP4 | GBM | 0.551 | 0.388 | 0.712 | 0.292 | 0.333 | 0.005 | 0.502 | 0.365 | 1000 0.3 |
| M0043 | full | exp05 | 1024bit-ECFP4 | GBM | 0.547 | 0.388 | 0.694 | 0.311 | 0.346 | 0.006 | 0.502 | 0.380 | 1000 0.2 |
| M0044 | full | exp06 | 1024bit-ECFP4 | GBM | 0.554 | 0.404 | 0.688 | 0.340 | 0.369 | 0.029 | 0.514 | 0.373 | 600 0.2 |
| M0045 | full | exp07 | 1024bit-ECFP4 | GBM | 0.583 | 0.448 | 0.718 | 0.368 | 0.404 | 0.090 | 0.543 | 0.368 | 200 0.6 |
| M0046 | full | exp08 | 1024bit-ECFP4 | GBM | 0.547 | 0.386 | 0.700 | 0.302 | 0.339 | 0.002 | 0.501 | 0.366 | 400 0.5 |
| M0047 | full | exp09 | 1024bit-ECFP4 | GBM | 0.565 | 0.429 | 0.671 | 0.396 | 0.412 | 0.068 | 0.534 | 0.417 | 900 0.5 |
| M0048 | full | exp10 | 1024bit-ECFP4 | GBM | 0.583 | 0.448 | 0.718 | 0.368 | 0.404 | 0.090 | 0.543 | 0.396 | 1000 0.2 |
| M0049 | full | exp11 | 1024bit-ECFP4 | GBM | 0.551 | 0.388 | 0.712 | 0.292 | 0.333 | 0.005 | 0.502 | 0.388 | 1000 0.2 |
| M0050 | full | exp12 | 1024bit-ECFP4 | GBM | 0.591 | 0.458 | 0.735 | 0.358 | 0.402 | 0.099 | 0.546 | 0.389 | 1000 0.8 |
| M0051 | full | exp13 | 1024bit-ECFP4 | GBM | 0.507 | 0.330 | 0.653 | 0.274 | 0.299 | -0.077 | 0.464 | 0.407 | 200 0.7 |
| M0052 | full | exp14 | 1024bit-ECFP4 | GBM | 0.562 | 0.423 | 0.671 | 0.387 | 0.404 | 0.058 | 0.529 | 0.411 | 400 0.9 |
| M0053 | full | exp15 | 1024bit-ECFP4 | GBM | 0.536 | 0.388 | 0.647 | 0.358 | 0.373 | 0.006 | 0.502 | 0.352 | 700 0.4 |
| M0054 | full | exp16 | 1024bit-ECFP4 | GBM | 0.562 | 0.410 | 0.712 | 0.321 | 0.360 | 0.034 | 0.516 | 0.359 | 700 0.6 |
| M0055 | full | exp17 | 1024bit-ECFP4 | GBM | 0.536 | 0.366 | 0.694 | 0.283 | 0.319 | -0.024 | 0.488 | 0.367 | 400 0.5 |
| M0056 | full | exp18 | 1024bit-ECFP4 | GBM | 0.518 | 0.355 | 0.647 | 0.311 | 0.332 | -0.043 | 0.479 | 0.394 | 1000 0.6 |
| M0057 | full | exp19 | 1024bit-ECFP4 | GBM | 0.569 | 0.418 | 0.729 | 0.311 | 0.357 | 0.044 | 0.520 | 0.386 | 100 0.7 |
| M0058 | full | exp01 | 1024bit-ECFP4 | RF | 0.500 | 0.340 | 0.612 | 0.321 | 0.330 | -0.068 | 0.467 | 0.382 | 10 |
| M0059 | full | exp02 | 1024bit-ECFP4 | RF | 0.464 | 0.290 | 0.582 | 0.274 | 0.282 | -0.146 | 0.428 | 0.343 | 10 |
| M0060 | full | exp03 | 1024bit-ECFP4 | RF | 0.522 | 0.356 | 0.659 | 0.302 | 0.327 | -0.041 | 0.481 | 0.410 | 10 |
| M0061 | full | exp04 | 1024bit-ECFP4 | RF | 0.496 | 0.337 | 0.606 | 0.321 | 0.329 | -0.074 | 0.464 | 0.406 | 10 |
| M0062 | full | exp05 | 1024bit-ECFP4 | RF | 0.551 | 0.385 | 0.718 | 0.283 | 0.326 | 0.001 | 0.500 | 0.372 | 10 |
| M0063 | full | exp06 | 1024bit-ECFP4 | RF | 0.540 | 0.389 | 0.659 | 0.349 | 0.368 | 0.008 | 0.504 | 0.357 | 10 |
| M0064 | full | exp07 | 1024bit-ECFP4 | RF | 0.540 | 0.387 | 0.665 | 0.340 | 0.362 | 0.004 | 0.503 | 0.375 | 10 |
| M0065 | full | exp08 | 1024bit-ECFP4 | RF | 0.504 | 0.322 | 0.653 | 0.264 | 0.290 | -0.087 | 0.459 | 0.360 | 10 |
| M0066 | full | exp09 | 1024bit-ECFP4 | RF | 0.565 | 0.436 | 0.635 | 0.453 | 0.444 | 0.088 | 0.544 | 0.407 | 10 |
| M0067 | full | exp10 | 1024bit-ECFP4 | RF | 0.522 | 0.356 | 0.659 | 0.302 | 0.327 | -0.041 | 0.481 | 0.355 | 10 |
| M0068 | full | exp11 | 1024bit-ECFP4 | RF | 0.525 | 0.363 | 0.659 | 0.311 | 0.335 | -0.031 | 0.485 | 0.397 | 10 |
| M0069 | full | exp12 | 1024bit-ECFP4 | RF | 0.609 | 0.489 | 0.724 | 0.425 | 0.455 | 0.153 | 0.575 | 0.377 | 10 |
| M0070 | full | exp13 | 1024bit-ECFP4 | RF | 0.558 | 0.400 | 0.718 | 0.302 | 0.344 | 0.021 | 0.510 | 0.373 | 10 |
| M0071 | full | exp14 | 1024bit-ECFP4 | RF | 0.558 | 0.420 | 0.659 | 0.396 | 0.408 | 0.056 | 0.528 | 0.403 | 10 |
| M0072 | full | exp15 | 1024bit-ECFP4 | RF | 0.547 | 0.396 | 0.676 | 0.340 | 0.365 | 0.017 | 0.508 | 0.371 | 10 |
| M0073 | full | exp16 | 1024bit-ECFP4 | RF | 0.543 | 0.386 | 0.682 | 0.321 | 0.351 | 0.003 | 0.502 | 0.352 | 10 |
| M0074 | full | exp17 | 1024bit-ECFP4 | RF | 0.496 | 0.315 | 0.641 | 0.264 | 0.287 | -0.099 | 0.453 | 0.336 | 10 |
| M0075 | full | exp18 | 1024bit-ECFP4 | RF | 0.504 | 0.340 | 0.624 | 0.311 | 0.325 | -0.066 | 0.468 | 0.367 | 10 |
| M0076 | full | exp19 | 1024bit-ECFP4 | RF | 0.569 | 0.424 | 0.712 | 0.340 | 0.377 | 0.054 | 0.526 | 0.406 | 10 |
| M0077 | full | exp01 | 1024bit-ECFP4 | DNN2 | 0.572 | 0.443 | 0.653 | 0.443 | 0.443 | 0.096 | 0.548 | 0.380 | 200 80 0.3 |
| M0078 | full | exp02 | 1024bit-ECFP4 | DNN2 | 0.558 | 0.400 | 0.718 | 0.302 | 0.344 | 0.021 | 0.510 | 0.346 | 300 100 0.4 |
| M0079 | full | exp03 | 1024bit-ECFP4 | DNN2 | 0.533 | 0.388 | 0.629 | 0.377 | 0.383 | 0.007 | 0.503 | 0.427 | 500 80 0.1 |
| M0080 | full | exp01 | 1024bit-ECFP4 | DNN3 | 0.576 | 0.444 | 0.676 | 0.415 | 0.429 | 0.093 | 0.545 | 0.379 | 600 120 0.4 |
| M0081 | full | exp02 | 1024bit-ECFP4 | DNN3 | 0.511 | 0.369 | 0.588 | 0.387 | 0.378 | -0.025 | 0.487 | 0.372 | 200 160 0.5 |
| M0082 | full | exp03 | 1024bit-ECFP4 | DNN3 | 0.547 | 0.410 | 0.635 | 0.406 | 0.408 | 0.041 | 0.520 | 0.420 | 200 120 0.3 |
| M0083 | full | exp01 | 2048bit-ECFP4 | KNN | 0.500 | 0.343 | 0.606 | 0.330 | 0.337 | -0.064 | 0.468 | 0.392 | 1 uniform |
| M0084 | full | exp02 | 2048bit-ECFP4 | KNN | 0.500 | 0.343 | 0.606 | 0.330 | 0.337 | -0.064 | 0.468 | 0.354 | 1 uniform |
| M0085 | full | exp03 | 2048bit-ECFP4 | KNN | 0.576 | 0.432 | 0.729 | 0.330 | 0.374 | 0.064 | 0.529 | 0.391 | 3 distance |
| M0086 | full | exp04 | 2048bit-ECFP4 | KNN | 0.518 | 0.381 | 0.588 | 0.406 | 0.393 | -0.006 | 0.497 | 0.393 | 1 uniform |
| M0087 | full | exp05 | 2048bit-ECFP4 | KNN | 0.518 | 0.374 | 0.606 | 0.377 | 0.376 | -0.017 | 0.491 | 0.385 | 1 uniform |
| M0088 | full | exp06 | 2048bit-ECFP4 | KNN | 0.551 | 0.425 | 0.594 | 0.481 | 0.451 | 0.074 | 0.537 | 0.370 | 1 uniform |
| M0089 | full | exp07 | 2048bit-ECFP4 | KNN | 0.486 | 0.357 | 0.524 | 0.425 | 0.388 | -0.051 | 0.475 | 0.402 | 1 uniform |
| M0090 | full | exp08 | 2048bit-ECFP4 | KNN | 0.504 | 0.350 | 0.606 | 0.340 | 0.344 | -0.055 | 0.473 | 0.362 | 1 uniform |
| M0091 | full | exp09 | 2048bit-ECFP4 | KNN | 0.565 | 0.439 | 0.624 | 0.472 | 0.455 | 0.094 | 0.548 | 0.425 | 1 uniform |
| M0092 | full | exp10 | 2048bit-ECFP4 | KNN | 0.493 | 0.340 | 0.588 | 0.340 | 0.340 | -0.072 | 0.464 | 0.402 | 1 uniform |
| M0093 | full | exp11 | 2048bit-ECFP4 | KNN | 0.486 | 0.336 | 0.571 | 0.349 | 0.343 | -0.080 | 0.460 | 0.436 | 3 uniform |
| M0094 | full | exp12 | 2048bit-ECFP4 | KNN | 0.518 | 0.366 | 0.624 | 0.349 | 0.357 | -0.028 | 0.486 | 0.403 | 1 uniform |
| M0095 | full | exp13 | 2048bit-ECFP4 | KNN | 0.529 | 0.375 | 0.647 | 0.340 | 0.356 | -0.014 | 0.494 | 0.379 | 1 uniform |
| M0096 | full | exp14 | 2048bit-ECFP4 | KNN | 0.583 | 0.457 | 0.665 | 0.453 | 0.455 | 0.118 | 0.559 | 0.392 | 1 uniform |
| M0097 | full | exp15 | 2048bit-ECFP4 | KNN | 0.514 | 0.363 | 0.618 | 0.349 | 0.356 | -0.034 | 0.483 | 0.368 | 1 uniform |
| M0098 | full | exp16 | 2048bit-ECFP4 | KNN | 0.518 | 0.361 | 0.635 | 0.330 | 0.345 | -0.035 | 0.483 | 0.393 | 1 uniform |
| M0099 | full | exp17 | 2048bit-ECFP4 | KNN | 0.554 | 0.414 | 0.659 | 0.387 | 0.400 | 0.046 | 0.523 | 0.400 | 1 uniform |
| M0100 | full | exp18 | 2048bit-ECFP4 | KNN | 0.482 | 0.342 | 0.547 | 0.377 | 0.359 | -0.074 | 0.462 | 0.433 | 1 uniform |
| M0101 | full | exp19 | 2048bit-ECFP4 | KNN | 0.580 | 0.446 | 0.700 | 0.387 | 0.414 | 0.090 | 0.543 | 0.392 | 1 uniform |
| M0102 | full | exp01 | 2048bit-ECFP4 | SVM | 0.616 | 0.000 | 1.000 | 0.000 | 0.000 | 0.000 | 0.500 | 0.005 | 100000.0 0.05 |
| M0103 | full | exp02 | 2048bit-ECFP4 | SVM | 0.620 | 1.000 | 1.000 | 0.009 | 0.019 | 0.076 | 0.504 | 0.019 | 1000.0 0.1 |
| M0104 | full | exp03 | 2048bit-ECFP4 | SVM | 0.616 | 0.000 | 1.000 | 0.000 | 0.000 | 0.000 | 0.500 | 0.000 | 500 0.1 |
| M0105 | full | exp04 | 2048bit-ECFP4 | SVM | 0.616 | 0.000 | 1.000 | 0.000 | 0.000 | 0.000 | 0.500 | 0.000 | 50000.0 0.0001 |
| M0106 | full | exp05 | 2048bit-ECFP4 | SVM | 0.616 | 0.000 | 1.000 | 0.000 | 0.000 | 0.000 | 0.500 | 0.005 | 5000.0 0.1 |
| M0107 | full | exp06 | 2048bit-ECFP4 | SVM | 0.616 | 0.000 | 1.000 | 0.000 | 0.000 | 0.000 | 0.500 | 0.000 | 5 0.1 |
| M0108 | full | exp07 | 2048bit-ECFP4 | SVM | 0.616 | 0.000 | 1.000 | 0.000 | 0.000 | 0.000 | 0.500 | 0.020 | 10 0.1 |
| M0109 | full | exp08 | 2048bit-ECFP4 | SVM | 0.616 | 0.000 | 1.000 | 0.000 | 0.000 | 0.000 | 0.500 | 0.009 | 100000.0 0.1 |
| M0110 | full | exp09 | 2048bit-ECFP4 | SVM | 0.616 | 0.000 | 1.000 | 0.000 | 0.000 | 0.000 | 0.500 | 0.000 | 500 0.1 |
| M0111 | full | exp10 | 2048bit-ECFP4 | SVM | 0.616 | 0.000 | 1.000 | 0.000 | 0.000 | 0.000 | 0.500 | 0.004 | 10 0.1 |
| M0112 | full | exp11 | 2048bit-ECFP4 | SVM | 0.616 | 0.000 | 1.000 | 0.000 | 0.000 | 0.000 | 0.500 | 0.025 | 5000.0 0.1 |
| M0113 | full | exp12 | 2048bit-ECFP4 | SVM | 0.616 | 0.000 | 1.000 | 0.000 | 0.000 | 0.000 | 0.500 | 0.000 | 5000.0 0.0005 |
| M0114 | full | exp13 | 2048bit-ECFP4 | SVM | 0.616 | 0.000 | 1.000 | 0.000 | 0.000 | 0.000 | 0.500 | 0.000 | 1000.0 0.0001 |
| M0115 | full | exp14 | 2048bit-ECFP4 | SVM | 0.616 | 0.000 | 1.000 | 0.000 | 0.000 | 0.000 | 0.500 | 0.000 | 1 0.01 |
| M0116 | full | exp15 | 2048bit-ECFP4 | SVM | 0.616 | 0.000 | 1.000 | 0.000 | 0.000 | 0.000 | 0.500 | 0.000 | 1000.0 0.1 |
| M0117 | full | exp16 | 2048bit-ECFP4 | SVM | 0.616 | 0.500 | 0.988 | 0.019 | 0.036 | 0.029 | 0.503 | 0.013 | 100 0.0005 |
| M0118 | full | exp17 | 2048bit-ECFP4 | SVM | 0.616 | 0.000 | 1.000 | 0.000 | 0.000 | 0.000 | 0.500 | 0.000 | 50000.0 0.0005 |
| M0119 | full | exp18 | 2048bit-ECFP4 | SVM | 0.616 | 0.000 | 1.000 | 0.000 | 0.000 | 0.000 | 0.500 | 0.017 | 5000.0 0.1 |
| M0120 | full | exp19 | 2048bit-ECFP4 | SVM | 0.616 | 0.000 | 1.000 | 0.000 | 0.000 | 0.000 | 0.500 | 0.012 | 100 0.1 |
| M0121 | full | exp01 | 2048bit-ECFP4 | GBM | 0.522 | 0.356 | 0.659 | 0.302 | 0.327 | -0.041 | 0.481 | 0.363 | 300 0.5 |
| M0122 | full | exp02 | 2048bit-ECFP4 | GBM | 0.547 | 0.393 | 0.682 | 0.330 | 0.359 | 0.013 | 0.506 | 0.329 | 200 0.8 |
| M0123 | full | exp03 | 2048bit-ECFP4 | GBM | 0.518 | 0.333 | 0.682 | 0.255 | 0.289 | -0.067 | 0.469 | 0.399 | 200 0.7 |
| M0124 | full | exp04 | 2048bit-ECFP4 | GBM | 0.511 | 0.321 | 0.676 | 0.245 | 0.278 | -0.084 | 0.461 | 0.361 | 700 0.7 |
| M0125 | full | exp05 | 2048bit-ECFP4 | GBM | 0.518 | 0.333 | 0.682 | 0.255 | 0.289 | -0.067 | 0.469 | 0.375 | 400 0.8 |
| M0126 | full | exp06 | 2048bit-ECFP4 | GBM | 0.562 | 0.414 | 0.700 | 0.340 | 0.373 | 0.041 | 0.520 | 0.379 | 500 0.4 |
| M0127 | full | exp07 | 2048bit-ECFP4 | GBM | 0.601 | 0.474 | 0.759 | 0.349 | 0.402 | 0.117 | 0.554 | 0.344 | 900 0.7 |
| M0128 | full | exp08 | 2048bit-ECFP4 | GBM | 0.536 | 0.355 | 0.712 | 0.255 | 0.297 | -0.036 | 0.483 | 0.379 | 100 0.6 |
| M0129 | full | exp09 | 2048bit-ECFP4 | GBM | 0.583 | 0.453 | 0.694 | 0.406 | 0.428 | 0.102 | 0.550 | 0.392 | 800 0.6 |
| M0130 | full | exp10 | 2048bit-ECFP4 | GBM | 0.511 | 0.354 | 0.624 | 0.330 | 0.341 | -0.047 | 0.477 | 0.403 | 700 0.9 |
| M0131 | full | exp11 | 2048bit-ECFP4 | GBM | 0.511 | 0.356 | 0.618 | 0.340 | 0.348 | -0.043 | 0.479 | 0.425 | 500 0.5 |
| M0132 | full | exp12 | 2048bit-ECFP4 | GBM | 0.580 | 0.448 | 0.688 | 0.406 | 0.426 | 0.096 | 0.547 | 0.381 | 700 0.8 |
| M0133 | full | exp13 | 2048bit-ECFP4 | GBM | 0.504 | 0.318 | 0.659 | 0.255 | 0.283 | -0.091 | 0.457 | 0.434 | 700 0.2 |
| M0134 | full | exp14 | 2048bit-ECFP4 | GBM | 0.551 | 0.404 | 0.671 | 0.358 | 0.380 | 0.030 | 0.514 | 0.405 | 700 0.5 |
| M0135 | full | exp15 | 2048bit-ECFP4 | GBM | 0.547 | 0.393 | 0.682 | 0.330 | 0.359 | 0.013 | 0.506 | 0.373 | 300 0.7 |
| M0136 | full | exp16 | 2048bit-ECFP4 | GBM | 0.598 | 0.471 | 0.735 | 0.377 | 0.419 | 0.119 | 0.556 | 0.354 | 900 0.9 |
| M0137 | full | exp17 | 2048bit-ECFP4 | GBM | 0.511 | 0.333 | 0.659 | 0.274 | 0.301 | -0.071 | 0.467 | 0.377 | 800 0.5 |
| M0138 | full | exp18 | 2048bit-ECFP4 | GBM | 0.507 | 0.333 | 0.647 | 0.283 | 0.306 | -0.073 | 0.465 | 0.395 | 500 0.5 |
| M0139 | full | exp19 | 2048bit-ECFP4 | GBM | 0.551 | 0.398 | 0.688 | 0.330 | 0.361 | 0.019 | 0.509 | 0.388 | 300 0.7 |
| M0140 | full | exp01 | 2048bit-ECFP4 | RF | 0.507 | 0.337 | 0.641 | 0.292 | 0.313 | -0.068 | 0.467 | 0.359 | 10 |
| M0141 | full | exp02 | 2048bit-ECFP4 | RF | 0.529 | 0.364 | 0.671 | 0.302 | 0.330 | -0.029 | 0.487 | 0.331 | 10 |
| M0142 | full | exp03 | 2048bit-ECFP4 | RF | 0.486 | 0.291 | 0.641 | 0.236 | 0.260 | -0.129 | 0.439 | 0.414 | 10 |
| M0143 | full | exp04 | 2048bit-ECFP4 | RF | 0.504 | 0.343 | 0.618 | 0.321 | 0.332 | -0.062 | 0.470 | 0.353 | 10 |
| M0144 | full | exp05 | 2048bit-ECFP4 | RF | 0.518 | 0.337 | 0.676 | 0.264 | 0.296 | -0.063 | 0.470 | 0.386 | 10 |
| M0145 | full | exp06 | 2048bit-ECFP4 | RF | 0.583 | 0.448 | 0.718 | 0.368 | 0.404 | 0.090 | 0.543 | 0.343 | 10 |
| M0146 | full | exp07 | 2048bit-ECFP4 | RF | 0.551 | 0.388 | 0.712 | 0.292 | 0.333 | 0.005 | 0.502 | 0.303 | 10 |
| M0147 | full | exp08 | 2048bit-ECFP4 | RF | 0.496 | 0.319 | 0.635 | 0.274 | 0.294 | -0.094 | 0.455 | 0.353 | 10 |
| M0148 | full | exp09 | 2048bit-ECFP4 | RF | 0.562 | 0.427 | 0.653 | 0.415 | 0.421 | 0.068 | 0.534 | 0.372 | 10 |
| M0149 | full | exp10 | 2048bit-ECFP4 | RF | 0.547 | 0.391 | 0.688 | 0.321 | 0.352 | 0.009 | 0.504 | 0.360 | 10 |
| M0150 | full | exp11 | 2048bit-ECFP4 | RF | 0.554 | 0.404 | 0.688 | 0.340 | 0.369 | 0.029 | 0.514 | 0.368 | 10 |
| M0151 | full | exp12 | 2048bit-ECFP4 | RF | 0.572 | 0.436 | 0.688 | 0.387 | 0.410 | 0.077 | 0.537 | 0.388 | 10 |
| M0152 | full | exp13 | 2048bit-ECFP4 | RF | 0.551 | 0.390 | 0.706 | 0.302 | 0.340 | 0.008 | 0.504 | 0.384 | 10 |
| M0153 | full | exp14 | 2048bit-ECFP4 | RF | 0.569 | 0.434 | 0.671 | 0.406 | 0.420 | 0.077 | 0.538 | 0.390 | 10 |
| M0154 | full | exp15 | 2048bit-ECFP4 | RF | 0.576 | 0.435 | 0.718 | 0.349 | 0.387 | 0.070 | 0.533 | 0.356 | 10 |
| M0155 | full | exp16 | 2048bit-ECFP4 | RF | 0.554 | 0.395 | 0.712 | 0.302 | 0.342 | 0.015 | 0.507 | 0.328 | 10 |
| M0156 | full | exp17 | 2048bit-ECFP4 | RF | 0.536 | 0.359 | 0.706 | 0.264 | 0.304 | -0.032 | 0.485 | 0.324 | 10 |
| M0157 | full | exp18 | 2048bit-ECFP4 | RF | 0.493 | 0.315 | 0.629 | 0.274 | 0.293 | -0.100 | 0.452 | 0.352 | 10 |
| M0158 | full | exp19 | 2048bit-ECFP4 | RF | 0.562 | 0.412 | 0.706 | 0.330 | 0.366 | 0.038 | 0.518 | 0.366 | 10 |
| M0159 | full | exp01 | 2048bit-ECFP4 | DNN2 | 0.616 | 0.000 | 1.000 | 0.000 | 0.000 | 0.000 | 0.500 | 0.085 | 100 80 0.4 |
| M0160 | full | exp02 | 2048bit-ECFP4 | DNN2 | 0.616 | 0.000 | 1.000 | 0.000 | 0.000 | 0.000 | 0.500 | 0.072 | 300 80 0.5 |
| M0161 | full | exp03 | 2048bit-ECFP4 | DNN2 | 0.536 | 0.390 | 0.641 | 0.368 | 0.379 | 0.009 | 0.504 | 0.402 | 100 60 0.4 |
| M0162 | full | exp01 | 2048bit-ECFP4 | DNN3 | 0.616 | 0.000 | 1.000 | 0.000 | 0.000 | 0.000 | 0.500 | 0.152 | 100 60 0.5 |
| M0163 | full | exp02 | 2048bit-ECFP4 | DNN3 | 0.616 | 0.000 | 1.000 | 0.000 | 0.000 | 0.000 | 0.500 | 0.000 | 100 60 0.1 |
| M0164 | full | exp03 | 2048bit-ECFP4 | DNN3 | 0.616 | 0.000 | 1.000 | 0.000 | 0.000 | 0.000 | 0.500 | 0.000 | 100 60 0.1 |
| M0165 | full | exp01 | 1024bit-ECFP6 | KNN | 0.547 | 0.406 | 0.647 | 0.387 | 0.396 | 0.034 | 0.517 | 0.400 | 1 uniform |
| M0166 | full | exp02 | 1024bit-ECFP6 | KNN | 0.511 | 0.344 | 0.641 | 0.302 | 0.322 | -0.059 | 0.472 | 0.326 | 1 uniform |
| M0167 | full | exp03 | 1024bit-ECFP6 | KNN | 0.554 | 0.419 | 0.641 | 0.415 | 0.417 | 0.056 | 0.528 | 0.399 | 1 uniform |
| M0168 | full | exp04 | 1024bit-ECFP6 | KNN | 0.475 | 0.336 | 0.535 | 0.377 | 0.356 | -0.086 | 0.456 | 0.411 | 1 uniform |
| M0169 | full | exp05 | 1024bit-ECFP6 | KNN | 0.522 | 0.386 | 0.588 | 0.415 | 0.400 | 0.003 | 0.501 | 0.380 | 1 uniform |
| M0170 | full | exp06 | 1024bit-ECFP6 | KNN | 0.475 | 0.341 | 0.524 | 0.396 | 0.367 | -0.079 | 0.460 | 0.375 | 1 uniform |
| M0171 | full | exp07 | 1024bit-ECFP6 | KNN | 0.475 | 0.362 | 0.471 | 0.481 | 0.413 | -0.047 | 0.476 | 0.369 | 1 uniform |
| M0172 | full | exp08 | 1024bit-ECFP6 | KNN | 0.493 | 0.337 | 0.594 | 0.330 | 0.333 | -0.076 | 0.462 | 0.367 | 3 uniform |
| M0173 | full | exp09 | 1024bit-ECFP6 | KNN | 0.536 | 0.398 | 0.618 | 0.406 | 0.402 | 0.023 | 0.512 | 0.429 | 1 uniform |
| M0174 | full | exp10 | 1024bit-ECFP6 | KNN | 0.460 | 0.291 | 0.571 | 0.283 | 0.287 | -0.147 | 0.427 | 0.403 | 1 uniform |
| M0175 | full | exp11 | 1024bit-ECFP6 | KNN | 0.384 | 0.246 | 0.441 | 0.292 | 0.267 | -0.260 | 0.366 | 0.439 | 11 distance |
| M0176 | full | exp12 | 1024bit-ECFP6 | KNN | 0.511 | 0.376 | 0.571 | 0.415 | 0.395 | -0.014 | 0.493 | 0.421 | 1 uniform |
| M0177 | full | exp13 | 1024bit-ECFP6 | KNN | 0.533 | 0.368 | 0.676 | 0.302 | 0.332 | -0.023 | 0.489 | 0.374 | 1 uniform |
| M0178 | full | exp14 | 1024bit-ECFP6 | KNN | 0.598 | 0.474 | 0.706 | 0.425 | 0.448 | 0.134 | 0.566 | 0.403 | 1 uniform |
| M0179 | full | exp15 | 1024bit-ECFP6 | KNN | 0.511 | 0.351 | 0.629 | 0.321 | 0.335 | -0.051 | 0.475 | 0.370 | 1 uniform |
| M0180 | full | exp16 | 1024bit-ECFP6 | KNN | 0.551 | 0.412 | 0.647 | 0.396 | 0.404 | 0.044 | 0.522 | 0.403 | 1 uniform |
| M0181 | full | exp17 | 1024bit-ECFP6 | KNN | 0.562 | 0.424 | 0.665 | 0.396 | 0.410 | 0.062 | 0.530 | 0.402 | 1 uniform |
| M0182 | full | exp18 | 1024bit-ECFP6 | KNN | 0.373 | 0.248 | 0.412 | 0.311 | 0.276 | -0.270 | 0.361 | 0.438 | 13 distance |
| M0183 | full | exp19 | 1024bit-ECFP6 | KNN | 0.551 | 0.400 | 0.682 | 0.340 | 0.367 | 0.023 | 0.511 | 0.412 | 1 uniform |
| M0184 | full | exp01 | 1024bit-ECFP6 | SVM | 0.616 | 0.000 | 1.000 | 0.000 | 0.000 | 0.000 | 0.500 | 0.026 | 10 0.01 |
| M0185 | full | exp02 | 1024bit-ECFP6 | SVM | 0.616 | 0.000 | 1.000 | 0.000 | 0.000 | 0.000 | 0.500 | 0.014 | 100000.0 0.1 |
| M0186 | full | exp03 | 1024bit-ECFP6 | SVM | 0.616 | 0.000 | 1.000 | 0.000 | 0.000 | 0.000 | 0.500 | 0.004 | 100000.0 0.1 |
| M0187 | full | exp04 | 1024bit-ECFP6 | SVM | 0.616 | 0.000 | 1.000 | 0.000 | 0.000 | 0.000 | 0.500 | 0.000 | 100 0.001 |
| M0188 | full | exp05 | 1024bit-ECFP6 | SVM | 0.616 | 0.000 | 1.000 | 0.000 | 0.000 | 0.000 | 0.500 | 0.005 | 50 0.1 |
| M0189 | full | exp06 | 1024bit-ECFP6 | SVM | 0.620 | 1.000 | 1.000 | 0.009 | 0.019 | 0.076 | 0.504 | 0.027 | 5 0.1 |
| M0190 | full | exp07 | 1024bit-ECFP6 | SVM | 0.616 | 0.000 | 1.000 | 0.000 | 0.000 | 0.000 | 0.500 | 0.046 | 50 0.1 |
| M0191 | full | exp08 | 1024bit-ECFP6 | SVM | 0.616 | 0.000 | 1.000 | 0.000 | 0.000 | 0.000 | 0.500 | 0.024 | 5000.0 0.1 |
| M0192 | full | exp09 | 1024bit-ECFP6 | SVM | 0.616 | 0.000 | 1.000 | 0.000 | 0.000 | 0.000 | 0.500 | 0.000 | 100 0.1 |
| M0193 | full | exp10 | 1024bit-ECFP6 | SVM | 0.609 | 0.000 | 0.988 | 0.000 | 0.000 | -0.067 | 0.494 | 0.015 | 5 0.1 |
| M0194 | full | exp11 | 1024bit-ECFP6 | SVM | 0.616 | 0.000 | 1.000 | 0.000 | 0.000 | 0.000 | 0.500 | 0.021 | 10000.0 0.1 |
| M0195 | full | exp12 | 1024bit-ECFP6 | SVM | 0.616 | 0.000 | 1.000 | 0.000 | 0.000 | 0.000 | 0.500 | 0.000 | 100000.0 0.001 |
| M0196 | full | exp13 | 1024bit-ECFP6 | SVM | 0.616 | 0.000 | 1.000 | 0.000 | 0.000 | 0.000 | 0.500 | 0.009 | 100000.0 5e-05 |
| M0197 | full | exp14 | 1024bit-ECFP6 | SVM | 0.616 | 0.000 | 1.000 | 0.000 | 0.000 | 0.000 | 0.500 | 0.014 | 1000.0 5e-05 |
| M0198 | full | exp15 | 1024bit-ECFP6 | SVM | 0.612 | 0.000 | 0.994 | 0.000 | 0.000 | -0.048 | 0.497 | 0.019 | 100000.0 0.0001 |
| M0199 | full | exp16 | 1024bit-ECFP6 | SVM | 0.612 | 0.400 | 0.982 | 0.019 | 0.036 | 0.004 | 0.500 | 0.103 | 10 0.005 |
| M0200 | full | exp17 | 1024bit-ECFP6 | SVM | 0.616 | 0.000 | 1.000 | 0.000 | 0.000 | 0.000 | 0.500 | 0.000 | 10000.0 5e-05 |
| M0201 | full | exp18 | 1024bit-ECFP6 | SVM | 0.616 | 0.500 | 0.994 | 0.009 | 0.019 | 0.020 | 0.501 | 0.033 | 10 0.1 |
| M0202 | full | exp19 | 1024bit-ECFP6 | SVM | 0.616 | 0.000 | 1.000 | 0.000 | 0.000 | 0.000 | 0.500 | 0.000 | 5 0.1 |
| M0203 | full | exp01 | 1024bit-ECFP6 | GBM | 0.547 | 0.362 | 0.741 | 0.236 | 0.286 | -0.026 | 0.488 | 0.406 | 100 0.9 |
| M0204 | full | exp02 | 1024bit-ECFP6 | GBM | 0.525 | 0.360 | 0.665 | 0.302 | 0.328 | -0.035 | 0.484 | 0.348 | 500 0.5 |
| M0205 | full | exp03 | 1024bit-ECFP6 | GBM | 0.562 | 0.407 | 0.718 | 0.311 | 0.353 | 0.031 | 0.514 | 0.390 | 800 0.7 |
| M0206 | full | exp04 | 1024bit-ECFP6 | GBM | 0.504 | 0.282 | 0.700 | 0.189 | 0.226 | -0.124 | 0.445 | 0.385 | 50 0.9 |
| M0207 | full | exp05 | 1024bit-ECFP6 | GBM | 0.504 | 0.337 | 0.629 | 0.302 | 0.318 | -0.070 | 0.466 | 0.376 | 600 0.9 |
| M0208 | full | exp06 | 1024bit-ECFP6 | GBM | 0.583 | 0.437 | 0.765 | 0.292 | 0.350 | 0.064 | 0.528 | 0.339 | 100 0.8 |
| M0209 | full | exp07 | 1024bit-ECFP6 | GBM | 0.638 | 0.538 | 0.788 | 0.396 | 0.457 | 0.199 | 0.592 | 0.371 | 100 0.8 |
| M0210 | full | exp08 | 1024bit-ECFP6 | GBM | 0.529 | 0.346 | 0.700 | 0.255 | 0.293 | -0.049 | 0.477 | 0.359 | 800 0.6 |
| M0211 | full | exp09 | 1024bit-ECFP6 | GBM | 0.565 | 0.430 | 0.665 | 0.406 | 0.417 | 0.071 | 0.536 | 0.380 | 1000 0.9 |
| M0212 | full | exp10 | 1024bit-ECFP6 | GBM | 0.583 | 0.442 | 0.747 | 0.321 | 0.372 | 0.074 | 0.534 | 0.412 | 600 0.6 |
| M0213 | full | exp11 | 1024bit-ECFP6 | GBM | 0.587 | 0.457 | 0.706 | 0.396 | 0.424 | 0.105 | 0.551 | 0.377 | 800 0.7 |
| M0214 | full | exp12 | 1024bit-ECFP6 | GBM | 0.587 | 0.458 | 0.694 | 0.415 | 0.436 | 0.112 | 0.554 | 0.395 | 800 0.6 |
| M0215 | full | exp13 | 1024bit-ECFP6 | GBM | 0.540 | 0.367 | 0.706 | 0.274 | 0.314 | -0.022 | 0.490 | 0.381 | 1000 0.4 |
| M0216 | full | exp14 | 1024bit-ECFP6 | GBM | 0.522 | 0.359 | 0.653 | 0.311 | 0.333 | -0.037 | 0.482 | 0.401 | 200 0.9 |
| M0217 | full | exp15 | 1024bit-ECFP6 | GBM | 0.558 | 0.400 | 0.718 | 0.302 | 0.344 | 0.021 | 0.510 | 0.366 | 100 0.8 |
| M0218 | full | exp16 | 1024bit-ECFP6 | GBM | 0.558 | 0.392 | 0.735 | 0.274 | 0.322 | 0.010 | 0.504 | 0.359 | 200 0.9 |
| M0219 | full | exp17 | 1024bit-ECFP6 | GBM | 0.533 | 0.333 | 0.729 | 0.217 | 0.263 | -0.060 | 0.473 | 0.366 | 50 0.9 |
| M0220 | full | exp18 | 1024bit-ECFP6 | GBM | 0.529 | 0.367 | 0.665 | 0.311 | 0.337 | -0.025 | 0.488 | 0.410 | 200 0.8 |
| M0221 | full | exp19 | 1024bit-ECFP6 | GBM | 0.565 | 0.403 | 0.747 | 0.274 | 0.326 | 0.023 | 0.510 | 0.365 | 50 0.8 |
| M0222 | full | exp01 | 1024bit-ECFP6 | RF | 0.453 | 0.286 | 0.559 | 0.283 | 0.284 | -0.158 | 0.421 | 0.421 | 10 |
| M0223 | full | exp02 | 1024bit-ECFP6 | RF | 0.457 | 0.300 | 0.547 | 0.311 | 0.306 | -0.141 | 0.429 | 0.392 | 10 |
| M0224 | full | exp03 | 1024bit-ECFP6 | RF | 0.522 | 0.365 | 0.641 | 0.330 | 0.347 | -0.029 | 0.486 | 0.406 | 10 |
| M0225 | full | exp04 | 1024bit-ECFP6 | RF | 0.496 | 0.357 | 0.565 | 0.387 | 0.371 | -0.048 | 0.476 | 0.399 | 10 |
| M0226 | full | exp05 | 1024bit-ECFP6 | RF | 0.475 | 0.314 | 0.576 | 0.311 | 0.313 | -0.112 | 0.444 | 0.401 | 10 |
| M0227 | full | exp06 | 1024bit-ECFP6 | RF | 0.514 | 0.365 | 0.612 | 0.358 | 0.362 | -0.030 | 0.485 | 0.386 | 10 |
| M0228 | full | exp07 | 1024bit-ECFP6 | RF | 0.543 | 0.406 | 0.629 | 0.406 | 0.406 | 0.035 | 0.518 | 0.365 | 10 |
| M0229 | full | exp08 | 1024bit-ECFP6 | RF | 0.478 | 0.310 | 0.594 | 0.292 | 0.301 | -0.115 | 0.443 | 0.395 | 10 |
| M0230 | full | exp09 | 1024bit-ECFP6 | RF | 0.540 | 0.392 | 0.653 | 0.358 | 0.374 | 0.012 | 0.506 | 0.397 | 10 |
| M0231 | full | exp10 | 1024bit-ECFP6 | RF | 0.514 | 0.360 | 0.624 | 0.340 | 0.350 | -0.037 | 0.482 | 0.402 | 10 |
| M0232 | full | exp11 | 1024bit-ECFP6 | RF | 0.536 | 0.390 | 0.641 | 0.368 | 0.379 | 0.009 | 0.504 | 0.414 | 10 |
| M0233 | full | exp12 | 1024bit-ECFP6 | RF | 0.525 | 0.391 | 0.588 | 0.425 | 0.407 | 0.013 | 0.506 | 0.419 | 10 |
| M0234 | full | exp13 | 1024bit-ECFP6 | RF | 0.493 | 0.323 | 0.618 | 0.292 | 0.307 | -0.092 | 0.455 | 0.414 | 10 |
| M0235 | full | exp14 | 1024bit-ECFP6 | RF | 0.518 | 0.383 | 0.582 | 0.415 | 0.398 | -0.003 | 0.498 | 0.417 | 10 |
| M0236 | full | exp15 | 1024bit-ECFP6 | RF | 0.507 | 0.350 | 0.618 | 0.330 | 0.340 | -0.053 | 0.474 | 0.394 | 10 |
| M0237 | full | exp16 | 1024bit-ECFP6 | RF | 0.518 | 0.348 | 0.659 | 0.292 | 0.318 | -0.051 | 0.476 | 0.332 | 10 |
| M0238 | full | exp17 | 1024bit-ECFP6 | RF | 0.457 | 0.284 | 0.571 | 0.274 | 0.279 | -0.157 | 0.422 | 0.381 | 10 |
| M0239 | full | exp18 | 1024bit-ECFP6 | RF | 0.431 | 0.278 | 0.512 | 0.302 | 0.290 | -0.184 | 0.407 | 0.416 | 10 |
| M0240 | full | exp19 | 1024bit-ECFP6 | RF | 0.551 | 0.400 | 0.682 | 0.340 | 0.367 | 0.023 | 0.511 | 0.420 | 10 |
| M0241 | full | exp01 | 1024bit-ECFP6 | DNN2 | 0.591 | 0.461 | 0.718 | 0.387 | 0.421 | 0.109 | 0.552 | 0.389 | 600 100 0.5 |
| M0242 | full | exp02 | 1024bit-ECFP6 | DNN2 | 0.551 | 0.395 | 0.694 | 0.321 | 0.354 | 0.016 | 0.507 | 0.358 | 400 120 0.4 |
| M0243 | full | exp03 | 1024bit-ECFP6 | DNN2 | 0.572 | 0.445 | 0.641 | 0.462 | 0.454 | 0.103 | 0.551 | 0.413 | 500 60 0.3 |
| M0244 | full | exp01 | 1024bit-ECFP6 | DNN3 | 0.565 | 0.427 | 0.676 | 0.387 | 0.406 | 0.065 | 0.532 | 0.400 | 600 60 0.2 |
| M0245 | full | exp02 | 1024bit-ECFP6 | DNN3 | 0.533 | 0.371 | 0.671 | 0.311 | 0.338 | -0.019 | 0.491 | 0.383 | 400 80 0.5 |
| M0246 | full | exp03 | 1024bit-ECFP6 | DNN3 | 0.587 | 0.458 | 0.694 | 0.415 | 0.436 | 0.112 | 0.554 | 0.405 | 600 120 0.3 |
| M0247 | full | exp01 | 2048bit-ECFP6 | KNN | 0.540 | 0.394 | 0.647 | 0.368 | 0.380 | 0.015 | 0.508 | 0.391 | 1 uniform |
| M0248 | full | exp02 | 2048bit-ECFP6 | KNN | 0.504 | 0.343 | 0.618 | 0.321 | 0.332 | -0.062 | 0.470 | 0.344 | 1 uniform |
| M0249 | full | exp03 | 2048bit-ECFP6 | KNN | 0.533 | 0.376 | 0.659 | 0.330 | 0.352 | -0.011 | 0.495 | 0.387 | 3 distance |
| M0250 | full | exp04 | 2048bit-ECFP6 | KNN | 0.460 | 0.325 | 0.512 | 0.377 | 0.349 | -0.108 | 0.445 | 0.400 | 1 uniform |
| M0251 | full | exp05 | 2048bit-ECFP6 | KNN | 0.533 | 0.394 | 0.612 | 0.406 | 0.400 | 0.017 | 0.509 | 0.383 | 1 uniform |
| M0252 | full | exp06 | 2048bit-ECFP6 | KNN | 0.496 | 0.370 | 0.529 | 0.443 | 0.403 | -0.027 | 0.486 | 0.387 | 1 uniform |
| M0253 | full | exp07 | 2048bit-ECFP6 | KNN | 0.449 | 0.336 | 0.453 | 0.443 | 0.382 | -0.101 | 0.448 | 0.378 | 1 uniform |
| M0254 | full | exp08 | 2048bit-ECFP6 | KNN | 0.482 | 0.313 | 0.600 | 0.292 | 0.302 | -0.109 | 0.446 | 0.373 | 3 uniform |
| M0255 | full | exp09 | 2048bit-ECFP6 | KNN | 0.522 | 0.386 | 0.588 | 0.415 | 0.400 | 0.003 | 0.501 | 0.418 | 1 uniform |
| M0256 | full | exp10 | 2048bit-ECFP6 | KNN | 0.475 | 0.314 | 0.576 | 0.311 | 0.313 | -0.112 | 0.444 | 0.402 | 1 uniform |
| M0257 | full | exp11 | 2048bit-ECFP6 | KNN | 0.373 | 0.259 | 0.394 | 0.340 | 0.294 | -0.259 | 0.367 | 0.453 | 11 distance |
| M0258 | full | exp12 | 2048bit-ECFP6 | KNN | 0.507 | 0.368 | 0.576 | 0.396 | 0.382 | -0.027 | 0.486 | 0.426 | 1 uniform |
| M0259 | full | exp13 | 2048bit-ECFP6 | KNN | 0.533 | 0.365 | 0.682 | 0.292 | 0.325 | -0.027 | 0.487 | 0.368 | 1 uniform |
| M0260 | full | exp14 | 2048bit-ECFP6 | KNN | 0.583 | 0.455 | 0.676 | 0.434 | 0.444 | 0.112 | 0.555 | 0.393 | 1 uniform |
| M0261 | full | exp15 | 2048bit-ECFP6 | KNN | 0.522 | 0.365 | 0.641 | 0.330 | 0.347 | -0.029 | 0.486 | 0.378 | 1 uniform |
| M0262 | full | exp16 | 2048bit-ECFP6 | KNN | 0.522 | 0.375 | 0.618 | 0.368 | 0.371 | -0.014 | 0.493 | 0.388 | 1 uniform |
| M0263 | full | exp17 | 2048bit-ECFP6 | KNN | 0.565 | 0.430 | 0.665 | 0.406 | 0.417 | 0.071 | 0.536 | 0.392 | 1 uniform |
| M0264 | full | exp18 | 2048bit-ECFP6 | KNN | 0.453 | 0.320 | 0.500 | 0.377 | 0.346 | -0.120 | 0.439 | 0.444 | 1 uniform |
| M0265 | full | exp19 | 2048bit-ECFP6 | KNN | 0.540 | 0.370 | 0.700 | 0.283 | 0.321 | -0.018 | 0.491 | 0.417 | 3 uniform |
| M0266 | full | exp01 | 2048bit-ECFP6 | SVM | 0.616 | 0.000 | 1.000 | 0.000 | 0.000 | 0.000 | 0.500 | 0.018 | 500 5e-05 |
| M0267 | full | exp02 | 2048bit-ECFP6 | SVM | 0.620 | 1.000 | 1.000 | 0.009 | 0.019 | 0.076 | 0.504 | 0.005 | 10000.0 0.1 |
| M0268 | full | exp03 | 2048bit-ECFP6 | SVM | 0.616 | 0.000 | 1.000 | 0.000 | 0.000 | 0.000 | 0.500 | 0.000 | 1000.0 0.1 |
| M0269 | full | exp04 | 2048bit-ECFP6 | SVM | 0.616 | 0.000 | 1.000 | 0.000 | 0.000 | 0.000 | 0.500 | 0.014 | 50000.0 0.0005 |
| M0270 | full | exp05 | 2048bit-ECFP6 | SVM | 0.616 | 0.000 | 1.000 | 0.000 | 0.000 | 0.000 | 0.500 | 0.000 | 50 0.1 |
| M0271 | full | exp06 | 2048bit-ECFP6 | SVM | 0.616 | 0.000 | 1.000 | 0.000 | 0.000 | 0.000 | 0.500 | 0.009 | 5 0.1 |
| M0272 | full | exp07 | 2048bit-ECFP6 | SVM | 0.616 | 0.000 | 1.000 | 0.000 | 0.000 | 0.000 | 0.500 | 0.034 | 100000.0 0.1 |
| M0273 | full | exp08 | 2048bit-ECFP6 | SVM | 0.616 | 0.000 | 1.000 | 0.000 | 0.000 | 0.000 | 0.500 | 0.024 | 50 0.1 |
| M0274 | full | exp09 | 2048bit-ECFP6 | SVM | 0.616 | 0.000 | 1.000 | 0.000 | 0.000 | 0.000 | 0.500 | 0.000 | 1 0.005 |
| M0275 | full | exp10 | 2048bit-ECFP6 | SVM | 0.623 | 0.750 | 0.994 | 0.028 | 0.055 | 0.091 | 0.511 | 0.054 | 5000.0 0.1 |
| M0276 | full | exp11 | 2048bit-ECFP6 | SVM | 0.616 | 0.000 | 1.000 | 0.000 | 0.000 | 0.000 | 0.500 | 0.032 | 50 0.1 |
| M0277 | full | exp12 | 2048bit-ECFP6 | SVM | 0.616 | 0.000 | 1.000 | 0.000 | 0.000 | 0.000 | 0.500 | 0.000 | 100000.0 0.005 |
| M0278 | full | exp13 | 2048bit-ECFP6 | SVM | 0.616 | 0.000 | 1.000 | 0.000 | 0.000 | 0.000 | 0.500 | 0.005 | 500 0.1 |
| M0279 | full | exp14 | 2048bit-ECFP6 | SVM | 0.616 | 0.000 | 1.000 | 0.000 | 0.000 | 0.000 | 0.500 | 0.000 | 100 0.05 |
| M0280 | full | exp15 | 2048bit-ECFP6 | SVM | 0.616 | 0.000 | 1.000 | 0.000 | 0.000 | 0.000 | 0.500 | 0.000 | 10 0.001 |
| M0281 | full | exp16 | 2048bit-ECFP6 | SVM | 0.627 | 1.000 | 1.000 | 0.028 | 0.055 | 0.133 | 0.514 | 0.026 | 1 0.005 |
| M0282 | full | exp17 | 2048bit-ECFP6 | SVM | 0.616 | 0.000 | 1.000 | 0.000 | 0.000 | 0.000 | 0.500 | 0.000 | 100000.0 1e-05 |
| M0283 | full | exp18 | 2048bit-ECFP6 | SVM | 0.620 | 0.667 | 0.994 | 0.019 | 0.037 | 0.061 | 0.506 | 0.037 | 5 0.1 |
| M0284 | full | exp19 | 2048bit-ECFP6 | SVM | 0.616 | 0.000 | 1.000 | 0.000 | 0.000 | 0.000 | 0.500 | 0.004 | 50000.0 0.1 |
| M0285 | full | exp01 | 2048bit-ECFP6 | GBM | 0.522 | 0.324 | 0.706 | 0.226 | 0.267 | -0.074 | 0.466 | 0.370 | 50 0.7 |
| M0286 | full | exp02 | 2048bit-ECFP6 | GBM | 0.529 | 0.367 | 0.665 | 0.311 | 0.337 | -0.025 | 0.488 | 0.365 | 300 0.6 |
| M0287 | full | exp03 | 2048bit-ECFP6 | GBM | 0.547 | 0.362 | 0.741 | 0.236 | 0.286 | -0.026 | 0.488 | 0.406 | 600 0.4 |
| M0288 | full | exp04 | 2048bit-ECFP6 | GBM | 0.533 | 0.368 | 0.676 | 0.302 | 0.332 | -0.023 | 0.489 | 0.368 | 100 0.7 |
| M0289 | full | exp05 | 2048bit-ECFP6 | GBM | 0.543 | 0.384 | 0.688 | 0.311 | 0.344 | 0.000 | 0.499 | 0.370 | 1000 0.3 |
| M0290 | full | exp06 | 2048bit-ECFP6 | GBM | 0.536 | 0.369 | 0.688 | 0.292 | 0.326 | -0.020 | 0.490 | 0.348 | 300 0.6 |
| M0291 | full | exp07 | 2048bit-ECFP6 | GBM | 0.591 | 0.458 | 0.735 | 0.358 | 0.402 | 0.099 | 0.546 | 0.357 | 500 0.7 |
| M0292 | full | exp08 | 2048bit-ECFP6 | GBM | 0.529 | 0.372 | 0.653 | 0.330 | 0.350 | -0.017 | 0.492 | 0.386 | 700 0.7 |
| M0293 | full | exp09 | 2048bit-ECFP6 | GBM | 0.554 | 0.404 | 0.688 | 0.340 | 0.369 | 0.029 | 0.514 | 0.396 | 900 0.9 |
| M0294 | full | exp10 | 2048bit-ECFP6 | GBM | 0.554 | 0.398 | 0.706 | 0.311 | 0.349 | 0.018 | 0.508 | 0.423 | 400 0.4 |
| M0295 | full | exp11 | 2048bit-ECFP6 | GBM | 0.536 | 0.380 | 0.665 | 0.330 | 0.354 | -0.005 | 0.498 | 0.405 | 900 0.5 |
| M0296 | full | exp12 | 2048bit-ECFP6 | GBM | 0.554 | 0.417 | 0.647 | 0.406 | 0.411 | 0.053 | 0.526 | 0.380 | 600 0.6 |
| M0297 | full | exp13 | 2048bit-ECFP6 | GBM | 0.551 | 0.385 | 0.718 | 0.283 | 0.326 | 0.001 | 0.500 | 0.424 | 500 0.2 |
| M0298 | full | exp14 | 2048bit-ECFP6 | GBM | 0.551 | 0.400 | 0.682 | 0.340 | 0.367 | 0.023 | 0.511 | 0.420 | 700 0.9 |
| M0299 | full | exp15 | 2048bit-ECFP6 | GBM | 0.525 | 0.368 | 0.647 | 0.330 | 0.348 | -0.023 | 0.489 | 0.391 | 900 0.7 |
| M0300 | full | exp16 | 2048bit-ECFP6 | GBM | 0.562 | 0.385 | 0.765 | 0.236 | 0.292 | 0.001 | 0.500 | 0.349 | 50 0.7 |
| M0301 | full | exp17 | 2048bit-ECFP6 | GBM | 0.518 | 0.337 | 0.676 | 0.264 | 0.296 | -0.063 | 0.470 | 0.369 | 700 0.5 |
| M0302 | full | exp18 | 2048bit-ECFP6 | GBM | 0.493 | 0.307 | 0.641 | 0.255 | 0.278 | -0.109 | 0.448 | 0.372 | 200 0.7 |
| M0303 | full | exp19 | 2048bit-ECFP6 | GBM | 0.543 | 0.378 | 0.700 | 0.292 | 0.330 | -0.008 | 0.496 | 0.394 | 300 0.3 |
| M0304 | full | exp01 | 2048bit-ECFP6 | RF | 0.489 | 0.320 | 0.612 | 0.292 | 0.305 | -0.098 | 0.452 | 0.387 | 10 |
| M0305 | full | exp02 | 2048bit-ECFP6 | RF | 0.471 | 0.304 | 0.582 | 0.292 | 0.298 | -0.126 | 0.437 | 0.352 | 10 |
| M0306 | full | exp03 | 2048bit-ECFP6 | RF | 0.522 | 0.359 | 0.653 | 0.311 | 0.333 | -0.037 | 0.482 | 0.401 | 10 |
| M0307 | full | exp04 | 2048bit-ECFP6 | RF | 0.464 | 0.322 | 0.529 | 0.358 | 0.339 | -0.110 | 0.444 | 0.402 | 10 |
| M0308 | full | exp05 | 2048bit-ECFP6 | RF | 0.500 | 0.322 | 0.641 | 0.274 | 0.296 | -0.088 | 0.458 | 0.366 | 10 |
| M0309 | full | exp06 | 2048bit-ECFP6 | RF | 0.496 | 0.340 | 0.600 | 0.330 | 0.335 | -0.070 | 0.465 | 0.352 | 10 |
| M0310 | full | exp07 | 2048bit-ECFP6 | RF | 0.511 | 0.356 | 0.618 | 0.340 | 0.348 | -0.043 | 0.479 | 0.339 | 10 |
| M0311 | full | exp08 | 2048bit-ECFP6 | RF | 0.489 | 0.320 | 0.612 | 0.292 | 0.305 | -0.098 | 0.452 | 0.382 | 10 |
| M0312 | full | exp09 | 2048bit-ECFP6 | RF | 0.554 | 0.411 | 0.671 | 0.368 | 0.388 | 0.039 | 0.520 | 0.368 | 10 |
| M0313 | full | exp10 | 2048bit-ECFP6 | RF | 0.522 | 0.380 | 0.606 | 0.387 | 0.383 | -0.007 | 0.496 | 0.424 | 10 |
| M0314 | full | exp11 | 2048bit-ECFP6 | RF | 0.496 | 0.319 | 0.635 | 0.274 | 0.294 | -0.094 | 0.455 | 0.367 | 10 |
| M0315 | full | exp12 | 2048bit-ECFP6 | RF | 0.547 | 0.408 | 0.641 | 0.396 | 0.402 | 0.038 | 0.518 | 0.376 | 10 |
| M0316 | full | exp13 | 2048bit-ECFP6 | RF | 0.551 | 0.388 | 0.712 | 0.292 | 0.333 | 0.005 | 0.502 | 0.394 | 10 |
| M0317 | full | exp14 | 2048bit-ECFP6 | RF | 0.522 | 0.356 | 0.659 | 0.302 | 0.327 | -0.041 | 0.481 | 0.408 | 10 |
| M0318 | full | exp15 | 2048bit-ECFP6 | RF | 0.489 | 0.327 | 0.600 | 0.311 | 0.319 | -0.090 | 0.456 | 0.374 | 10 |
| M0319 | full | exp16 | 2048bit-ECFP6 | RF | 0.518 | 0.348 | 0.659 | 0.292 | 0.318 | -0.051 | 0.476 | 0.335 | 10 |
| M0320 | full | exp17 | 2048bit-ECFP6 | RF | 0.493 | 0.298 | 0.653 | 0.236 | 0.263 | -0.118 | 0.445 | 0.378 | 10 |
| M0321 | full | exp18 | 2048bit-ECFP6 | RF | 0.489 | 0.312 | 0.624 | 0.274 | 0.291 | -0.106 | 0.449 | 0.389 | 10 |
| M0322 | full | exp19 | 2048bit-ECFP6 | RF | 0.533 | 0.381 | 0.647 | 0.349 | 0.365 | -0.004 | 0.498 | 0.388 | 10 |
| M0323 | full | exp01 | 2048bit-ECFP6 | DNN2 | 0.616 | 0.000 | 1.000 | 0.000 | 0.000 | 0.000 | 0.500 | 0.086 | 200 80 0.5 |
| M0324 | full | exp02 | 2048bit-ECFP6 | DNN2 | 0.616 | 0.000 | 1.000 | 0.000 | 0.000 | 0.000 | 0.500 | 0.000 | 100 60 0.1 |
| M0325 | full | exp03 | 2048bit-ECFP6 | DNN2 | 0.562 | 0.423 | 0.671 | 0.387 | 0.404 | 0.058 | 0.529 | 0.413 | 600 60 0.5 |
| M0326 | full | exp01 | 2048bit-ECFP6 | DNN3 | 0.616 | 0.000 | 1.000 | 0.000 | 0.000 | 0.000 | 0.500 | 0.071 | 400 140 0.5 |
| M0327 | full | exp02 | 2048bit-ECFP6 | DNN3 | 0.616 | 0.000 | 1.000 | 0.000 | 0.000 | 0.000 | 0.500 | 0.000 | 100 60 0.1 |
| M0328 | full | exp03 | 2048bit-ECFP6 | DNN3 | 0.616 | 0.000 | 1.000 | 0.000 | 0.000 | 0.000 | 0.500 | 0.000 | 100 60 0.1 |
| M0329 | 512 | exp01 | 1024bit-ECFP4 | KNN | 0.540 | 0.385 | 0.671 | 0.330 | 0.355 | 0.001 | 0.501 | 0.390 | 1 uniform |
| M0330 | 512 | exp02 | 1024bit-ECFP4 | KNN | 0.518 | 0.374 | 0.606 | 0.377 | 0.376 | -0.017 | 0.491 | 0.352 | 1 uniform |
| M0331 | 512 | exp03 | 1024bit-ECFP4 | KNN | 0.562 | 0.421 | 0.676 | 0.377 | 0.398 | 0.055 | 0.526 | 0.372 | 1 uniform |
| M0332 | 512 | exp04 | 1024bit-ECFP4 | KNN | 0.554 | 0.411 | 0.671 | 0.368 | 0.388 | 0.039 | 0.520 | 0.396 | 1 uniform |
| M0333 | 512 | exp05 | 1024bit-ECFP4 | KNN | 0.536 | 0.383 | 0.659 | 0.340 | 0.360 | -0.002 | 0.500 | 0.389 | 1 uniform |
| M0334 | 512 | exp06 | 1024bit-ECFP4 | KNN | 0.536 | 0.404 | 0.600 | 0.434 | 0.418 | 0.034 | 0.517 | 0.359 | 1 uniform |
| M0335 | 512 | exp07 | 1024bit-ECFP4 | KNN | 0.489 | 0.339 | 0.576 | 0.349 | 0.344 | -0.074 | 0.462 | 0.356 | 1 uniform |
| M0336 | 512 | exp08 | 1024bit-ECFP4 | KNN | 0.493 | 0.330 | 0.606 | 0.311 | 0.320 | -0.084 | 0.459 | 0.357 | 1 uniform |
| M0337 | 512 | exp09 | 1024bit-ECFP4 | KNN | 0.591 | 0.463 | 0.700 | 0.415 | 0.438 | 0.118 | 0.557 | 0.387 | 1 uniform |
| M0338 | 512 | exp10 | 1024bit-ECFP4 | KNN | 0.489 | 0.333 | 0.588 | 0.330 | 0.332 | -0.082 | 0.459 | 0.421 | 1 uniform |
| M0339 | 512 | exp11 | 1024bit-ECFP4 | KNN | 0.507 | 0.356 | 0.606 | 0.349 | 0.352 | -0.045 | 0.477 | 0.435 | 1 uniform |
| M0340 | 512 | exp12 | 1024bit-ECFP4 | KNN | 0.533 | 0.379 | 0.653 | 0.340 | 0.358 | -0.008 | 0.497 | 0.376 | 1 uniform |
| M0341 | 512 | exp13 | 1024bit-ECFP4 | KNN | 0.554 | 0.404 | 0.688 | 0.340 | 0.369 | 0.029 | 0.514 | 0.376 | 1 uniform |
| M0342 | 512 | exp14 | 1024bit-ECFP4 | KNN | 0.580 | 0.450 | 0.676 | 0.425 | 0.437 | 0.102 | 0.550 | 0.387 | 1 uniform |
| M0343 | 512 | exp15 | 1024bit-ECFP4 | KNN | 0.504 | 0.347 | 0.612 | 0.330 | 0.338 | -0.059 | 0.471 | 0.360 | 1 uniform |
| M0344 | 512 | exp16 | 1024bit-ECFP4 | KNN | 0.518 | 0.358 | 0.641 | 0.321 | 0.338 | -0.039 | 0.481 | 0.379 | 1 uniform |
| M0345 | 512 | exp17 | 1024bit-ECFP4 | KNN | 0.533 | 0.388 | 0.629 | 0.377 | 0.383 | 0.007 | 0.503 | 0.369 | 1 uniform |
| M0346 | 512 | exp18 | 1024bit-ECFP4 | KNN | 0.496 | 0.326 | 0.624 | 0.292 | 0.308 | -0.086 | 0.458 | 0.399 | 1 uniform |
| M0347 | 512 | exp19 | 1024bit-ECFP4 | KNN | 0.562 | 0.410 | 0.712 | 0.321 | 0.360 | 0.034 | 0.516 | 0.375 | 1 uniform |
| M0348 | 512 | exp01 | 1024bit-ECFP4 | SVM | 0.612 | 0.000 | 0.994 | 0.000 | 0.000 | -0.048 | 0.497 | 0.058 | 100000.0 0.05 |
| M0349 | 512 | exp02 | 1024bit-ECFP4 | SVM | 0.616 | 0.000 | 1.000 | 0.000 | 0.000 | 0.000 | 0.500 | 0.005 | 10000.0 0.0005 |
| M0350 | 512 | exp03 | 1024bit-ECFP4 | SVM | 0.616 | 0.000 | 1.000 | 0.000 | 0.000 | 0.000 | 0.500 | 0.000 | 1000.0 0.0001 |
| M0351 | 512 | exp04 | 1024bit-ECFP4 | SVM | 0.616 | 0.000 | 1.000 | 0.000 | 0.000 | 0.000 | 0.500 | 0.000 | 50000.0 0.1 |
| M0352 | 512 | exp05 | 1024bit-ECFP4 | SVM | 0.616 | 0.000 | 1.000 | 0.000 | 0.000 | 0.000 | 0.500 | 0.000 | 1000.0 0.001 |
| M0353 | 512 | exp06 | 1024bit-ECFP4 | SVM | 0.616 | 0.000 | 1.000 | 0.000 | 0.000 | 0.000 | 0.500 | 0.000 | 1000.0 0.0005 |
| M0354 | 512 | exp07 | 1024bit-ECFP4 | SVM | 0.616 | 0.000 | 1.000 | 0.000 | 0.000 | 0.000 | 0.500 | 0.016 | 5 0.001 |
| M0355 | 512 | exp08 | 1024bit-ECFP4 | SVM | 0.616 | 0.000 | 1.000 | 0.000 | 0.000 | 0.000 | 0.500 | 0.036 | 50 0.01 |
| M0356 | 512 | exp09 | 1024bit-ECFP4 | SVM | 0.616 | 0.000 | 1.000 | 0.000 | 0.000 | 0.000 | 0.500 | 0.047 | 10000.0 0.05 |
| M0357 | 512 | exp10 | 1024bit-ECFP4 | SVM | 0.616 | 0.000 | 1.000 | 0.000 | 0.000 | 0.000 | 0.500 | 0.014 | 100 0.001 |
| M0358 | 512 | exp11 | 1024bit-ECFP4 | SVM | 0.612 | 0.000 | 0.994 | 0.000 | 0.000 | -0.048 | 0.497 | 0.043 | 10000.0 0.005 |
| M0359 | 512 | exp12 | 1024bit-ECFP4 | SVM | 0.616 | 0.000 | 1.000 | 0.000 | 0.000 | 0.000 | 0.500 | 0.000 | 10 0.05 |
| M0360 | 512 | exp13 | 1024bit-ECFP4 | SVM | 0.616 | 0.000 | 1.000 | 0.000 | 0.000 | 0.000 | 0.500 | 0.046 | 100000.0 0.005 |
| M0361 | 512 | exp14 | 1024bit-ECFP4 | SVM | 0.616 | 0.000 | 1.000 | 0.000 | 0.000 | 0.000 | 0.500 | 0.004 | 1000.0 0.0001 |
| M0362 | 512 | exp15 | 1024bit-ECFP4 | SVM | 0.616 | 0.000 | 1.000 | 0.000 | 0.000 | 0.000 | 0.500 | 0.027 | 10 0.1 |
| M0363 | 512 | exp16 | 1024bit-ECFP4 | SVM | 0.616 | 0.000 | 1.000 | 0.000 | 0.000 | 0.000 | 0.500 | 0.020 | 5 0.01 |
| M0364 | 512 | exp17 | 1024bit-ECFP4 | SVM | 0.616 | 0.000 | 1.000 | 0.000 | 0.000 | 0.000 | 0.500 | 0.000 | 1000.0 0.0005 |
| M0365 | 512 | exp18 | 1024bit-ECFP4 | SVM | 0.623 | 1.000 | 1.000 | 0.019 | 0.037 | 0.108 | 0.509 | 0.058 | 10000.0 0.1 |
| M0366 | 512 | exp19 | 1024bit-ECFP4 | SVM | 0.620 | 1.000 | 1.000 | 0.009 | 0.019 | 0.076 | 0.504 | 0.062 | 10 0.005 |
| M0367 | 512 | exp01 | 1024bit-ECFP4 | GBM | 0.507 | 0.326 | 0.659 | 0.264 | 0.292 | -0.081 | 0.462 | 0.393 | 200 0.7 |
| M0368 | 512 | exp02 | 1024bit-ECFP4 | GBM | 0.504 | 0.326 | 0.647 | 0.274 | 0.297 | -0.083 | 0.461 | 0.356 | 900 0.8 |
| M0369 | 512 | exp03 | 1024bit-ECFP4 | GBM | 0.536 | 0.362 | 0.700 | 0.274 | 0.312 | -0.028 | 0.487 | 0.420 | 100 0.8 |
| M0370 | 512 | exp04 | 1024bit-ECFP4 | GBM | 0.529 | 0.378 | 0.641 | 0.349 | 0.363 | -0.010 | 0.495 | 0.389 | 400 0.9 |
| M0371 | 512 | exp05 | 1024bit-ECFP4 | GBM | 0.496 | 0.310 | 0.647 | 0.255 | 0.280 | -0.103 | 0.451 | 0.396 | 300 0.4 |
| M0372 | 512 | exp06 | 1024bit-ECFP4 | GBM | 0.514 | 0.337 | 0.665 | 0.274 | 0.302 | -0.065 | 0.470 | 0.389 | 300 0.5 |
| M0373 | 512 | exp07 | 1024bit-ECFP4 | GBM | 0.543 | 0.398 | 0.653 | 0.368 | 0.382 | 0.021 | 0.510 | 0.379 | 200 0.9 |
| M0374 | 512 | exp08 | 1024bit-ECFP4 | GBM | 0.507 | 0.333 | 0.647 | 0.283 | 0.306 | -0.073 | 0.465 | 0.414 | 700 0.8 |
| M0375 | 512 | exp09 | 1024bit-ECFP4 | GBM | 0.565 | 0.430 | 0.665 | 0.406 | 0.417 | 0.071 | 0.536 | 0.428 | 1000 0.8 |
| M0376 | 512 | exp10 | 1024bit-ECFP4 | GBM | 0.583 | 0.451 | 0.706 | 0.387 | 0.416 | 0.096 | 0.546 | 0.408 | 700 0.7 |
| M0377 | 512 | exp11 | 1024bit-ECFP4 | GBM | 0.540 | 0.385 | 0.671 | 0.330 | 0.355 | 0.001 | 0.501 | 0.416 | 300 0.6 |
| M0378 | 512 | exp12 | 1024bit-ECFP4 | GBM | 0.569 | 0.425 | 0.706 | 0.349 | 0.383 | 0.058 | 0.527 | 0.408 | 500 0.9 |
| M0379 | 512 | exp13 | 1024bit-ECFP4 | GBM | 0.511 | 0.341 | 0.647 | 0.292 | 0.315 | -0.063 | 0.470 | 0.439 | 900 0.8 |
| M0380 | 512 | exp14 | 1024bit-ECFP4 | GBM | 0.565 | 0.422 | 0.694 | 0.358 | 0.388 | 0.055 | 0.526 | 0.419 | 1000 0.7 |
| M0381 | 512 | exp15 | 1024bit-ECFP4 | GBM | 0.558 | 0.411 | 0.688 | 0.349 | 0.378 | 0.039 | 0.518 | 0.396 | 400 0.5 |
| M0382 | 512 | exp16 | 1024bit-ECFP4 | GBM | 0.525 | 0.360 | 0.665 | 0.302 | 0.328 | -0.035 | 0.484 | 0.380 | 500 0.5 |
| M0383 | 512 | exp17 | 1024bit-ECFP4 | GBM | 0.514 | 0.341 | 0.659 | 0.283 | 0.309 | -0.061 | 0.471 | 0.404 | 800 0.7 |
| M0384 | 512 | exp18 | 1024bit-ECFP4 | GBM | 0.536 | 0.383 | 0.659 | 0.340 | 0.360 | -0.002 | 0.500 | 0.439 | 200 0.7 |
| M0385 | 512 | exp19 | 1024bit-ECFP4 | GBM | 0.572 | 0.412 | 0.765 | 0.264 | 0.322 | 0.033 | 0.514 | 0.429 | 100 0.9 |
| M0386 | 512 | exp01 | 1024bit-ECFP4 | RF | 0.543 | 0.381 | 0.694 | 0.302 | 0.337 | -0.004 | 0.498 | 0.375 | 10 |
| M0387 | 512 | exp02 | 1024bit-ECFP4 | RF | 0.514 | 0.348 | 0.647 | 0.302 | 0.323 | -0.053 | 0.475 | 0.365 | 10 |
| M0388 | 512 | exp03 | 1024bit-ECFP4 | RF | 0.507 | 0.312 | 0.676 | 0.236 | 0.269 | -0.094 | 0.456 | 0.413 | 10 |
| M0389 | 512 | exp04 | 1024bit-ECFP4 | RF | 0.540 | 0.382 | 0.676 | 0.321 | 0.349 | -0.003 | 0.499 | 0.382 | 10 |
| M0390 | 512 | exp05 | 1024bit-ECFP4 | RF | 0.525 | 0.353 | 0.676 | 0.283 | 0.314 | -0.043 | 0.480 | 0.383 | 10 |
| M0391 | 512 | exp06 | 1024bit-ECFP4 | RF | 0.569 | 0.434 | 0.671 | 0.406 | 0.420 | 0.077 | 0.538 | 0.350 | 10 |
| M0392 | 512 | exp07 | 1024bit-ECFP4 | RF | 0.518 | 0.337 | 0.676 | 0.264 | 0.296 | -0.063 | 0.470 | 0.358 | 10 |
| M0393 | 512 | exp08 | 1024bit-ECFP4 | RF | 0.507 | 0.326 | 0.659 | 0.264 | 0.292 | -0.081 | 0.462 | 0.367 | 10 |
| M0394 | 512 | exp09 | 1024bit-ECFP4 | RF | 0.601 | 0.480 | 0.688 | 0.462 | 0.471 | 0.152 | 0.575 | 0.401 | 10 |
| M0395 | 512 | exp10 | 1024bit-ECFP4 | RF | 0.558 | 0.407 | 0.700 | 0.330 | 0.365 | 0.032 | 0.515 | 0.396 | 10 |
| M0396 | 512 | exp11 | 1024bit-ECFP4 | RF | 0.547 | 0.393 | 0.682 | 0.330 | 0.359 | 0.013 | 0.506 | 0.404 | 10 |
| M0397 | 512 | exp12 | 1024bit-ECFP4 | RF | 0.591 | 0.462 | 0.712 | 0.396 | 0.426 | 0.112 | 0.554 | 0.362 | 10 |
| M0398 | 512 | exp13 | 1024bit-ECFP4 | RF | 0.569 | 0.424 | 0.712 | 0.340 | 0.377 | 0.054 | 0.526 | 0.382 | 10 |
| M0399 | 512 | exp14 | 1024bit-ECFP4 | RF | 0.565 | 0.424 | 0.688 | 0.368 | 0.394 | 0.058 | 0.528 | 0.401 | 10 |
| M0400 | 512 | exp15 | 1024bit-ECFP4 | RF | 0.529 | 0.387 | 0.618 | 0.387 | 0.387 | 0.004 | 0.502 | 0.380 | 10 |
| M0401 | 512 | exp16 | 1024bit-ECFP4 | RF | 0.551 | 0.393 | 0.700 | 0.311 | 0.347 | 0.012 | 0.505 | 0.372 | 10 |
| M0402 | 512 | exp17 | 1024bit-ECFP4 | RF | 0.525 | 0.342 | 0.694 | 0.255 | 0.292 | -0.055 | 0.474 | 0.358 | 10 |
| M0403 | 512 | exp18 | 1024bit-ECFP4 | RF | 0.500 | 0.326 | 0.635 | 0.283 | 0.303 | -0.084 | 0.459 | 0.348 | 10 |
| M0404 | 512 | exp19 | 1024bit-ECFP4 | RF | 0.572 | 0.412 | 0.765 | 0.264 | 0.322 | 0.033 | 0.514 | 0.410 | 10 |
| M0405 | 512 | exp01 | 1024bit-ECFP4 | DNN2 | 0.536 | 0.390 | 0.641 | 0.368 | 0.379 | 0.009 | 0.504 | 0.409 | 300 60 0.3 |
| M0406 | 512 | exp02 | 1024bit-ECFP4 | DNN2 | 0.514 | 0.344 | 0.653 | 0.292 | 0.316 | -0.057 | 0.473 | 0.378 | 300 120 0.2 |
| M0407 | 512 | exp03 | 1024bit-ECFP4 | DNN2 | 0.543 | 0.386 | 0.682 | 0.321 | 0.351 | 0.003 | 0.502 | 0.414 | 600 120 0.1 |
| M0408 | 512 | exp01 | 1024bit-ECFP4 | DNN3 | 0.522 | 0.377 | 0.612 | 0.377 | 0.377 | -0.011 | 0.494 | 0.405 | 200 60 0.3 |
| M0409 | 512 | exp02 | 1024bit-ECFP4 | DNN3 | 0.522 | 0.359 | 0.653 | 0.311 | 0.333 | -0.037 | 0.482 | 0.373 | 200 60 0.5 |
| M0410 | 512 | exp03 | 1024bit-ECFP4 | DNN3 | 0.536 | 0.390 | 0.641 | 0.368 | 0.379 | 0.009 | 0.504 | 0.425 | 100 60 0.5 |
| M0411 | 512 | exp01 | 2048bit-ECFP4 | KNN | 0.525 | 0.360 | 0.665 | 0.302 | 0.328 | -0.035 | 0.484 | 0.382 | 1 uniform |
| M0412 | 512 | exp02 | 2048bit-ECFP4 | KNN | 0.543 | 0.384 | 0.688 | 0.311 | 0.344 | 0.000 | 0.499 | 0.344 | 1 uniform |
| M0413 | 512 | exp03 | 2048bit-ECFP4 | KNN | 0.576 | 0.441 | 0.694 | 0.387 | 0.412 | 0.083 | 0.540 | 0.386 | 1 uniform |
| M0414 | 512 | exp04 | 2048bit-ECFP4 | KNN | 0.540 | 0.379 | 0.682 | 0.311 | 0.342 | -0.007 | 0.497 | 0.411 | 1 uniform |
| M0415 | 512 | exp05 | 2048bit-ECFP4 | KNN | 0.565 | 0.420 | 0.700 | 0.349 | 0.381 | 0.051 | 0.524 | 0.400 | 1 uniform |
| M0416 | 512 | exp06 | 2048bit-ECFP4 | KNN | 0.529 | 0.393 | 0.600 | 0.415 | 0.404 | 0.015 | 0.507 | 0.350 | 1 uniform |
| M0417 | 512 | exp07 | 2048bit-ECFP4 | KNN | 0.504 | 0.350 | 0.606 | 0.340 | 0.344 | -0.055 | 0.473 | 0.349 | 1 uniform |
| M0418 | 512 | exp08 | 2048bit-ECFP4 | KNN | 0.493 | 0.307 | 0.641 | 0.255 | 0.278 | -0.109 | 0.448 | 0.355 | 1 uniform |
| M0419 | 512 | exp09 | 2048bit-ECFP4 | KNN | 0.580 | 0.448 | 0.688 | 0.406 | 0.426 | 0.096 | 0.547 | 0.383 | 1 uniform |
| M0420 | 512 | exp10 | 2048bit-ECFP4 | KNN | 0.504 | 0.340 | 0.624 | 0.311 | 0.325 | -0.066 | 0.468 | 0.385 | 1 uniform |
| M0421 | 512 | exp11 | 2048bit-ECFP4 | KNN | 0.525 | 0.374 | 0.635 | 0.349 | 0.361 | -0.016 | 0.492 | 0.436 | 1 uniform |
| M0422 | 512 | exp12 | 2048bit-ECFP4 | KNN | 0.540 | 0.396 | 0.641 | 0.377 | 0.386 | 0.019 | 0.509 | 0.355 | 1 uniform |
| M0423 | 512 | exp13 | 2048bit-ECFP4 | KNN | 0.551 | 0.406 | 0.665 | 0.368 | 0.386 | 0.033 | 0.516 | 0.395 | 1 uniform |
| M0424 | 512 | exp14 | 2048bit-ECFP4 | KNN | 0.547 | 0.408 | 0.641 | 0.396 | 0.402 | 0.038 | 0.518 | 0.377 | 1 uniform |
| M0425 | 512 | exp15 | 2048bit-ECFP4 | KNN | 0.562 | 0.414 | 0.700 | 0.340 | 0.373 | 0.041 | 0.520 | 0.346 | 1 uniform |
| M0426 | 512 | exp16 | 2048bit-ECFP4 | KNN | 0.572 | 0.435 | 0.694 | 0.377 | 0.404 | 0.074 | 0.535 | 0.375 | 1 uniform |
| M0427 | 512 | exp17 | 2048bit-ECFP4 | KNN | 0.514 | 0.354 | 0.635 | 0.321 | 0.337 | -0.045 | 0.478 | 0.363 | 1 uniform |
| M0428 | 512 | exp18 | 2048bit-ECFP4 | KNN | 0.496 | 0.330 | 0.618 | 0.302 | 0.315 | -0.082 | 0.460 | 0.419 | 1 uniform |
| M0429 | 512 | exp19 | 2048bit-ECFP4 | KNN | 0.591 | 0.457 | 0.741 | 0.349 | 0.396 | 0.096 | 0.545 | 0.377 | 1 uniform |
| M0430 | 512 | exp01 | 2048bit-ECFP4 | SVM | 0.601 | 0.250 | 0.965 | 0.019 | 0.035 | -0.048 | 0.492 | 0.147 | 100000.0 0.05 |
| M0431 | 512 | exp02 | 2048bit-ECFP4 | SVM | 0.612 | 0.000 | 0.994 | 0.000 | 0.000 | -0.048 | 0.497 | 0.016 | 5 0.01 |
| M0432 | 512 | exp03 | 2048bit-ECFP4 | SVM | 0.612 | 0.429 | 0.976 | 0.028 | 0.053 | 0.015 | 0.502 | 0.088 | 100 0.005 |
| M0433 | 512 | exp04 | 2048bit-ECFP4 | SVM | 0.601 | 0.167 | 0.971 | 0.009 | 0.018 | -0.067 | 0.490 | 0.090 | 50000.0 0.01 |
| M0434 | 512 | exp05 | 2048bit-ECFP4 | SVM | 0.620 | 1.000 | 1.000 | 0.009 | 0.019 | 0.076 | 0.504 | 0.049 | 1000.0 0.05 |
| M0435 | 512 | exp06 | 2048bit-ECFP4 | SVM | 0.620 | 0.667 | 0.994 | 0.019 | 0.037 | 0.061 | 0.506 | 0.058 | 10 0.01 |
| M0436 | 512 | exp07 | 2048bit-ECFP4 | SVM | 0.616 | 0.000 | 1.000 | 0.000 | 0.000 | 0.000 | 0.500 | 0.033 | 500 1e-05 |
| M0437 | 512 | exp08 | 2048bit-ECFP4 | SVM | 0.594 | 0.000 | 0.965 | 0.000 | 0.000 | -0.118 | 0.482 | 0.067 | 50000.0 0.01 |
| M0438 | 512 | exp09 | 2048bit-ECFP4 | SVM | 0.674 | 0.864 | 0.982 | 0.179 | 0.297 | 0.290 | 0.581 | 0.170 | 5 0.05 |
| M0439 | 512 | exp10 | 2048bit-ECFP4 | SVM | 0.630 | 0.643 | 0.971 | 0.085 | 0.150 | 0.123 | 0.528 | 0.132 | 100000.0 0.05 |
| M0440 | 512 | exp11 | 2048bit-ECFP4 | SVM | 0.601 | 0.000 | 0.976 | 0.000 | 0.000 | -0.096 | 0.488 | 0.080 | 5000.0 0.05 |
| M0441 | 512 | exp12 | 2048bit-ECFP4 | SVM | 0.609 | 0.333 | 0.976 | 0.019 | 0.036 | -0.016 | 0.497 | 0.079 | 100 0.01 |
| M0442 | 512 | exp13 | 2048bit-ECFP4 | SVM | 0.609 | 0.429 | 0.953 | 0.057 | 0.100 | 0.021 | 0.505 | 0.160 | 50 0.01 |
| M0443 | 512 | exp14 | 2048bit-ECFP4 | SVM | 0.616 | 0.000 | 1.000 | 0.000 | 0.000 | 0.000 | 0.500 | 0.027 | 1 0.05 |
| M0444 | 512 | exp15 | 2048bit-ECFP4 | SVM | 0.616 | 0.000 | 1.000 | 0.000 | 0.000 | 0.000 | 0.500 | 0.028 | 100000.0 0.0005 |
| M0445 | 512 | exp16 | 2048bit-ECFP4 | SVM | 0.616 | 0.000 | 1.000 | 0.000 | 0.000 | 0.000 | 0.500 | 0.005 | 50000.0 0.0005 |
| M0446 | 512 | exp17 | 2048bit-ECFP4 | SVM | 0.616 | 0.000 | 1.000 | 0.000 | 0.000 | 0.000 | 0.500 | 0.028 | 10000.0 0.0005 |
| M0447 | 512 | exp18 | 2048bit-ECFP4 | SVM | 0.609 | 0.375 | 0.971 | 0.028 | 0.053 | -0.003 | 0.499 | 0.128 | 10000.0 0.005 |
| M0448 | 512 | exp19 | 2048bit-ECFP4 | SVM | 0.612 | 0.000 | 0.994 | 0.000 | 0.000 | -0.048 | 0.497 | 0.067 | 10 0.01 |
| M0449 | 512 | exp01 | 2048bit-ECFP4 | GBM | 0.547 | 0.380 | 0.712 | 0.283 | 0.324 | -0.006 | 0.497 | 0.410 | 300 0.8 |
| M0450 | 512 | exp02 | 2048bit-ECFP4 | GBM | 0.507 | 0.330 | 0.653 | 0.274 | 0.299 | -0.077 | 0.464 | 0.393 | 600 0.4 |
| M0451 | 512 | exp03 | 2048bit-ECFP4 | GBM | 0.572 | 0.429 | 0.718 | 0.340 | 0.379 | 0.061 | 0.529 | 0.438 | 100 0.7 |
| M0452 | 512 | exp04 | 2048bit-ECFP4 | GBM | 0.493 | 0.302 | 0.647 | 0.245 | 0.271 | -0.113 | 0.446 | 0.426 | 300 0.8 |
| M0453 | 512 | exp05 | 2048bit-ECFP4 | GBM | 0.558 | 0.392 | 0.735 | 0.274 | 0.322 | 0.010 | 0.504 | 0.408 | 100 0.6 |
| M0454 | 512 | exp06 | 2048bit-ECFP4 | GBM | 0.569 | 0.424 | 0.712 | 0.340 | 0.377 | 0.054 | 0.526 | 0.407 | 100 0.8 |
| M0455 | 512 | exp07 | 2048bit-ECFP4 | GBM | 0.554 | 0.400 | 0.700 | 0.321 | 0.356 | 0.022 | 0.510 | 0.379 | 1000 0.6 |
| M0456 | 512 | exp08 | 2048bit-ECFP4 | GBM | 0.543 | 0.372 | 0.712 | 0.274 | 0.315 | -0.016 | 0.493 | 0.430 | 50 0.8 |
| M0457 | 512 | exp09 | 2048bit-ECFP4 | GBM | 0.580 | 0.449 | 0.682 | 0.415 | 0.431 | 0.099 | 0.548 | 0.453 | 800 0.7 |
| M0458 | 512 | exp10 | 2048bit-ECFP4 | GBM | 0.554 | 0.417 | 0.647 | 0.406 | 0.411 | 0.053 | 0.526 | 0.428 | 200 0.8 |
| M0459 | 512 | exp11 | 2048bit-ECFP4 | GBM | 0.522 | 0.345 | 0.676 | 0.274 | 0.305 | -0.053 | 0.475 | 0.457 | 1000 0.9 |
| M0460 | 512 | exp12 | 2048bit-ECFP4 | GBM | 0.533 | 0.374 | 0.665 | 0.321 | 0.345 | -0.015 | 0.493 | 0.439 | 600 0.8 |
| M0461 | 512 | exp13 | 2048bit-ECFP4 | GBM | 0.543 | 0.391 | 0.671 | 0.340 | 0.364 | 0.011 | 0.506 | 0.484 | 1000 0.8 |
| M0462 | 512 | exp14 | 2048bit-ECFP4 | GBM | 0.572 | 0.433 | 0.700 | 0.368 | 0.398 | 0.070 | 0.534 | 0.438 | 400 0.6 |
| M0463 | 512 | exp15 | 2048bit-ECFP4 | GBM | 0.572 | 0.429 | 0.718 | 0.340 | 0.379 | 0.061 | 0.529 | 0.438 | 700 0.4 |
| M0464 | 512 | exp16 | 2048bit-ECFP4 | GBM | 0.554 | 0.400 | 0.700 | 0.321 | 0.356 | 0.022 | 0.510 | 0.399 | 300 0.9 |
| M0465 | 512 | exp17 | 2048bit-ECFP4 | GBM | 0.522 | 0.341 | 0.682 | 0.264 | 0.298 | -0.057 | 0.473 | 0.438 | 100 0.9 |
| M0466 | 512 | exp18 | 2048bit-ECFP4 | GBM | 0.525 | 0.366 | 0.653 | 0.321 | 0.342 | -0.027 | 0.487 | 0.477 | 900 0.7 |
| M0467 | 512 | exp19 | 2048bit-ECFP4 | GBM | 0.551 | 0.388 | 0.712 | 0.292 | 0.333 | 0.005 | 0.502 | 0.446 | 400 0.6 |
| M0468 | 512 | exp01 | 2048bit-ECFP4 | RF | 0.540 | 0.376 | 0.688 | 0.302 | 0.335 | -0.010 | 0.495 | 0.383 | 10 |
| M0469 | 512 | exp02 | 2048bit-ECFP4 | RF | 0.514 | 0.354 | 0.635 | 0.321 | 0.337 | -0.045 | 0.478 | 0.410 | 10 |
| M0470 | 512 | exp03 | 2048bit-ECFP4 | RF | 0.558 | 0.409 | 0.694 | 0.340 | 0.371 | 0.035 | 0.517 | 0.408 | 10 |
| M0471 | 512 | exp04 | 2048bit-ECFP4 | RF | 0.558 | 0.405 | 0.706 | 0.321 | 0.358 | 0.028 | 0.513 | 0.410 | 10 |
| M0472 | 512 | exp05 | 2048bit-ECFP4 | RF | 0.558 | 0.392 | 0.735 | 0.274 | 0.322 | 0.010 | 0.504 | 0.383 | 10 |
| M0473 | 512 | exp06 | 2048bit-ECFP4 | RF | 0.551 | 0.400 | 0.682 | 0.340 | 0.367 | 0.023 | 0.511 | 0.387 | 10 |
| M0474 | 512 | exp07 | 2048bit-ECFP4 | RF | 0.533 | 0.354 | 0.700 | 0.264 | 0.303 | -0.039 | 0.482 | 0.352 | 10 |
| M0475 | 512 | exp08 | 2048bit-ECFP4 | RF | 0.529 | 0.357 | 0.682 | 0.283 | 0.316 | -0.037 | 0.483 | 0.370 | 10 |
| M0476 | 512 | exp09 | 2048bit-ECFP4 | RF | 0.587 | 0.457 | 0.706 | 0.396 | 0.424 | 0.105 | 0.551 | 0.408 | 10 |
| M0477 | 512 | exp10 | 2048bit-ECFP4 | RF | 0.576 | 0.429 | 0.741 | 0.311 | 0.361 | 0.057 | 0.526 | 0.427 | 10 |
| M0478 | 512 | exp11 | 2048bit-ECFP4 | RF | 0.536 | 0.372 | 0.682 | 0.302 | 0.333 | -0.017 | 0.492 | 0.437 | 10 |
| M0479 | 512 | exp12 | 2048bit-ECFP4 | RF | 0.601 | 0.476 | 0.741 | 0.377 | 0.421 | 0.125 | 0.559 | 0.377 | 10 |
| M0480 | 512 | exp13 | 2048bit-ECFP4 | RF | 0.565 | 0.417 | 0.712 | 0.330 | 0.368 | 0.044 | 0.521 | 0.471 | 10 |
| M0481 | 512 | exp14 | 2048bit-ECFP4 | RF | 0.551 | 0.395 | 0.694 | 0.321 | 0.354 | 0.016 | 0.507 | 0.421 | 10 |
| M0482 | 512 | exp15 | 2048bit-ECFP4 | RF | 0.558 | 0.395 | 0.729 | 0.283 | 0.330 | 0.014 | 0.506 | 0.384 | 10 |
| M0483 | 512 | exp16 | 2048bit-ECFP4 | RF | 0.587 | 0.450 | 0.741 | 0.340 | 0.387 | 0.087 | 0.540 | 0.365 | 10 |
| M0484 | 512 | exp17 | 2048bit-ECFP4 | RF | 0.522 | 0.329 | 0.700 | 0.236 | 0.275 | -0.070 | 0.468 | 0.355 | 10 |
| M0485 | 512 | exp18 | 2048bit-ECFP4 | RF | 0.500 | 0.326 | 0.635 | 0.283 | 0.303 | -0.084 | 0.459 | 0.418 | 10 |
| M0486 | 512 | exp19 | 2048bit-ECFP4 | RF | 0.536 | 0.355 | 0.712 | 0.255 | 0.297 | -0.036 | 0.483 | 0.377 | 10 |
| M0487 | 512 | exp01 | 2048bit-ECFP4 | DNN2 | 0.565 | 0.426 | 0.682 | 0.377 | 0.400 | 0.061 | 0.530 | 0.401 | 200 100 0.4 |
| M0488 | 512 | exp02 | 2048bit-ECFP4 | DNN2 | 0.518 | 0.352 | 0.653 | 0.302 | 0.325 | -0.047 | 0.478 | 0.400 | 400 80 0.3 |
| M0489 | 512 | exp03 | 2048bit-ECFP4 | DNN2 | 0.572 | 0.433 | 0.700 | 0.368 | 0.398 | 0.070 | 0.534 | 0.443 | 600 160 0.4 |
| M0490 | 512 | exp01 | 2048bit-ECFP4 | DNN3 | 0.569 | 0.434 | 0.671 | 0.406 | 0.420 | 0.077 | 0.538 | 0.407 | 400 100 0.4 |
| M0491 | 512 | exp02 | 2048bit-ECFP4 | DNN3 | 0.522 | 0.362 | 0.647 | 0.321 | 0.340 | -0.033 | 0.484 | 0.412 | 300 140 0.4 |
| M0492 | 512 | exp03 | 2048bit-ECFP4 | DNN3 | 0.572 | 0.443 | 0.653 | 0.443 | 0.443 | 0.096 | 0.548 | 0.455 | 600 160 0.2 |
| M0493 | 512 | exp01 | 1024bit-ECFP6 | KNN | 0.540 | 0.376 | 0.688 | 0.302 | 0.335 | -0.010 | 0.495 | 0.366 | 1 uniform |
| M0494 | 512 | exp02 | 1024bit-ECFP6 | KNN | 0.558 | 0.409 | 0.694 | 0.340 | 0.371 | 0.035 | 0.517 | 0.335 | 1 uniform |
| M0495 | 512 | exp03 | 1024bit-ECFP6 | KNN | 0.583 | 0.454 | 0.688 | 0.415 | 0.433 | 0.105 | 0.551 | 0.388 | 1 uniform |
| M0496 | 512 | exp04 | 1024bit-ECFP6 | KNN | 0.565 | 0.419 | 0.706 | 0.340 | 0.375 | 0.048 | 0.523 | 0.392 | 1 uniform |
| M0497 | 512 | exp05 | 1024bit-ECFP6 | KNN | 0.518 | 0.361 | 0.635 | 0.330 | 0.345 | -0.035 | 0.483 | 0.384 | 1 uniform |
| M0498 | 512 | exp06 | 1024bit-ECFP6 | KNN | 0.533 | 0.390 | 0.624 | 0.387 | 0.389 | 0.010 | 0.506 | 0.354 | 1 uniform |
| M0499 | 512 | exp07 | 1024bit-ECFP6 | KNN | 0.551 | 0.417 | 0.629 | 0.425 | 0.421 | 0.054 | 0.527 | 0.353 | 1 uniform |
| M0500 | 512 | exp08 | 1024bit-ECFP6 | KNN | 0.522 | 0.365 | 0.641 | 0.330 | 0.347 | -0.029 | 0.486 | 0.372 | 1 uniform |
| M0501 | 512 | exp09 | 1024bit-ECFP6 | KNN | 0.594 | 0.464 | 0.735 | 0.368 | 0.411 | 0.109 | 0.551 | 0.376 | 1 uniform |
| M0502 | 512 | exp10 | 1024bit-ECFP6 | KNN | 0.438 | 0.262 | 0.553 | 0.255 | 0.258 | -0.193 | 0.404 | 0.395 | 1 uniform |
| M0503 | 512 | exp11 | 1024bit-ECFP6 | KNN | 0.496 | 0.349 | 0.582 | 0.358 | 0.353 | -0.059 | 0.470 | 0.437 | 1 uniform |
| M0504 | 512 | exp12 | 1024bit-ECFP6 | KNN | 0.558 | 0.418 | 0.665 | 0.387 | 0.402 | 0.052 | 0.526 | 0.382 | 1 uniform |
| M0505 | 512 | exp13 | 1024bit-ECFP6 | KNN | 0.565 | 0.420 | 0.700 | 0.349 | 0.381 | 0.051 | 0.524 | 0.399 | 1 uniform |
| M0506 | 512 | exp14 | 1024bit-ECFP6 | KNN | 0.565 | 0.426 | 0.682 | 0.377 | 0.400 | 0.061 | 0.530 | 0.429 | 1 uniform |
| M0507 | 512 | exp15 | 1024bit-ECFP6 | KNN | 0.562 | 0.423 | 0.671 | 0.387 | 0.404 | 0.058 | 0.529 | 0.363 | 1 uniform |
| M0508 | 512 | exp16 | 1024bit-ECFP6 | KNN | 0.547 | 0.400 | 0.665 | 0.358 | 0.378 | 0.024 | 0.512 | 0.392 | 1 uniform |
| M0509 | 512 | exp17 | 1024bit-ECFP6 | KNN | 0.554 | 0.409 | 0.676 | 0.358 | 0.382 | 0.036 | 0.517 | 0.379 | 1 uniform |
| M0510 | 512 | exp18 | 1024bit-ECFP6 | KNN | 0.507 | 0.344 | 0.629 | 0.311 | 0.327 | -0.061 | 0.470 | 0.415 | 1 uniform |
| M0511 | 512 | exp19 | 1024bit-ECFP6 | KNN | 0.536 | 0.375 | 0.676 | 0.311 | 0.340 | -0.013 | 0.494 | 0.371 | 1 uniform |
| M0512 | 512 | exp01 | 1024bit-ECFP6 | SVM | 0.612 | 0.333 | 0.988 | 0.009 | 0.018 | -0.011 | 0.498 | 0.064 | 100000.0 0.01 |
| M0513 | 512 | exp02 | 1024bit-ECFP6 | SVM | 0.616 | 0.000 | 1.000 | 0.000 | 0.000 | 0.000 | 0.500 | 0.000 | 500 0.0005 |
| M0514 | 512 | exp03 | 1024bit-ECFP6 | SVM | 0.623 | 1.000 | 1.000 | 0.019 | 0.037 | 0.108 | 0.509 | 0.058 | 100000.0 5e-05 |
| M0515 | 512 | exp04 | 1024bit-ECFP6 | SVM | 0.612 | 0.000 | 0.994 | 0.000 | 0.000 | -0.048 | 0.497 | 0.071 | 100000.0 0.01 |
| M0516 | 512 | exp05 | 1024bit-ECFP6 | SVM | 0.612 | 0.000 | 0.994 | 0.000 | 0.000 | -0.048 | 0.497 | 0.013 | 100000.0 0.01 |
| M0517 | 512 | exp06 | 1024bit-ECFP6 | SVM | 0.616 | 0.000 | 1.000 | 0.000 | 0.000 | 0.000 | 0.500 | 0.000 | 50000.0 1e-05 |
| M0518 | 512 | exp07 | 1024bit-ECFP6 | SVM | 0.605 | 0.000 | 0.982 | 0.000 | 0.000 | -0.083 | 0.491 | 0.099 | 100000.0 0.0001 |
| M0519 | 512 | exp08 | 1024bit-ECFP6 | SVM | 0.616 | 0.000 | 1.000 | 0.000 | 0.000 | 0.000 | 0.500 | 0.000 | 500 0.01 |
| M0520 | 512 | exp09 | 1024bit-ECFP6 | SVM | 0.649 | 0.846 | 0.988 | 0.104 | 0.185 | 0.211 | 0.546 | 0.068 | 5 0.1 |
| M0521 | 512 | exp10 | 1024bit-ECFP6 | SVM | 0.623 | 1.000 | 1.000 | 0.019 | 0.037 | 0.108 | 0.509 | 0.030 | 5000.0 0.05 |
| M0522 | 512 | exp11 | 1024bit-ECFP6 | SVM | 0.620 | 1.000 | 1.000 | 0.009 | 0.019 | 0.076 | 0.504 | 0.079 | 50 0.005 |
| M0523 | 512 | exp12 | 1024bit-ECFP6 | SVM | 0.612 | 0.000 | 0.994 | 0.000 | 0.000 | -0.048 | 0.497 | 0.000 | 1000.0 0.001 |
| M0524 | 512 | exp13 | 1024bit-ECFP6 | SVM | 0.616 | 0.000 | 1.000 | 0.000 | 0.000 | 0.000 | 0.500 | 0.038 | 500 0.01 |
| M0525 | 512 | exp14 | 1024bit-ECFP6 | SVM | 0.616 | 0.500 | 0.994 | 0.009 | 0.019 | 0.020 | 0.501 | 0.057 | 1000.0 5e-05 |
| M0526 | 512 | exp15 | 1024bit-ECFP6 | SVM | 0.616 | 0.000 | 1.000 | 0.000 | 0.000 | 0.000 | 0.500 | 0.023 | 100000.0 5e-05 |
| M0527 | 512 | exp16 | 1024bit-ECFP6 | SVM | 0.616 | 0.000 | 1.000 | 0.000 | 0.000 | 0.000 | 0.500 | 0.000 | 1 0.005 |
| M0528 | 512 | exp17 | 1024bit-ECFP6 | SVM | 0.612 | 0.000 | 0.994 | 0.000 | 0.000 | -0.048 | 0.497 | 0.037 | 100000.0 0.0005 |
| M0529 | 512 | exp18 | 1024bit-ECFP6 | SVM | 0.616 | 0.500 | 0.988 | 0.019 | 0.036 | 0.029 | 0.503 | 0.049 | 500 0.05 |
| M0530 | 512 | exp19 | 1024bit-ECFP6 | SVM | 0.616 | 0.000 | 1.000 | 0.000 | 0.000 | 0.000 | 0.500 | 0.038 | 10 0.01 |
| M0531 | 512 | exp01 | 1024bit-ECFP6 | GBM | 0.475 | 0.290 | 0.612 | 0.255 | 0.271 | -0.137 | 0.433 | 0.430 | 900 0.9 |
| M0532 | 512 | exp02 | 1024bit-ECFP6 | GBM | 0.493 | 0.323 | 0.618 | 0.292 | 0.307 | -0.092 | 0.455 | 0.392 | 900 0.6 |
| M0533 | 512 | exp03 | 1024bit-ECFP6 | GBM | 0.558 | 0.402 | 0.712 | 0.311 | 0.351 | 0.025 | 0.511 | 0.442 | 600 0.9 |
| M0534 | 512 | exp04 | 1024bit-ECFP6 | GBM | 0.504 | 0.337 | 0.629 | 0.302 | 0.318 | -0.070 | 0.466 | 0.440 | 600 0.7 |
| M0535 | 512 | exp05 | 1024bit-ECFP6 | GBM | 0.457 | 0.284 | 0.571 | 0.274 | 0.279 | -0.157 | 0.422 | 0.399 | 1000 0.8 |
| M0536 | 512 | exp06 | 1024bit-ECFP6 | GBM | 0.496 | 0.330 | 0.618 | 0.302 | 0.315 | -0.082 | 0.460 | 0.363 | 900 0.9 |
| M0537 | 512 | exp07 | 1024bit-ECFP6 | GBM | 0.583 | 0.447 | 0.724 | 0.358 | 0.398 | 0.086 | 0.541 | 0.415 | 900 0.8 |
| M0538 | 512 | exp08 | 1024bit-ECFP6 | GBM | 0.507 | 0.330 | 0.653 | 0.274 | 0.299 | -0.077 | 0.464 | 0.395 | 1000 0.9 |
| M0539 | 512 | exp09 | 1024bit-ECFP6 | GBM | 0.598 | 0.473 | 0.718 | 0.406 | 0.437 | 0.128 | 0.562 | 0.429 | 900 0.4 |
| M0540 | 512 | exp10 | 1024bit-ECFP6 | GBM | 0.547 | 0.386 | 0.700 | 0.302 | 0.339 | 0.002 | 0.501 | 0.441 | 900 0.5 |
| M0541 | 512 | exp11 | 1024bit-ECFP6 | GBM | 0.569 | 0.430 | 0.688 | 0.377 | 0.402 | 0.067 | 0.532 | 0.413 | 600 0.6 |
| M0542 | 512 | exp12 | 1024bit-ECFP6 | GBM | 0.576 | 0.440 | 0.700 | 0.377 | 0.406 | 0.080 | 0.538 | 0.428 | 200 0.8 |
| M0543 | 512 | exp13 | 1024bit-ECFP6 | GBM | 0.536 | 0.362 | 0.700 | 0.274 | 0.312 | -0.028 | 0.487 | 0.459 | 1000 0.6 |
| M0544 | 512 | exp14 | 1024bit-ECFP6 | GBM | 0.507 | 0.337 | 0.641 | 0.292 | 0.313 | -0.068 | 0.467 | 0.426 | 500 0.9 |
| M0545 | 512 | exp15 | 1024bit-ECFP6 | GBM | 0.536 | 0.378 | 0.671 | 0.321 | 0.347 | -0.009 | 0.496 | 0.397 | 200 0.9 |
| M0546 | 512 | exp16 | 1024bit-ECFP6 | GBM | 0.562 | 0.412 | 0.706 | 0.330 | 0.366 | 0.038 | 0.518 | 0.386 | 1000 0.9 |
| M0547 | 512 | exp17 | 1024bit-ECFP6 | GBM | 0.482 | 0.287 | 0.635 | 0.236 | 0.259 | -0.135 | 0.435 | 0.377 | 900 0.8 |
| M0548 | 512 | exp18 | 1024bit-ECFP6 | GBM | 0.525 | 0.366 | 0.653 | 0.321 | 0.342 | -0.027 | 0.487 | 0.439 | 300 0.9 |
| M0549 | 512 | exp19 | 1024bit-ECFP6 | GBM | 0.551 | 0.395 | 0.694 | 0.321 | 0.354 | 0.016 | 0.507 | 0.419 | 600 0.9 |
| M0550 | 512 | exp01 | 1024bit-ECFP6 | RF | 0.496 | 0.306 | 0.653 | 0.245 | 0.272 | -0.107 | 0.449 | 0.416 | 10 |
| M0551 | 512 | exp02 | 1024bit-ECFP6 | RF | 0.482 | 0.313 | 0.600 | 0.292 | 0.302 | -0.109 | 0.446 | 0.387 | 10 |
| M0552 | 512 | exp03 | 1024bit-ECFP6 | RF | 0.529 | 0.372 | 0.653 | 0.330 | 0.350 | -0.017 | 0.492 | 0.437 | 10 |
| M0553 | 512 | exp04 | 1024bit-ECFP6 | RF | 0.478 | 0.306 | 0.600 | 0.283 | 0.294 | -0.119 | 0.442 | 0.414 | 10 |
| M0554 | 512 | exp05 | 1024bit-ECFP6 | RF | 0.514 | 0.354 | 0.635 | 0.321 | 0.337 | -0.045 | 0.478 | 0.383 | 10 |
| M0555 | 512 | exp06 | 1024bit-ECFP6 | RF | 0.580 | 0.440 | 0.724 | 0.349 | 0.389 | 0.077 | 0.536 | 0.393 | 10 |
| M0556 | 512 | exp07 | 1024bit-ECFP6 | RF | 0.580 | 0.451 | 0.671 | 0.434 | 0.442 | 0.105 | 0.552 | 0.390 | 10 |
| M0557 | 512 | exp08 | 1024bit-ECFP6 | RF | 0.496 | 0.319 | 0.635 | 0.274 | 0.294 | -0.094 | 0.455 | 0.374 | 10 |
| M0558 | 512 | exp09 | 1024bit-ECFP6 | RF | 0.591 | 0.462 | 0.712 | 0.396 | 0.426 | 0.112 | 0.554 | 0.424 | 10 |
| M0559 | 512 | exp10 | 1024bit-ECFP6 | RF | 0.583 | 0.421 | 0.806 | 0.226 | 0.294 | 0.039 | 0.516 | 0.377 | 100 |
| M0560 | 512 | exp11 | 1024bit-ECFP6 | RF | 0.518 | 0.361 | 0.635 | 0.330 | 0.345 | -0.035 | 0.483 | 0.401 | 10 |
| M0561 | 512 | exp12 | 1024bit-ECFP6 | RF | 0.533 | 0.388 | 0.629 | 0.377 | 0.383 | 0.007 | 0.503 | 0.444 | 10 |
| M0562 | 512 | exp13 | 1024bit-ECFP6 | RF | 0.536 | 0.372 | 0.682 | 0.302 | 0.333 | -0.017 | 0.492 | 0.411 | 10 |
| M0563 | 512 | exp14 | 1024bit-ECFP6 | RF | 0.587 | 0.441 | 0.776 | 0.283 | 0.345 | 0.067 | 0.529 | 0.362 | 100 |
| M0564 | 512 | exp15 | 1024bit-ECFP6 | RF | 0.536 | 0.378 | 0.671 | 0.321 | 0.347 | -0.009 | 0.496 | 0.392 | 10 |
| M0565 | 512 | exp16 | 1024bit-ECFP6 | RF | 0.540 | 0.382 | 0.676 | 0.321 | 0.349 | -0.003 | 0.499 | 0.340 | 10 |
| M0566 | 512 | exp17 | 1024bit-ECFP6 | RF | 0.496 | 0.291 | 0.671 | 0.217 | 0.249 | -0.121 | 0.444 | 0.342 | 10 |
| M0567 | 512 | exp18 | 1024bit-ECFP6 | RF | 0.482 | 0.320 | 0.588 | 0.311 | 0.316 | -0.101 | 0.450 | 0.438 | 10 |
| M0568 | 512 | exp19 | 1024bit-ECFP6 | RF | 0.543 | 0.381 | 0.694 | 0.302 | 0.337 | -0.004 | 0.498 | 0.356 | 10 |
| M0569 | 512 | exp01 | 1024bit-ECFP6 | DNN2 | 0.507 | 0.340 | 0.635 | 0.302 | 0.320 | -0.064 | 0.469 | 0.424 | 600 60 0.2 |
| M0570 | 512 | exp02 | 1024bit-ECFP6 | DNN2 | 0.493 | 0.323 | 0.618 | 0.292 | 0.307 | -0.092 | 0.455 | 0.377 | 400 80 0.2 |
| M0571 | 512 | exp03 | 1024bit-ECFP6 | DNN2 | 0.562 | 0.414 | 0.700 | 0.340 | 0.373 | 0.041 | 0.520 | 0.443 | 300 80 0.2 |
| M0572 | 512 | exp01 | 1024bit-ECFP6 | DNN3 | 0.522 | 0.370 | 0.629 | 0.349 | 0.359 | -0.022 | 0.489 | 0.432 | 300 60 0.5 |
| M0573 | 512 | exp02 | 1024bit-ECFP6 | DNN3 | 0.482 | 0.317 | 0.594 | 0.302 | 0.309 | -0.105 | 0.448 | 0.386 | 500 60 0.3 |
| M0574 | 512 | exp03 | 1024bit-ECFP6 | DNN3 | 0.543 | 0.389 | 0.676 | 0.330 | 0.357 | 0.007 | 0.503 | 0.436 | 400 160 0.5 |
| M0575 | 512 | exp01 | 2048bit-ECFP6 | KNN | 0.583 | 0.442 | 0.747 | 0.321 | 0.372 | 0.074 | 0.534 | 0.372 | 1 uniform |
| M0576 | 512 | exp02 | 2048bit-ECFP6 | KNN | 0.547 | 0.386 | 0.700 | 0.302 | 0.339 | 0.002 | 0.501 | 0.309 | 1 uniform |
| M0577 | 512 | exp03 | 2048bit-ECFP6 | KNN | 0.587 | 0.457 | 0.706 | 0.396 | 0.424 | 0.105 | 0.551 | 0.391 | 1 uniform |
| M0578 | 512 | exp04 | 2048bit-ECFP6 | KNN | 0.525 | 0.353 | 0.676 | 0.283 | 0.314 | -0.043 | 0.480 | 0.398 | 1 uniform |
| M0579 | 512 | exp05 | 2048bit-ECFP6 | KNN | 0.551 | 0.388 | 0.712 | 0.292 | 0.333 | 0.005 | 0.502 | 0.371 | 1 uniform |
| M0580 | 512 | exp06 | 2048bit-ECFP6 | KNN | 0.569 | 0.427 | 0.700 | 0.358 | 0.390 | 0.061 | 0.529 | 0.329 | 1 uniform |
| M0581 | 512 | exp07 | 2048bit-ECFP6 | KNN | 0.504 | 0.350 | 0.606 | 0.340 | 0.344 | -0.055 | 0.473 | 0.331 | 1 uniform |
| M0582 | 512 | exp08 | 2048bit-ECFP6 | KNN | 0.525 | 0.363 | 0.659 | 0.311 | 0.335 | -0.031 | 0.485 | 0.348 | 1 uniform |
| M0583 | 512 | exp09 | 2048bit-ECFP6 | KNN | 0.609 | 0.487 | 0.765 | 0.358 | 0.413 | 0.133 | 0.561 | 0.365 | 1 uniform |
| M0584 | 512 | exp10 | 2048bit-ECFP6 | KNN | 0.536 | 0.378 | 0.671 | 0.321 | 0.347 | -0.009 | 0.496 | 0.412 | 1 uniform |
| M0585 | 512 | exp11 | 2048bit-ECFP6 | KNN | 0.507 | 0.344 | 0.629 | 0.311 | 0.327 | -0.061 | 0.470 | 0.413 | 1 uniform |
| M0586 | 512 | exp12 | 2048bit-ECFP6 | KNN | 0.551 | 0.404 | 0.671 | 0.358 | 0.380 | 0.030 | 0.514 | 0.365 | 1 uniform |
| M0587 | 512 | exp13 | 2048bit-ECFP6 | KNN | 0.576 | 0.432 | 0.729 | 0.330 | 0.374 | 0.064 | 0.529 | 0.396 | 1 uniform |
| M0588 | 512 | exp14 | 2048bit-ECFP6 | KNN | 0.554 | 0.395 | 0.712 | 0.302 | 0.342 | 0.015 | 0.507 | 0.412 | 1 uniform |
| M0589 | 512 | exp15 | 2048bit-ECFP6 | KNN | 0.525 | 0.346 | 0.688 | 0.264 | 0.299 | -0.051 | 0.476 | 0.333 | 1 uniform |
| M0590 | 512 | exp16 | 2048bit-ECFP6 | KNN | 0.565 | 0.417 | 0.712 | 0.330 | 0.368 | 0.044 | 0.521 | 0.346 | 1 uniform |
| M0591 | 512 | exp17 | 2048bit-ECFP6 | KNN | 0.554 | 0.395 | 0.712 | 0.302 | 0.342 | 0.015 | 0.507 | 0.362 | 1 uniform |
| M0592 | 512 | exp18 | 2048bit-ECFP6 | KNN | 0.533 | 0.354 | 0.700 | 0.264 | 0.303 | -0.039 | 0.482 | 0.386 | 1 uniform |
| M0593 | 512 | exp19 | 2048bit-ECFP6 | KNN | 0.598 | 0.468 | 0.759 | 0.340 | 0.393 | 0.107 | 0.549 | 0.361 | 1 uniform |
| M0594 | 512 | exp01 | 2048bit-ECFP6 | SVM | 0.601 | 0.300 | 0.959 | 0.028 | 0.052 | -0.034 | 0.493 | 0.193 | 100000.0 0.01 |
| M0595 | 512 | exp02 | 2048bit-ECFP6 | SVM | 0.612 | 0.333 | 0.988 | 0.009 | 0.018 | -0.011 | 0.498 | 0.028 | 5 0.005 |
| M0596 | 512 | exp03 | 2048bit-ECFP6 | SVM | 0.612 | 0.455 | 0.965 | 0.047 | 0.085 | 0.030 | 0.506 | 0.177 | 10 0.01 |
| M0597 | 512 | exp04 | 2048bit-ECFP6 | SVM | 0.612 | 0.474 | 0.941 | 0.085 | 0.144 | 0.050 | 0.513 | 0.150 | 5 0.05 |
| M0598 | 512 | exp05 | 2048bit-ECFP6 | SVM | 0.623 | 0.562 | 0.959 | 0.085 | 0.148 | 0.091 | 0.522 | 0.164 | 50000.0 0.05 |
| M0599 | 512 | exp06 | 2048bit-ECFP6 | SVM | 0.641 | 0.733 | 0.976 | 0.104 | 0.182 | 0.172 | 0.540 | 0.108 | 5 0.01 |
| M0600 | 512 | exp07 | 2048bit-ECFP6 | SVM | 0.612 | 0.429 | 0.976 | 0.028 | 0.053 | 0.015 | 0.502 | 0.110 | 100000.0 0.0001 |
| M0601 | 512 | exp08 | 2048bit-ECFP6 | SVM | 0.572 | 0.250 | 0.894 | 0.057 | 0.092 | -0.085 | 0.476 | 0.116 | 10 0.005 |
| M0602 | 512 | exp09 | 2048bit-ECFP6 | SVM | 0.656 | 0.720 | 0.959 | 0.170 | 0.275 | 0.218 | 0.565 | 0.220 | 100 0.05 |
| M0603 | 512 | exp10 | 2048bit-ECFP6 | SVM | 0.598 | 0.447 | 0.847 | 0.198 | 0.275 | 0.058 | 0.522 | 0.299 | 10 0.01 |
| M0604 | 512 | exp11 | 2048bit-ECFP6 | SVM | 0.583 | 0.263 | 0.918 | 0.047 | 0.080 | -0.068 | 0.483 | 0.275 | 100000.0 0.0001 |
| M0605 | 512 | exp12 | 2048bit-ECFP6 | SVM | 0.601 | 0.429 | 0.906 | 0.113 | 0.179 | 0.031 | 0.510 | 0.226 | 100000.0 0.01 |
| M0606 | 512 | exp13 | 2048bit-ECFP6 | SVM | 0.591 | 0.360 | 0.906 | 0.085 | 0.137 | -0.016 | 0.495 | 0.309 | 500 0.005 |
| M0607 | 512 | exp14 | 2048bit-ECFP6 | SVM | 0.616 | 0.500 | 0.929 | 0.113 | 0.185 | 0.074 | 0.521 | 0.194 | 1 0.05 |
| M0608 | 512 | exp15 | 2048bit-ECFP6 | SVM | 0.572 | 0.125 | 0.918 | 0.019 | 0.033 | -0.132 | 0.469 | 0.166 | 10000.0 0.001 |
| M0609 | 512 | exp16 | 2048bit-ECFP6 | SVM | 0.623 | 1.000 | 1.000 | 0.019 | 0.037 | 0.108 | 0.509 | 0.048 | 100000.0 0.0001 |
| M0610 | 512 | exp17 | 2048bit-ECFP6 | SVM | 0.587 | 0.250 | 0.929 | 0.038 | 0.066 | -0.068 | 0.484 | 0.159 | 1000.0 0.05 |
| M0611 | 512 | exp18 | 2048bit-ECFP6 | SVM | 0.583 | 0.355 | 0.882 | 0.104 | 0.161 | -0.021 | 0.493 | 0.221 | 50 0.05 |
| M0612 | 512 | exp19 | 2048bit-ECFP6 | SVM | 0.634 | 0.692 | 0.976 | 0.085 | 0.151 | 0.141 | 0.530 | 0.160 | 1 0.1 |
| M0613 | 512 | exp01 | 2048bit-ECFP6 | GBM | 0.551 | 0.359 | 0.759 | 0.217 | 0.271 | -0.028 | 0.488 | 0.434 | 50 0.9 |
| M0614 | 512 | exp02 | 2048bit-ECFP6 | GBM | 0.547 | 0.396 | 0.676 | 0.340 | 0.365 | 0.017 | 0.508 | 0.440 | 200 0.7 |
| M0615 | 512 | exp03 | 2048bit-ECFP6 | GBM | 0.540 | 0.367 | 0.706 | 0.274 | 0.314 | -0.022 | 0.490 | 0.458 | 600 0.7 |
| M0616 | 512 | exp04 | 2048bit-ECFP6 | GBM | 0.486 | 0.291 | 0.641 | 0.236 | 0.260 | -0.129 | 0.439 | 0.439 | 600 0.8 |
| M0617 | 512 | exp05 | 2048bit-ECFP6 | GBM | 0.514 | 0.329 | 0.676 | 0.255 | 0.287 | -0.073 | 0.466 | 0.435 | 700 0.8 |
| M0618 | 512 | exp06 | 2048bit-ECFP6 | GBM | 0.540 | 0.376 | 0.688 | 0.302 | 0.335 | -0.010 | 0.495 | 0.412 | 1000 0.8 |
| M0619 | 512 | exp07 | 2048bit-ECFP6 | GBM | 0.558 | 0.405 | 0.706 | 0.321 | 0.358 | 0.028 | 0.513 | 0.394 | 1000 0.8 |
| M0620 | 512 | exp08 | 2048bit-ECFP6 | GBM | 0.507 | 0.326 | 0.659 | 0.264 | 0.292 | -0.081 | 0.462 | 0.443 | 900 0.4 |
| M0621 | 512 | exp09 | 2048bit-ECFP6 | GBM | 0.583 | 0.454 | 0.688 | 0.415 | 0.433 | 0.105 | 0.551 | 0.434 | 1000 0.8 |
| M0622 | 512 | exp10 | 2048bit-ECFP6 | GBM | 0.554 | 0.412 | 0.665 | 0.377 | 0.394 | 0.043 | 0.521 | 0.474 | 300 0.7 |
| M0623 | 512 | exp11 | 2048bit-ECFP6 | GBM | 0.547 | 0.393 | 0.682 | 0.330 | 0.359 | 0.013 | 0.506 | 0.459 | 400 0.9 |
| M0624 | 512 | exp12 | 2048bit-ECFP6 | GBM | 0.547 | 0.411 | 0.629 | 0.415 | 0.413 | 0.044 | 0.522 | 0.451 | 800 0.7 |
| M0625 | 512 | exp13 | 2048bit-ECFP6 | GBM | 0.554 | 0.398 | 0.706 | 0.311 | 0.349 | 0.018 | 0.508 | 0.492 | 800 0.7 |
| M0626 | 512 | exp14 | 2048bit-ECFP6 | GBM | 0.547 | 0.400 | 0.665 | 0.358 | 0.378 | 0.024 | 0.512 | 0.448 | 1000 0.8 |
| M0627 | 512 | exp15 | 2048bit-ECFP6 | GBM | 0.507 | 0.350 | 0.618 | 0.330 | 0.340 | -0.053 | 0.474 | 0.468 | 1000 0.5 |
| M0628 | 512 | exp16 | 2048bit-ECFP6 | GBM | 0.558 | 0.409 | 0.694 | 0.340 | 0.371 | 0.035 | 0.517 | 0.387 | 400 0.6 |
| M0629 | 512 | exp17 | 2048bit-ECFP6 | GBM | 0.543 | 0.375 | 0.706 | 0.283 | 0.323 | -0.012 | 0.494 | 0.438 | 800 0.4 |
| M0630 | 512 | exp18 | 2048bit-ECFP6 | GBM | 0.551 | 0.395 | 0.694 | 0.321 | 0.354 | 0.016 | 0.507 | 0.424 | 500 0.6 |
| M0631 | 512 | exp19 | 2048bit-ECFP6 | GBM | 0.536 | 0.375 | 0.676 | 0.311 | 0.340 | -0.013 | 0.494 | 0.454 | 400 0.2 |
| M0632 | 512 | exp01 | 2048bit-ECFP6 | RF | 0.486 | 0.263 | 0.671 | 0.189 | 0.220 | -0.153 | 0.430 | 0.418 | 10 |
| M0633 | 512 | exp02 | 2048bit-ECFP6 | RF | 0.507 | 0.350 | 0.618 | 0.330 | 0.340 | -0.053 | 0.474 | 0.412 | 10 |
| M0634 | 512 | exp03 | 2048bit-ECFP6 | RF | 0.514 | 0.311 | 0.700 | 0.217 | 0.256 | -0.091 | 0.458 | 0.436 | 10 |
| M0635 | 512 | exp04 | 2048bit-ECFP6 | RF | 0.496 | 0.319 | 0.635 | 0.274 | 0.294 | -0.094 | 0.455 | 0.442 | 10 |
| M0636 | 512 | exp05 | 2048bit-ECFP6 | RF | 0.533 | 0.351 | 0.706 | 0.255 | 0.295 | -0.043 | 0.480 | 0.435 | 10 |
| M0637 | 512 | exp06 | 2048bit-ECFP6 | RF | 0.543 | 0.389 | 0.676 | 0.330 | 0.357 | 0.007 | 0.503 | 0.405 | 10 |
| M0638 | 512 | exp07 | 2048bit-ECFP6 | RF | 0.533 | 0.361 | 0.688 | 0.283 | 0.317 | -0.030 | 0.485 | 0.370 | 10 |
| M0639 | 512 | exp08 | 2048bit-ECFP6 | RF | 0.489 | 0.294 | 0.647 | 0.236 | 0.262 | -0.123 | 0.442 | 0.396 | 10 |
| M0640 | 512 | exp09 | 2048bit-ECFP6 | RF | 0.601 | 0.478 | 0.718 | 0.415 | 0.444 | 0.137 | 0.567 | 0.416 | 10 |
| M0641 | 512 | exp10 | 2048bit-ECFP6 | RF | 0.583 | 0.444 | 0.735 | 0.340 | 0.385 | 0.080 | 0.537 | 0.417 | 10 |
| M0642 | 512 | exp11 | 2048bit-ECFP6 | RF | 0.551 | 0.398 | 0.688 | 0.330 | 0.361 | 0.019 | 0.509 | 0.428 | 10 |
| M0643 | 512 | exp12 | 2048bit-ECFP6 | RF | 0.583 | 0.454 | 0.688 | 0.415 | 0.433 | 0.105 | 0.551 | 0.413 | 10 |
| M0644 | 512 | exp13 | 2048bit-ECFP6 | RF | 0.558 | 0.392 | 0.735 | 0.274 | 0.322 | 0.010 | 0.504 | 0.473 | 10 |
| M0645 | 512 | exp14 | 2048bit-ECFP6 | RF | 0.514 | 0.337 | 0.665 | 0.274 | 0.302 | -0.065 | 0.470 | 0.431 | 10 |
| M0646 | 512 | exp15 | 2048bit-ECFP6 | RF | 0.514 | 0.341 | 0.659 | 0.283 | 0.309 | -0.061 | 0.471 | 0.415 | 10 |
| M0647 | 512 | exp16 | 2048bit-ECFP6 | RF | 0.514 | 0.325 | 0.682 | 0.245 | 0.280 | -0.078 | 0.464 | 0.357 | 10 |
| M0648 | 512 | exp17 | 2048bit-ECFP6 | RF | 0.543 | 0.357 | 0.735 | 0.236 | 0.284 | -0.032 | 0.485 | 0.376 | 10 |
| M0649 | 512 | exp18 | 2048bit-ECFP6 | RF | 0.500 | 0.300 | 0.671 | 0.226 | 0.258 | -0.110 | 0.449 | 0.441 | 10 |
| M0650 | 512 | exp19 | 2048bit-ECFP6 | RF | 0.547 | 0.388 | 0.694 | 0.311 | 0.346 | 0.006 | 0.502 | 0.435 | 10 |
| M0651 | 512 | exp01 | 2048bit-ECFP6 | DNN2 | 0.533 | 0.381 | 0.647 | 0.349 | 0.365 | -0.004 | 0.498 | 0.436 | 300 140 0.5 |
| M0652 | 512 | exp02 | 2048bit-ECFP6 | DNN2 | 0.475 | 0.290 | 0.612 | 0.255 | 0.271 | -0.137 | 0.433 | 0.417 | 200 60 0.4 |
| M0653 | 512 | exp03 | 2048bit-ECFP6 | DNN2 | 0.543 | 0.381 | 0.694 | 0.302 | 0.337 | -0.004 | 0.498 | 0.479 | 400 60 0.1 |
| M0654 | 512 | exp01 | 2048bit-ECFP6 | DNN3 | 0.529 | 0.378 | 0.641 | 0.349 | 0.363 | -0.010 | 0.495 | 0.447 | 600 140 0.4 |
| M0655 | 512 | exp02 | 2048bit-ECFP6 | DNN3 | 0.507 | 0.340 | 0.635 | 0.302 | 0.320 | -0.064 | 0.469 | 0.412 | 400 120 0.5 |
| M0656 | 512 | exp03 | 2048bit-ECFP6 | DNN3 | 0.536 | 0.375 | 0.676 | 0.311 | 0.340 | -0.013 | 0.494 | 0.475 | 500 60 0.4 |
| M0657 | 256 | exp01 | 1024bit-ECFP4 | KNN | 0.569 | 0.434 | 0.671 | 0.406 | 0.420 | 0.077 | 0.538 | 0.376 | 1 uniform |
| M0658 | 256 | exp02 | 1024bit-ECFP4 | KNN | 0.529 | 0.370 | 0.659 | 0.321 | 0.343 | -0.021 | 0.490 | 0.328 | 1 uniform |
| M0659 | 256 | exp03 | 1024bit-ECFP4 | KNN | 0.594 | 0.469 | 0.694 | 0.434 | 0.451 | 0.130 | 0.564 | 0.394 | 1 uniform |
| M0660 | 256 | exp04 | 1024bit-ECFP4 | KNN | 0.493 | 0.323 | 0.618 | 0.292 | 0.307 | -0.092 | 0.455 | 0.413 | 1 uniform |
| M0661 | 256 | exp05 | 1024bit-ECFP4 | KNN | 0.489 | 0.294 | 0.647 | 0.236 | 0.262 | -0.123 | 0.442 | 0.384 | 1 uniform |
| M0662 | 256 | exp06 | 1024bit-ECFP4 | KNN | 0.525 | 0.385 | 0.606 | 0.396 | 0.391 | 0.002 | 0.501 | 0.382 | 1 uniform |
| M0663 | 256 | exp07 | 1024bit-ECFP4 | KNN | 0.525 | 0.383 | 0.612 | 0.387 | 0.385 | -0.001 | 0.499 | 0.335 | 1 uniform |
| M0664 | 256 | exp08 | 1024bit-ECFP4 | KNN | 0.500 | 0.340 | 0.612 | 0.321 | 0.330 | -0.068 | 0.467 | 0.366 | 1 uniform |
| M0665 | 256 | exp09 | 1024bit-ECFP4 | KNN | 0.623 | 0.511 | 0.747 | 0.425 | 0.464 | 0.179 | 0.586 | 0.395 | 1 uniform |
| M0666 | 256 | exp10 | 1024bit-ECFP4 | KNN | 0.533 | 0.374 | 0.665 | 0.321 | 0.345 | -0.015 | 0.493 | 0.415 | 1 uniform |
| M0667 | 256 | exp11 | 1024bit-ECFP4 | KNN | 0.514 | 0.365 | 0.612 | 0.358 | 0.362 | -0.030 | 0.485 | 0.423 | 1 uniform |
| M0668 | 256 | exp12 | 1024bit-ECFP4 | KNN | 0.587 | 0.460 | 0.682 | 0.434 | 0.447 | 0.118 | 0.558 | 0.385 | 1 uniform |
| M0669 | 256 | exp13 | 1024bit-ECFP4 | KNN | 0.569 | 0.430 | 0.688 | 0.377 | 0.402 | 0.067 | 0.532 | 0.392 | 1 uniform |
| M0670 | 256 | exp14 | 1024bit-ECFP4 | KNN | 0.518 | 0.371 | 0.612 | 0.368 | 0.370 | -0.020 | 0.490 | 0.382 | 1 uniform |
| M0671 | 256 | exp15 | 1024bit-ECFP4 | KNN | 0.525 | 0.371 | 0.641 | 0.340 | 0.355 | -0.020 | 0.491 | 0.367 | 1 uniform |
| M0672 | 256 | exp16 | 1024bit-ECFP4 | KNN | 0.565 | 0.429 | 0.671 | 0.396 | 0.412 | 0.068 | 0.534 | 0.380 | 1 uniform |
| M0673 | 256 | exp17 | 1024bit-ECFP4 | KNN | 0.533 | 0.386 | 0.635 | 0.368 | 0.377 | 0.003 | 0.502 | 0.360 | 1 uniform |
| M0674 | 256 | exp18 | 1024bit-ECFP4 | KNN | 0.493 | 0.340 | 0.588 | 0.340 | 0.340 | -0.072 | 0.464 | 0.413 | 1 uniform |
| M0675 | 256 | exp19 | 1024bit-ECFP4 | KNN | 0.514 | 0.344 | 0.653 | 0.292 | 0.316 | -0.057 | 0.473 | 0.397 | 1 uniform |
| M0676 | 256 | exp01 | 1024bit-ECFP4 | SVM | 0.616 | 0.500 | 0.994 | 0.009 | 0.019 | 0.020 | 0.501 | 0.112 | 100 0.005 |
| M0677 | 256 | exp02 | 1024bit-ECFP4 | SVM | 0.616 | 0.000 | 1.000 | 0.000 | 0.000 | 0.000 | 0.500 | 0.000 | 10000.0 5e-05 |
| M0678 | 256 | exp03 | 1024bit-ECFP4 | SVM | 0.609 | 0.000 | 0.988 | 0.000 | 0.000 | -0.067 | 0.494 | 0.033 | 50 0.005 |
| M0679 | 256 | exp04 | 1024bit-ECFP4 | SVM | 0.612 | 0.000 | 0.994 | 0.000 | 0.000 | -0.048 | 0.497 | 0.042 | 5000.0 0.005 |
| M0680 | 256 | exp05 | 1024bit-ECFP4 | SVM | 0.616 | 0.500 | 0.994 | 0.009 | 0.019 | 0.020 | 0.501 | 0.074 | 500 0.0005 |
| M0681 | 256 | exp06 | 1024bit-ECFP4 | SVM | 0.616 | 0.000 | 1.000 | 0.000 | 0.000 | 0.000 | 0.500 | 0.051 | 10000.0 0.0001 |
| M0682 | 256 | exp07 | 1024bit-ECFP4 | SVM | 0.616 | 0.000 | 1.000 | 0.000 | 0.000 | 0.000 | 0.500 | 0.004 | 100000.0 0.01 |
| M0683 | 256 | exp08 | 1024bit-ECFP4 | SVM | 0.605 | 0.412 | 0.941 | 0.066 | 0.114 | 0.015 | 0.503 | 0.135 | 10000.0 5e-05 |
| M0684 | 256 | exp09 | 1024bit-ECFP4 | SVM | 0.620 | 0.600 | 0.988 | 0.028 | 0.054 | 0.060 | 0.508 | 0.084 | 50 0.01 |
| M0685 | 256 | exp10 | 1024bit-ECFP4 | SVM | 0.583 | 0.000 | 0.947 | 0.000 | 0.000 | -0.145 | 0.473 | 0.123 | 100000.0 1e-05 |
| M0686 | 256 | exp11 | 1024bit-ECFP4 | SVM | 0.572 | 0.000 | 0.929 | 0.000 | 0.000 | -0.168 | 0.465 | 0.164 | 500 0.005 |
| M0687 | 256 | exp12 | 1024bit-ECFP4 | SVM | 0.616 | 0.000 | 1.000 | 0.000 | 0.000 | 0.000 | 0.500 | 0.009 | 10000.0 0.001 |
| M0688 | 256 | exp13 | 1024bit-ECFP4 | SVM | 0.601 | 0.000 | 0.976 | 0.000 | 0.000 | -0.096 | 0.488 | 0.060 | 100000.0 0.01 |
| M0689 | 256 | exp14 | 1024bit-ECFP4 | SVM | 0.623 | 0.667 | 0.988 | 0.038 | 0.071 | 0.087 | 0.513 | 0.082 | 5 0.01 |
| M0690 | 256 | exp15 | 1024bit-ECFP4 | SVM | 0.605 | 0.000 | 0.982 | 0.000 | 0.000 | -0.083 | 0.491 | 0.057 | 1000.0 0.005 |
| M0691 | 256 | exp16 | 1024bit-ECFP4 | SVM | 0.616 | 0.000 | 1.000 | 0.000 | 0.000 | 0.000 | 0.500 | 0.000 | 10 0.0005 |
| M0692 | 256 | exp17 | 1024bit-ECFP4 | SVM | 0.616 | 0.000 | 1.000 | 0.000 | 0.000 | 0.000 | 0.500 | 0.019 | 100000.0 0.0001 |
| M0693 | 256 | exp18 | 1024bit-ECFP4 | SVM | 0.620 | 1.000 | 1.000 | 0.009 | 0.019 | 0.076 | 0.504 | 0.042 | 10 0.1 |
| M0694 | 256 | exp19 | 1024bit-ECFP4 | SVM | 0.591 | 0.182 | 0.947 | 0.019 | 0.034 | -0.085 | 0.483 | 0.106 | 10 0.005 |
| M0695 | 256 | exp01 | 1024bit-ECFP4 | GBM | 0.540 | 0.382 | 0.676 | 0.321 | 0.349 | -0.003 | 0.499 | 0.418 | 100 0.9 |
| M0696 | 256 | exp02 | 1024bit-ECFP4 | GBM | 0.565 | 0.403 | 0.747 | 0.274 | 0.326 | 0.023 | 0.510 | 0.381 | 200 0.4 |
| M0697 | 256 | exp03 | 1024bit-ECFP4 | GBM | 0.504 | 0.293 | 0.688 | 0.208 | 0.243 | -0.114 | 0.448 | 0.457 | 100 0.4 |
| M0698 | 256 | exp04 | 1024bit-ECFP4 | GBM | 0.533 | 0.376 | 0.659 | 0.330 | 0.352 | -0.011 | 0.495 | 0.458 | 100 0.7 |
| M0699 | 256 | exp05 | 1024bit-ECFP4 | GBM | 0.511 | 0.333 | 0.659 | 0.274 | 0.301 | -0.071 | 0.467 | 0.415 | 400 0.9 |
| M0700 | 256 | exp06 | 1024bit-ECFP4 | GBM | 0.547 | 0.383 | 0.706 | 0.292 | 0.332 | -0.002 | 0.499 | 0.416 | 100 0.7 |
| M0701 | 256 | exp07 | 1024bit-ECFP4 | GBM | 0.511 | 0.351 | 0.629 | 0.321 | 0.335 | -0.051 | 0.475 | 0.401 | 1000 0.8 |
| M0702 | 256 | exp08 | 1024bit-ECFP4 | GBM | 0.529 | 0.360 | 0.676 | 0.292 | 0.323 | -0.033 | 0.484 | 0.426 | 900 0.2 |
| M0703 | 256 | exp09 | 1024bit-ECFP4 | GBM | 0.569 | 0.437 | 0.659 | 0.425 | 0.431 | 0.084 | 0.542 | 0.452 | 600 0.9 |
| M0704 | 256 | exp10 | 1024bit-ECFP4 | GBM | 0.565 | 0.430 | 0.665 | 0.406 | 0.417 | 0.071 | 0.536 | 0.446 | 900 0.3 |
| M0705 | 256 | exp11 | 1024bit-ECFP4 | GBM | 0.507 | 0.340 | 0.635 | 0.302 | 0.320 | -0.064 | 0.469 | 0.457 | 500 0.8 |
| M0706 | 256 | exp12 | 1024bit-ECFP4 | GBM | 0.591 | 0.460 | 0.724 | 0.377 | 0.415 | 0.106 | 0.550 | 0.415 | 200 0.8 |
| M0707 | 256 | exp13 | 1024bit-ECFP4 | GBM | 0.551 | 0.388 | 0.712 | 0.292 | 0.333 | 0.005 | 0.502 | 0.471 | 1000 0.5 |
| M0708 | 256 | exp14 | 1024bit-ECFP4 | GBM | 0.540 | 0.387 | 0.665 | 0.340 | 0.362 | 0.004 | 0.503 | 0.452 | 300 0.5 |
| M0709 | 256 | exp15 | 1024bit-ECFP4 | GBM | 0.540 | 0.360 | 0.718 | 0.255 | 0.298 | -0.030 | 0.486 | 0.443 | 50 0.8 |
| M0710 | 256 | exp16 | 1024bit-ECFP4 | GBM | 0.572 | 0.446 | 0.635 | 0.472 | 0.459 | 0.106 | 0.553 | 0.415 | 1000 0.5 |
| M0711 | 256 | exp17 | 1024bit-ECFP4 | GBM | 0.529 | 0.364 | 0.671 | 0.302 | 0.330 | -0.029 | 0.487 | 0.419 | 900 0.8 |
| M0712 | 256 | exp18 | 1024bit-ECFP4 | GBM | 0.518 | 0.369 | 0.618 | 0.358 | 0.364 | -0.024 | 0.488 | 0.454 | 700 0.7 |
| M0713 | 256 | exp19 | 1024bit-ECFP4 | GBM | 0.558 | 0.400 | 0.718 | 0.302 | 0.344 | 0.021 | 0.510 | 0.460 | 800 0.1 |
| M0714 | 256 | exp01 | 1024bit-ECFP4 | RF | 0.522 | 0.373 | 0.624 | 0.358 | 0.365 | -0.018 | 0.491 | 0.411 | 10 |
| M0715 | 256 | exp02 | 1024bit-ECFP4 | RF | 0.511 | 0.329 | 0.665 | 0.264 | 0.293 | -0.075 | 0.465 | 0.393 | 10 |
| M0716 | 256 | exp03 | 1024bit-ECFP4 | RF | 0.565 | 0.412 | 0.724 | 0.311 | 0.355 | 0.037 | 0.517 | 0.427 | 10 |
| M0717 | 256 | exp04 | 1024bit-ECFP4 | RF | 0.518 | 0.364 | 0.629 | 0.340 | 0.351 | -0.031 | 0.485 | 0.409 | 10 |
| M0718 | 256 | exp05 | 1024bit-ECFP4 | RF | 0.562 | 0.400 | 0.735 | 0.283 | 0.331 | 0.020 | 0.509 | 0.401 | 10 |
| M0719 | 256 | exp06 | 1024bit-ECFP4 | RF | 0.562 | 0.414 | 0.700 | 0.340 | 0.373 | 0.041 | 0.520 | 0.385 | 10 |
| M0720 | 256 | exp07 | 1024bit-ECFP4 | RF | 0.536 | 0.369 | 0.688 | 0.292 | 0.326 | -0.020 | 0.490 | 0.326 | 10 |
| M0721 | 256 | exp08 | 1024bit-ECFP4 | RF | 0.507 | 0.321 | 0.665 | 0.255 | 0.284 | -0.085 | 0.460 | 0.404 | 10 |
| M0722 | 256 | exp09 | 1024bit-ECFP4 | RF | 0.601 | 0.479 | 0.706 | 0.434 | 0.455 | 0.143 | 0.570 | 0.421 | 10 |
| M0723 | 256 | exp10 | 1024bit-ECFP4 | RF | 0.554 | 0.412 | 0.665 | 0.377 | 0.394 | 0.043 | 0.521 | 0.439 | 10 |
| M0724 | 256 | exp11 | 1024bit-ECFP4 | RF | 0.547 | 0.396 | 0.676 | 0.340 | 0.365 | 0.017 | 0.508 | 0.431 | 10 |
| M0725 | 256 | exp12 | 1024bit-ECFP4 | RF | 0.601 | 0.474 | 0.759 | 0.349 | 0.402 | 0.117 | 0.554 | 0.389 | 10 |
| M0726 | 256 | exp13 | 1024bit-ECFP4 | RF | 0.565 | 0.419 | 0.706 | 0.340 | 0.375 | 0.048 | 0.523 | 0.436 | 10 |
| M0727 | 256 | exp14 | 1024bit-ECFP4 | RF | 0.540 | 0.387 | 0.665 | 0.340 | 0.362 | 0.004 | 0.503 | 0.428 | 10 |
| M0728 | 256 | exp15 | 1024bit-ECFP4 | RF | 0.551 | 0.406 | 0.665 | 0.368 | 0.386 | 0.033 | 0.516 | 0.433 | 10 |
| M0729 | 256 | exp16 | 1024bit-ECFP4 | RF | 0.554 | 0.407 | 0.682 | 0.349 | 0.376 | 0.032 | 0.516 | 0.364 | 10 |
| M0730 | 256 | exp17 | 1024bit-ECFP4 | RF | 0.511 | 0.329 | 0.665 | 0.264 | 0.293 | -0.075 | 0.465 | 0.337 | 10 |
| M0731 | 256 | exp18 | 1024bit-ECFP4 | RF | 0.507 | 0.333 | 0.647 | 0.283 | 0.306 | -0.073 | 0.465 | 0.386 | 10 |
| M0732 | 256 | exp19 | 1024bit-ECFP4 | RF | 0.572 | 0.425 | 0.729 | 0.321 | 0.366 | 0.054 | 0.525 | 0.403 | 10 |
| M0733 | 256 | exp01 | 1024bit-ECFP4 | DNN2 | 0.554 | 0.419 | 0.641 | 0.415 | 0.417 | 0.056 | 0.528 | 0.429 | 500 80 0.3 |
| M0734 | 256 | exp02 | 1024bit-ECFP4 | DNN2 | 0.504 | 0.337 | 0.629 | 0.302 | 0.318 | -0.070 | 0.466 | 0.378 | 400 160 0.5 |
| M0735 | 256 | exp03 | 1024bit-ECFP4 | DNN2 | 0.562 | 0.431 | 0.635 | 0.443 | 0.437 | 0.078 | 0.539 | 0.448 | 100 80 0.3 |
| M0736 | 256 | exp01 | 1024bit-ECFP4 | DNN3 | 0.569 | 0.438 | 0.653 | 0.434 | 0.436 | 0.087 | 0.543 | 0.429 | 400 160 0.1 |
| M0737 | 256 | exp02 | 1024bit-ECFP4 | DNN3 | 0.543 | 0.386 | 0.682 | 0.321 | 0.351 | 0.003 | 0.502 | 0.373 | 600 160 0.5 |
| M0738 | 256 | exp03 | 1024bit-ECFP4 | DNN3 | 0.565 | 0.438 | 0.629 | 0.462 | 0.450 | 0.091 | 0.545 | 0.459 | 500 100 0.2 |
| M0739 | 256 | exp01 | 2048bit-ECFP4 | KNN | 0.562 | 0.407 | 0.718 | 0.311 | 0.353 | 0.031 | 0.514 | 0.392 | 1 uniform |
| M0740 | 256 | exp02 | 2048bit-ECFP4 | KNN | 0.562 | 0.410 | 0.712 | 0.321 | 0.360 | 0.034 | 0.516 | 0.345 | 1 uniform |
| M0741 | 256 | exp03 | 2048bit-ECFP4 | KNN | 0.551 | 0.410 | 0.653 | 0.387 | 0.398 | 0.040 | 0.520 | 0.366 | 1 uniform |
| M0742 | 256 | exp04 | 2048bit-ECFP4 | KNN | 0.536 | 0.380 | 0.665 | 0.330 | 0.354 | -0.005 | 0.498 | 0.418 | 1 uniform |
| M0743 | 256 | exp05 | 2048bit-ECFP4 | KNN | 0.496 | 0.286 | 0.676 | 0.208 | 0.240 | -0.126 | 0.442 | 0.395 | 1 uniform |
| M0744 | 256 | exp06 | 2048bit-ECFP4 | KNN | 0.514 | 0.368 | 0.606 | 0.368 | 0.368 | -0.026 | 0.487 | 0.360 | 1 uniform |
| M0745 | 256 | exp07 | 2048bit-ECFP4 | KNN | 0.529 | 0.395 | 0.594 | 0.425 | 0.409 | 0.018 | 0.509 | 0.323 | 1 uniform |
| M0746 | 256 | exp08 | 2048bit-ECFP4 | KNN | 0.482 | 0.309 | 0.606 | 0.283 | 0.296 | -0.113 | 0.445 | 0.366 | 1 uniform |
| M0747 | 256 | exp09 | 2048bit-ECFP4 | KNN | 0.598 | 0.474 | 0.706 | 0.425 | 0.448 | 0.134 | 0.566 | 0.384 | 1 uniform |
| M0748 | 256 | exp10 | 2048bit-ECFP4 | KNN | 0.565 | 0.403 | 0.747 | 0.274 | 0.326 | 0.023 | 0.510 | 0.390 | 3 distance |
| M0749 | 256 | exp11 | 2048bit-ECFP4 | KNN | 0.529 | 0.367 | 0.665 | 0.311 | 0.337 | -0.025 | 0.488 | 0.405 | 1 uniform |
| M0750 | 256 | exp12 | 2048bit-ECFP4 | KNN | 0.565 | 0.426 | 0.682 | 0.377 | 0.400 | 0.061 | 0.530 | 0.391 | 1 uniform |
| M0751 | 256 | exp13 | 2048bit-ECFP4 | KNN | 0.565 | 0.430 | 0.665 | 0.406 | 0.417 | 0.071 | 0.536 | 0.397 | 1 uniform |
| M0752 | 256 | exp14 | 2048bit-ECFP4 | KNN | 0.558 | 0.431 | 0.612 | 0.472 | 0.450 | 0.082 | 0.542 | 0.410 | 1 uniform |
| M0753 | 256 | exp15 | 2048bit-ECFP4 | KNN | 0.525 | 0.363 | 0.659 | 0.311 | 0.335 | -0.031 | 0.485 | 0.349 | 1 uniform |
| M0754 | 256 | exp16 | 2048bit-ECFP4 | KNN | 0.543 | 0.406 | 0.629 | 0.406 | 0.406 | 0.035 | 0.518 | 0.372 | 1 uniform |
| M0755 | 256 | exp17 | 2048bit-ECFP4 | KNN | 0.507 | 0.353 | 0.612 | 0.340 | 0.346 | -0.049 | 0.476 | 0.335 | 1 uniform |
| M0756 | 256 | exp18 | 2048bit-ECFP4 | KNN | 0.504 | 0.337 | 0.629 | 0.302 | 0.318 | -0.070 | 0.466 | 0.409 | 1 uniform |
| M0757 | 256 | exp19 | 2048bit-ECFP4 | KNN | 0.587 | 0.449 | 0.747 | 0.330 | 0.380 | 0.083 | 0.538 | 0.397 | 1 uniform |
| M0758 | 256 | exp01 | 2048bit-ECFP4 | SVM | 0.623 | 0.625 | 0.982 | 0.047 | 0.088 | 0.086 | 0.514 | 0.133 | 100000.0 0.1 |
| M0759 | 256 | exp02 | 2048bit-ECFP4 | SVM | 0.605 | 0.200 | 0.976 | 0.009 | 0.018 | -0.051 | 0.492 | 0.053 | 5000.0 0.0001 |
| M0760 | 256 | exp03 | 2048bit-ECFP4 | SVM | 0.609 | 0.250 | 0.982 | 0.009 | 0.018 | -0.033 | 0.495 | 0.110 | 1000.0 0.0005 |
| M0761 | 256 | exp04 | 2048bit-ECFP4 | SVM | 0.623 | 0.750 | 0.994 | 0.028 | 0.055 | 0.091 | 0.511 | 0.050 | 50000.0 0.1 |
| M0762 | 256 | exp05 | 2048bit-ECFP4 | SVM | 0.605 | 0.200 | 0.976 | 0.009 | 0.018 | -0.051 | 0.492 | 0.119 | 100000.0 0.005 |
| M0763 | 256 | exp06 | 2048bit-ECFP4 | SVM | 0.612 | 0.462 | 0.959 | 0.057 | 0.101 | 0.035 | 0.508 | 0.147 | 5000.0 0.0005 |
| M0764 | 256 | exp07 | 2048bit-ECFP4 | SVM | 0.616 | 0.000 | 1.000 | 0.000 | 0.000 | 0.000 | 0.500 | 0.018 | 1000.0 0.001 |
| M0765 | 256 | exp08 | 2048bit-ECFP4 | SVM | 0.587 | 0.318 | 0.912 | 0.066 | 0.109 | -0.040 | 0.489 | 0.170 | 100 0.005 |
| M0766 | 256 | exp09 | 2048bit-ECFP4 | SVM | 0.623 | 0.750 | 0.994 | 0.028 | 0.055 | 0.091 | 0.511 | 0.108 | 100 0.01 |
| M0767 | 256 | exp10 | 2048bit-ECFP4 | SVM | 0.605 | 0.435 | 0.924 | 0.094 | 0.155 | 0.031 | 0.509 | 0.225 | 10 0.01 |
| M0768 | 256 | exp11 | 2048bit-ECFP4 | SVM | 0.605 | 0.200 | 0.976 | 0.009 | 0.018 | -0.051 | 0.492 | 0.097 | 1000.0 0.0005 |
| M0769 | 256 | exp12 | 2048bit-ECFP4 | SVM | 0.605 | 0.333 | 0.965 | 0.028 | 0.052 | -0.019 | 0.496 | 0.079 | 500 0.005 |
| M0770 | 256 | exp13 | 2048bit-ECFP4 | SVM | 0.594 | 0.393 | 0.900 | 0.104 | 0.164 | 0.006 | 0.502 | 0.261 | 500 0.0005 |
| M0771 | 256 | exp14 | 2048bit-ECFP4 | SVM | 0.612 | 0.400 | 0.982 | 0.019 | 0.036 | 0.004 | 0.500 | 0.126 | 5000.0 5e-05 |
| M0772 | 256 | exp15 | 2048bit-ECFP4 | SVM | 0.609 | 0.000 | 0.988 | 0.000 | 0.000 | -0.067 | 0.494 | 0.045 | 50000.0 0.05 |
| M0773 | 256 | exp16 | 2048bit-ECFP4 | SVM | 0.616 | 0.000 | 1.000 | 0.000 | 0.000 | 0.000 | 0.500 | 0.033 | 100000.0 1e-05 |
| M0774 | 256 | exp17 | 2048bit-ECFP4 | SVM | 0.612 | 0.333 | 0.988 | 0.009 | 0.018 | -0.011 | 0.498 | 0.066 | 1000.0 0.001 |
| M0775 | 256 | exp18 | 2048bit-ECFP4 | SVM | 0.616 | 0.500 | 0.988 | 0.019 | 0.036 | 0.029 | 0.503 | 0.103 | 1000.0 0.001 |
| M0776 | 256 | exp19 | 2048bit-ECFP4 | SVM | 0.616 | 0.500 | 0.947 | 0.085 | 0.145 | 0.063 | 0.516 | 0.165 | 5 0.01 |
| M0777 | 256 | exp01 | 2048bit-ECFP4 | GBM | 0.533 | 0.351 | 0.706 | 0.255 | 0.295 | -0.043 | 0.480 | 0.436 | 50 0.7 |
| M0778 | 256 | exp02 | 2048bit-ECFP4 | GBM | 0.529 | 0.372 | 0.653 | 0.330 | 0.350 | -0.017 | 0.492 | 0.426 | 300 0.8 |
| M0779 | 256 | exp03 | 2048bit-ECFP4 | GBM | 0.533 | 0.388 | 0.629 | 0.377 | 0.383 | 0.007 | 0.503 | 0.446 | 400 0.8 |
| M0780 | 256 | exp04 | 2048bit-ECFP4 | GBM | 0.511 | 0.333 | 0.659 | 0.274 | 0.301 | -0.071 | 0.467 | 0.453 | 900 0.9 |
| M0781 | 256 | exp05 | 2048bit-ECFP4 | GBM | 0.518 | 0.366 | 0.624 | 0.349 | 0.357 | -0.028 | 0.486 | 0.441 | 1000 0.7 |
| M0782 | 256 | exp06 | 2048bit-ECFP4 | GBM | 0.576 | 0.442 | 0.688 | 0.396 | 0.418 | 0.086 | 0.542 | 0.433 | 900 0.4 |
| M0783 | 256 | exp07 | 2048bit-ECFP4 | GBM | 0.562 | 0.416 | 0.694 | 0.349 | 0.379 | 0.045 | 0.521 | 0.408 | 500 0.9 |
| M0784 | 256 | exp08 | 2048bit-ECFP4 | GBM | 0.507 | 0.321 | 0.665 | 0.255 | 0.284 | -0.085 | 0.460 | 0.436 | 200 0.5 |
| M0785 | 256 | exp09 | 2048bit-ECFP4 | GBM | 0.594 | 0.467 | 0.712 | 0.406 | 0.434 | 0.121 | 0.559 | 0.455 | 600 0.2 |
| M0786 | 256 | exp10 | 2048bit-ECFP4 | GBM | 0.558 | 0.417 | 0.671 | 0.377 | 0.396 | 0.049 | 0.524 | 0.453 | 600 0.4 |
| M0787 | 256 | exp11 | 2048bit-ECFP4 | GBM | 0.533 | 0.361 | 0.688 | 0.283 | 0.317 | -0.030 | 0.485 | 0.469 | 1000 0.9 |
| M0788 | 256 | exp12 | 2048bit-ECFP4 | GBM | 0.536 | 0.392 | 0.635 | 0.377 | 0.385 | 0.013 | 0.506 | 0.457 | 300 0.6 |
| M0789 | 256 | exp13 | 2048bit-ECFP4 | GBM | 0.554 | 0.400 | 0.700 | 0.321 | 0.356 | 0.022 | 0.510 | 0.525 | 300 0.6 |
| M0790 | 256 | exp14 | 2048bit-ECFP4 | GBM | 0.540 | 0.398 | 0.635 | 0.387 | 0.392 | 0.022 | 0.511 | 0.479 | 700 0.9 |
| M0791 | 256 | exp15 | 2048bit-ECFP4 | GBM | 0.547 | 0.402 | 0.659 | 0.368 | 0.384 | 0.027 | 0.514 | 0.450 | 900 0.7 |
| M0792 | 256 | exp16 | 2048bit-ECFP4 | GBM | 0.591 | 0.468 | 0.653 | 0.491 | 0.479 | 0.142 | 0.572 | 0.434 | 900 0.9 |
| M0793 | 256 | exp17 | 2048bit-ECFP4 | GBM | 0.518 | 0.320 | 0.700 | 0.226 | 0.265 | -0.080 | 0.463 | 0.449 | 400 0.2 |
| M0794 | 256 | exp18 | 2048bit-ECFP4 | GBM | 0.529 | 0.382 | 0.629 | 0.368 | 0.375 | -0.003 | 0.498 | 0.484 | 200 0.8 |
| M0795 | 256 | exp19 | 2048bit-ECFP4 | GBM | 0.580 | 0.440 | 0.724 | 0.349 | 0.389 | 0.077 | 0.536 | 0.466 | 700 0.6 |
| M0796 | 256 | exp01 | 2048bit-ECFP4 | RF | 0.580 | 0.414 | 0.800 | 0.226 | 0.293 | 0.032 | 0.513 | 0.362 | 800 |
| M0797 | 256 | exp02 | 2048bit-ECFP4 | RF | 0.529 | 0.354 | 0.688 | 0.274 | 0.309 | -0.041 | 0.481 | 0.375 | 10 |
| M0798 | 256 | exp03 | 2048bit-ECFP4 | RF | 0.554 | 0.395 | 0.712 | 0.302 | 0.342 | 0.015 | 0.507 | 0.402 | 10 |
| M0799 | 256 | exp04 | 2048bit-ECFP4 | RF | 0.533 | 0.371 | 0.671 | 0.311 | 0.338 | -0.019 | 0.491 | 0.413 | 10 |
| M0800 | 256 | exp05 | 2048bit-ECFP4 | RF | 0.529 | 0.350 | 0.694 | 0.264 | 0.301 | -0.045 | 0.479 | 0.398 | 10 |
| M0801 | 256 | exp06 | 2048bit-ECFP4 | RF | 0.583 | 0.454 | 0.688 | 0.415 | 0.433 | 0.105 | 0.551 | 0.375 | 10 |
| M0802 | 256 | exp07 | 2048bit-ECFP4 | RF | 0.580 | 0.442 | 0.718 | 0.358 | 0.396 | 0.080 | 0.538 | 0.365 | 10 |
| M0803 | 256 | exp08 | 2048bit-ECFP4 | RF | 0.522 | 0.349 | 0.671 | 0.283 | 0.312 | -0.049 | 0.477 | 0.414 | 10 |
| M0804 | 256 | exp09 | 2048bit-ECFP4 | RF | 0.569 | 0.430 | 0.688 | 0.377 | 0.402 | 0.067 | 0.532 | 0.431 | 10 |
| M0805 | 256 | exp10 | 2048bit-ECFP4 | RF | 0.591 | 0.457 | 0.741 | 0.349 | 0.396 | 0.096 | 0.545 | 0.434 | 10 |
| M0806 | 256 | exp11 | 2048bit-ECFP4 | RF | 0.551 | 0.400 | 0.682 | 0.340 | 0.367 | 0.023 | 0.511 | 0.436 | 10 |
| M0807 | 256 | exp12 | 2048bit-ECFP4 | RF | 0.569 | 0.425 | 0.706 | 0.349 | 0.383 | 0.058 | 0.527 | 0.413 | 10 |
| M0808 | 256 | exp13 | 2048bit-ECFP4 | RF | 0.540 | 0.367 | 0.706 | 0.274 | 0.314 | -0.022 | 0.490 | 0.465 | 10 |
| M0809 | 256 | exp14 | 2048bit-ECFP4 | RF | 0.551 | 0.408 | 0.659 | 0.377 | 0.392 | 0.037 | 0.518 | 0.427 | 10 |
| M0810 | 256 | exp15 | 2048bit-ECFP4 | RF | 0.562 | 0.421 | 0.676 | 0.377 | 0.398 | 0.055 | 0.526 | 0.428 | 10 |
| M0811 | 256 | exp16 | 2048bit-ECFP4 | RF | 0.601 | 0.476 | 0.747 | 0.368 | 0.415 | 0.122 | 0.557 | 0.382 | 10 |
| M0812 | 256 | exp17 | 2048bit-ECFP4 | RF | 0.514 | 0.325 | 0.682 | 0.245 | 0.280 | -0.078 | 0.464 | 0.389 | 10 |
| M0813 | 256 | exp18 | 2048bit-ECFP4 | RF | 0.504 | 0.337 | 0.629 | 0.302 | 0.318 | -0.070 | 0.466 | 0.420 | 10 |
| M0814 | 256 | exp19 | 2048bit-ECFP4 | RF | 0.558 | 0.402 | 0.712 | 0.311 | 0.351 | 0.025 | 0.511 | 0.401 | 10 |
| M0815 | 256 | exp01 | 2048bit-ECFP4 | DNN2 | 0.536 | 0.369 | 0.688 | 0.292 | 0.326 | -0.020 | 0.490 | 0.423 | 400 140 0.1 |
| M0816 | 256 | exp02 | 2048bit-ECFP4 | DNN2 | 0.514 | 0.357 | 0.629 | 0.330 | 0.343 | -0.041 | 0.480 | 0.414 | 400 140 0.1 |
| M0817 | 256 | exp03 | 2048bit-ECFP4 | DNN2 | 0.525 | 0.387 | 0.600 | 0.406 | 0.396 | 0.006 | 0.503 | 0.458 | 500 140 0.1 |
| M0818 | 256 | exp01 | 2048bit-ECFP4 | DNN3 | 0.569 | 0.416 | 0.735 | 0.302 | 0.350 | 0.040 | 0.518 | 0.423 | 400 140 0.3 |
| M0819 | 256 | exp02 | 2048bit-ECFP4 | DNN3 | 0.525 | 0.371 | 0.641 | 0.340 | 0.355 | -0.020 | 0.491 | 0.407 | 300 160 0.2 |
| M0820 | 256 | exp03 | 2048bit-ECFP4 | DNN3 | 0.562 | 0.419 | 0.682 | 0.368 | 0.392 | 0.052 | 0.525 | 0.457 | 400 100 0.2 |
| M0821 | 256 | exp01 | 1024bit-ECFP6 | KNN | 0.558 | 0.397 | 0.724 | 0.292 | 0.337 | 0.017 | 0.508 | 0.398 | 1 uniform |
| M0822 | 256 | exp02 | 1024bit-ECFP6 | KNN | 0.547 | 0.393 | 0.682 | 0.330 | 0.359 | 0.013 | 0.506 | 0.367 | 1 uniform |
| M0823 | 256 | exp03 | 1024bit-ECFP6 | KNN | 0.569 | 0.418 | 0.729 | 0.311 | 0.357 | 0.044 | 0.520 | 0.381 | 3 distance |
| M0824 | 256 | exp04 | 1024bit-ECFP6 | KNN | 0.518 | 0.355 | 0.647 | 0.311 | 0.332 | -0.043 | 0.479 | 0.416 | 1 uniform |
| M0825 | 256 | exp05 | 1024bit-ECFP6 | KNN | 0.533 | 0.379 | 0.653 | 0.340 | 0.358 | -0.008 | 0.497 | 0.392 | 1 uniform |
| M0826 | 256 | exp06 | 1024bit-ECFP6 | KNN | 0.489 | 0.339 | 0.576 | 0.349 | 0.344 | -0.074 | 0.462 | 0.347 | 1 uniform |
| M0827 | 256 | exp07 | 1024bit-ECFP6 | KNN | 0.514 | 0.375 | 0.588 | 0.396 | 0.385 | -0.015 | 0.492 | 0.345 | 1 uniform |
| M0828 | 256 | exp08 | 1024bit-ECFP6 | KNN | 0.540 | 0.389 | 0.659 | 0.349 | 0.368 | 0.008 | 0.504 | 0.359 | 1 uniform |
| M0829 | 256 | exp09 | 1024bit-ECFP6 | KNN | 0.587 | 0.457 | 0.700 | 0.406 | 0.430 | 0.108 | 0.553 | 0.398 | 1 uniform |
| M0830 | 256 | exp10 | 1024bit-ECFP6 | KNN | 0.478 | 0.302 | 0.606 | 0.274 | 0.287 | -0.123 | 0.440 | 0.410 | 1 uniform |
| M0831 | 256 | exp11 | 1024bit-ECFP6 | KNN | 0.500 | 0.337 | 0.618 | 0.311 | 0.324 | -0.072 | 0.465 | 0.412 | 1 uniform |
| M0832 | 256 | exp12 | 1024bit-ECFP6 | KNN | 0.543 | 0.394 | 0.665 | 0.349 | 0.370 | 0.014 | 0.507 | 0.383 | 1 uniform |
| M0833 | 256 | exp13 | 1024bit-ECFP6 | KNN | 0.583 | 0.449 | 0.712 | 0.377 | 0.410 | 0.093 | 0.544 | 0.404 | 1 uniform |
| M0834 | 256 | exp14 | 1024bit-ECFP6 | KNN | 0.551 | 0.406 | 0.665 | 0.368 | 0.386 | 0.033 | 0.516 | 0.386 | 1 uniform |
| M0835 | 256 | exp15 | 1024bit-ECFP6 | KNN | 0.507 | 0.347 | 0.624 | 0.321 | 0.333 | -0.057 | 0.473 | 0.343 | 1 uniform |
| M0836 | 256 | exp16 | 1024bit-ECFP6 | KNN | 0.551 | 0.415 | 0.635 | 0.415 | 0.415 | 0.050 | 0.525 | 0.335 | 1 uniform |
| M0837 | 256 | exp17 | 1024bit-ECFP6 | KNN | 0.496 | 0.330 | 0.618 | 0.302 | 0.315 | -0.082 | 0.460 | 0.380 | 1 uniform |
| M0838 | 256 | exp18 | 1024bit-ECFP6 | KNN | 0.522 | 0.370 | 0.629 | 0.349 | 0.359 | -0.022 | 0.489 | 0.405 | 1 uniform |
| M0839 | 256 | exp19 | 1024bit-ECFP6 | KNN | 0.569 | 0.424 | 0.712 | 0.340 | 0.377 | 0.054 | 0.526 | 0.389 | 1 uniform |
| M0840 | 256 | exp01 | 1024bit-ECFP6 | SVM | 0.620 | 0.556 | 0.976 | 0.047 | 0.087 | 0.065 | 0.511 | 0.139 | 50 0.05 |
| M0841 | 256 | exp02 | 1024bit-ECFP6 | SVM | 0.605 | 0.000 | 0.982 | 0.000 | 0.000 | -0.083 | 0.491 | 0.048 | 5000.0 1e-05 |
| M0842 | 256 | exp03 | 1024bit-ECFP6 | SVM | 0.612 | 0.444 | 0.971 | 0.038 | 0.070 | 0.023 | 0.504 | 0.142 | 500 0.0001 |
| M0843 | 256 | exp04 | 1024bit-ECFP6 | SVM | 0.572 | 0.167 | 0.912 | 0.028 | 0.048 | -0.118 | 0.470 | 0.137 | 1000.0 0.0001 |
| M0844 | 256 | exp05 | 1024bit-ECFP6 | SVM | 0.609 | 0.333 | 0.976 | 0.019 | 0.036 | -0.016 | 0.497 | 0.101 | 10000.0 1e-05 |
| M0845 | 256 | exp06 | 1024bit-ECFP6 | SVM | 0.616 | 0.000 | 1.000 | 0.000 | 0.000 | 0.000 | 0.500 | 0.000 | 1 5e-05 |
| M0846 | 256 | exp07 | 1024bit-ECFP6 | SVM | 0.598 | 0.273 | 0.953 | 0.028 | 0.051 | -0.047 | 0.490 | 0.143 | 10000.0 1e-05 |
| M0847 | 256 | exp08 | 1024bit-ECFP6 | SVM | 0.601 | 0.300 | 0.959 | 0.028 | 0.052 | -0.034 | 0.493 | 0.087 | 10000.0 1e-05 |
| M0848 | 256 | exp09 | 1024bit-ECFP6 | SVM | 0.623 | 0.583 | 0.971 | 0.066 | 0.119 | 0.087 | 0.518 | 0.122 | 5000.0 0.05 |
| M0849 | 256 | exp10 | 1024bit-ECFP6 | SVM | 0.612 | 0.429 | 0.976 | 0.028 | 0.053 | 0.015 | 0.502 | 0.186 | 10000.0 0.005 |
| M0850 | 256 | exp11 | 1024bit-ECFP6 | SVM | 0.598 | 0.273 | 0.953 | 0.028 | 0.051 | -0.047 | 0.490 | 0.125 | 500 0.0005 |
| M0851 | 256 | exp12 | 1024bit-ECFP6 | SVM | 0.620 | 0.600 | 0.988 | 0.028 | 0.054 | 0.060 | 0.508 | 0.063 | 1000.0 0.0001 |
| M0852 | 256 | exp13 | 1024bit-ECFP6 | SVM | 0.612 | 0.333 | 0.988 | 0.009 | 0.018 | -0.011 | 0.498 | 0.124 | 5000.0 0.005 |
| M0853 | 256 | exp14 | 1024bit-ECFP6 | SVM | 0.620 | 0.526 | 0.947 | 0.094 | 0.160 | 0.080 | 0.520 | 0.170 | 10 0.01 |
| M0854 | 256 | exp15 | 1024bit-ECFP6 | SVM | 0.612 | 0.400 | 0.982 | 0.019 | 0.036 | 0.004 | 0.500 | 0.064 | 10000.0 0.005 |
| M0855 | 256 | exp16 | 1024bit-ECFP6 | SVM | 0.616 | 0.000 | 1.000 | 0.000 | 0.000 | 0.000 | 0.500 | 0.000 | 100 0.0001 |
| M0856 | 256 | exp17 | 1024bit-ECFP6 | SVM | 0.572 | 0.000 | 0.929 | 0.000 | 0.000 | -0.168 | 0.465 | 0.109 | 50000.0 0.005 |
| M0857 | 256 | exp18 | 1024bit-ECFP6 | SVM | 0.623 | 0.750 | 0.994 | 0.028 | 0.055 | 0.091 | 0.511 | 0.081 | 100 0.1 |
| M0858 | 256 | exp19 | 1024bit-ECFP6 | SVM | 0.594 | 0.312 | 0.935 | 0.047 | 0.082 | -0.036 | 0.491 | 0.128 | 10 0.005 |
| M0859 | 256 | exp01 | 1024bit-ECFP6 | GBM | 0.543 | 0.375 | 0.706 | 0.283 | 0.323 | -0.012 | 0.494 | 0.428 | 300 0.8 |
| M0860 | 256 | exp02 | 1024bit-ECFP6 | GBM | 0.525 | 0.363 | 0.659 | 0.311 | 0.335 | -0.031 | 0.485 | 0.436 | 500 0.5 |
| M0861 | 256 | exp03 | 1024bit-ECFP6 | GBM | 0.551 | 0.398 | 0.688 | 0.330 | 0.361 | 0.019 | 0.509 | 0.466 | 900 0.6 |
| M0862 | 256 | exp04 | 1024bit-ECFP6 | GBM | 0.514 | 0.370 | 0.600 | 0.377 | 0.374 | -0.023 | 0.488 | 0.460 | 1000 0.5 |
| M0863 | 256 | exp05 | 1024bit-ECFP6 | GBM | 0.478 | 0.317 | 0.582 | 0.311 | 0.314 | -0.107 | 0.447 | 0.423 | 1000 0.9 |
| M0864 | 256 | exp06 | 1024bit-ECFP6 | GBM | 0.540 | 0.367 | 0.706 | 0.274 | 0.314 | -0.022 | 0.490 | 0.393 | 700 0.2 |
| M0865 | 256 | exp07 | 1024bit-ECFP6 | GBM | 0.598 | 0.472 | 0.724 | 0.396 | 0.431 | 0.125 | 0.560 | 0.422 | 1000 0.8 |
| M0866 | 256 | exp08 | 1024bit-ECFP6 | GBM | 0.478 | 0.302 | 0.606 | 0.274 | 0.287 | -0.123 | 0.440 | 0.426 | 500 0.7 |
| M0867 | 256 | exp09 | 1024bit-ECFP6 | GBM | 0.572 | 0.440 | 0.671 | 0.415 | 0.427 | 0.087 | 0.543 | 0.458 | 600 0.5 |
| M0868 | 256 | exp10 | 1024bit-ECFP6 | GBM | 0.554 | 0.400 | 0.700 | 0.321 | 0.356 | 0.022 | 0.510 | 0.472 | 100 0.7 |
| M0869 | 256 | exp11 | 1024bit-ECFP6 | GBM | 0.536 | 0.378 | 0.671 | 0.321 | 0.347 | -0.009 | 0.496 | 0.456 | 800 0.8 |
| M0870 | 256 | exp12 | 1024bit-ECFP6 | GBM | 0.587 | 0.456 | 0.712 | 0.387 | 0.418 | 0.102 | 0.549 | 0.463 | 900 0.6 |
| M0871 | 256 | exp13 | 1024bit-ECFP6 | GBM | 0.569 | 0.427 | 0.700 | 0.358 | 0.390 | 0.061 | 0.529 | 0.478 | 200 0.7 |
| M0872 | 256 | exp14 | 1024bit-ECFP6 | GBM | 0.529 | 0.372 | 0.653 | 0.330 | 0.350 | -0.017 | 0.492 | 0.466 | 300 0.7 |
| M0873 | 256 | exp15 | 1024bit-ECFP6 | GBM | 0.514 | 0.360 | 0.624 | 0.340 | 0.350 | -0.037 | 0.482 | 0.418 | 200 0.9 |
| M0874 | 256 | exp16 | 1024bit-ECFP6 | GBM | 0.536 | 0.372 | 0.682 | 0.302 | 0.333 | -0.017 | 0.492 | 0.415 | 900 0.5 |
| M0875 | 256 | exp17 | 1024bit-ECFP6 | GBM | 0.478 | 0.302 | 0.606 | 0.274 | 0.287 | -0.123 | 0.440 | 0.434 | 600 0.4 |
| M0876 | 256 | exp18 | 1024bit-ECFP6 | GBM | 0.529 | 0.370 | 0.659 | 0.321 | 0.343 | -0.021 | 0.490 | 0.488 | 200 0.4 |
| M0877 | 256 | exp19 | 1024bit-ECFP6 | GBM | 0.558 | 0.400 | 0.718 | 0.302 | 0.344 | 0.021 | 0.510 | 0.432 | 500 0.9 |
| M0878 | 256 | exp01 | 1024bit-ECFP6 | RF | 0.529 | 0.357 | 0.682 | 0.283 | 0.316 | -0.037 | 0.483 | 0.397 | 10 |
| M0879 | 256 | exp02 | 1024bit-ECFP6 | RF | 0.489 | 0.312 | 0.624 | 0.274 | 0.291 | -0.106 | 0.449 | 0.416 | 10 |
| M0880 | 256 | exp03 | 1024bit-ECFP6 | RF | 0.562 | 0.416 | 0.694 | 0.349 | 0.379 | 0.045 | 0.521 | 0.423 | 10 |
| M0881 | 256 | exp04 | 1024bit-ECFP6 | RF | 0.460 | 0.310 | 0.541 | 0.330 | 0.320 | -0.127 | 0.435 | 0.467 | 10 |
| M0882 | 256 | exp05 | 1024bit-ECFP6 | RF | 0.525 | 0.363 | 0.659 | 0.311 | 0.335 | -0.031 | 0.485 | 0.399 | 10 |
| M0883 | 256 | exp06 | 1024bit-ECFP6 | RF | 0.540 | 0.392 | 0.653 | 0.358 | 0.374 | 0.012 | 0.506 | 0.363 | 10 |
| M0884 | 256 | exp07 | 1024bit-ECFP6 | RF | 0.562 | 0.424 | 0.665 | 0.396 | 0.410 | 0.062 | 0.530 | 0.359 | 10 |
| M0885 | 256 | exp08 | 1024bit-ECFP6 | RF | 0.511 | 0.337 | 0.653 | 0.283 | 0.308 | -0.067 | 0.468 | 0.376 | 10 |
| M0886 | 256 | exp09 | 1024bit-ECFP6 | RF | 0.547 | 0.398 | 0.671 | 0.349 | 0.372 | 0.020 | 0.510 | 0.402 | 10 |
| M0887 | 256 | exp10 | 1024bit-ECFP6 | RF | 0.504 | 0.340 | 0.624 | 0.311 | 0.325 | -0.066 | 0.468 | 0.434 | 10 |
| M0888 | 256 | exp11 | 1024bit-ECFP6 | RF | 0.514 | 0.354 | 0.635 | 0.321 | 0.337 | -0.045 | 0.478 | 0.414 | 10 |
| M0889 | 256 | exp12 | 1024bit-ECFP6 | RF | 0.547 | 0.400 | 0.665 | 0.358 | 0.378 | 0.024 | 0.512 | 0.430 | 10 |
| M0890 | 256 | exp13 | 1024bit-ECFP6 | RF | 0.562 | 0.410 | 0.712 | 0.321 | 0.360 | 0.034 | 0.516 | 0.435 | 10 |
| M0891 | 256 | exp14 | 1024bit-ECFP6 | RF | 0.580 | 0.446 | 0.700 | 0.387 | 0.414 | 0.090 | 0.543 | 0.417 | 10 |
| M0892 | 256 | exp15 | 1024bit-ECFP6 | RF | 0.529 | 0.382 | 0.629 | 0.368 | 0.375 | -0.003 | 0.498 | 0.422 | 10 |
| M0893 | 256 | exp16 | 1024bit-ECFP6 | RF | 0.580 | 0.439 | 0.729 | 0.340 | 0.383 | 0.073 | 0.534 | 0.360 | 10 |
| M0894 | 256 | exp17 | 1024bit-ECFP6 | RF | 0.478 | 0.293 | 0.618 | 0.255 | 0.273 | -0.132 | 0.436 | 0.431 | 10 |
| M0895 | 256 | exp18 | 1024bit-ECFP6 | RF | 0.507 | 0.337 | 0.641 | 0.292 | 0.313 | -0.068 | 0.467 | 0.452 | 10 |
| M0896 | 256 | exp19 | 1024bit-ECFP6 | RF | 0.591 | 0.451 | 0.771 | 0.302 | 0.362 | 0.081 | 0.536 | 0.390 | 10 |
| M0897 | 256 | exp01 | 1024bit-ECFP6 | DNN2 | 0.558 | 0.415 | 0.676 | 0.368 | 0.390 | 0.046 | 0.522 | 0.436 | 300 140 0.5 |
| M0898 | 256 | exp02 | 1024bit-ECFP6 | DNN2 | 0.504 | 0.343 | 0.618 | 0.321 | 0.332 | -0.062 | 0.470 | 0.443 | 500 60 0.3 |
| M0899 | 256 | exp03 | 1024bit-ECFP6 | DNN2 | 0.511 | 0.354 | 0.624 | 0.330 | 0.341 | -0.047 | 0.477 | 0.460 | 100 160 0.3 |
| M0900 | 256 | exp01 | 1024bit-ECFP6 | DNN3 | 0.558 | 0.417 | 0.671 | 0.377 | 0.396 | 0.049 | 0.524 | 0.427 | 200 80 0.3 |
| M0901 | 256 | exp02 | 1024bit-ECFP6 | DNN3 | 0.518 | 0.369 | 0.618 | 0.358 | 0.364 | -0.024 | 0.488 | 0.434 | 400 120 0.4 |
| M0902 | 256 | exp03 | 1024bit-ECFP6 | DNN3 | 0.551 | 0.398 | 0.688 | 0.330 | 0.361 | 0.019 | 0.509 | 0.467 | 100 160 0.3 |
| M0903 | 256 | exp01 | 2048bit-ECFP6 | KNN | 0.576 | 0.429 | 0.741 | 0.311 | 0.361 | 0.057 | 0.526 | 0.379 | 1 uniform |
| M0904 | 256 | exp02 | 2048bit-ECFP6 | KNN | 0.529 | 0.354 | 0.688 | 0.274 | 0.309 | -0.041 | 0.481 | 0.351 | 1 uniform |
| M0905 | 256 | exp03 | 2048bit-ECFP6 | KNN | 0.583 | 0.452 | 0.700 | 0.396 | 0.422 | 0.099 | 0.548 | 0.366 | 1 uniform |
| M0906 | 256 | exp04 | 2048bit-ECFP6 | KNN | 0.529 | 0.372 | 0.653 | 0.330 | 0.350 | -0.017 | 0.492 | 0.397 | 1 uniform |
| M0907 | 256 | exp05 | 2048bit-ECFP6 | KNN | 0.554 | 0.395 | 0.712 | 0.302 | 0.342 | 0.015 | 0.507 | 0.389 | 1 uniform |
| M0908 | 256 | exp06 | 2048bit-ECFP6 | KNN | 0.554 | 0.404 | 0.688 | 0.340 | 0.369 | 0.029 | 0.514 | 0.326 | 1 uniform |
| M0909 | 256 | exp07 | 2048bit-ECFP6 | KNN | 0.529 | 0.372 | 0.653 | 0.330 | 0.350 | -0.017 | 0.492 | 0.334 | 1 uniform |
| M0910 | 256 | exp08 | 2048bit-ECFP6 | KNN | 0.507 | 0.330 | 0.653 | 0.274 | 0.299 | -0.077 | 0.464 | 0.358 | 1 uniform |
| M0911 | 256 | exp09 | 2048bit-ECFP6 | KNN | 0.572 | 0.429 | 0.718 | 0.340 | 0.379 | 0.061 | 0.529 | 0.359 | 1 uniform |
| M0912 | 256 | exp10 | 2048bit-ECFP6 | KNN | 0.543 | 0.386 | 0.682 | 0.321 | 0.351 | 0.003 | 0.502 | 0.406 | 1 uniform |
| M0913 | 256 | exp11 | 2048bit-ECFP6 | KNN | 0.572 | 0.435 | 0.694 | 0.377 | 0.404 | 0.074 | 0.535 | 0.399 | 1 uniform |
| M0914 | 256 | exp12 | 2048bit-ECFP6 | KNN | 0.572 | 0.432 | 0.706 | 0.358 | 0.392 | 0.067 | 0.532 | 0.371 | 1 uniform |
| M0915 | 256 | exp13 | 2048bit-ECFP6 | KNN | 0.565 | 0.419 | 0.706 | 0.340 | 0.375 | 0.048 | 0.523 | 0.396 | 1 uniform |
| M0916 | 256 | exp14 | 2048bit-ECFP6 | KNN | 0.551 | 0.404 | 0.671 | 0.358 | 0.380 | 0.030 | 0.514 | 0.406 | 1 uniform |
| M0917 | 256 | exp15 | 2048bit-ECFP6 | KNN | 0.493 | 0.323 | 0.618 | 0.292 | 0.307 | -0.092 | 0.455 | 0.381 | 1 uniform |
| M0918 | 256 | exp16 | 2048bit-ECFP6 | KNN | 0.562 | 0.410 | 0.712 | 0.321 | 0.360 | 0.034 | 0.516 | 0.338 | 1 uniform |
| M0919 | 256 | exp17 | 2048bit-ECFP6 | KNN | 0.598 | 0.470 | 0.741 | 0.368 | 0.413 | 0.116 | 0.554 | 0.355 | 1 uniform |
| M0920 | 256 | exp18 | 2048bit-ECFP6 | KNN | 0.518 | 0.337 | 0.676 | 0.264 | 0.296 | -0.063 | 0.470 | 0.403 | 1 uniform |
| M0921 | 256 | exp19 | 2048bit-ECFP6 | KNN | 0.551 | 0.388 | 0.712 | 0.292 | 0.333 | 0.005 | 0.502 | 0.391 | 1 uniform |
| M0922 | 256 | exp01 | 2048bit-ECFP6 | SVM | 0.601 | 0.433 | 0.900 | 0.123 | 0.191 | 0.035 | 0.512 | 0.290 | 1 0.1 |
| M0923 | 256 | exp02 | 2048bit-ECFP6 | SVM | 0.583 | 0.263 | 0.918 | 0.047 | 0.080 | -0.068 | 0.483 | 0.157 | 10 0.01 |
| M0924 | 256 | exp03 | 2048bit-ECFP6 | SVM | 0.598 | 0.308 | 0.947 | 0.038 | 0.067 | -0.035 | 0.492 | 0.182 | 10 0.01 |
| M0925 | 256 | exp04 | 2048bit-ECFP6 | SVM | 0.551 | 0.179 | 0.865 | 0.047 | 0.075 | -0.142 | 0.456 | 0.214 | 5000.0 5e-05 |
| M0926 | 256 | exp05 | 2048bit-ECFP6 | SVM | 0.583 | 0.355 | 0.882 | 0.104 | 0.161 | -0.021 | 0.493 | 0.224 | 10 0.05 |
| M0927 | 256 | exp06 | 2048bit-ECFP6 | SVM | 0.641 | 0.600 | 0.918 | 0.198 | 0.298 | 0.169 | 0.558 | 0.191 | 5 0.01 |
| M0928 | 256 | exp07 | 2048bit-ECFP6 | SVM | 0.609 | 0.455 | 0.929 | 0.094 | 0.156 | 0.043 | 0.512 | 0.195 | 10000.0 5e-05 |
| M0929 | 256 | exp08 | 2048bit-ECFP6 | SVM | 0.576 | 0.296 | 0.888 | 0.075 | 0.120 | -0.059 | 0.481 | 0.263 | 100000.0 1e-05 |
| M0930 | 256 | exp09 | 2048bit-ECFP6 | SVM | 0.667 | 0.733 | 0.953 | 0.208 | 0.324 | 0.251 | 0.581 | 0.260 | 1 0.05 |
| M0931 | 256 | exp10 | 2048bit-ECFP6 | SVM | 0.583 | 0.400 | 0.841 | 0.170 | 0.238 | 0.014 | 0.505 | 0.404 | 50 0.01 |
| M0932 | 256 | exp11 | 2048bit-ECFP6 | SVM | 0.576 | 0.372 | 0.841 | 0.151 | 0.215 | -0.011 | 0.496 | 0.317 | 50 0.01 |
| M0933 | 256 | exp12 | 2048bit-ECFP6 | SVM | 0.634 | 0.571 | 0.912 | 0.189 | 0.284 | 0.147 | 0.550 | 0.263 | 10 0.01 |
| M0934 | 256 | exp13 | 2048bit-ECFP6 | SVM | 0.583 | 0.364 | 0.876 | 0.113 | 0.173 | -0.015 | 0.494 | 0.390 | 100 0.01 |
| M0935 | 256 | exp14 | 2048bit-ECFP6 | SVM | 0.601 | 0.444 | 0.882 | 0.151 | 0.225 | 0.048 | 0.516 | 0.249 | 1 0.05 |
| M0936 | 256 | exp15 | 2048bit-ECFP6 | SVM | 0.612 | 0.483 | 0.912 | 0.132 | 0.207 | 0.070 | 0.522 | 0.260 | 100 0.005 |
| M0937 | 256 | exp16 | 2048bit-ECFP6 | SVM | 0.620 | 1.000 | 1.000 | 0.009 | 0.019 | 0.076 | 0.504 | 0.070 | 50000.0 0.0001 |
| M0938 | 256 | exp17 | 2048bit-ECFP6 | SVM | 0.601 | 0.400 | 0.929 | 0.075 | 0.127 | 0.009 | 0.502 | 0.205 | 1 0.1 |
| M0939 | 256 | exp18 | 2048bit-ECFP6 | SVM | 0.601 | 0.433 | 0.900 | 0.123 | 0.191 | 0.035 | 0.512 | 0.256 | 50 0.05 |
| M0940 | 256 | exp19 | 2048bit-ECFP6 | SVM | 0.612 | 0.488 | 0.876 | 0.189 | 0.272 | 0.089 | 0.532 | 0.324 | 1 0.05 |
| M0941 | 256 | exp01 | 2048bit-ECFP6 | GBM | 0.493 | 0.293 | 0.659 | 0.226 | 0.255 | -0.122 | 0.443 | 0.447 | 50 0.9 |
| M0942 | 256 | exp02 | 2048bit-ECFP6 | GBM | 0.543 | 0.389 | 0.676 | 0.330 | 0.357 | 0.007 | 0.503 | 0.474 | 600 0.9 |
| M0943 | 256 | exp03 | 2048bit-ECFP6 | GBM | 0.547 | 0.391 | 0.688 | 0.321 | 0.352 | 0.009 | 0.504 | 0.499 | 1000 0.9 |
| M0944 | 256 | exp04 | 2048bit-ECFP6 | GBM | 0.525 | 0.363 | 0.659 | 0.311 | 0.335 | -0.031 | 0.485 | 0.478 | 50 0.9 |
| M0945 | 256 | exp05 | 2048bit-ECFP6 | GBM | 0.529 | 0.375 | 0.647 | 0.340 | 0.356 | -0.014 | 0.494 | 0.489 | 300 0.8 |
| M0946 | 256 | exp06 | 2048bit-ECFP6 | GBM | 0.562 | 0.423 | 0.671 | 0.387 | 0.404 | 0.058 | 0.529 | 0.436 | 1000 0.5 |
| M0947 | 256 | exp07 | 2048bit-ECFP6 | GBM | 0.572 | 0.423 | 0.735 | 0.311 | 0.359 | 0.050 | 0.523 | 0.447 | 300 0.7 |
| M0948 | 256 | exp08 | 2048bit-ECFP6 | GBM | 0.486 | 0.316 | 0.606 | 0.292 | 0.304 | -0.103 | 0.449 | 0.480 | 1000 0.9 |
| M0949 | 256 | exp09 | 2048bit-ECFP6 | GBM | 0.540 | 0.398 | 0.635 | 0.387 | 0.392 | 0.022 | 0.511 | 0.465 | 1000 0.7 |
| M0950 | 256 | exp10 | 2048bit-ECFP6 | GBM | 0.522 | 0.370 | 0.629 | 0.349 | 0.359 | -0.022 | 0.489 | 0.504 | 1000 0.8 |
| M0951 | 256 | exp11 | 2048bit-ECFP6 | GBM | 0.529 | 0.380 | 0.635 | 0.358 | 0.369 | -0.006 | 0.496 | 0.475 | 900 0.9 |
| M0952 | 256 | exp12 | 2048bit-ECFP6 | GBM | 0.554 | 0.409 | 0.676 | 0.358 | 0.382 | 0.036 | 0.517 | 0.471 | 100 0.8 |
| M0953 | 256 | exp13 | 2048bit-ECFP6 | GBM | 0.558 | 0.405 | 0.706 | 0.321 | 0.358 | 0.028 | 0.513 | 0.549 | 500 0.7 |
| M0954 | 256 | exp14 | 2048bit-ECFP6 | GBM | 0.540 | 0.392 | 0.653 | 0.358 | 0.374 | 0.012 | 0.506 | 0.471 | 800 0.8 |
| M0955 | 256 | exp15 | 2048bit-ECFP6 | GBM | 0.540 | 0.387 | 0.665 | 0.340 | 0.362 | 0.004 | 0.503 | 0.480 | 500 0.2 |
| M0956 | 256 | exp16 | 2048bit-ECFP6 | GBM | 0.554 | 0.398 | 0.706 | 0.311 | 0.349 | 0.018 | 0.508 | 0.447 | 50 0.8 |
| M0957 | 256 | exp17 | 2048bit-ECFP6 | GBM | 0.536 | 0.359 | 0.706 | 0.264 | 0.304 | -0.032 | 0.485 | 0.470 | 500 0.2 |
| M0958 | 256 | exp18 | 2048bit-ECFP6 | GBM | 0.518 | 0.348 | 0.659 | 0.292 | 0.318 | -0.051 | 0.476 | 0.480 | 1000 0.9 |
| M0959 | 256 | exp19 | 2048bit-ECFP6 | GBM | 0.540 | 0.385 | 0.671 | 0.330 | 0.355 | 0.001 | 0.501 | 0.492 | 400 0.8 |
| M0960 | 256 | exp01 | 2048bit-ECFP6 | RF | 0.529 | 0.346 | 0.700 | 0.255 | 0.293 | -0.049 | 0.477 | 0.422 | 10 |
| M0961 | 256 | exp02 | 2048bit-ECFP6 | RF | 0.547 | 0.398 | 0.671 | 0.349 | 0.372 | 0.020 | 0.510 | 0.396 | 10 |
| M0962 | 256 | exp03 | 2048bit-ECFP6 | RF | 0.558 | 0.400 | 0.718 | 0.302 | 0.344 | 0.021 | 0.510 | 0.448 | 10 |
| M0963 | 256 | exp04 | 2048bit-ECFP6 | RF | 0.482 | 0.313 | 0.600 | 0.292 | 0.302 | -0.109 | 0.446 | 0.456 | 10 |
| M0964 | 256 | exp05 | 2048bit-ECFP6 | RF | 0.536 | 0.366 | 0.694 | 0.283 | 0.319 | -0.024 | 0.488 | 0.412 | 10 |
| M0965 | 256 | exp06 | 2048bit-ECFP6 | RF | 0.554 | 0.412 | 0.665 | 0.377 | 0.394 | 0.043 | 0.521 | 0.388 | 10 |
| M0966 | 256 | exp07 | 2048bit-ECFP6 | RF | 0.540 | 0.373 | 0.694 | 0.292 | 0.328 | -0.014 | 0.493 | 0.384 | 10 |
| M0967 | 256 | exp08 | 2048bit-ECFP6 | RF | 0.504 | 0.333 | 0.635 | 0.292 | 0.312 | -0.074 | 0.464 | 0.448 | 10 |
| M0968 | 256 | exp09 | 2048bit-ECFP6 | RF | 0.580 | 0.447 | 0.694 | 0.396 | 0.420 | 0.093 | 0.545 | 0.432 | 10 |
| M0969 | 256 | exp10 | 2048bit-ECFP6 | RF | 0.529 | 0.367 | 0.665 | 0.311 | 0.337 | -0.025 | 0.488 | 0.463 | 10 |
| M0970 | 256 | exp11 | 2048bit-ECFP6 | RF | 0.591 | 0.460 | 0.724 | 0.377 | 0.415 | 0.106 | 0.550 | 0.452 | 10 |
| M0971 | 256 | exp12 | 2048bit-ECFP6 | RF | 0.594 | 0.464 | 0.735 | 0.368 | 0.411 | 0.109 | 0.551 | 0.423 | 10 |
| M0972 | 256 | exp13 | 2048bit-ECFP6 | RF | 0.569 | 0.427 | 0.700 | 0.358 | 0.390 | 0.061 | 0.529 | 0.471 | 10 |
| M0973 | 256 | exp14 | 2048bit-ECFP6 | RF | 0.543 | 0.402 | 0.641 | 0.387 | 0.394 | 0.028 | 0.514 | 0.419 | 10 |
| M0974 | 256 | exp15 | 2048bit-ECFP6 | RF | 0.525 | 0.356 | 0.671 | 0.292 | 0.321 | -0.039 | 0.482 | 0.436 | 10 |
| M0975 | 256 | exp16 | 2048bit-ECFP6 | RF | 0.569 | 0.420 | 0.724 | 0.321 | 0.364 | 0.047 | 0.522 | 0.388 | 10 |
| M0976 | 256 | exp17 | 2048bit-ECFP6 | RF | 0.569 | 0.413 | 0.741 | 0.292 | 0.343 | 0.037 | 0.516 | 0.405 | 10 |
| M0977 | 256 | exp18 | 2048bit-ECFP6 | RF | 0.522 | 0.329 | 0.700 | 0.236 | 0.275 | -0.070 | 0.468 | 0.415 | 10 |
| M0978 | 256 | exp19 | 2048bit-ECFP6 | RF | 0.587 | 0.444 | 0.765 | 0.302 | 0.360 | 0.074 | 0.533 | 0.419 | 10 |
| M0979 | 256 | exp01 | 2048bit-ECFP6 | DNN2 | 0.536 | 0.378 | 0.671 | 0.321 | 0.347 | -0.009 | 0.496 | 0.471 | 500 140 0.4 |
| M0980 | 256 | exp02 | 2048bit-ECFP6 | DNN2 | 0.547 | 0.406 | 0.647 | 0.387 | 0.396 | 0.034 | 0.517 | 0.454 | 100 160 0.2 |
| M0981 | 256 | exp03 | 2048bit-ECFP6 | DNN2 | 0.569 | 0.436 | 0.665 | 0.415 | 0.425 | 0.081 | 0.540 | 0.470 | 600 160 0.4 |
| M0982 | 256 | exp01 | 2048bit-ECFP6 | DNN3 | 0.572 | 0.432 | 0.706 | 0.358 | 0.392 | 0.067 | 0.532 | 0.462 | 200 120 0.5 |
| M0983 | 256 | exp02 | 2048bit-ECFP6 | DNN3 | 0.562 | 0.423 | 0.671 | 0.387 | 0.404 | 0.058 | 0.529 | 0.455 | 400 80 0.2 |
| M0984 | 256 | exp03 | 2048bit-ECFP6 | DNN3 | 0.576 | 0.438 | 0.706 | 0.368 | 0.400 | 0.077 | 0.537 | 0.464 | 400 140 0.5 |
| M0985 | 128 | exp01 | 1024bit-ECFP4 | KNN | 0.554 | 0.417 | 0.647 | 0.406 | 0.411 | 0.053 | 0.526 | 0.390 | 1 uniform |
| M0986 | 128 | exp02 | 1024bit-ECFP4 | KNN | 0.543 | 0.378 | 0.700 | 0.292 | 0.330 | -0.008 | 0.496 | 0.336 | 3 distance |
| M0987 | 128 | exp03 | 1024bit-ECFP4 | KNN | 0.543 | 0.406 | 0.629 | 0.406 | 0.406 | 0.035 | 0.518 | 0.388 | 1 uniform |
| M0988 | 128 | exp04 | 1024bit-ECFP4 | KNN | 0.522 | 0.373 | 0.624 | 0.358 | 0.365 | -0.018 | 0.491 | 0.433 | 1 uniform |
| M0989 | 128 | exp05 | 1024bit-ECFP4 | KNN | 0.525 | 0.338 | 0.700 | 0.245 | 0.284 | -0.059 | 0.472 | 0.408 | 1 uniform |
| M0990 | 128 | exp06 | 1024bit-ECFP4 | KNN | 0.489 | 0.333 | 0.588 | 0.330 | 0.332 | -0.082 | 0.459 | 0.361 | 1 uniform |
| M0991 | 128 | exp07 | 1024bit-ECFP4 | KNN | 0.533 | 0.390 | 0.624 | 0.387 | 0.389 | 0.010 | 0.506 | 0.342 | 1 uniform |
| M0992 | 128 | exp08 | 1024bit-ECFP4 | KNN | 0.500 | 0.352 | 0.588 | 0.358 | 0.355 | -0.053 | 0.473 | 0.411 | 1 uniform |
| M0993 | 128 | exp09 | 1024bit-ECFP4 | KNN | 0.605 | 0.485 | 0.688 | 0.472 | 0.478 | 0.161 | 0.580 | 0.391 | 1 uniform |
| M0994 | 128 | exp10 | 1024bit-ECFP4 | KNN | 0.518 | 0.333 | 0.682 | 0.255 | 0.289 | -0.067 | 0.469 | 0.428 | 1 uniform |
| M0995 | 128 | exp11 | 1024bit-ECFP4 | KNN | 0.486 | 0.324 | 0.594 | 0.311 | 0.317 | -0.095 | 0.453 | 0.391 | 1 uniform |
| M0996 | 128 | exp12 | 1024bit-ECFP4 | KNN | 0.583 | 0.449 | 0.712 | 0.377 | 0.410 | 0.093 | 0.544 | 0.422 | 1 uniform |
| M0997 | 128 | exp13 | 1024bit-ECFP4 | KNN | 0.554 | 0.411 | 0.671 | 0.368 | 0.388 | 0.039 | 0.520 | 0.412 | 1 uniform |
| M0998 | 128 | exp14 | 1024bit-ECFP4 | KNN | 0.547 | 0.410 | 0.635 | 0.406 | 0.408 | 0.041 | 0.520 | 0.433 | 1 uniform |
| M0999 | 128 | exp15 | 1024bit-ECFP4 | KNN | 0.460 | 0.287 | 0.576 | 0.274 | 0.280 | -0.151 | 0.425 | 0.409 | 1 uniform |
| M1000 | 128 | exp16 | 1024bit-ECFP4 | KNN | 0.565 | 0.429 | 0.671 | 0.396 | 0.412 | 0.068 | 0.534 | 0.374 | 1 uniform |
| M1001 | 128 | exp17 | 1024bit-ECFP4 | KNN | 0.504 | 0.347 | 0.612 | 0.330 | 0.338 | -0.059 | 0.471 | 0.385 | 1 uniform |
| M1002 | 128 | exp18 | 1024bit-ECFP4 | KNN | 0.500 | 0.349 | 0.594 | 0.349 | 0.349 | -0.057 | 0.471 | 0.408 | 1 uniform |
| M1003 | 128 | exp19 | 1024bit-ECFP4 | KNN | 0.558 | 0.415 | 0.676 | 0.368 | 0.390 | 0.046 | 0.522 | 0.399 | 1 uniform |
| M1004 | 128 | exp01 | 1024bit-ECFP4 | SVM | 0.612 | 0.400 | 0.982 | 0.019 | 0.036 | 0.004 | 0.500 | 0.124 | 5 0.05 |
| M1005 | 128 | exp02 | 1024bit-ECFP4 | SVM | 0.623 | 1.000 | 1.000 | 0.019 | 0.037 | 0.108 | 0.509 | 0.056 | 500 0.001 |
| M1006 | 128 | exp03 | 1024bit-ECFP4 | SVM | 0.605 | 0.200 | 0.976 | 0.009 | 0.018 | -0.051 | 0.492 | 0.084 | 5000.0 0.001 |
| M1007 | 128 | exp04 | 1024bit-ECFP4 | SVM | 0.616 | 0.500 | 0.959 | 0.066 | 0.117 | 0.055 | 0.512 | 0.097 | 50000.0 1e-05 |
| M1008 | 128 | exp05 | 1024bit-ECFP4 | SVM | 0.616 | 0.000 | 1.000 | 0.000 | 0.000 | 0.000 | 0.500 | 0.056 | 100 0.01 |
| M1009 | 128 | exp06 | 1024bit-ECFP4 | SVM | 0.620 | 0.571 | 0.982 | 0.038 | 0.071 | 0.062 | 0.510 | 0.075 | 100 0.005 |
| M1010 | 128 | exp07 | 1024bit-ECFP4 | SVM | 0.612 | 0.000 | 0.994 | 0.000 | 0.000 | -0.048 | 0.497 | 0.018 | 100 0.005 |
| M1011 | 128 | exp08 | 1024bit-ECFP4 | SVM | 0.609 | 0.438 | 0.947 | 0.066 | 0.115 | 0.027 | 0.506 | 0.144 | 1000.0 0.0005 |
| M1012 | 128 | exp09 | 1024bit-ECFP4 | SVM | 0.627 | 0.636 | 0.976 | 0.066 | 0.120 | 0.106 | 0.521 | 0.178 | 100000.0 0.0005 |
| M1013 | 128 | exp10 | 1024bit-ECFP4 | SVM | 0.609 | 0.375 | 0.971 | 0.028 | 0.053 | -0.003 | 0.499 | 0.137 | 50000.0 0.0005 |
| M1014 | 128 | exp11 | 1024bit-ECFP4 | SVM | 0.612 | 0.400 | 0.982 | 0.019 | 0.036 | 0.004 | 0.500 | 0.070 | 5 0.1 |
| M1015 | 128 | exp12 | 1024bit-ECFP4 | SVM | 0.616 | 0.000 | 1.000 | 0.000 | 0.000 | 0.000 | 0.500 | 0.010 | 100000.0 0.0005 |
| M1016 | 128 | exp13 | 1024bit-ECFP4 | SVM | 0.620 | 0.526 | 0.947 | 0.094 | 0.160 | 0.080 | 0.520 | 0.155 | 10 0.05 |
| M1017 | 128 | exp14 | 1024bit-ECFP4 | SVM | 0.601 | 0.250 | 0.965 | 0.019 | 0.035 | -0.048 | 0.492 | 0.189 | 500 0.0005 |
| M1018 | 128 | exp15 | 1024bit-ECFP4 | SVM | 0.612 | 0.000 | 0.994 | 0.000 | 0.000 | -0.048 | 0.497 | 0.035 | 50000.0 0.005 |
| M1019 | 128 | exp16 | 1024bit-ECFP4 | SVM | 0.616 | 0.000 | 1.000 | 0.000 | 0.000 | 0.000 | 0.500 | 0.000 | 50 0.001 |
| M1020 | 128 | exp17 | 1024bit-ECFP4 | SVM | 0.605 | 0.000 | 0.982 | 0.000 | 0.000 | -0.083 | 0.491 | 0.039 | 5000.0 0.0005 |
| M1021 | 128 | exp18 | 1024bit-ECFP4 | SVM | 0.616 | 0.000 | 1.000 | 0.000 | 0.000 | 0.000 | 0.500 | 0.010 | 5 0.1 |
| M1022 | 128 | exp19 | 1024bit-ECFP4 | SVM | 0.601 | 0.250 | 0.965 | 0.019 | 0.035 | -0.048 | 0.492 | 0.123 | 10000.0 0.001 |
| M1023 | 128 | exp01 | 1024bit-ECFP4 | GBM | 0.543 | 0.396 | 0.659 | 0.358 | 0.376 | 0.018 | 0.508 | 0.425 | 500 0.6 |
| M1024 | 128 | exp02 | 1024bit-ECFP4 | GBM | 0.500 | 0.278 | 0.694 | 0.189 | 0.225 | -0.130 | 0.442 | 0.396 | 400 0.3 |
| M1025 | 128 | exp03 | 1024bit-ECFP4 | GBM | 0.543 | 0.400 | 0.647 | 0.377 | 0.388 | 0.025 | 0.512 | 0.459 | 500 0.6 |
| M1026 | 128 | exp04 | 1024bit-ECFP4 | GBM | 0.572 | 0.430 | 0.712 | 0.349 | 0.385 | 0.064 | 0.530 | 0.450 | 600 0.3 |
| M1027 | 128 | exp05 | 1024bit-ECFP4 | GBM | 0.547 | 0.388 | 0.694 | 0.311 | 0.346 | 0.006 | 0.502 | 0.420 | 600 0.6 |
| M1028 | 128 | exp06 | 1024bit-ECFP4 | GBM | 0.533 | 0.381 | 0.647 | 0.349 | 0.365 | -0.004 | 0.498 | 0.421 | 1000 0.2 |
| M1029 | 128 | exp07 | 1024bit-ECFP4 | GBM | 0.554 | 0.416 | 0.653 | 0.396 | 0.406 | 0.050 | 0.524 | 0.412 | 900 0.3 |
| M1030 | 128 | exp08 | 1024bit-ECFP4 | GBM | 0.518 | 0.345 | 0.665 | 0.283 | 0.311 | -0.055 | 0.474 | 0.460 | 800 0.9 |
| M1031 | 128 | exp09 | 1024bit-ECFP4 | GBM | 0.609 | 0.490 | 0.694 | 0.472 | 0.481 | 0.167 | 0.583 | 0.463 | 200 0.9 |
| M1032 | 128 | exp10 | 1024bit-ECFP4 | GBM | 0.529 | 0.370 | 0.659 | 0.321 | 0.343 | -0.021 | 0.490 | 0.464 | 500 0.9 |
| M1033 | 128 | exp11 | 1024bit-ECFP4 | GBM | 0.529 | 0.385 | 0.624 | 0.377 | 0.381 | 0.001 | 0.500 | 0.459 | 600 0.5 |
| M1034 | 128 | exp12 | 1024bit-ECFP4 | GBM | 0.630 | 0.523 | 0.759 | 0.425 | 0.469 | 0.193 | 0.592 | 0.433 | 300 0.7 |
| M1035 | 128 | exp13 | 1024bit-ECFP4 | GBM | 0.565 | 0.415 | 0.718 | 0.321 | 0.362 | 0.041 | 0.519 | 0.478 | 900 0.6 |
| M1036 | 128 | exp14 | 1024bit-ECFP4 | GBM | 0.540 | 0.387 | 0.665 | 0.340 | 0.362 | 0.004 | 0.503 | 0.455 | 100 0.8 |
| M1037 | 128 | exp15 | 1024bit-ECFP4 | GBM | 0.507 | 0.353 | 0.612 | 0.340 | 0.346 | -0.049 | 0.476 | 0.453 | 100 0.9 |
| M1038 | 128 | exp16 | 1024bit-ECFP4 | GBM | 0.547 | 0.411 | 0.629 | 0.415 | 0.413 | 0.044 | 0.522 | 0.408 | 800 0.4 |
| M1039 | 128 | exp17 | 1024bit-ECFP4 | GBM | 0.511 | 0.359 | 0.612 | 0.349 | 0.354 | -0.039 | 0.480 | 0.422 | 1000 0.9 |
| M1040 | 128 | exp18 | 1024bit-ECFP4 | GBM | 0.518 | 0.355 | 0.647 | 0.311 | 0.332 | -0.043 | 0.479 | 0.437 | 200 0.9 |
| M1041 | 128 | exp19 | 1024bit-ECFP4 | GBM | 0.580 | 0.439 | 0.729 | 0.340 | 0.383 | 0.073 | 0.534 | 0.458 | 400 0.4 |
| M1042 | 128 | exp01 | 1024bit-ECFP4 | RF | 0.511 | 0.354 | 0.624 | 0.330 | 0.341 | -0.047 | 0.477 | 0.406 | 10 |
| M1043 | 128 | exp02 | 1024bit-ECFP4 | RF | 0.543 | 0.389 | 0.676 | 0.330 | 0.357 | 0.007 | 0.503 | 0.367 | 10 |
| M1044 | 128 | exp03 | 1024bit-ECFP4 | RF | 0.504 | 0.322 | 0.653 | 0.264 | 0.290 | -0.087 | 0.459 | 0.428 | 10 |
| M1045 | 128 | exp04 | 1024bit-ECFP4 | RF | 0.533 | 0.381 | 0.647 | 0.349 | 0.365 | -0.004 | 0.498 | 0.409 | 10 |
| M1046 | 128 | exp05 | 1024bit-ECFP4 | RF | 0.554 | 0.380 | 0.741 | 0.255 | 0.305 | -0.005 | 0.498 | 0.401 | 10 |
| M1047 | 128 | exp06 | 1024bit-ECFP4 | RF | 0.536 | 0.380 | 0.665 | 0.330 | 0.354 | -0.005 | 0.498 | 0.396 | 10 |
| M1048 | 128 | exp07 | 1024bit-ECFP4 | RF | 0.569 | 0.430 | 0.688 | 0.377 | 0.402 | 0.067 | 0.532 | 0.336 | 10 |
| M1049 | 128 | exp08 | 1024bit-ECFP4 | RF | 0.475 | 0.281 | 0.624 | 0.236 | 0.256 | -0.146 | 0.430 | 0.416 | 10 |
| M1050 | 128 | exp09 | 1024bit-ECFP4 | RF | 0.605 | 0.485 | 0.700 | 0.453 | 0.468 | 0.155 | 0.577 | 0.442 | 10 |
| M1051 | 128 | exp10 | 1024bit-ECFP4 | RF | 0.525 | 0.338 | 0.700 | 0.245 | 0.284 | -0.059 | 0.472 | 0.438 | 10 |
| M1052 | 128 | exp11 | 1024bit-ECFP4 | RF | 0.572 | 0.429 | 0.718 | 0.340 | 0.379 | 0.061 | 0.529 | 0.399 | 10 |
| M1053 | 128 | exp12 | 1024bit-ECFP4 | RF | 0.591 | 0.458 | 0.735 | 0.358 | 0.402 | 0.099 | 0.546 | 0.388 | 10 |
| M1054 | 128 | exp13 | 1024bit-ECFP4 | RF | 0.562 | 0.405 | 0.724 | 0.302 | 0.346 | 0.027 | 0.513 | 0.400 | 10 |
| M1055 | 128 | exp14 | 1024bit-ECFP4 | RF | 0.565 | 0.430 | 0.665 | 0.406 | 0.417 | 0.071 | 0.536 | 0.428 | 10 |
| M1056 | 128 | exp15 | 1024bit-ECFP4 | RF | 0.518 | 0.366 | 0.624 | 0.349 | 0.357 | -0.028 | 0.486 | 0.423 | 10 |
| M1057 | 128 | exp16 | 1024bit-ECFP4 | RF | 0.598 | 0.474 | 0.706 | 0.425 | 0.448 | 0.134 | 0.566 | 0.372 | 10 |
| M1058 | 128 | exp17 | 1024bit-ECFP4 | RF | 0.518 | 0.345 | 0.665 | 0.283 | 0.311 | -0.055 | 0.474 | 0.389 | 10 |
| M1059 | 128 | exp18 | 1024bit-ECFP4 | RF | 0.536 | 0.375 | 0.676 | 0.311 | 0.340 | -0.013 | 0.494 | 0.419 | 10 |
| M1060 | 128 | exp19 | 1024bit-ECFP4 | RF | 0.536 | 0.359 | 0.706 | 0.264 | 0.304 | -0.032 | 0.485 | 0.436 | 10 |
| M1061 | 128 | exp01 | 1024bit-ECFP4 | DNN2 | 0.551 | 0.402 | 0.676 | 0.349 | 0.374 | 0.026 | 0.512 | 0.446 | 200 100 0.1 |
| M1062 | 128 | exp02 | 1024bit-ECFP4 | DNN2 | 0.514 | 0.333 | 0.671 | 0.264 | 0.295 | -0.069 | 0.468 | 0.416 | 400 100 0.5 |
| M1063 | 128 | exp03 | 1024bit-ECFP4 | DNN2 | 0.547 | 0.406 | 0.647 | 0.387 | 0.396 | 0.034 | 0.517 | 0.463 | 100 100 0.1 |
| M1064 | 128 | exp01 | 1024bit-ECFP4 | DNN3 | 0.551 | 0.412 | 0.647 | 0.396 | 0.404 | 0.044 | 0.522 | 0.443 | 300 80 0.3 |
| M1065 | 128 | exp02 | 1024bit-ECFP4 | DNN3 | 0.547 | 0.391 | 0.688 | 0.321 | 0.352 | 0.009 | 0.504 | 0.411 | 100 160 0.3 |
| M1066 | 128 | exp03 | 1024bit-ECFP4 | DNN3 | 0.525 | 0.376 | 0.629 | 0.358 | 0.367 | -0.012 | 0.493 | 0.451 | 100 80 0.2 |
| M1067 | 128 | exp01 | 2048bit-ECFP4 | KNN | 0.587 | 0.452 | 0.729 | 0.358 | 0.400 | 0.093 | 0.543 | 0.416 | 3 distance |
| M1068 | 128 | exp02 | 2048bit-ECFP4 | KNN | 0.518 | 0.369 | 0.618 | 0.358 | 0.364 | -0.024 | 0.488 | 0.325 | 1 uniform |
| M1069 | 128 | exp03 | 2048bit-ECFP4 | KNN | 0.529 | 0.385 | 0.624 | 0.377 | 0.381 | 0.001 | 0.500 | 0.370 | 1 uniform |
| M1070 | 128 | exp04 | 2048bit-ECFP4 | KNN | 0.536 | 0.390 | 0.641 | 0.368 | 0.379 | 0.009 | 0.504 | 0.426 | 1 uniform |
| M1071 | 128 | exp05 | 2048bit-ECFP4 | KNN | 0.525 | 0.363 | 0.659 | 0.311 | 0.335 | -0.031 | 0.485 | 0.397 | 1 uniform |
| M1072 | 128 | exp06 | 2048bit-ECFP4 | KNN | 0.547 | 0.400 | 0.665 | 0.358 | 0.378 | 0.024 | 0.512 | 0.371 | 1 uniform |
| M1073 | 128 | exp07 | 2048bit-ECFP4 | KNN | 0.522 | 0.377 | 0.612 | 0.377 | 0.377 | -0.011 | 0.494 | 0.382 | 1 uniform |
| M1074 | 128 | exp08 | 2048bit-ECFP4 | KNN | 0.529 | 0.387 | 0.618 | 0.387 | 0.387 | 0.004 | 0.502 | 0.397 | 1 uniform |
| M1075 | 128 | exp09 | 2048bit-ECFP4 | KNN | 0.562 | 0.429 | 0.647 | 0.425 | 0.427 | 0.072 | 0.536 | 0.399 | 1 uniform |
| M1076 | 128 | exp10 | 2048bit-ECFP4 | KNN | 0.475 | 0.299 | 0.600 | 0.274 | 0.286 | -0.129 | 0.437 | 0.417 | 1 uniform |
| M1077 | 128 | exp11 | 2048bit-ECFP4 | KNN | 0.504 | 0.337 | 0.629 | 0.302 | 0.318 | -0.070 | 0.466 | 0.398 | 1 uniform |
| M1078 | 128 | exp12 | 2048bit-ECFP4 | KNN | 0.551 | 0.413 | 0.641 | 0.406 | 0.410 | 0.047 | 0.524 | 0.372 | 1 uniform |
| M1079 | 128 | exp13 | 2048bit-ECFP4 | KNN | 0.558 | 0.415 | 0.676 | 0.368 | 0.390 | 0.046 | 0.522 | 0.408 | 1 uniform |
| M1080 | 128 | exp14 | 2048bit-ECFP4 | KNN | 0.562 | 0.432 | 0.629 | 0.453 | 0.442 | 0.082 | 0.541 | 0.399 | 1 uniform |
| M1081 | 128 | exp15 | 2048bit-ECFP4 | KNN | 0.504 | 0.347 | 0.612 | 0.330 | 0.338 | -0.059 | 0.471 | 0.382 | 1 uniform |
| M1082 | 128 | exp16 | 2048bit-ECFP4 | KNN | 0.551 | 0.420 | 0.618 | 0.443 | 0.431 | 0.060 | 0.530 | 0.386 | 1 uniform |
| M1083 | 128 | exp17 | 2048bit-ECFP4 | KNN | 0.507 | 0.356 | 0.606 | 0.349 | 0.352 | -0.045 | 0.477 | 0.374 | 1 uniform |
| M1084 | 128 | exp18 | 2048bit-ECFP4 | KNN | 0.493 | 0.330 | 0.606 | 0.311 | 0.320 | -0.084 | 0.459 | 0.400 | 1 uniform |
| M1085 | 128 | exp19 | 2048bit-ECFP4 | KNN | 0.543 | 0.386 | 0.682 | 0.321 | 0.351 | 0.003 | 0.502 | 0.419 | 1 uniform |
| M1086 | 128 | exp01 | 2048bit-ECFP4 | SVM | 0.591 | 0.182 | 0.947 | 0.019 | 0.034 | -0.085 | 0.483 | 0.183 | 50 0.005 |
| M1087 | 128 | exp02 | 2048bit-ECFP4 | SVM | 0.598 | 0.000 | 0.971 | 0.000 | 0.000 | -0.107 | 0.485 | 0.090 | 100000.0 1e-05 |
| M1088 | 128 | exp03 | 2048bit-ECFP4 | SVM | 0.605 | 0.000 | 0.982 | 0.000 | 0.000 | -0.083 | 0.491 | 0.081 | 10000.0 0.0001 |
| M1089 | 128 | exp04 | 2048bit-ECFP4 | SVM | 0.609 | 0.250 | 0.982 | 0.009 | 0.018 | -0.033 | 0.495 | 0.088 | 10000.0 0.0001 |
| M1090 | 128 | exp05 | 2048bit-ECFP4 | SVM | 0.627 | 0.636 | 0.976 | 0.066 | 0.120 | 0.106 | 0.521 | 0.101 | 500 0.001 |
| M1091 | 128 | exp06 | 2048bit-ECFP4 | SVM | 0.612 | 0.471 | 0.947 | 0.075 | 0.130 | 0.046 | 0.511 | 0.146 | 10000.0 0.0005 |
| M1092 | 128 | exp07 | 2048bit-ECFP4 | SVM | 0.620 | 0.667 | 0.994 | 0.019 | 0.037 | 0.061 | 0.506 | 0.036 | 100000.0 1e-05 |
| M1093 | 128 | exp08 | 2048bit-ECFP4 | SVM | 0.587 | 0.278 | 0.924 | 0.047 | 0.081 | -0.058 | 0.486 | 0.164 | 50000.0 1e-05 |
| M1094 | 128 | exp09 | 2048bit-ECFP4 | SVM | 0.620 | 1.000 | 1.000 | 0.009 | 0.019 | 0.076 | 0.504 | 0.043 | 5000.0 0.0005 |
| M1095 | 128 | exp10 | 2048bit-ECFP4 | SVM | 0.601 | 0.417 | 0.918 | 0.094 | 0.154 | 0.021 | 0.506 | 0.201 | 5 0.05 |
| M1096 | 128 | exp11 | 2048bit-ECFP4 | SVM | 0.605 | 0.286 | 0.971 | 0.019 | 0.035 | -0.033 | 0.495 | 0.149 | 1000.0 0.001 |
| M1097 | 128 | exp12 | 2048bit-ECFP4 | SVM | 0.601 | 0.333 | 0.953 | 0.038 | 0.068 | -0.022 | 0.495 | 0.132 | 5000.0 0.0005 |
| M1098 | 128 | exp13 | 2048bit-ECFP4 | SVM | 0.558 | 0.265 | 0.853 | 0.085 | 0.129 | -0.092 | 0.469 | 0.266 | 1000.0 0.001 |
| M1099 | 128 | exp14 | 2048bit-ECFP4 | SVM | 0.609 | 0.438 | 0.947 | 0.066 | 0.115 | 0.027 | 0.506 | 0.193 | 5000.0 0.001 |
| M1100 | 128 | exp15 | 2048bit-ECFP4 | SVM | 0.616 | 0.500 | 0.976 | 0.038 | 0.070 | 0.041 | 0.507 | 0.146 | 500 0.005 |
| M1101 | 128 | exp16 | 2048bit-ECFP4 | SVM | 0.616 | 0.000 | 1.000 | 0.000 | 0.000 | 0.000 | 0.500 | 0.034 | 100000.0 5e-05 |
| M1102 | 128 | exp17 | 2048bit-ECFP4 | SVM | 0.594 | 0.250 | 0.947 | 0.028 | 0.051 | -0.059 | 0.487 | 0.161 | 50000.0 0.0001 |
| M1103 | 128 | exp18 | 2048bit-ECFP4 | SVM | 0.616 | 0.500 | 0.971 | 0.047 | 0.086 | 0.046 | 0.509 | 0.181 | 10000.0 0.0005 |
| M1104 | 128 | exp19 | 2048bit-ECFP4 | SVM | 0.612 | 0.471 | 0.947 | 0.075 | 0.130 | 0.046 | 0.511 | 0.153 | 100000.0 1e-05 |
| M1105 | 128 | exp01 | 2048bit-ECFP4 | GBM | 0.525 | 0.366 | 0.653 | 0.321 | 0.342 | -0.027 | 0.487 | 0.452 | 600 0.3 |
| M1106 | 128 | exp02 | 2048bit-ECFP4 | GBM | 0.543 | 0.372 | 0.712 | 0.274 | 0.315 | -0.016 | 0.493 | 0.429 | 700 0.1 |
| M1107 | 128 | exp03 | 2048bit-ECFP4 | GBM | 0.504 | 0.343 | 0.618 | 0.321 | 0.332 | -0.062 | 0.470 | 0.454 | 1000 0.3 |
| M1108 | 128 | exp04 | 2048bit-ECFP4 | GBM | 0.518 | 0.333 | 0.682 | 0.255 | 0.289 | -0.067 | 0.469 | 0.463 | 50 0.9 |
| M1109 | 128 | exp05 | 2048bit-ECFP4 | GBM | 0.529 | 0.380 | 0.635 | 0.358 | 0.369 | -0.006 | 0.496 | 0.439 | 500 0.6 |
| M1110 | 128 | exp06 | 2048bit-ECFP4 | GBM | 0.562 | 0.412 | 0.706 | 0.330 | 0.366 | 0.038 | 0.518 | 0.429 | 200 0.3 |
| M1111 | 128 | exp07 | 2048bit-ECFP4 | GBM | 0.554 | 0.409 | 0.676 | 0.358 | 0.382 | 0.036 | 0.517 | 0.428 | 1000 0.8 |
| M1112 | 128 | exp08 | 2048bit-ECFP4 | GBM | 0.489 | 0.320 | 0.612 | 0.292 | 0.305 | -0.098 | 0.452 | 0.448 | 300 0.8 |
| M1113 | 128 | exp09 | 2048bit-ECFP4 | GBM | 0.580 | 0.448 | 0.688 | 0.406 | 0.426 | 0.096 | 0.547 | 0.445 | 100 0.7 |
| M1114 | 128 | exp10 | 2048bit-ECFP4 | GBM | 0.540 | 0.385 | 0.671 | 0.330 | 0.355 | 0.001 | 0.501 | 0.443 | 100 0.8 |
| M1115 | 128 | exp11 | 2048bit-ECFP4 | GBM | 0.554 | 0.390 | 0.724 | 0.283 | 0.328 | 0.007 | 0.503 | 0.450 | 200 0.4 |
| M1116 | 128 | exp12 | 2048bit-ECFP4 | GBM | 0.569 | 0.427 | 0.700 | 0.358 | 0.390 | 0.061 | 0.529 | 0.483 | 200 0.9 |
| M1117 | 128 | exp13 | 2048bit-ECFP4 | GBM | 0.562 | 0.407 | 0.718 | 0.311 | 0.353 | 0.031 | 0.514 | 0.525 | 200 0.6 |
| M1118 | 128 | exp14 | 2048bit-ECFP4 | GBM | 0.525 | 0.376 | 0.629 | 0.358 | 0.367 | -0.012 | 0.493 | 0.466 | 400 0.6 |
| M1119 | 128 | exp15 | 2048bit-ECFP4 | GBM | 0.482 | 0.324 | 0.582 | 0.321 | 0.322 | -0.097 | 0.452 | 0.463 | 500 0.9 |
| M1120 | 128 | exp16 | 2048bit-ECFP4 | GBM | 0.536 | 0.404 | 0.600 | 0.434 | 0.418 | 0.034 | 0.517 | 0.410 | 700 0.9 |
| M1121 | 128 | exp17 | 2048bit-ECFP4 | GBM | 0.554 | 0.387 | 0.729 | 0.274 | 0.320 | 0.003 | 0.502 | 0.439 | 100 0.6 |
| M1122 | 128 | exp18 | 2048bit-ECFP4 | GBM | 0.540 | 0.370 | 0.700 | 0.283 | 0.321 | -0.018 | 0.491 | 0.457 | 100 0.5 |
| M1123 | 128 | exp19 | 2048bit-ECFP4 | GBM | 0.562 | 0.397 | 0.741 | 0.274 | 0.324 | 0.016 | 0.508 | 0.492 | 100 0.8 |
| M1124 | 128 | exp01 | 2048bit-ECFP4 | RF | 0.565 | 0.394 | 0.765 | 0.245 | 0.302 | 0.011 | 0.505 | 0.418 | 50 |
| M1125 | 128 | exp02 | 2048bit-ECFP4 | RF | 0.522 | 0.359 | 0.653 | 0.311 | 0.333 | -0.037 | 0.482 | 0.396 | 10 |
| M1126 | 128 | exp03 | 2048bit-ECFP4 | RF | 0.547 | 0.402 | 0.659 | 0.368 | 0.384 | 0.027 | 0.514 | 0.411 | 10 |
| M1127 | 128 | exp04 | 2048bit-ECFP4 | RF | 0.525 | 0.374 | 0.635 | 0.349 | 0.361 | -0.016 | 0.492 | 0.409 | 10 |
| M1128 | 128 | exp05 | 2048bit-ECFP4 | RF | 0.587 | 0.446 | 0.759 | 0.311 | 0.367 | 0.077 | 0.535 | 0.414 | 10 |
| M1129 | 128 | exp06 | 2048bit-ECFP4 | RF | 0.580 | 0.443 | 0.712 | 0.368 | 0.402 | 0.083 | 0.540 | 0.379 | 10 |
| M1130 | 128 | exp07 | 2048bit-ECFP4 | RF | 0.529 | 0.367 | 0.665 | 0.311 | 0.337 | -0.025 | 0.488 | 0.403 | 10 |
| M1131 | 128 | exp08 | 2048bit-ECFP4 | RF | 0.482 | 0.305 | 0.612 | 0.274 | 0.289 | -0.117 | 0.443 | 0.394 | 10 |
| M1132 | 128 | exp09 | 2048bit-ECFP4 | RF | 0.598 | 0.474 | 0.706 | 0.425 | 0.448 | 0.134 | 0.566 | 0.398 | 10 |
| M1133 | 128 | exp10 | 2048bit-ECFP4 | RF | 0.540 | 0.385 | 0.671 | 0.330 | 0.355 | 0.001 | 0.501 | 0.428 | 10 |
| M1134 | 128 | exp11 | 2048bit-ECFP4 | RF | 0.551 | 0.395 | 0.694 | 0.321 | 0.354 | 0.016 | 0.507 | 0.423 | 10 |
| M1135 | 128 | exp12 | 2048bit-ECFP4 | RF | 0.543 | 0.396 | 0.659 | 0.358 | 0.376 | 0.018 | 0.508 | 0.425 | 10 |
| M1136 | 128 | exp13 | 2048bit-ECFP4 | RF | 0.576 | 0.434 | 0.724 | 0.340 | 0.381 | 0.067 | 0.532 | 0.426 | 10 |
| M1137 | 128 | exp14 | 2048bit-ECFP4 | RF | 0.536 | 0.385 | 0.653 | 0.349 | 0.366 | 0.002 | 0.501 | 0.445 | 10 |
| M1138 | 128 | exp15 | 2048bit-ECFP4 | RF | 0.522 | 0.365 | 0.641 | 0.330 | 0.347 | -0.029 | 0.486 | 0.405 | 10 |
| M1139 | 128 | exp16 | 2048bit-ECFP4 | RF | 0.620 | 0.505 | 0.700 | 0.491 | 0.498 | 0.192 | 0.595 | 0.397 | 10 |
| M1140 | 128 | exp17 | 2048bit-ECFP4 | RF | 0.547 | 0.388 | 0.694 | 0.311 | 0.346 | 0.006 | 0.502 | 0.395 | 10 |
| M1141 | 128 | exp18 | 2048bit-ECFP4 | RF | 0.522 | 0.362 | 0.647 | 0.321 | 0.340 | -0.033 | 0.484 | 0.418 | 10 |
| M1142 | 128 | exp19 | 2048bit-ECFP4 | RF | 0.591 | 0.448 | 0.782 | 0.283 | 0.347 | 0.074 | 0.532 | 0.432 | 10 |
| M1143 | 128 | exp01 | 2048bit-ECFP4 | DNN2 | 0.522 | 0.367 | 0.635 | 0.340 | 0.353 | -0.025 | 0.488 | 0.467 | 300 60 0.4 |
| M1144 | 128 | exp02 | 2048bit-ECFP4 | DNN2 | 0.518 | 0.366 | 0.624 | 0.349 | 0.357 | -0.028 | 0.486 | 0.444 | 100 100 0.4 |
| M1145 | 128 | exp03 | 2048bit-ECFP4 | DNN2 | 0.504 | 0.360 | 0.582 | 0.377 | 0.369 | -0.040 | 0.479 | 0.479 | 200 140 0.4 |
| M1146 | 128 | exp01 | 2048bit-ECFP4 | DNN3 | 0.518 | 0.358 | 0.641 | 0.321 | 0.338 | -0.039 | 0.481 | 0.468 | 400 60 0.4 |
| M1147 | 128 | exp02 | 2048bit-ECFP4 | DNN3 | 0.525 | 0.366 | 0.653 | 0.321 | 0.342 | -0.027 | 0.487 | 0.428 | 200 80 0.1 |
| M1148 | 128 | exp03 | 2048bit-ECFP4 | DNN3 | 0.507 | 0.361 | 0.594 | 0.368 | 0.364 | -0.038 | 0.481 | 0.469 | 200 120 0.3 |
| M1149 | 128 | exp01 | 1024bit-ECFP6 | KNN | 0.576 | 0.434 | 0.724 | 0.340 | 0.381 | 0.067 | 0.532 | 0.385 | 1 uniform |
| M1150 | 128 | exp02 | 1024bit-ECFP6 | KNN | 0.551 | 0.402 | 0.676 | 0.349 | 0.374 | 0.026 | 0.512 | 0.358 | 1 uniform |
| M1151 | 128 | exp03 | 1024bit-ECFP6 | KNN | 0.576 | 0.443 | 0.682 | 0.406 | 0.424 | 0.090 | 0.544 | 0.383 | 1 uniform |
| M1152 | 128 | exp04 | 1024bit-ECFP6 | KNN | 0.522 | 0.362 | 0.647 | 0.321 | 0.340 | -0.033 | 0.484 | 0.389 | 1 uniform |
| M1153 | 128 | exp05 | 1024bit-ECFP6 | KNN | 0.554 | 0.400 | 0.700 | 0.321 | 0.356 | 0.022 | 0.510 | 0.390 | 1 uniform |
| M1154 | 128 | exp06 | 1024bit-ECFP6 | KNN | 0.547 | 0.411 | 0.629 | 0.415 | 0.413 | 0.044 | 0.522 | 0.352 | 1 uniform |
| M1155 | 128 | exp07 | 1024bit-ECFP6 | KNN | 0.536 | 0.396 | 0.624 | 0.396 | 0.396 | 0.020 | 0.510 | 0.336 | 1 uniform |
| M1156 | 128 | exp08 | 1024bit-ECFP6 | KNN | 0.529 | 0.367 | 0.665 | 0.311 | 0.337 | -0.025 | 0.488 | 0.377 | 1 uniform |
| M1157 | 128 | exp09 | 1024bit-ECFP6 | KNN | 0.627 | 0.517 | 0.747 | 0.434 | 0.472 | 0.188 | 0.591 | 0.382 | 1 uniform |
| M1158 | 128 | exp10 | 1024bit-ECFP6 | KNN | 0.522 | 0.349 | 0.671 | 0.283 | 0.312 | -0.049 | 0.477 | 0.420 | 1 uniform |
| M1159 | 128 | exp11 | 1024bit-ECFP6 | KNN | 0.514 | 0.375 | 0.588 | 0.396 | 0.385 | -0.015 | 0.492 | 0.378 | 1 uniform |
| M1160 | 128 | exp12 | 1024bit-ECFP6 | KNN | 0.562 | 0.426 | 0.659 | 0.406 | 0.415 | 0.065 | 0.532 | 0.407 | 1 uniform |
| M1161 | 128 | exp13 | 1024bit-ECFP6 | KNN | 0.598 | 0.471 | 0.729 | 0.387 | 0.425 | 0.122 | 0.558 | 0.400 | 1 uniform |
| M1162 | 128 | exp14 | 1024bit-ECFP6 | KNN | 0.547 | 0.408 | 0.641 | 0.396 | 0.402 | 0.038 | 0.518 | 0.395 | 1 uniform |
| M1163 | 128 | exp15 | 1024bit-ECFP6 | KNN | 0.482 | 0.313 | 0.600 | 0.292 | 0.302 | -0.109 | 0.446 | 0.360 | 1 uniform |
| M1164 | 128 | exp16 | 1024bit-ECFP6 | KNN | 0.562 | 0.429 | 0.647 | 0.425 | 0.427 | 0.072 | 0.536 | 0.378 | 1 uniform |
| M1165 | 128 | exp17 | 1024bit-ECFP6 | KNN | 0.540 | 0.389 | 0.659 | 0.349 | 0.368 | 0.008 | 0.504 | 0.361 | 1 uniform |
| M1166 | 128 | exp18 | 1024bit-ECFP6 | KNN | 0.500 | 0.340 | 0.612 | 0.321 | 0.330 | -0.068 | 0.467 | 0.399 | 1 uniform |
| M1167 | 128 | exp19 | 1024bit-ECFP6 | KNN | 0.562 | 0.421 | 0.676 | 0.377 | 0.398 | 0.055 | 0.526 | 0.401 | 1 uniform |
| M1168 | 128 | exp01 | 1024bit-ECFP6 | SVM | 0.605 | 0.400 | 0.947 | 0.057 | 0.099 | 0.008 | 0.502 | 0.200 | 10000.0 0.0001 |
| M1169 | 128 | exp02 | 1024bit-ECFP6 | SVM | 0.598 | 0.333 | 0.941 | 0.047 | 0.083 | -0.025 | 0.494 | 0.160 | 100 0.001 |
| M1170 | 128 | exp03 | 1024bit-ECFP6 | SVM | 0.612 | 0.462 | 0.959 | 0.057 | 0.101 | 0.035 | 0.508 | 0.179 | 5000.0 0.0001 |
| M1171 | 128 | exp04 | 1024bit-ECFP6 | SVM | 0.565 | 0.208 | 0.888 | 0.047 | 0.077 | -0.111 | 0.468 | 0.177 | 10000.0 0.0001 |
| M1172 | 128 | exp05 | 1024bit-ECFP6 | SVM | 0.598 | 0.143 | 0.965 | 0.009 | 0.018 | -0.080 | 0.487 | 0.105 | 50000.0 1e-05 |
| M1173 | 128 | exp06 | 1024bit-ECFP6 | SVM | 0.612 | 0.467 | 0.953 | 0.066 | 0.116 | 0.041 | 0.509 | 0.120 | 50000.0 1e-05 |
| M1174 | 128 | exp07 | 1024bit-ECFP6 | SVM | 0.598 | 0.333 | 0.941 | 0.047 | 0.083 | -0.025 | 0.494 | 0.169 | 1000.0 0.0005 |
| M1175 | 128 | exp08 | 1024bit-ECFP6 | SVM | 0.601 | 0.389 | 0.935 | 0.066 | 0.113 | 0.003 | 0.501 | 0.181 | 5000.0 0.0005 |
| M1176 | 128 | exp09 | 1024bit-ECFP6 | SVM | 0.598 | 0.391 | 0.918 | 0.085 | 0.140 | 0.004 | 0.502 | 0.142 | 10000.0 5e-05 |
| M1177 | 128 | exp10 | 1024bit-ECFP6 | SVM | 0.594 | 0.417 | 0.876 | 0.142 | 0.211 | 0.026 | 0.509 | 0.294 | 100 0.01 |
| M1178 | 128 | exp11 | 1024bit-ECFP6 | SVM | 0.609 | 0.444 | 0.941 | 0.075 | 0.129 | 0.033 | 0.508 | 0.208 | 1000.0 0.0001 |
| M1179 | 128 | exp12 | 1024bit-ECFP6 | SVM | 0.612 | 0.000 | 0.994 | 0.000 | 0.000 | -0.048 | 0.497 | 0.056 | 10000.0 0.0005 |
| M1180 | 128 | exp13 | 1024bit-ECFP6 | SVM | 0.634 | 0.667 | 0.971 | 0.094 | 0.165 | 0.139 | 0.532 | 0.163 | 10 0.05 |
| M1181 | 128 | exp14 | 1024bit-ECFP6 | SVM | 0.630 | 0.600 | 0.953 | 0.113 | 0.190 | 0.124 | 0.533 | 0.177 | 10 0.005 |
| M1182 | 128 | exp15 | 1024bit-ECFP6 | SVM | 0.587 | 0.167 | 0.941 | 0.019 | 0.034 | -0.095 | 0.480 | 0.164 | 10000.0 0.0005 |
| M1183 | 128 | exp16 | 1024bit-ECFP6 | SVM | 0.616 | 0.000 | 1.000 | 0.000 | 0.000 | 0.000 | 0.500 | 0.000 | 1000.0 5e-05 |
| M1184 | 128 | exp17 | 1024bit-ECFP6 | SVM | 0.536 | 0.077 | 0.859 | 0.019 | 0.030 | -0.204 | 0.439 | 0.212 | 10000.0 0.0001 |
| M1185 | 128 | exp18 | 1024bit-ECFP6 | SVM | 0.616 | 0.000 | 1.000 | 0.000 | 0.000 | 0.000 | 0.500 | 0.032 | 10000.0 5e-05 |
| M1186 | 128 | exp19 | 1024bit-ECFP6 | SVM | 0.605 | 0.385 | 0.953 | 0.047 | 0.084 | 0.000 | 0.500 | 0.137 | 10000.0 0.0001 |
| M1187 | 128 | exp01 | 1024bit-ECFP6 | GBM | 0.533 | 0.354 | 0.700 | 0.264 | 0.303 | -0.039 | 0.482 | 0.434 | 300 0.3 |
| M1188 | 128 | exp02 | 1024bit-ECFP6 | GBM | 0.489 | 0.320 | 0.612 | 0.292 | 0.305 | -0.098 | 0.452 | 0.452 | 800 0.7 |
| M1189 | 128 | exp03 | 1024bit-ECFP6 | GBM | 0.540 | 0.382 | 0.676 | 0.321 | 0.349 | -0.003 | 0.499 | 0.452 | 200 0.9 |
| M1190 | 128 | exp04 | 1024bit-ECFP6 | GBM | 0.514 | 0.357 | 0.629 | 0.330 | 0.343 | -0.041 | 0.480 | 0.463 | 800 0.1 |
| M1191 | 128 | exp05 | 1024bit-ECFP6 | GBM | 0.533 | 0.376 | 0.659 | 0.330 | 0.352 | -0.011 | 0.495 | 0.431 | 800 0.5 |
| M1192 | 128 | exp06 | 1024bit-ECFP6 | GBM | 0.540 | 0.392 | 0.653 | 0.358 | 0.374 | 0.012 | 0.506 | 0.418 | 100 0.9 |
| M1193 | 128 | exp07 | 1024bit-ECFP6 | GBM | 0.576 | 0.434 | 0.724 | 0.340 | 0.381 | 0.067 | 0.532 | 0.441 | 200 0.4 |
| M1194 | 128 | exp08 | 1024bit-ECFP6 | GBM | 0.533 | 0.354 | 0.700 | 0.264 | 0.303 | -0.039 | 0.482 | 0.413 | 50 0.9 |
| M1195 | 128 | exp09 | 1024bit-ECFP6 | GBM | 0.551 | 0.395 | 0.694 | 0.321 | 0.354 | 0.016 | 0.507 | 0.445 | 100 0.7 |
| M1196 | 128 | exp10 | 1024bit-ECFP6 | GBM | 0.551 | 0.406 | 0.665 | 0.368 | 0.386 | 0.033 | 0.516 | 0.487 | 1000 0.9 |
| M1197 | 128 | exp11 | 1024bit-ECFP6 | GBM | 0.514 | 0.348 | 0.647 | 0.302 | 0.323 | -0.053 | 0.475 | 0.473 | 700 0.6 |
| M1198 | 128 | exp12 | 1024bit-ECFP6 | GBM | 0.580 | 0.449 | 0.682 | 0.415 | 0.431 | 0.099 | 0.548 | 0.470 | 200 0.4 |
| M1199 | 128 | exp13 | 1024bit-ECFP6 | GBM | 0.576 | 0.442 | 0.688 | 0.396 | 0.418 | 0.086 | 0.542 | 0.477 | 500 0.2 |
| M1200 | 128 | exp14 | 1024bit-ECFP6 | GBM | 0.562 | 0.419 | 0.682 | 0.368 | 0.392 | 0.052 | 0.525 | 0.452 | 100 0.9 |
| M1201 | 128 | exp15 | 1024bit-ECFP6 | GBM | 0.533 | 0.379 | 0.653 | 0.340 | 0.358 | -0.008 | 0.497 | 0.457 | 600 0.8 |
| M1202 | 128 | exp16 | 1024bit-ECFP6 | GBM | 0.562 | 0.431 | 0.635 | 0.443 | 0.437 | 0.078 | 0.539 | 0.429 | 900 0.8 |
| M1203 | 128 | exp17 | 1024bit-ECFP6 | GBM | 0.489 | 0.316 | 0.618 | 0.283 | 0.299 | -0.102 | 0.451 | 0.445 | 500 0.8 |
| M1204 | 128 | exp18 | 1024bit-ECFP6 | GBM | 0.518 | 0.352 | 0.653 | 0.302 | 0.325 | -0.047 | 0.478 | 0.476 | 600 0.9 |
| M1205 | 128 | exp19 | 1024bit-ECFP6 | GBM | 0.547 | 0.377 | 0.718 | 0.274 | 0.317 | -0.010 | 0.496 | 0.462 | 400 0.4 |
| M1206 | 128 | exp01 | 1024bit-ECFP6 | RF | 0.529 | 0.354 | 0.688 | 0.274 | 0.309 | -0.041 | 0.481 | 0.402 | 10 |
| M1207 | 128 | exp02 | 1024bit-ECFP6 | RF | 0.496 | 0.326 | 0.624 | 0.292 | 0.308 | -0.086 | 0.458 | 0.406 | 10 |
| M1208 | 128 | exp03 | 1024bit-ECFP6 | RF | 0.558 | 0.407 | 0.700 | 0.330 | 0.365 | 0.032 | 0.515 | 0.434 | 10 |
| M1209 | 128 | exp04 | 1024bit-ECFP6 | RF | 0.522 | 0.373 | 0.624 | 0.358 | 0.365 | -0.018 | 0.491 | 0.402 | 10 |
| M1210 | 128 | exp05 | 1024bit-ECFP6 | RF | 0.529 | 0.350 | 0.694 | 0.264 | 0.301 | -0.045 | 0.479 | 0.399 | 10 |
| M1211 | 128 | exp06 | 1024bit-ECFP6 | RF | 0.554 | 0.412 | 0.665 | 0.377 | 0.394 | 0.043 | 0.521 | 0.368 | 10 |
| M1212 | 128 | exp07 | 1024bit-ECFP6 | RF | 0.587 | 0.455 | 0.718 | 0.377 | 0.412 | 0.099 | 0.547 | 0.395 | 10 |
| M1213 | 128 | exp08 | 1024bit-ECFP6 | RF | 0.518 | 0.358 | 0.641 | 0.321 | 0.338 | -0.039 | 0.481 | 0.408 | 10 |
| M1214 | 128 | exp09 | 1024bit-ECFP6 | RF | 0.565 | 0.429 | 0.671 | 0.396 | 0.412 | 0.068 | 0.534 | 0.421 | 10 |
| M1215 | 128 | exp10 | 1024bit-ECFP6 | RF | 0.569 | 0.427 | 0.700 | 0.358 | 0.390 | 0.061 | 0.529 | 0.456 | 10 |
| M1216 | 128 | exp11 | 1024bit-ECFP6 | RF | 0.536 | 0.372 | 0.682 | 0.302 | 0.333 | -0.017 | 0.492 | 0.403 | 10 |
| M1217 | 128 | exp12 | 1024bit-ECFP6 | RF | 0.605 | 0.480 | 0.771 | 0.340 | 0.398 | 0.120 | 0.555 | 0.420 | 10 |
| M1218 | 128 | exp13 | 1024bit-ECFP6 | RF | 0.583 | 0.444 | 0.735 | 0.340 | 0.385 | 0.080 | 0.537 | 0.423 | 10 |
| M1219 | 128 | exp14 | 1024bit-ECFP6 | RF | 0.522 | 0.367 | 0.635 | 0.340 | 0.353 | -0.025 | 0.488 | 0.427 | 10 |
| M1220 | 128 | exp15 | 1024bit-ECFP6 | RF | 0.540 | 0.389 | 0.659 | 0.349 | 0.368 | 0.008 | 0.504 | 0.420 | 10 |
| M1221 | 128 | exp16 | 1024bit-ECFP6 | RF | 0.587 | 0.457 | 0.706 | 0.396 | 0.424 | 0.105 | 0.551 | 0.405 | 10 |
| M1222 | 128 | exp17 | 1024bit-ECFP6 | RF | 0.507 | 0.333 | 0.647 | 0.283 | 0.306 | -0.073 | 0.465 | 0.430 | 10 |
| M1223 | 128 | exp18 | 1024bit-ECFP6 | RF | 0.500 | 0.322 | 0.641 | 0.274 | 0.296 | -0.088 | 0.458 | 0.447 | 10 |
| M1224 | 128 | exp19 | 1024bit-ECFP6 | RF | 0.580 | 0.431 | 0.759 | 0.292 | 0.348 | 0.057 | 0.525 | 0.430 | 10 |
| M1225 | 128 | exp01 | 1024bit-ECFP6 | DNN2 | 0.518 | 0.366 | 0.624 | 0.349 | 0.357 | -0.028 | 0.486 | 0.441 | 200 160 0.1 |
| M1226 | 128 | exp02 | 1024bit-ECFP6 | DNN2 | 0.511 | 0.367 | 0.594 | 0.377 | 0.372 | -0.028 | 0.485 | 0.469 | 300 60 0.5 |
| M1227 | 128 | exp03 | 1024bit-ECFP6 | DNN2 | 0.522 | 0.365 | 0.641 | 0.330 | 0.347 | -0.029 | 0.486 | 0.466 | 600 160 0.1 |
| M1228 | 128 | exp01 | 1024bit-ECFP6 | DNN3 | 0.551 | 0.393 | 0.700 | 0.311 | 0.347 | 0.012 | 0.505 | 0.433 | 600 80 0.3 |
| M1229 | 128 | exp02 | 1024bit-ECFP6 | DNN3 | 0.493 | 0.307 | 0.641 | 0.255 | 0.278 | -0.109 | 0.448 | 0.445 | 400 100 0.3 |
| M1230 | 128 | exp03 | 1024bit-ECFP6 | DNN3 | 0.522 | 0.359 | 0.653 | 0.311 | 0.333 | -0.037 | 0.482 | 0.456 | 300 80 0.3 |
| M1231 | 128 | exp01 | 2048bit-ECFP6 | KNN | 0.540 | 0.370 | 0.700 | 0.283 | 0.321 | -0.018 | 0.491 | 0.388 | 1 uniform |
| M1232 | 128 | exp02 | 2048bit-ECFP6 | KNN | 0.529 | 0.360 | 0.676 | 0.292 | 0.323 | -0.033 | 0.484 | 0.365 | 1 uniform |
| M1233 | 128 | exp03 | 2048bit-ECFP6 | KNN | 0.587 | 0.459 | 0.688 | 0.425 | 0.441 | 0.115 | 0.556 | 0.366 | 1 uniform |
| M1234 | 128 | exp04 | 2048bit-ECFP6 | KNN | 0.511 | 0.321 | 0.676 | 0.245 | 0.278 | -0.084 | 0.461 | 0.395 | 1 uniform |
| M1235 | 128 | exp05 | 2048bit-ECFP6 | KNN | 0.587 | 0.452 | 0.729 | 0.358 | 0.400 | 0.093 | 0.543 | 0.388 | 1 uniform |
| M1236 | 128 | exp06 | 2048bit-ECFP6 | KNN | 0.540 | 0.392 | 0.653 | 0.358 | 0.374 | 0.012 | 0.506 | 0.373 | 1 uniform |
| M1237 | 128 | exp07 | 2048bit-ECFP6 | KNN | 0.514 | 0.368 | 0.606 | 0.368 | 0.368 | -0.026 | 0.487 | 0.331 | 1 uniform |
| M1238 | 128 | exp08 | 2048bit-ECFP6 | KNN | 0.496 | 0.301 | 0.659 | 0.236 | 0.265 | -0.112 | 0.448 | 0.356 | 1 uniform |
| M1239 | 128 | exp09 | 2048bit-ECFP6 | KNN | 0.572 | 0.433 | 0.700 | 0.368 | 0.398 | 0.070 | 0.534 | 0.385 | 1 uniform |
| M1240 | 128 | exp10 | 2048bit-ECFP6 | KNN | 0.514 | 0.341 | 0.659 | 0.283 | 0.309 | -0.061 | 0.471 | 0.403 | 1 uniform |
| M1241 | 128 | exp11 | 2048bit-ECFP6 | KNN | 0.569 | 0.427 | 0.700 | 0.358 | 0.390 | 0.061 | 0.529 | 0.371 | 1 uniform |
| M1242 | 128 | exp12 | 2048bit-ECFP6 | KNN | 0.518 | 0.364 | 0.629 | 0.340 | 0.351 | -0.031 | 0.485 | 0.362 | 1 uniform |
| M1243 | 128 | exp13 | 2048bit-ECFP6 | KNN | 0.543 | 0.378 | 0.700 | 0.292 | 0.330 | -0.008 | 0.496 | 0.400 | 1 uniform |
| M1244 | 128 | exp14 | 2048bit-ECFP6 | KNN | 0.569 | 0.441 | 0.635 | 0.462 | 0.452 | 0.097 | 0.548 | 0.414 | 1 uniform |
| M1245 | 128 | exp15 | 2048bit-ECFP6 | KNN | 0.507 | 0.344 | 0.629 | 0.311 | 0.327 | -0.061 | 0.470 | 0.366 | 1 uniform |
| M1246 | 128 | exp16 | 2048bit-ECFP6 | KNN | 0.554 | 0.398 | 0.706 | 0.311 | 0.349 | 0.018 | 0.508 | 0.389 | 1 uniform |
| M1247 | 128 | exp17 | 2048bit-ECFP6 | KNN | 0.551 | 0.398 | 0.688 | 0.330 | 0.361 | 0.019 | 0.509 | 0.386 | 1 uniform |
| M1248 | 128 | exp18 | 2048bit-ECFP6 | KNN | 0.489 | 0.308 | 0.629 | 0.264 | 0.284 | -0.110 | 0.447 | 0.382 | 1 uniform |
| M1249 | 128 | exp19 | 2048bit-ECFP6 | KNN | 0.551 | 0.393 | 0.700 | 0.311 | 0.347 | 0.012 | 0.505 | 0.359 | 1 uniform |
| M1250 | 128 | exp01 | 2048bit-ECFP6 | SVM | 0.543 | 0.222 | 0.835 | 0.075 | 0.113 | -0.129 | 0.455 | 0.325 | 5 0.1 |
| M1251 | 128 | exp02 | 2048bit-ECFP6 | SVM | 0.587 | 0.367 | 0.888 | 0.104 | 0.162 | -0.012 | 0.496 | 0.225 | 500 0.001 |
| M1252 | 128 | exp03 | 2048bit-ECFP6 | SVM | 0.598 | 0.368 | 0.929 | 0.066 | 0.112 | -0.009 | 0.498 | 0.283 | 500 0.0005 |
| M1253 | 128 | exp04 | 2048bit-ECFP6 | SVM | 0.536 | 0.176 | 0.835 | 0.057 | 0.086 | -0.160 | 0.446 | 0.253 | 5000.0 5e-05 |
| M1254 | 128 | exp05 | 2048bit-ECFP6 | SVM | 0.630 | 0.583 | 0.941 | 0.132 | 0.215 | 0.126 | 0.536 | 0.291 | 500 0.001 |
| M1255 | 128 | exp06 | 2048bit-ECFP6 | SVM | 0.638 | 0.588 | 0.918 | 0.189 | 0.286 | 0.157 | 0.553 | 0.237 | 50 0.005 |
| M1256 | 128 | exp07 | 2048bit-ECFP6 | SVM | 0.609 | 0.438 | 0.947 | 0.066 | 0.115 | 0.027 | 0.506 | 0.232 | 100000.0 1e-05 |
| M1257 | 128 | exp08 | 2048bit-ECFP6 | SVM | 0.572 | 0.350 | 0.847 | 0.132 | 0.192 | -0.029 | 0.489 | 0.319 | 50000.0 5e-05 |
| M1258 | 128 | exp09 | 2048bit-ECFP6 | SVM | 0.659 | 0.667 | 0.929 | 0.226 | 0.338 | 0.225 | 0.578 | 0.280 | 5 0.05 |
| M1259 | 128 | exp10 | 2048bit-ECFP6 | SVM | 0.583 | 0.418 | 0.812 | 0.217 | 0.286 | 0.035 | 0.515 | 0.383 | 5 0.05 |
| M1260 | 128 | exp11 | 2048bit-ECFP6 | SVM | 0.587 | 0.333 | 0.906 | 0.075 | 0.123 | -0.032 | 0.490 | 0.264 | 50000.0 5e-05 |
| M1261 | 128 | exp12 | 2048bit-ECFP6 | SVM | 0.623 | 0.536 | 0.924 | 0.142 | 0.224 | 0.105 | 0.533 | 0.289 | 100000.0 1e-05 |
| M1262 | 128 | exp13 | 2048bit-ECFP6 | SVM | 0.587 | 0.389 | 0.871 | 0.132 | 0.197 | 0.004 | 0.502 | 0.401 | 5000.0 0.0005 |
| M1263 | 128 | exp14 | 2048bit-ECFP6 | SVM | 0.572 | 0.375 | 0.824 | 0.170 | 0.234 | -0.009 | 0.497 | 0.364 | 500 0.001 |
| M1264 | 128 | exp15 | 2048bit-ECFP6 | SVM | 0.598 | 0.381 | 0.924 | 0.075 | 0.126 | -0.002 | 0.499 | 0.233 | 100 0.001 |
| M1265 | 128 | exp16 | 2048bit-ECFP6 | SVM | 0.616 | 0.500 | 0.971 | 0.047 | 0.086 | 0.046 | 0.509 | 0.165 | 500 0.001 |
| M1266 | 128 | exp17 | 2048bit-ECFP6 | SVM | 0.594 | 0.412 | 0.882 | 0.132 | 0.200 | 0.021 | 0.507 | 0.291 | 100000.0 1e-05 |
| M1267 | 128 | exp18 | 2048bit-ECFP6 | SVM | 0.583 | 0.385 | 0.859 | 0.142 | 0.207 | 0.000 | 0.500 | 0.347 | 100000.0 1e-05 |
| M1268 | 128 | exp19 | 2048bit-ECFP6 | SVM | 0.634 | 0.564 | 0.900 | 0.208 | 0.303 | 0.150 | 0.554 | 0.316 | 1 0.1 |
| M1269 | 128 | exp01 | 2048bit-ECFP6 | GBM | 0.514 | 0.321 | 0.688 | 0.236 | 0.272 | -0.082 | 0.462 | 0.472 | 300 0.2 |
| M1270 | 128 | exp02 | 2048bit-ECFP6 | GBM | 0.536 | 0.390 | 0.641 | 0.368 | 0.379 | 0.009 | 0.504 | 0.477 | 200 0.4 |
| M1271 | 128 | exp03 | 2048bit-ECFP6 | GBM | 0.562 | 0.418 | 0.688 | 0.358 | 0.386 | 0.048 | 0.523 | 0.481 | 500 0.4 |
| M1272 | 128 | exp04 | 2048bit-ECFP6 | GBM | 0.482 | 0.309 | 0.606 | 0.283 | 0.296 | -0.113 | 0.445 | 0.464 | 1000 0.5 |
| M1273 | 128 | exp05 | 2048bit-ECFP6 | GBM | 0.540 | 0.396 | 0.641 | 0.377 | 0.386 | 0.019 | 0.509 | 0.476 | 600 0.5 |
| M1274 | 128 | exp06 | 2048bit-ECFP6 | GBM | 0.569 | 0.438 | 0.653 | 0.434 | 0.436 | 0.087 | 0.543 | 0.440 | 200 0.9 |
| M1275 | 128 | exp07 | 2048bit-ECFP6 | GBM | 0.529 | 0.382 | 0.629 | 0.368 | 0.375 | -0.003 | 0.498 | 0.446 | 200 0.8 |
| M1276 | 128 | exp08 | 2048bit-ECFP6 | GBM | 0.551 | 0.404 | 0.671 | 0.358 | 0.380 | 0.030 | 0.514 | 0.475 | 400 0.6 |
| M1277 | 128 | exp09 | 2048bit-ECFP6 | GBM | 0.598 | 0.473 | 0.718 | 0.406 | 0.437 | 0.128 | 0.562 | 0.474 | 300 0.5 |
| M1278 | 128 | exp10 | 2048bit-ECFP6 | GBM | 0.576 | 0.442 | 0.688 | 0.396 | 0.418 | 0.086 | 0.542 | 0.507 | 400 0.4 |
| M1279 | 128 | exp11 | 2048bit-ECFP6 | GBM | 0.504 | 0.326 | 0.647 | 0.274 | 0.297 | -0.083 | 0.461 | 0.457 | 400 0.3 |
| M1280 | 128 | exp12 | 2048bit-ECFP6 | GBM | 0.547 | 0.402 | 0.659 | 0.368 | 0.384 | 0.027 | 0.514 | 0.466 | 300 0.4 |
| M1281 | 128 | exp13 | 2048bit-ECFP6 | GBM | 0.547 | 0.386 | 0.700 | 0.302 | 0.339 | 0.002 | 0.501 | 0.533 | 100 0.7 |
| M1282 | 128 | exp14 | 2048bit-ECFP6 | GBM | 0.554 | 0.422 | 0.629 | 0.434 | 0.428 | 0.063 | 0.531 | 0.498 | 600 0.8 |
| M1283 | 128 | exp15 | 2048bit-ECFP6 | GBM | 0.562 | 0.397 | 0.741 | 0.274 | 0.324 | 0.016 | 0.508 | 0.471 | 50 0.6 |
| M1284 | 128 | exp16 | 2048bit-ECFP6 | GBM | 0.558 | 0.386 | 0.747 | 0.255 | 0.307 | 0.002 | 0.501 | 0.442 | 100 0.3 |
| M1285 | 128 | exp17 | 2048bit-ECFP6 | GBM | 0.547 | 0.377 | 0.718 | 0.274 | 0.317 | -0.010 | 0.496 | 0.455 | 100 0.9 |
| M1286 | 128 | exp18 | 2048bit-ECFP6 | GBM | 0.533 | 0.381 | 0.647 | 0.349 | 0.365 | -0.004 | 0.498 | 0.447 | 200 0.6 |
| M1287 | 128 | exp19 | 2048bit-ECFP6 | GBM | 0.580 | 0.438 | 0.735 | 0.330 | 0.376 | 0.070 | 0.532 | 0.464 | 400 0.2 |
| M1288 | 128 | exp01 | 2048bit-ECFP6 | RF | 0.511 | 0.333 | 0.659 | 0.274 | 0.301 | -0.071 | 0.467 | 0.456 | 10 |
| M1289 | 128 | exp02 | 2048bit-ECFP6 | RF | 0.543 | 0.389 | 0.676 | 0.330 | 0.357 | 0.007 | 0.503 | 0.427 | 10 |
| M1290 | 128 | exp03 | 2048bit-ECFP6 | RF | 0.547 | 0.393 | 0.682 | 0.330 | 0.359 | 0.013 | 0.506 | 0.413 | 10 |
| M1291 | 128 | exp04 | 2048bit-ECFP6 | RF | 0.493 | 0.315 | 0.629 | 0.274 | 0.293 | -0.100 | 0.452 | 0.443 | 10 |
| M1292 | 128 | exp05 | 2048bit-ECFP6 | RF | 0.562 | 0.407 | 0.718 | 0.311 | 0.353 | 0.031 | 0.514 | 0.435 | 10 |
| M1293 | 128 | exp06 | 2048bit-ECFP6 | RF | 0.594 | 0.467 | 0.718 | 0.396 | 0.429 | 0.118 | 0.557 | 0.380 | 10 |
| M1294 | 128 | exp07 | 2048bit-ECFP6 | RF | 0.558 | 0.409 | 0.694 | 0.340 | 0.371 | 0.035 | 0.517 | 0.388 | 10 |
| M1295 | 128 | exp08 | 2048bit-ECFP6 | RF | 0.489 | 0.320 | 0.612 | 0.292 | 0.305 | -0.098 | 0.452 | 0.405 | 10 |
| M1296 | 128 | exp09 | 2048bit-ECFP6 | RF | 0.601 | 0.477 | 0.729 | 0.396 | 0.433 | 0.131 | 0.562 | 0.403 | 10 |
| M1297 | 128 | exp10 | 2048bit-ECFP6 | RF | 0.569 | 0.411 | 0.747 | 0.283 | 0.335 | 0.033 | 0.515 | 0.434 | 900 |
| M1298 | 128 | exp11 | 2048bit-ECFP6 | RF | 0.525 | 0.363 | 0.659 | 0.311 | 0.335 | -0.031 | 0.485 | 0.444 | 10 |
| M1299 | 128 | exp12 | 2048bit-ECFP6 | RF | 0.594 | 0.468 | 0.706 | 0.415 | 0.440 | 0.124 | 0.560 | 0.408 | 10 |
| M1300 | 128 | exp13 | 2048bit-ECFP6 | RF | 0.587 | 0.449 | 0.747 | 0.330 | 0.380 | 0.083 | 0.538 | 0.447 | 10 |
| M1301 | 128 | exp14 | 2048bit-ECFP6 | RF | 0.580 | 0.440 | 0.724 | 0.349 | 0.389 | 0.077 | 0.536 | 0.453 | 900 |
| M1302 | 128 | exp15 | 2048bit-ECFP6 | RF | 0.580 | 0.429 | 0.765 | 0.283 | 0.341 | 0.053 | 0.524 | 0.394 | 50 |
| M1303 | 128 | exp16 | 2048bit-ECFP6 | RF | 0.522 | 0.341 | 0.682 | 0.264 | 0.298 | -0.057 | 0.473 | 0.425 | 10 |
| M1304 | 128 | exp17 | 2048bit-ECFP6 | RF | 0.569 | 0.418 | 0.729 | 0.311 | 0.357 | 0.044 | 0.520 | 0.387 | 10 |
| M1305 | 128 | exp18 | 2048bit-ECFP6 | RF | 0.507 | 0.317 | 0.671 | 0.245 | 0.277 | -0.090 | 0.458 | 0.430 | 10 |
| M1306 | 128 | exp19 | 2048bit-ECFP6 | RF | 0.598 | 0.464 | 0.782 | 0.302 | 0.366 | 0.095 | 0.542 | 0.431 | 10 |
| M1307 | 128 | exp01 | 2048bit-ECFP6 | DNN2 | 0.533 | 0.371 | 0.671 | 0.311 | 0.338 | -0.019 | 0.491 | 0.475 | 300 160 0.2 |
| M1308 | 128 | exp02 | 2048bit-ECFP6 | DNN2 | 0.529 | 0.393 | 0.600 | 0.415 | 0.404 | 0.015 | 0.507 | 0.481 | 100 140 0.4 |
| M1309 | 128 | exp03 | 2048bit-ECFP6 | DNN2 | 0.500 | 0.337 | 0.618 | 0.311 | 0.324 | -0.072 | 0.465 | 0.489 | 400 120 0.4 |
| M1310 | 128 | exp01 | 2048bit-ECFP6 | DNN3 | 0.551 | 0.398 | 0.688 | 0.330 | 0.361 | 0.019 | 0.509 | 0.471 | 200 160 0.1 |
| M1311 | 128 | exp02 | 2048bit-ECFP6 | DNN3 | 0.511 | 0.359 | 0.612 | 0.349 | 0.354 | -0.039 | 0.480 | 0.457 | 100 160 0.2 |
| M1312 | 128 | exp03 | 2048bit-ECFP6 | DNN3 | 0.540 | 0.394 | 0.647 | 0.368 | 0.380 | 0.015 | 0.508 | 0.482 | 600 100 0.1 |

* Optimal parameters for each machine-learning methods as follows

(1) KNN: two parameters are the number of nearest neighbors (K) and weighting schemes (uniform weight or distance-dependent weight) respectively

(2) SVM: two parameters are the penalty parameter C and kernel parameter gamma respectively

(3) RF: one parameter is the number of decision trees

(4) GBM: two parameters are the number of decision trees and the learning rate

(5) DNN2 and DNN3: three parameters are number of epochs, the size of mini-batches, and the dropout rate

**Table S6.** All the average classification models by averaging over different data-splitting schemes after Y-randomization test

| **Model** | **FS** | **FP** | **method** | **Accuracy**  **(test)** | **Precision**  **(test)** | **Specificity**  **(test)** | **Sensitivity**  **(test)** | **F1-score**  **(test)** | **MCC**  **(test)** | **NER**  **(test)** | **F1-score**  **(CV)** |
| --- | --- | --- | --- | --- | --- | --- | --- | --- | --- | --- | --- |
| AM01 | full | 1024bit-ECFP4 | KNN | 0.524(0.030) | 0.380(0.035) | 0.616(0.048) | 0.377(0.053) | 0.377(0.041) | -0.007(0.059) | 0.497(0.029) | 0.391(0.025) |
| AM02 | full | 1024bit-ECFP4 | SVM | 0.618(0.007) | 0.105(0.307) | 1.000(0.000) | 0.005(0.019) | 0.009(0.035) | 0.016(0.054) | 0.502(0.009) | 0.014(0.024) |
| AM03 | full | 1024bit-ECFP4 | GBM | 0.552(0.021) | 0.397(0.036) | 0.695(0.026) | 0.323(0.043) | 0.355(0.039) | 0.018(0.050) | 0.509(0.024) | 0.380(0.021) |
| AM04 | full | 1024bit-ECFP4 | RF | 0.532(0.033) | 0.375(0.047) | 0.660(0.038) | 0.327(0.049) | 0.349(0.046) | -0.013(0.070) | 0.494(0.034) | 0.376(0.022) |
| AM05 | full | 1024bit-ECFP4 | DNN2 | 0.554(0.016) | 0.410(0.024) | 0.667(0.038) | 0.374(0.058) | 0.390(0.041) | 0.041(0.039) | 0.520(0.020) | 0.384(0.033) |
| AM06 | full | 1024bit-ECFP4 | DNN3 | 0.545(0.027) | 0.408(0.031) | 0.633(0.036) | 0.403(0.012) | 0.405(0.021) | 0.036(0.048) | 0.517(0.024) | 0.390(0.021) |
| AM07 | full | 2048bit-ECFP4 | KNN | 0.525(0.032) | 0.381(0.040) | 0.618(0.048) | 0.376(0.048) | 0.378(0.039) | -0.006(0.063) | 0.497(0.031) | 0.393(0.021) |
| AM08 | full | 2048bit-ECFP4 | SVM | 0.616(0.001) | 0.079(0.244) | 0.999(0.003) | 0.001(0.005) | 0.003(0.009) | 0.006(0.018) | 0.500(0.001) | 0.007(0.008) |
| AM09 | full | 2048bit-ECFP4 | GBM | 0.540(0.031) | 0.381(0.050) | 0.680(0.033) | 0.317(0.050) | 0.346(0.049) | -0.004(0.070) | 0.498(0.033) | 0.382(0.025) |
| AM10 | full | 2048bit-ECFP4 | RF | 0.539(0.030) | 0.380(0.046) | 0.678(0.032) | 0.318(0.048) | 0.346(0.046) | -0.005(0.065) | 0.498(0.031) | 0.359(0.026) |
| AM11 | full | 2048bit-ECFP4 | DNN2 | 0.589(0.038) | 0.130(0.184) | 0.880(0.169) | 0.123(0.173) | 0.126(0.179) | 0.003(0.004) | 0.501(0.002) | 0.186(0.153) |
| AM12 | full | 2048bit-ECFP4 | DNN3 | 0.616(0.000) | 0.000(0.000) | 1.000(0.000) | 0.000(0.000) | 0.000(0.000) | 0.000(0.000) | 0.500(0.000) | 0.051(0.072) |
| AM13 | full | 1024bit-ECFP6 | KNN | 0.506(0.056) | 0.364(0.057) | 0.593(0.081) | 0.368(0.054) | 0.365(0.050) | -0.039(0.100) | 0.480(0.051) | 0.396(0.028) |
| AM14 | full | 1024bit-ECFP6 | SVM | 0.615(0.002) | 0.100(0.253) | 0.998(0.005) | 0.002(0.005) | 0.004(0.010) | -0.001(0.026) | 0.500(0.002) | 0.019(0.023) |
| AM15 | full | 1024bit-ECFP6 | GBM | 0.554(0.032) | 0.394(0.057) | 0.709(0.040) | 0.304(0.061) | 0.342(0.058) | 0.014(0.075) | 0.507(0.035) | 0.378(0.020) |
| AM16 | full | 1024bit-ECFP6 | RF | 0.501(0.034) | 0.347(0.041) | 0.604(0.042) | 0.338(0.045) | 0.342(0.041) | -0.059(0.067) | 0.471(0.033) | 0.398(0.021) |
| AM17 | full | 1024bit-ECFP6 | DNN2 | 0.571(0.016) | 0.434(0.028) | 0.684(0.032) | 0.390(0.058) | 0.410(0.042) | 0.076(0.042) | 0.537(0.021) | 0.386(0.022) |
| AM18 | full | 1024bit-ECFP6 | DNN3 | 0.562(0.022) | 0.419(0.036) | 0.680(0.010) | 0.371(0.044) | 0.393(0.041) | 0.053(0.054) | 0.526(0.026) | 0.396(0.010) |
| AM19 | full | 2048bit-ECFP6 | KNN | 0.505(0.047) | 0.361(0.043) | 0.592(0.080) | 0.365(0.051) | 0.361(0.040) | -0.042(0.079) | 0.479(0.040) | 0.396(0.026) |
| AM20 | full | 2048bit-ECFP6 | SVM | 0.617(0.003) | 0.180(0.355) | 0.999(0.002) | 0.004(0.009) | 0.009(0.018) | 0.019(0.039) | 0.502(0.004) | 0.014(0.016) |
| AM21 | full | 2048bit-ECFP6 | GBM | 0.541(0.020) | 0.377(0.032) | 0.689(0.033) | 0.303(0.045) | 0.335(0.038) | -0.009(0.044) | 0.496(0.021) | 0.384(0.023) |
| AM22 | full | 2048bit-ECFP6 | RF | 0.508(0.025) | 0.346(0.034) | 0.629(0.038) | 0.314(0.041) | 0.329(0.036) | -0.058(0.051) | 0.472(0.025) | 0.378(0.023) |
| AM23 | full | 2048bit-ECFP6 | DNN2 | 0.598(0.025) | 0.141(0.199) | 0.890(0.155) | 0.129(0.182) | 0.135(0.190) | 0.019(0.027) | 0.510(0.014) | 0.166(0.178) |
| AM24 | full | 2048bit-ECFP6 | DNN3 | 0.616(0.000) | 0.000(0.000) | 1.000(0.000) | 0.000(0.000) | 0.000(0.000) | 0.000(0.000) | 0.500(0.000) | 0.024(0.033) |
| AM25 | 512 | 1024bit-ECFP4 | KNN | 0.531(0.030) | 0.382(0.038) | 0.642(0.039) | 0.354(0.038) | 0.367(0.036) | -0.004(0.059) | 0.498(0.029) | 0.381(0.021) |
| AM26 | 512 | 1024bit-ECFP4 | SVM | 0.616(0.002) | 0.105(0.307) | 0.999(0.002) | 0.001(0.005) | 0.003(0.009) | 0.005(0.034) | 0.500(0.002) | 0.023(0.023) |
| AM27 | 512 | 1024bit-ECFP4 | GBM | 0.535(0.026) | 0.375(0.041) | 0.673(0.030) | 0.316(0.045) | 0.342(0.042) | -0.012(0.058) | 0.494(0.028) | 0.405(0.021) |
| AM28 | 512 | 1024bit-ECFP4 | RF | 0.544(0.028) | 0.385(0.046) | 0.683(0.031) | 0.321(0.058) | 0.350(0.052) | 0.004(0.067) | 0.502(0.032) | 0.379(0.020) |
| AM29 | 512 | 1024bit-ECFP4 | DNN2 | 0.531(0.012) | 0.373(0.021) | 0.659(0.017) | 0.327(0.031) | 0.349(0.026) | -0.015(0.030) | 0.493(0.014) | 0.400(0.016) |
| AM30 | 512 | 1024bit-ECFP4 | DNN3 | 0.527(0.007) | 0.375(0.013) | 0.635(0.017) | 0.352(0.029) | 0.363(0.021) | -0.013(0.019) | 0.493(0.009) | 0.401(0.021) |
| AM31 | 512 | 2048bit-ECFP4 | KNN | 0.540(0.029) | 0.389(0.041) | 0.661(0.037) | 0.346(0.041) | 0.365(0.038) | 0.007(0.059) | 0.503(0.028) | 0.378(0.025) |
| AM32 | 512 | 2048bit-ECFP4 | SVM | 0.615(0.016) | 0.271(0.317) | 0.984(0.015) | 0.024(0.043) | 0.042(0.072) | 0.007(0.087) | 0.504(0.020) | 0.076(0.049) |
| AM33 | 512 | 2048bit-ECFP4 | GBM | 0.546(0.023) | 0.387(0.038) | 0.689(0.027) | 0.316(0.046) | 0.348(0.041) | 0.005(0.054) | 0.503(0.026) | 0.431(0.026) |
| AM34 | 512 | 2048bit-ECFP4 | RF | 0.551(0.025) | 0.392(0.041) | 0.701(0.030) | 0.309(0.039) | 0.345(0.039) | 0.011(0.056) | 0.505(0.026) | 0.397(0.029) |
| AM35 | 512 | 2048bit-ECFP4 | DNN2 | 0.552(0.024) | 0.404(0.037) | 0.678(0.019) | 0.349(0.033) | 0.374(0.035) | 0.028(0.053) | 0.514(0.026) | 0.414(0.020) |
| AM36 | 512 | 2048bit-ECFP4 | DNN3 | 0.554(0.023) | 0.413(0.036) | 0.657(0.010) | 0.390(0.051) | 0.401(0.044) | 0.047(0.057) | 0.523(0.028) | 0.425(0.022) |
| AM37 | 512 | 1024bit-ECFP6 | KNN | 0.542(0.034) | 0.394(0.045) | 0.660(0.043) | 0.352(0.040) | 0.371(0.040) | 0.013(0.068) | 0.506(0.033) | 0.383(0.025) |
| AM38 | 512 | 1024bit-ECFP6 | SVM | 0.617(0.009) | 0.273(0.392) | 0.996(0.005) | 0.010(0.023) | 0.018(0.042) | 0.014(0.068) | 0.503(0.011) | 0.038(0.030) |
| AM39 | 512 | 1024bit-ECFP6 | GBM | 0.530(0.039) | 0.369(0.055) | 0.665(0.043) | 0.312(0.043) | 0.338(0.047) | -0.023(0.080) | 0.489(0.039) | 0.415(0.025) |
| AM40 | 512 | 1024bit-ECFP6 | RF | 0.532(0.037) | 0.371(0.052) | 0.669(0.055) | 0.311(0.053) | 0.337(0.048) | -0.020(0.074) | 0.490(0.035) | 0.394(0.030) |
| AM41 | 512 | 1024bit-ECFP6 | DNN2 | 0.521(0.030) | 0.359(0.040) | 0.651(0.035) | 0.311(0.021) | 0.333(0.029) | -0.038(0.057) | 0.481(0.028) | 0.415(0.028) |
| AM42 | 512 | 1024bit-ECFP6 | DNN3 | 0.516(0.025) | 0.359(0.030) | 0.633(0.034) | 0.327(0.019) | 0.342(0.023) | -0.040(0.047) | 0.480(0.023) | 0.418(0.023) |
| AM43 | 512 | 2048bit-ECFP6 | KNN | 0.553(0.029) | 0.399(0.043) | 0.698(0.039) | 0.320(0.033) | 0.355(0.034) | 0.019(0.058) | 0.509(0.027) | 0.368(0.030) |
| AM44 | 512 | 2048bit-ECFP6 | SVM | 0.607(0.022) | 0.457(0.206) | 0.938(0.039) | 0.075(0.050) | 0.124(0.074) | 0.029(0.089) | 0.507(0.023) | 0.175(0.073) |
| AM45 | 512 | 2048bit-ECFP6 | GBM | 0.541(0.022) | 0.380(0.037) | 0.681(0.031) | 0.316(0.053) | 0.344(0.045) | -0.004(0.052) | 0.498(0.025) | 0.441(0.025) |
| AM46 | 512 | 2048bit-ECFP6 | RF | 0.532(0.032) | 0.361(0.055) | 0.685(0.032) | 0.288(0.060) | 0.319(0.057) | -0.030(0.075) | 0.486(0.036) | 0.417(0.027) |
| AM47 | 512 | 2048bit-ECFP6 | DNN2 | 0.517(0.030) | 0.351(0.043) | 0.651(0.034) | 0.302(0.038) | 0.324(0.039) | -0.048(0.063) | 0.476(0.031) | 0.444(0.026) |
| AM48 | 512 | 2048bit-ECFP6 | DNN3 | 0.524(0.012) | 0.364(0.017) | 0.651(0.018) | 0.321(0.020) | 0.341(0.018) | -0.029(0.025) | 0.486(0.012) | 0.445(0.026) |
| AM49 | 256 | 1024bit-ECFP4 | KNN | 0.537(0.037) | 0.388(0.053) | 0.648(0.038) | 0.359(0.052) | 0.373(0.051) | 0.007(0.078) | 0.503(0.038) | 0.384(0.025) |
| AM50 | 256 | 1024bit-ECFP4 | SVM | 0.609(0.013) | 0.203(0.301) | 0.983(0.023) | 0.009(0.017) | 0.017(0.030) | -0.022(0.068) | 0.496(0.011) | 0.063(0.047) |
| AM51 | 256 | 1024bit-ECFP4 | GBM | 0.541(0.024) | 0.382(0.040) | 0.677(0.035) | 0.323(0.061) | 0.349(0.050) | -0.001(0.058) | 0.500(0.028) | 0.434(0.024) |
| AM52 | 256 | 1024bit-ECFP4 | RF | 0.547(0.028) | 0.392(0.044) | 0.684(0.035) | 0.327(0.044) | 0.356(0.042) | 0.011(0.061) | 0.505(0.029) | 0.401(0.031) |
| AM53 | 256 | 1024bit-ECFP4 | DNN2 | 0.540(0.026) | 0.396(0.042) | 0.635(0.005) | 0.387(0.061) | 0.391(0.052) | 0.021(0.065) | 0.511(0.032) | 0.418(0.030) |
| AM54 | 256 | 1024bit-ECFP4 | DNN3 | 0.559(0.011) | 0.421(0.025) | 0.655(0.022) | 0.406(0.061) | 0.412(0.044) | 0.060(0.041) | 0.530(0.020) | 0.420(0.036) |
| AM55 | 256 | 2048bit-ECFP4 | KNN | 0.541(0.031) | 0.390(0.046) | 0.662(0.046) | 0.347(0.062) | 0.365(0.051) | 0.009(0.066) | 0.505(0.032) | 0.379(0.026) |
| AM56 | 256 | 2048bit-ECFP4 | SVM | 0.610(0.009) | 0.350(0.219) | 0.970(0.029) | 0.033(0.032) | 0.057(0.053) | 0.006(0.049) | 0.501(0.008) | 0.111(0.062) |
| AM57 | 256 | 2048bit-ECFP4 | GBM | 0.545(0.025) | 0.391(0.043) | 0.670(0.031) | 0.344(0.062) | 0.365(0.053) | 0.013(0.062) | 0.507(0.030) | 0.453(0.025) |
| AM58 | 256 | 2048bit-ECFP4 | RF | 0.554(0.026) | 0.399(0.043) | 0.698(0.036) | 0.321(0.051) | 0.355(0.045) | 0.021(0.059) | 0.510(0.028) | 0.407(0.027) |
| AM59 | 256 | 2048bit-ECFP4 | DNN2 | 0.525(0.009) | 0.371(0.012) | 0.639(0.037) | 0.343(0.047) | 0.355(0.030) | -0.018(0.019) | 0.491(0.009) | 0.432(0.019) |
| AM60 | 256 | 2048bit-ECFP4 | DNN3 | 0.552(0.019) | 0.402(0.022) | 0.686(0.038) | 0.337(0.027) | 0.366(0.019) | 0.024(0.031) | 0.511(0.015) | 0.429(0.021) |
| AM61 | 256 | 1024bit-ECFP6 | KNN | 0.534(0.031) | 0.383(0.040) | 0.655(0.045) | 0.342(0.037) | 0.360(0.035) | -0.003(0.060) | 0.498(0.029) | 0.382(0.025) |
| AM62 | 256 | 1024bit-ECFP6 | SVM | 0.607(0.015) | 0.330(0.218) | 0.968(0.024) | 0.028(0.023) | 0.051(0.040) | -0.007(0.067) | 0.498(0.014) | 0.104(0.050) |
| AM63 | 256 | 1024bit-ECFP6 | GBM | 0.536(0.033) | 0.381(0.045) | 0.667(0.043) | 0.328(0.041) | 0.352(0.041) | -0.006(0.067) | 0.497(0.032) | 0.444(0.025) |
| AM64 | 256 | 1024bit-ECFP6 | RF | 0.532(0.035) | 0.377(0.047) | 0.661(0.048) | 0.326(0.038) | 0.349(0.038) | -0.013(0.068) | 0.493(0.033) | 0.410(0.030) |
| AM65 | 256 | 1024bit-ECFP6 | DNN2 | 0.524(0.024) | 0.371(0.032) | 0.639(0.026) | 0.340(0.020) | 0.354(0.025) | -0.021(0.048) | 0.490(0.023) | 0.446(0.010) |
| AM66 | 256 | 1024bit-ECFP6 | DNN3 | 0.542(0.017) | 0.395(0.020) | 0.659(0.030) | 0.355(0.019) | 0.374(0.016) | 0.015(0.030) | 0.507(0.015) | 0.443(0.018) |
| AM67 | 256 | 2048bit-ECFP6 | KNN | 0.550(0.027) | 0.397(0.040) | 0.691(0.031) | 0.326(0.036) | 0.357(0.036) | 0.017(0.056) | 0.508(0.027) | 0.374(0.025) |
| AM68 | 256 | 2048bit-ECFP6 | SVM | 0.602(0.026) | 0.451(0.178) | 0.904(0.038) | 0.118(0.057) | 0.180(0.082) | 0.033(0.088) | 0.511(0.028) | 0.248(0.077) |
| AM69 | 256 | 2048bit-ECFP6 | GBM | 0.536(0.021) | 0.379(0.032) | 0.667(0.031) | 0.326(0.039) | 0.350(0.034) | -0.007(0.045) | 0.497(0.022) | 0.477(0.025) |
| AM70 | 256 | 2048bit-ECFP6 | RF | 0.549(0.029) | 0.393(0.044) | 0.691(0.039) | 0.320(0.045) | 0.352(0.042) | 0.012(0.062) | 0.506(0.029) | 0.425(0.026) |
| AM71 | 256 | 2048bit-ECFP6 | DNN2 | 0.551(0.014) | 0.407(0.024) | 0.661(0.010) | 0.374(0.039) | 0.389(0.032) | 0.035(0.037) | 0.518(0.018) | 0.465(0.008) |
| AM72 | 256 | 2048bit-ECFP6 | DNN3 | 0.570(0.006) | 0.431(0.006) | 0.694(0.016) | 0.371(0.012) | 0.399(0.005) | 0.067(0.008) | 0.533(0.003) | 0.460(0.004) |
| AM73 | 128 | 1024bit-ECFP4 | KNN | 0.531(0.035) | 0.380(0.048) | 0.643(0.043) | 0.352(0.057) | 0.365(0.051) | -0.005(0.074) | 0.497(0.037) | 0.396(0.027) |
| AM74 | 128 | 1024bit-ECFP4 | SVM | 0.613(0.007) | 0.292(0.277) | 0.980(0.017) | 0.024(0.028) | 0.044(0.049) | 0.006(0.054) | 0.502(0.009) | 0.084(0.058) |
| AM75 | 128 | 1024bit-ECFP4 | GBM | 0.546(0.033) | 0.396(0.052) | 0.669(0.040) | 0.349(0.058) | 0.370(0.053) | 0.018(0.073) | 0.509(0.035) | 0.441(0.022) |
| AM76 | 128 | 1024bit-ECFP4 | RF | 0.545(0.033) | 0.388(0.051) | 0.681(0.036) | 0.327(0.060) | 0.354(0.055) | 0.007(0.074) | 0.504(0.036) | 0.405(0.026) |
| AM77 | 128 | 1024bit-ECFP4 | DNN2 | 0.537(0.017) | 0.380(0.034) | 0.665(0.013) | 0.333(0.051) | 0.355(0.043) | -0.003(0.047) | 0.499(0.022) | 0.441(0.019) |
| AM78 | 128 | 1024bit-ECFP4 | DNN3 | 0.541(0.011) | 0.393(0.015) | 0.655(0.025) | 0.358(0.031) | 0.374(0.022) | 0.014(0.023) | 0.506(0.012) | 0.435(0.017) |
| AM79 | 128 | 2048bit-ECFP4 | KNN | 0.532(0.027) | 0.384(0.038) | 0.637(0.032) | 0.362(0.047) | 0.372(0.040) | -0.001(0.058) | 0.500(0.029) | 0.391(0.023) |
| AM80 | 128 | 2048bit-ECFP4 | SVM | 0.605(0.015) | 0.365(0.243) | 0.958(0.034) | 0.039(0.030) | 0.067(0.049) | -0.005(0.062) | 0.498(0.012) | 0.134(0.060) |
| AM81 | 128 | 2048bit-ECFP4 | GBM | 0.538(0.026) | 0.382(0.033) | 0.672(0.046) | 0.323(0.046) | 0.349(0.036) | -0.004(0.050) | 0.498(0.024) | 0.453(0.025) |
| AM82 | 128 | 2048bit-ECFP4 | RF | 0.552(0.033) | 0.401(0.046) | 0.686(0.046) | 0.337(0.053) | 0.365(0.046) | 0.024(0.067) | 0.511(0.033) | 0.411(0.016) |
| AM83 | 128 | 2048bit-ECFP4 | DNN2 | 0.515(0.008) | 0.364(0.003) | 0.614(0.023) | 0.355(0.016) | 0.360(0.007) | -0.031(0.006) | 0.484(0.004) | 0.463(0.015) |
| AM84 | 128 | 2048bit-ECFP4 | DNN3 | 0.517(0.007) | 0.362(0.003) | 0.629(0.025) | 0.337(0.022) | 0.348(0.011) | -0.035(0.005) | 0.483(0.003) | 0.455(0.019) |
| AM85 | 128 | 1024bit-ECFP6 | KNN | 0.548(0.033) | 0.403(0.046) | 0.662(0.042) | 0.364(0.046) | 0.382(0.043) | 0.027(0.068) | 0.513(0.033) | 0.382(0.021) |
| AM86 | 128 | 1024bit-ECFP6 | SVM | 0.601(0.021) | 0.310(0.194) | 0.944(0.037) | 0.052(0.038) | 0.088(0.062) | -0.009(0.076) | 0.498(0.020) | 0.151(0.066) |
| AM87 | 128 | 1024bit-ECFP6 | GBM | 0.539(0.026) | 0.383(0.038) | 0.668(0.031) | 0.332(0.049) | 0.355(0.043) | -0.000(0.057) | 0.500(0.028) | 0.451(0.020) |
| AM88 | 128 | 1024bit-ECFP6 | RF | 0.547(0.032) | 0.394(0.047) | 0.682(0.042) | 0.330(0.041) | 0.358(0.040) | 0.013(0.065) | 0.506(0.031) | 0.416(0.020) |
| AM89 | 128 | 1024bit-ECFP6 | DNN2 | 0.517(0.005) | 0.366(0.001) | 0.620(0.019) | 0.352(0.019) | 0.359(0.010) | -0.028(0.000) | 0.486(0.000) | 0.458(0.013) |
| AM90 | 128 | 1024bit-ECFP6 | DNN3 | 0.522(0.024) | 0.353(0.035) | 0.665(0.025) | 0.292(0.026) | 0.319(0.030) | -0.045(0.050) | 0.478(0.023) | 0.445(0.009) |
| AM91 | 128 | 2048bit-ECFP6 | KNN | 0.539(0.029) | 0.381(0.046) | 0.672(0.033) | 0.326(0.056) | 0.351(0.050) | -0.002(0.066) | 0.499(0.032) | 0.378(0.019) |
| AM92 | 128 | 2048bit-ECFP6 | SVM | 0.597(0.031) | 0.424(0.121) | 0.892(0.045) | 0.126(0.055) | 0.190(0.075) | 0.027(0.092) | 0.509(0.031) | 0.289(0.058) |
| AM93 | 128 | 2048bit-ECFP6 | GBM | 0.547(0.027) | 0.394(0.041) | 0.676(0.041) | 0.339(0.059) | 0.363(0.049) | 0.015(0.059) | 0.508(0.029) | 0.471(0.023) |
| AM94 | 128 | 2048bit-ECFP6 | RF | 0.554(0.036) | 0.401(0.054) | 0.702(0.044) | 0.318(0.045) | 0.354(0.046) | 0.021(0.074) | 0.510(0.035) | 0.421(0.023) |
| AM95 | 128 | 2048bit-ECFP6 | DNN2 | 0.521(0.015) | 0.367(0.023) | 0.630(0.030) | 0.346(0.049) | 0.355(0.035) | -0.025(0.036) | 0.488(0.017) | 0.482(0.006) |
| AM96 | 128 | 2048bit-ECFP6 | DNN3 | 0.534(0.017) | 0.384(0.018) | 0.649(0.031) | 0.349(0.016) | 0.365(0.011) | -0.002(0.026) | 0.499(0.013) | 0.470(0.010) |

**Table S7.** Nineteen individual models (from **Table S2**) used to construct the consensus model 1 (**CM01**) for the sweetener/non-sweetener classification

| **Model** | **FS** | **Exp** | **FP** | **Method** | **Accuracy**  **(test)** | **Precision**  **(test)** | **Specificity**  **(test)** | **Sensitivity**  **(test)** | **F1-score (test)** | **MCC**  **(test)** | **F1-score**  **(CV)** | **∆F1-score** | **Parameters from CV** |
| --- | --- | --- | --- | --- | --- | --- | --- | --- | --- | --- | --- | --- | --- |
| M0020 | full | exp01 | 1024bit_ECFP4 | SVM | 0.899 | 0.924 | 0.959 | 0.802 | 0.859 | 0.785 | 0.847 | 0.012 | 5 0.01 |
| M0325 | 512 | exp02 | 1024bit_ECFP4 | SVM | 0.902 | 0.891 | 0.935 | 0.849 | 0.870 | 0.792 | 0.848 | 0.022 | 10000.0 0.05 |
| M0022 | full | exp03 | 1024bit_ECFP4 | SVM | 0.906 | 0.908 | 0.947 | 0.840 | 0.873 | 0.800 | 0.842 | 0.031 | 100 0.05 |
| M1030 | 128 | exp04 | 2048bit_ECFP4 | GBM | 0.909 | 0.886 | 0.929 | 0.877 | 0.882 | 0.808 | 0.839 | 0.043 | 50 0.8 |
| M0708 | 256 | exp05 | 2048bit_ECFP4 | SVM | 0.888 | 0.850 | 0.906 | 0.858 | 0.854 | 0.763 | 0.873 | 0.019 | 100 0.1 |
| M1165 | 128 | exp06 | 2048bit_ECFP6 | SVM | 0.909 | 0.901 | 0.941 | 0.858 | 0.879 | 0.807 | 0.855 | 0.024 | 5 0.1 |
| M0995 | 128 | exp07 | 2048bit_ECFP4 | KNN | 0.909 | 0.918 | 0.953 | 0.840 | 0.877 | 0.807 | 0.828 | 0.049 | 5 distance |
| M0540 | 512 | exp08 | 2048bit_ECFP6 | KNN | 0.902 | 0.862 | 0.912 | 0.887 | 0.874 | 0.795 | 0.843 | 0.031 | 1 uniform |
| M0959 | 128 | exp09 | 1024bit_ECFP4 | GBM | 0.920 | 0.920 | 0.953 | 0.868 | 0.893 | 0.831 | 0.845 | 0.048 | 300 0.1 |
| M0333 | 512 | exp10 | 1024bit_ECFP4 | SVM | 0.917 | 0.937 | 0.965 | 0.840 | 0.886 | 0.823 | 0.844 | 0.042 | 50000.0 0.05 |
| M0847 | 256 | exp11 | 2048bit_ECFP6 | KNN | 0.902 | 0.891 | 0.935 | 0.849 | 0.870 | 0.792 | 0.847 | 0.023 | 3 distance |
| M1095 | 128 | exp12 | 1024bit_ECFP6 | SVM | 0.928 | 0.948 | 0.971 | 0.858 | 0.901 | 0.847 | 0.830 | 0.071 | 5 0.1 |
| M0678 | 256 | exp13 | 1024bit_ECFP4 | RF | 0.913 | 0.902 | 0.941 | 0.868 | 0.885 | 0.815 | 0.835 | 0.050 | 50 |
| M0869 | 256 | exp14 | 2048bit_ECFP6 | SVM | 0.888 | 0.844 | 0.900 | 0.868 | 0.856 | 0.764 | 0.878 | 0.022 | 100 0.1 |
| M0338 | 512 | exp15 | 1024bit_ECFP4 | SVM | 0.913 | 0.946 | 0.971 | 0.821 | 0.879 | 0.816 | 0.846 | 0.033 | 50 0.05 |
| M0415 | 512 | exp16 | 2048bit_ECFP4 | SVM | 0.891 | 0.904 | 0.947 | 0.802 | 0.850 | 0.769 | 0.862 | 0.012 | 1000.0 0.1 |
| M0948 | 128 | exp17 | 1024bit_ECFP4 | SVM | 0.899 | 0.868 | 0.918 | 0.868 | 0.868 | 0.786 | 0.845 | 0.023 | 5 0.1 |
| M1063 | 128 | exp18 | 2048bit_ECFP4 | RF | 0.906 | 0.917 | 0.953 | 0.830 | 0.871 | 0.800 | 0.847 | 0.024 | 200 |
| M0266 | full | exp19 | 2048bit_ECFP6 | SVM | 0.931 | 0.931 | 0.959 | 0.887 | 0.908 | 0.854 | 0.832 | 0.076 | 5 0.01 |

**Table S8.** Nineteen individual models (from **Table S2**) used to construct the consensus model 2 (**CM02**) for the sweetener/non-sweetener classification

| **Model** | **FS** | **Exp** | **FP** | **Method** | **Accuracy**  **(test)** | **Precision**  **(test)** | **Specificity**  **(test)** | **Sensitivity**  **(test)** | **F1-score (test)** | **MCC**  **(test)** | **F1-score**  **(CV)** | **∆F1-score** | **Parameters from CV** |
| --- | --- | --- | --- | --- | --- | --- | --- | --- | --- | --- | --- | --- | --- |
| M0020 | full | exp01 | 1024bit_ECFP4 | SVM | 0.899 | 0.924 | 0.959 | 0.802 | 0.859 | 0.785 | 0.847 | 0.012 | 5 0.01 |
| M1062 | 128 | exp02 | 1024bit_ECFP4 | DNN2 | 0.920 | 0.904 | 0.941 | 0.887 | 0.895 | 0.831 | 0.831 | 0.064 | 200 60 0.2 |
| M0164 | full | exp03 | 2048bit_ECFP4 | DNN3 | 0.920 | 0.896 | 0.935 | 0.896 | 0.896 | 0.832 | 0.837 | 0.059 | 100 80 0.5 |
| M1030 | 128 | exp04 | 2048bit_ECFP4 | GBM | 0.909 | 0.886 | 0.929 | 0.877 | 0.882 | 0.808 | 0.839 | 0.043 | 50 0.8 |
| M0708 | 256 | exp05 | 2048bit_ECFP4 | SVM | 0.888 | 0.850 | 0.906 | 0.858 | 0.854 | 0.763 | 0.873 | 0.019 | 100 0.1 |
| M1165 | 128 | exp06 | 2048bit_ECFP6 | SVM | 0.909 | 0.901 | 0.941 | 0.858 | 0.879 | 0.807 | 0.855 | 0.024 | 5 0.1 |
| M0995 | 128 | exp07 | 2048bit_ECFP4 | KNN | 0.909 | 0.918 | 0.953 | 0.840 | 0.877 | 0.807 | 0.828 | 0.049 | 5 distance |
| M0540 | 512 | exp08 | 2048bit_ECFP6 | KNN | 0.902 | 0.862 | 0.912 | 0.887 | 0.874 | 0.795 | 0.843 | 0.031 | 1 uniform |
| M0959 | 128 | exp09 | 1024bit_ECFP4 | GBM | 0.920 | 0.920 | 0.953 | 0.868 | 0.893 | 0.831 | 0.845 | 0.048 | 300 0.1 |
| M0333 | 512 | exp10 | 1024bit_ECFP4 | SVM | 0.917 | 0.937 | 0.965 | 0.840 | 0.886 | 0.823 | 0.844 | 0.042 | 50000.0 0.05 |
| M0847 | 256 | exp11 | 2048bit_ECFP6 | KNN | 0.902 | 0.891 | 0.935 | 0.849 | 0.870 | 0.792 | 0.847 | 0.023 | 3 distance |
| M1095 | 128 | exp12 | 1024bit_ECFP6 | SVM | 0.928 | 0.948 | 0.971 | 0.858 | 0.901 | 0.847 | 0.830 | 0.071 | 5 0.1 |
| M0678 | 256 | exp13 | 1024bit_ECFP4 | RF | 0.913 | 0.902 | 0.941 | 0.868 | 0.885 | 0.815 | 0.835 | 0.050 | 50 |
| M0869 | 256 | exp14 | 2048bit_ECFP6 | SVM | 0.888 | 0.844 | 0.900 | 0.868 | 0.856 | 0.764 | 0.878 | 0.022 | 100 0.1 |
| M0338 | 512 | exp15 | 1024bit_ECFP4 | SVM | 0.913 | 0.946 | 0.971 | 0.821 | 0.879 | 0.816 | 0.846 | 0.033 | 50 0.05 |
| M0415 | 512 | exp16 | 2048bit_ECFP4 | SVM | 0.891 | 0.904 | 0.947 | 0.802 | 0.850 | 0.769 | 0.862 | 0.012 | 1000.0 0.1 |
| M0948 | 128 | exp17 | 1024bit_ECFP4 | SVM | 0.899 | 0.868 | 0.918 | 0.868 | 0.868 | 0.786 | 0.845 | 0.023 | 5 0.1 |
| M1063 | 128 | exp18 | 2048bit_ECFP4 | RF | 0.906 | 0.917 | 0.953 | 0.830 | 0.871 | 0.800 | 0.847 | 0.024 | 200 |
| M0266 | full | exp19 | 2048bit_ECFP6 | SVM | 0.931 | 0.931 | 0.959 | 0.887 | 0.908 | 0.854 | 0.832 | 0.076 | 5 0.01 |

**Table S9.** Five average models (from **Table S3**) used to construct the consensus model 3 (**CM03**) for the sweetener/non-sweetener classification

| **Model** | **FS** | **FP** | **method** | **Accuracy**  **(test)** | **Precision**  **(test)** | **Specificity**  **(test)** | **Sensitivity**  **(test)** | **F1-score**  **(test)** | **MCC**  **(test)** | **F1-score**  **(CV)** | **∆F1-score** |
| --- | --- | --- | --- | --- | --- | --- | --- | --- | --- | --- | --- |
| AM29 | 512 | 1024bit-ECFP4 | DNN2 | 0.897(0.006) | 0.904(0.018) | 0.945(0.012) | 0.821(0.021) | 0.860(0.009) | 0.782(0.013) | 0.842(0.003) | 0.018(0.010) |
| AM26 | 512 | 1024bit-ECFP4 | SVM | 0.893(0.017) | 0.887(0.032) | 0.933(0.021) | 0.830(0.031) | 0.857(0.023) | 0.774(0.037) | 0.849(0.006) | 0.026(0.011) |
| AM77 | 128 | 1024bit-ECFP4 | DNN2 | 0.891(0.021) | 0.877(0.019) | 0.928(0.010) | 0.833(0.040) | 0.855(0.029) | 0.769(0.045) | 0.839(0.006) | 0.027(0.027) |
| AM50 | 256 | 1024bit-ECFP4 | SVM | 0.892(0.012) | 0.881(0.026) | 0.929(0.019) | 0.832(0.033) | 0.855(0.017) | 0.771(0.025) | 0.853(0.006) | 0.014(0.015) |
| AM86 | 128 | 1024bit-ECFP6 | SVM | 0.891(0.016) | 0.880(0.033) | 0.929(0.024) | 0.831(0.036) | 0.854(0.021) | 0.769(0.033) | 0.848(0.009) | 0.024(0.017) |

**Table S10.** Five average models (from **Table S3**) used to construct the consensus model 4 (**CM04**) for the sweetener/non-sweetener classification.

| **Model** | **FS** | **FP** | **method** | **Accuracy**  **(test)** | **Precision**  **(test)** | **Specificity**  **(test)** | **Sensitivity**  **(test)** | **F1-score**  **(test)** | **MCC**  **(test)** | **F1-score**  **(CV)** | **∆F1-score** |
| --- | --- | --- | --- | --- | --- | --- | --- | --- | --- | --- | --- |
| AM29 | 512 | 1024bit-ECFP4 | DNN2 | 0.897(0.006) | 0.904(0.018) | 0.945(0.012) | 0.821(0.021) | 0.860(0.009) | 0.782(0.013) | 0.842(0.003) | 0.018(0.010) |
| AM26 | 512 | 1024bit-ECFP4 | SVM | 0.893(0.017) | 0.887(0.032) | 0.933(0.021) | 0.830(0.031) | 0.857(0.023) | 0.774(0.037) | 0.849(0.006) | 0.026(0.011) |
| AM28 | 512 | 1024bit-ECFP4 | RF | 0.892(0.015) | 0.917(0.026) | 0.955(0.016) | 0.791(0.033) | 0.849(0.021) | 0.771(0.031) | 0.841(0.007) | 0.022(0.016) |
| AM75 | 128 | 1024bit-ECFP4 | GBM | 0.884(0.019) | 0.869(0.035) | 0.922(0.024) | 0.822(0.037) | 0.844(0.026) | 0.753(0.040) | 0.843(0.008) | 0.026(0.015) |
| AM67 | 256 | 1024bit-ECFP6 | KNN | 0.878(0.017) | 0.863(0.040) | 0.918(0.028) | 0.816(0.029) | 0.838(0.021) | 0.743(0.035) | 0.839(0.008) | 0.022(0.013) |

**Table S11.** All the individual regression models in this work

| **Model** | **FS** | **EXP** | **FP** | **Method** | **R^2^**  **(test)** | **MSE**  **(test)** | **MAE**  **(test)** | **R^2^**  **(CV)** | $\boldsymbol{\Delta}$**R^2^** | **Parameters from CV*** |
| --- | --- | --- | --- | --- | --- | --- | --- | --- | --- | --- |
| M0001 | full | exp01 | 1024bit-ECFP4 | KNN | 0.581 | 0.520 | 0.499 | 0.526 | 0.055 | 3 distance |
| M0002 | full | exp02 | 1024bit-ECFP4 | KNN | 0.647 | 0.412 | 0.458 | 0.581 | 0.066 | 3 distance |
| M0003 | full | exp03 | 1024bit-ECFP4 | KNN | 0.639 | 0.421 | 0.449 | 0.515 | 0.124 | 3 distance |
| M0004 | full | exp04 | 1024bit-ECFP4 | KNN | 0.626 | 0.487 | 0.469 | 0.541 | 0.085 | 1 distance |
| M0005 | full | exp05 | 1024bit-ECFP4 | KNN | 0.711 | 0.378 | 0.441 | 0.556 | 0.155 | 3 distance |
| M0006 | full | exp06 | 1024bit-ECFP4 | KNN | 0.651 | 0.462 | 0.475 | 0.525 | 0.126 | 1 uniform |
| M0007 | full | exp07 | 1024bit-ECFP4 | KNN | 0.453 | 0.704 | 0.512 | 0.309 | 0.144 | 3 distance |
| M0008 | full | exp08 | 1024bit-ECFP4 | KNN | 0.487 | 0.652 | 0.535 | 0.393 | 0.094 | 3 distance |
| M0009 | full | exp09 | 1024bit-ECFP4 | KNN | 0.469 | 0.743 | 0.590 | 0.519 | 0.050 | 1 uniform |
| M0010 | full | exp10 | 1024bit-ECFP4 | KNN | 0.794 | 0.271 | 0.407 | 0.542 | 0.252 | 1 distance |
| M0011 | full | exp11 | 1024bit-ECFP4 | KNN | 0.576 | 0.509 | 0.497 | 0.551 | 0.025 | 1 uniform |
| M0012 | full | exp12 | 1024bit-ECFP4 | KNN | 0.784 | 0.245 | 0.380 | 0.563 | 0.221 | 1 uniform |
| M0013 | full | exp13 | 1024bit-ECFP4 | KNN | 0.610 | 0.496 | 0.502 | 0.547 | 0.063 | 1 uniform |
| M0014 | full | exp14 | 1024bit-ECFP4 | KNN | 0.293 | 1.078 | 0.608 | 0.394 | 0.101 | 1 uniform |
| M0015 | full | exp15 | 1024bit-ECFP4 | KNN | 0.446 | 0.735 | 0.538 | 0.664 | 0.218 | 1 distance |
| M0016 | full | exp16 | 1024bit-ECFP4 | KNN | 0.495 | 0.632 | 0.529 | 0.416 | 0.079 | 1 distance |
| M0017 | full | exp17 | 1024bit-ECFP4 | KNN | 0.592 | 0.481 | 0.478 | 0.495 | 0.097 | 3 distance |
| M0018 | full | exp18 | 1024bit-ECFP4 | KNN | 0.459 | 0.801 | 0.576 | 0.585 | 0.126 | 1 distance |
| M0019 | full | exp19 | 1024bit-ECFP4 | KNN | 0.506 | 0.703 | 0.513 | 0.609 | 0.103 | 1 uniform |
| M0020 | full | exp01 | 1024bit-ECFP4 | SVM | 0.764 | 0.280 | 0.422 | 0.740 | 0.024 | 10 0.01 |
| M0021 | full | exp02 | 1024bit-ECFP4 | SVM | 0.796 | 0.264 | 0.393 | 0.759 | 0.037 | 10 0.01 |
| M0022 | full | exp03 | 1024bit-ECFP4 | SVM | 0.809 | 0.236 | 0.371 | 0.733 | 0.076 | 10000.0 0.01 |
| M0023 | full | exp04 | 1024bit-ECFP4 | SVM | 0.779 | 0.280 | 0.413 | 0.746 | 0.033 | 10 0.01 |
| M0024 | full | exp05 | 1024bit-ECFP4 | SVM | 0.811 | 0.250 | 0.365 | 0.725 | 0.086 | 100 0.01 |
| M0025 | full | exp06 | 1024bit-ECFP4 | SVM | 0.758 | 0.272 | 0.374 | 0.740 | 0.018 | 500 0.01 |
| M0026 | full | exp07 | 1024bit-ECFP4 | SVM | 0.731 | 0.334 | 0.415 | 0.688 | 0.043 | 50 0.005 |
| M0027 | full | exp08 | 1024bit-ECFP4 | SVM | 0.716 | 0.320 | 0.438 | 0.677 | 0.039 | 100000.0 0.01 |
| M0028 | full | exp09 | 1024bit-ECFP4 | SVM | 0.693 | 0.351 | 0.447 | 0.799 | 0.106 | 10 0.01 |
| M0029 | full | exp10 | 1024bit-ECFP4 | SVM | 0.823 | 0.222 | 0.388 | 0.764 | 0.059 | 100000.0 0.01 |
| M0030 | full | exp11 | 1024bit-ECFP4 | SVM | 0.816 | 0.233 | 0.361 | 0.681 | 0.135 | 10 0.01 |
| M0031 | full | exp12 | 1024bit-ECFP4 | SVM | 0.836 | 0.200 | 0.332 | 0.749 | 0.087 | 500 0.01 |
| M0032 | full | exp13 | 1024bit-ECFP4 | SVM | 0.736 | 0.361 | 0.430 | 0.724 | 0.012 | 10 0.01 |
| M0033 | full | exp14 | 1024bit-ECFP4 | SVM | 0.641 | 0.437 | 0.425 | 0.698 | 0.057 | 10 0.01 |
| M0034 | full | exp15 | 1024bit-ECFP4 | SVM | 0.724 | 0.315 | 0.423 | 0.771 | 0.047 | 10 0.01 |
| M0035 | full | exp16 | 1024bit-ECFP4 | SVM | 0.666 | 0.375 | 0.469 | 0.730 | 0.064 | 10 0.01 |
| M0036 | full | exp17 | 1024bit-ECFP4 | SVM | 0.767 | 0.265 | 0.395 | 0.723 | 0.044 | 50 0.01 |
| M0037 | full | exp18 | 1024bit-ECFP4 | SVM | 0.773 | 0.278 | 0.408 | 0.784 | 0.011 | 10 0.01 |
| M0038 | full | exp19 | 1024bit-ECFP4 | SVM | 0.745 | 0.288 | 0.412 | 0.762 | 0.017 | 10 0.01 |
| M0039 | full | exp01 | 1024bit-ECFP4 | GBM | 0.665 | 0.376 | 0.484 | 0.644 | 0.021 | 50 0.4 |
| M0040 | full | exp02 | 1024bit-ECFP4 | GBM | 0.754 | 0.304 | 0.419 | 0.652 | 0.103 | 50 0.3 |
| M0041 | full | exp03 | 1024bit-ECFP4 | GBM | 0.717 | 0.321 | 0.420 | 0.573 | 0.144 | 600 0.6 |
| M0042 | full | exp04 | 1024bit-ECFP4 | GBM | 0.677 | 0.383 | 0.467 | 0.641 | 0.036 | 300 0.1 |
| M0043 | full | exp05 | 1024bit-ECFP4 | GBM | 0.691 | 0.370 | 0.428 | 0.660 | 0.031 | 400 0.1 |
| M0044 | full | exp06 | 1024bit-ECFP4 | GBM | 0.716 | 0.320 | 0.427 | 0.603 | 0.113 | 800 0.1 |
| M0045 | full | exp07 | 1024bit-ECFP4 | GBM | 0.625 | 0.458 | 0.441 | 0.491 | 0.134 | 800 0.1 |
| M0046 | full | exp08 | 1024bit-ECFP4 | GBM | 0.608 | 0.441 | 0.502 | 0.573 | 0.035 | 200 0.1 |
| M0047 | full | exp09 | 1024bit-ECFP4 | GBM | 0.561 | 0.496 | 0.538 | 0.686 | 0.125 | 200 0.2 |
| M0048 | full | exp10 | 1024bit-ECFP4 | GBM | 0.700 | 0.362 | 0.457 | 0.679 | 0.021 | 600 0.1 |
| M0049 | full | exp11 | 1024bit-ECFP4 | GBM | 0.734 | 0.316 | 0.432 | 0.586 | 0.148 | 900 0.1 |
| M0050 | full | exp12 | 1024bit-ECFP4 | GBM | 0.766 | 0.284 | 0.413 | 0.720 | 0.046 | 500 0.1 |
| M0051 | full | exp13 | 1024bit-ECFP4 | GBM | 0.584 | 0.539 | 0.497 | 0.577 | 0.007 | 100 0.2 |
| M0052 | full | exp14 | 1024bit-ECFP4 | GBM | 0.501 | 0.630 | 0.457 | 0.469 | 0.032 | 50 0.3 |
| M0053 | full | exp15 | 1024bit-ECFP4 | GBM | 0.603 | 0.449 | 0.488 | 0.699 | 0.096 | 100 0.2 |
| M0054 | full | exp16 | 1024bit-ECFP4 | GBM | 0.561 | 0.503 | 0.531 | 0.633 | 0.072 | 50 0.2 |
| M0055 | full | exp17 | 1024bit-ECFP4 | GBM | 0.740 | 0.296 | 0.438 | 0.634 | 0.106 | 300 0.1 |
| M0056 | full | exp18 | 1024bit-ECFP4 | GBM | 0.729 | 0.330 | 0.443 | 0.676 | 0.053 | 200 0.1 |
| M0057 | full | exp19 | 1024bit-ECFP4 | GBM | 0.644 | 0.401 | 0.490 | 0.687 | 0.043 | 100 0.2 |
| M0058 | full | exp01 | 1024bit-ECFP4 | RF | 0.667 | 0.404 | 0.485 | 0.669 | 0.002 | 600 |
| M0059 | full | exp02 | 1024bit-ECFP4 | RF | 0.767 | 0.322 | 0.411 | 0.646 | 0.121 | 700 |
| M0060 | full | exp03 | 1024bit-ECFP4 | RF | 0.727 | 0.348 | 0.439 | 0.563 | 0.164 | 10 |
| M0061 | full | exp04 | 1024bit-ECFP4 | RF | 0.648 | 0.439 | 0.506 | 0.628 | 0.020 | 100 |
| M0062 | full | exp05 | 1024bit-ECFP4 | RF | 0.699 | 0.415 | 0.444 | 0.648 | 0.051 | 600 |
| M0063 | full | exp06 | 1024bit-ECFP4 | RF | 0.695 | 0.362 | 0.442 | 0.588 | 0.107 | 600 |
| M0064 | full | exp07 | 1024bit-ECFP4 | RF | 0.626 | 0.472 | 0.460 | 0.510 | 0.116 | 10 |
| M0065 | full | exp08 | 1024bit-ECFP4 | RF | 0.649 | 0.406 | 0.475 | 0.589 | 0.061 | 700 |
| M0066 | full | exp09 | 1024bit-ECFP4 | RF | 0.576 | 0.481 | 0.522 | 0.675 | 0.099 | 700 |
| M0067 | full | exp10 | 1024bit-ECFP4 | RF | 0.680 | 0.424 | 0.487 | 0.646 | 0.034 | 600 |
| M0068 | full | exp11 | 1024bit-ECFP4 | RF | 0.690 | 0.386 | 0.479 | 0.598 | 0.092 | 100 |
| M0069 | full | exp12 | 1024bit-ECFP4 | RF | 0.764 | 0.346 | 0.445 | 0.665 | 0.099 | 900 |
| M0070 | full | exp13 | 1024bit-ECFP4 | RF | 0.576 | 0.566 | 0.485 | 0.612 | 0.036 | 1000 |
| M0071 | full | exp14 | 1024bit-ECFP4 | RF | 0.514 | 0.610 | 0.465 | 0.534 | 0.020 | 300 |
| M0072 | full | exp15 | 1024bit-ECFP4 | RF | 0.642 | 0.432 | 0.478 | 0.661 | 0.019 | 1000 |
| M0073 | full | exp16 | 1024bit-ECFP4 | RF | 0.515 | 0.554 | 0.546 | 0.585 | 0.070 | 1000 |
| M0074 | full | exp17 | 1024bit-ECFP4 | RF | 0.699 | 0.341 | 0.454 | 0.640 | 0.059 | 10 |
| M0075 | full | exp18 | 1024bit-ECFP4 | RF | 0.679 | 0.387 | 0.469 | 0.732 | 0.053 | 50 |
| M0076 | full | exp19 | 1024bit-ECFP4 | RF | 0.625 | 0.429 | 0.495 | 0.660 | 0.035 | 1000 |
| M0077 | full | exp01 | 1024bit-ECFP4 | DNN2 | 0.747 | 0.618 | 0.595 | 0.726 | 0.021 | 600 120 0.2 |
| M0078 | full | exp02 | 1024bit-ECFP4 | DNN2 | 0.753 | 0.354 | 0.448 | 0.748 | 0.005 | 500 160 0.1 |
| M0079 | full | exp03 | 1024bit-ECFP4 | DNN2 | 0.777 | 0.392 | 0.456 | 0.702 | 0.075 | 400 120 0.1 |
| M0080 | full | exp01 | 1024bit-ECFP4 | DNN3 | 0.743 | 1.379 | 1.004 | 0.712 | 0.031 | 300 120 0.2 |
| M0081 | full | exp02 | 1024bit-ECFP4 | DNN3 | 0.769 | 0.720 | 0.667 | 0.757 | 0.012 | 600 120 0.1 |
| M0082 | full | exp03 | 1024bit-ECFP4 | DNN3 | 0.778 | 1.262 | 0.963 | 0.699 | 0.079 | 500 160 0.2 |
| M0083 | full | exp01 | 2048bit-ECFP4 | KNN | 0.581 | 0.522 | 0.498 | 0.546 | 0.035 | 1 uniform |
| M0084 | full | exp02 | 2048bit-ECFP4 | KNN | 0.697 | 0.364 | 0.430 | 0.608 | 0.089 | 1 uniform |
| M0085 | full | exp03 | 2048bit-ECFP4 | KNN | 0.625 | 0.442 | 0.458 | 0.510 | 0.115 | 3 distance |
| M0086 | full | exp04 | 2048bit-ECFP4 | KNN | 0.603 | 0.531 | 0.493 | 0.545 | 0.058 | 1 distance |
| M0087 | full | exp05 | 2048bit-ECFP4 | KNN | 0.743 | 0.302 | 0.400 | 0.542 | 0.201 | 1 uniform |
| M0088 | full | exp06 | 2048bit-ECFP4 | KNN | 0.652 | 0.448 | 0.479 | 0.533 | 0.119 | 1 uniform |
| M0089 | full | exp07 | 2048bit-ECFP4 | KNN | 0.379 | 0.804 | 0.542 | 0.335 | 0.044 | 5 distance |
| M0090 | full | exp08 | 2048bit-ECFP4 | KNN | 0.478 | 0.669 | 0.540 | 0.400 | 0.078 | 3 distance |
| M0091 | full | exp09 | 2048bit-ECFP4 | KNN | 0.300 | 1.206 | 0.702 | 0.470 | 0.170 | 1 uniform |
| M0092 | full | exp10 | 2048bit-ECFP4 | KNN | 0.642 | 0.459 | 0.475 | 0.566 | 0.076 | 3 distance |
| M0093 | full | exp11 | 2048bit-ECFP4 | KNN | 0.521 | 0.568 | 0.489 | 0.547 | 0.026 | 3 distance |
| M0094 | full | exp12 | 2048bit-ECFP4 | KNN | 0.780 | 0.250 | 0.377 | 0.559 | 0.221 | 1 uniform |
| M0095 | full | exp13 | 2048bit-ECFP4 | KNN | 0.688 | 0.399 | 0.458 | 0.520 | 0.168 | 1 uniform |
| M0096 | full | exp14 | 2048bit-ECFP4 | KNN | 0.290 | 1.056 | 0.585 | 0.448 | 0.158 | 3 distance |
| M0097 | full | exp15 | 2048bit-ECFP4 | KNN | 0.436 | 0.749 | 0.530 | 0.656 | 0.220 | 1 distance |
| M0098 | full | exp16 | 2048bit-ECFP4 | KNN | 0.491 | 0.638 | 0.534 | 0.403 | 0.088 | 1 uniform |
| M0099 | full | exp17 | 2048bit-ECFP4 | KNN | 0.570 | 0.507 | 0.482 | 0.502 | 0.068 | 3 distance |
| M0100 | full | exp18 | 2048bit-ECFP4 | KNN | 0.507 | 0.707 | 0.549 | 0.568 | 0.061 | 1 distance |
| M0101 | full | exp19 | 2048bit-ECFP4 | KNN | 0.493 | 0.720 | 0.519 | 0.614 | 0.121 | 1 distance |
| M0102 | full | exp01 | 2048bit-ECFP4 | SVM | 0.771 | 0.274 | 0.414 | 0.742 | 0.029 | 10 0.01 |
| M0103 | full | exp02 | 2048bit-ECFP4 | SVM | 0.794 | 0.265 | 0.393 | 0.761 | 0.033 | 10 0.01 |
| M0104 | full | exp03 | 2048bit-ECFP4 | SVM | 0.806 | 0.233 | 0.371 | 0.721 | 0.085 | 100 0.01 |
| M0105 | full | exp04 | 2048bit-ECFP4 | SVM | 0.777 | 0.281 | 0.412 | 0.751 | 0.026 | 10 0.01 |
| M0106 | full | exp05 | 2048bit-ECFP4 | SVM | 0.794 | 0.279 | 0.414 | 0.725 | 0.069 | 5 0.01 |
| M0107 | full | exp06 | 2048bit-ECFP4 | SVM | 0.771 | 0.258 | 0.371 | 0.726 | 0.045 | 1000.0 0.01 |
| M0108 | full | exp07 | 2048bit-ECFP4 | SVM | 0.709 | 0.364 | 0.408 | 0.682 | 0.027 | 10 0.01 |
| M0109 | full | exp08 | 2048bit-ECFP4 | SVM | 0.735 | 0.301 | 0.414 | 0.672 | 0.063 | 10 0.01 |
| M0110 | full | exp09 | 2048bit-ECFP4 | SVM | 0.693 | 0.351 | 0.446 | 0.790 | 0.097 | 10 0.01 |
| M0111 | full | exp10 | 2048bit-ECFP4 | SVM | 0.807 | 0.241 | 0.403 | 0.774 | 0.033 | 10 0.01 |
| M0112 | full | exp11 | 2048bit-ECFP4 | SVM | 0.802 | 0.249 | 0.371 | 0.685 | 0.117 | 10 0.01 |
| M0113 | full | exp12 | 2048bit-ECFP4 | SVM | 0.840 | 0.198 | 0.335 | 0.744 | 0.096 | 1000.0 0.01 |
| M0114 | full | exp13 | 2048bit-ECFP4 | SVM | 0.736 | 0.362 | 0.426 | 0.707 | 0.029 | 10 0.01 |
| M0115 | full | exp14 | 2048bit-ECFP4 | SVM | 0.628 | 0.454 | 0.426 | 0.694 | 0.066 | 5 0.01 |
| M0116 | full | exp15 | 2048bit-ECFP4 | SVM | 0.741 | 0.298 | 0.410 | 0.762 | 0.021 | 10 0.01 |
| M0117 | full | exp16 | 2048bit-ECFP4 | SVM | 0.698 | 0.342 | 0.444 | 0.717 | 0.019 | 10 0.01 |
| M0118 | full | exp17 | 2048bit-ECFP4 | SVM | 0.764 | 0.266 | 0.397 | 0.723 | 0.041 | 50 0.01 |
| M0119 | full | exp18 | 2048bit-ECFP4 | SVM | 0.762 | 0.284 | 0.408 | 0.788 | 0.026 | 100 0.01 |
| M0120 | full | exp19 | 2048bit-ECFP4 | SVM | 0.739 | 0.294 | 0.414 | 0.757 | 0.018 | 10 0.01 |
| M0121 | full | exp01 | 2048bit-ECFP4 | GBM | 0.689 | 0.390 | 0.502 | 0.640 | 0.049 | 50 0.2 |
| M0122 | full | exp02 | 2048bit-ECFP4 | GBM | 0.729 | 0.330 | 0.426 | 0.683 | 0.046 | 200 0.1 |
| M0123 | full | exp03 | 2048bit-ECFP4 | GBM | 0.723 | 0.326 | 0.420 | 0.602 | 0.121 | 200 0.3 |
| M0124 | full | exp04 | 2048bit-ECFP4 | GBM | 0.646 | 0.414 | 0.459 | 0.629 | 0.017 | 300 0.3 |
| M0125 | full | exp05 | 2048bit-ECFP4 | GBM | 0.729 | 0.337 | 0.424 | 0.646 | 0.083 | 500 0.1 |
| M0126 | full | exp06 | 2048bit-ECFP4 | GBM | 0.732 | 0.303 | 0.410 | 0.600 | 0.132 | 400 0.2 |
| M0127 | full | exp07 | 2048bit-ECFP4 | GBM | 0.627 | 0.455 | 0.451 | 0.527 | 0.100 | 900 0.2 |
| M0128 | full | exp08 | 2048bit-ECFP4 | GBM | 0.649 | 0.406 | 0.477 | 0.584 | 0.065 | 1000 0.1 |
| M0129 | full | exp09 | 2048bit-ECFP4 | GBM | 0.621 | 0.423 | 0.473 | 0.672 | 0.051 | 300 0.2 |
| M0130 | full | exp10 | 2048bit-ECFP4 | GBM | 0.722 | 0.335 | 0.450 | 0.663 | 0.059 | 800 0.2 |
| M0131 | full | exp11 | 2048bit-ECFP4 | GBM | 0.736 | 0.307 | 0.419 | 0.620 | 0.116 | 1000 0.2 |
| M0132 | full | exp12 | 2048bit-ECFP4 | GBM | 0.753 | 0.307 | 0.427 | 0.716 | 0.037 | 100 0.4 |
| M0133 | full | exp13 | 2048bit-ECFP4 | GBM | 0.616 | 0.489 | 0.467 | 0.581 | 0.035 | 400 0.1 |
| M0134 | full | exp14 | 2048bit-ECFP4 | GBM | 0.585 | 0.525 | 0.459 | 0.522 | 0.063 | 100 0.3 |
| M0135 | full | exp15 | 2048bit-ECFP4 | GBM | 0.624 | 0.423 | 0.476 | 0.696 | 0.072 | 200 0.1 |
| M0136 | full | exp16 | 2048bit-ECFP4 | GBM | 0.609 | 0.460 | 0.523 | 0.597 | 0.012 | 50 0.2 |
| M0137 | full | exp17 | 2048bit-ECFP4 | GBM | 0.768 | 0.257 | 0.390 | 0.624 | 0.144 | 200 0.4 |
| M0138 | full | exp18 | 2048bit-ECFP4 | GBM | 0.698 | 0.345 | 0.453 | 0.720 | 0.022 | 100 0.4 |
| M0139 | full | exp19 | 2048bit-ECFP4 | GBM | 0.678 | 0.360 | 0.464 | 0.664 | 0.014 | 1000 0.1 |
| M0140 | full | exp01 | 2048bit-ECFP4 | RF | 0.681 | 0.388 | 0.481 | 0.689 | 0.008 | 400 |
| M0141 | full | exp02 | 2048bit-ECFP4 | RF | 0.746 | 0.334 | 0.420 | 0.676 | 0.070 | 900 |
| M0142 | full | exp03 | 2048bit-ECFP4 | RF | 0.698 | 0.386 | 0.442 | 0.580 | 0.118 | 1000 |
| M0143 | full | exp04 | 2048bit-ECFP4 | RF | 0.651 | 0.436 | 0.492 | 0.627 | 0.024 | 900 |
| M0144 | full | exp05 | 2048bit-ECFP4 | RF | 0.745 | 0.372 | 0.439 | 0.620 | 0.125 | 700 |
| M0145 | full | exp06 | 2048bit-ECFP4 | RF | 0.707 | 0.343 | 0.440 | 0.576 | 0.131 | 700 |
| M0146 | full | exp07 | 2048bit-ECFP4 | RF | 0.589 | 0.514 | 0.467 | 0.528 | 0.061 | 50 |
| M0147 | full | exp08 | 2048bit-ECFP4 | RF | 0.638 | 0.410 | 0.465 | 0.593 | 0.045 | 700 |
| M0148 | full | exp09 | 2048bit-ECFP4 | RF | 0.560 | 0.496 | 0.524 | 0.679 | 0.119 | 500 |
| M0149 | full | exp10 | 2048bit-ECFP4 | RF | 0.639 | 0.457 | 0.497 | 0.633 | 0.006 | 500 |
| M0150 | full | exp11 | 2048bit-ECFP4 | RF | 0.685 | 0.394 | 0.477 | 0.598 | 0.087 | 50 |
| M0151 | full | exp12 | 2048bit-ECFP4 | RF | 0.750 | 0.361 | 0.456 | 0.680 | 0.070 | 800 |
| M0152 | full | exp13 | 2048bit-ECFP4 | RF | 0.610 | 0.527 | 0.469 | 0.589 | 0.021 | 300 |
| M0153 | full | exp14 | 2048bit-ECFP4 | RF | 0.566 | 0.551 | 0.450 | 0.573 | 0.007 | 50 |
| M0154 | full | exp15 | 2048bit-ECFP4 | RF | 0.659 | 0.414 | 0.461 | 0.664 | 0.005 | 700 |
| M0155 | full | exp16 | 2048bit-ECFP4 | RF | 0.527 | 0.531 | 0.533 | 0.613 | 0.086 | 10 |
| M0156 | full | exp17 | 2048bit-ECFP4 | RF | 0.701 | 0.346 | 0.466 | 0.659 | 0.042 | 100 |
| M0157 | full | exp18 | 2048bit-ECFP4 | RF | 0.676 | 0.383 | 0.464 | 0.735 | 0.059 | 200 |
| M0158 | full | exp19 | 2048bit-ECFP4 | RF | 0.643 | 0.412 | 0.474 | 0.657 | 0.014 | 800 |
| M0159 | full | exp01 | 2048bit-ECFP4 | DNN2 | 0.715 | 0.792 | 0.742 | 0.761 | 0.046 | 600 140 0.2 |
| M0160 | full | exp02 | 2048bit-ECFP4 | DNN2 | 0.797 | 0.347 | 0.452 | 0.774 | 0.023 | 400 120 0.1 |
| M0161 | full | exp03 | 2048bit-ECFP4 | DNN2 | 0.835 | 0.388 | 0.477 | 0.738 | 0.097 | 300 80 0.1 |
| M0162 | full | exp01 | 2048bit-ECFP4 | DNN3 | 0.702 | 0.496 | 0.554 | 0.756 | 0.054 | 200 60 0.1 |
| M0163 | full | exp02 | 2048bit-ECFP4 | DNN3 | 0.787 | 0.390 | 0.485 | 0.769 | 0.018 | 100 140 0.1 |
| M0164 | full | exp03 | 2048bit-ECFP4 | DNN3 | 0.805 | 1.115 | 0.907 | 0.726 | 0.079 | 500 160 0.2 |
| M0165 | full | exp01 | 1024bit-ECFP6 | KNN | 0.560 | 0.558 | 0.510 | 0.475 | 0.085 | 1 distance |
| M0166 | full | exp02 | 1024bit-ECFP6 | KNN | 0.657 | 0.433 | 0.465 | 0.542 | 0.115 | 1 uniform |
| M0167 | full | exp03 | 1024bit-ECFP6 | KNN | 0.670 | 0.362 | 0.449 | 0.470 | 0.201 | 1 uniform |
| M0168 | full | exp04 | 1024bit-ECFP6 | KNN | 0.580 | 0.550 | 0.507 | 0.534 | 0.046 | 1 distance |
| M0169 | full | exp05 | 1024bit-ECFP6 | KNN | 0.534 | 0.566 | 0.475 | 0.551 | 0.017 | 1 distance |
| M0170 | full | exp06 | 1024bit-ECFP6 | KNN | 0.686 | 0.396 | 0.450 | 0.500 | 0.186 | 1 distance |
| M0171 | full | exp07 | 1024bit-ECFP6 | KNN | 0.322 | 0.897 | 0.567 | 0.265 | 0.057 | 5 distance |
| M0172 | full | exp08 | 1024bit-ECFP6 | KNN | 0.424 | 0.756 | 0.572 | 0.357 | 0.067 | 3 distance |
| M0173 | full | exp09 | 1024bit-ECFP6 | KNN | 0.243 | 1.282 | 0.739 | 0.454 | 0.211 | 1 uniform |
| M0174 | full | exp10 | 1024bit-ECFP6 | KNN | 0.776 | 0.298 | 0.421 | 0.532 | 0.244 | 1 distance |
| M0175 | full | exp11 | 1024bit-ECFP6 | KNN | 0.525 | 0.599 | 0.505 | 0.478 | 0.047 | 1 uniform |
| M0176 | full | exp12 | 1024bit-ECFP6 | KNN | 0.681 | 0.362 | 0.415 | 0.536 | 0.145 | 1 uniform |
| M0177 | full | exp13 | 1024bit-ECFP6 | KNN | 0.329 | 0.874 | 0.608 | 0.439 | 0.110 | 5 distance |
| M0178 | full | exp14 | 1024bit-ECFP6 | KNN | 0.221 | 1.182 | 0.633 | 0.381 | 0.160 | 5 distance |
| M0179 | full | exp15 | 1024bit-ECFP6 | KNN | 0.439 | 0.757 | 0.535 | 0.586 | 0.147 | 1 uniform |
| M0180 | full | exp16 | 1024bit-ECFP6 | KNN | 0.530 | 0.578 | 0.491 | 0.361 | 0.169 | 1 uniform |
| M0181 | full | exp17 | 1024bit-ECFP6 | KNN | 0.619 | 0.463 | 0.447 | 0.471 | 0.148 | 1 uniform |
| M0182 | full | exp18 | 1024bit-ECFP6 | KNN | 0.503 | 0.719 | 0.551 | 0.558 | 0.055 | 1 uniform |
| M0183 | full | exp19 | 1024bit-ECFP6 | KNN | 0.442 | 0.784 | 0.521 | 0.602 | 0.160 | 1 uniform |
| M0184 | full | exp01 | 1024bit-ECFP6 | SVM | 0.741 | 0.328 | 0.446 | 0.705 | 0.036 | 5 0.01 |
| M0185 | full | exp02 | 1024bit-ECFP6 | SVM | 0.756 | 0.332 | 0.435 | 0.746 | 0.010 | 5 0.01 |
| M0186 | full | exp03 | 1024bit-ECFP6 | SVM | 0.796 | 0.256 | 0.382 | 0.690 | 0.106 | 5 0.01 |
| M0187 | full | exp04 | 1024bit-ECFP6 | SVM | 0.713 | 0.361 | 0.463 | 0.727 | 0.014 | 5 0.01 |
| M0188 | full | exp05 | 1024bit-ECFP6 | SVM | 0.793 | 0.276 | 0.403 | 0.704 | 0.089 | 10 0.005 |
| M0189 | full | exp06 | 1024bit-ECFP6 | SVM | 0.755 | 0.283 | 0.404 | 0.663 | 0.092 | 5 0.01 |
| M0190 | full | exp07 | 1024bit-ECFP6 | SVM | 0.678 | 0.408 | 0.445 | 0.632 | 0.046 | 5 0.01 |
| M0191 | full | exp08 | 1024bit-ECFP6 | SVM | 0.700 | 0.343 | 0.458 | 0.660 | 0.040 | 5 0.01 |
| M0192 | full | exp09 | 1024bit-ECFP6 | SVM | 0.672 | 0.380 | 0.474 | 0.766 | 0.094 | 10 0.005 |
| M0193 | full | exp10 | 1024bit-ECFP6 | SVM | 0.789 | 0.285 | 0.428 | 0.732 | 0.057 | 5 0.01 |
| M0194 | full | exp11 | 1024bit-ECFP6 | SVM | 0.769 | 0.292 | 0.415 | 0.661 | 0.108 | 5 0.01 |
| M0195 | full | exp12 | 1024bit-ECFP6 | SVM | 0.806 | 0.248 | 0.365 | 0.714 | 0.092 | 50 0.01 |
| M0196 | full | exp13 | 1024bit-ECFP6 | SVM | 0.721 | 0.396 | 0.450 | 0.702 | 0.019 | 10 0.005 |
| M0197 | full | exp14 | 1024bit-ECFP6 | SVM | 0.614 | 0.464 | 0.452 | 0.677 | 0.063 | 10 0.005 |
| M0198 | full | exp15 | 1024bit-ECFP6 | SVM | 0.675 | 0.375 | 0.447 | 0.731 | 0.056 | 5 0.01 |
| M0199 | full | exp16 | 1024bit-ECFP6 | SVM | 0.661 | 0.384 | 0.467 | 0.707 | 0.046 | 10 0.005 |
| M0200 | full | exp17 | 1024bit-ECFP6 | SVM | 0.732 | 0.301 | 0.433 | 0.685 | 0.047 | 10 0.005 |
| M0201 | full | exp18 | 1024bit-ECFP6 | SVM | 0.731 | 0.327 | 0.420 | 0.757 | 0.026 | 5 0.01 |
| M0202 | full | exp19 | 1024bit-ECFP6 | SVM | 0.706 | 0.335 | 0.427 | 0.734 | 0.028 | 50 0.01 |
| M0203 | full | exp01 | 1024bit-ECFP6 | GBM | 0.560 | 0.522 | 0.544 | 0.655 | 0.095 | 100 0.1 |
| M0204 | full | exp02 | 1024bit-ECFP6 | GBM | 0.656 | 0.431 | 0.477 | 0.622 | 0.034 | 200 0.1 |
| M0205 | full | exp03 | 1024bit-ECFP6 | GBM | 0.669 | 0.382 | 0.463 | 0.566 | 0.103 | 200 0.2 |
| M0206 | full | exp04 | 1024bit-ECFP6 | GBM | 0.518 | 0.556 | 0.561 | 0.610 | 0.092 | 50 0.2 |
| M0207 | full | exp05 | 1024bit-ECFP6 | GBM | 0.656 | 0.432 | 0.485 | 0.587 | 0.069 | 100 0.2 |
| M0208 | full | exp06 | 1024bit-ECFP6 | GBM | 0.676 | 0.366 | 0.460 | 0.499 | 0.177 | 600 0.3 |
| M0209 | full | exp07 | 1024bit-ECFP6 | GBM | 0.545 | 0.554 | 0.481 | 0.468 | 0.077 | 400 0.1 |
| M0210 | full | exp08 | 1024bit-ECFP6 | GBM | 0.588 | 0.471 | 0.504 | 0.519 | 0.069 | 900 0.1 |
| M0211 | full | exp09 | 1024bit-ECFP6 | GBM | 0.543 | 0.530 | 0.549 | 0.629 | 0.086 | 1000 0.2 |
| M0212 | full | exp10 | 1024bit-ECFP6 | GBM | 0.650 | 0.428 | 0.504 | 0.614 | 0.036 | 200 0.2 |
| M0213 | full | exp11 | 1024bit-ECFP6 | GBM | 0.614 | 0.434 | 0.479 | 0.564 | 0.050 | 400 0.6 |
| M0214 | full | exp12 | 1024bit-ECFP6 | GBM | 0.676 | 0.410 | 0.508 | 0.656 | 0.020 | 100 0.1 |
| M0215 | full | exp13 | 1024bit-ECFP6 | GBM | 0.548 | 0.579 | 0.512 | 0.555 | 0.007 | 300 0.1 |
| M0216 | full | exp14 | 1024bit-ECFP6 | GBM | 0.441 | 0.693 | 0.524 | 0.482 | 0.041 | 50 0.5 |
| M0217 | full | exp15 | 1024bit-ECFP6 | GBM | 0.569 | 0.483 | 0.496 | 0.653 | 0.084 | 300 0.1 |
| M0218 | full | exp16 | 1024bit-ECFP6 | GBM | 0.544 | 0.513 | 0.538 | 0.528 | 0.016 | 50 0.4 |
| M0219 | full | exp17 | 1024bit-ECFP6 | GBM | 0.657 | 0.394 | 0.507 | 0.581 | 0.077 | 50 0.2 |
| M0220 | full | exp18 | 1024bit-ECFP6 | GBM | 0.624 | 0.428 | 0.486 | 0.641 | 0.017 | 100 0.2 |
| M0221 | full | exp19 | 1024bit-ECFP6 | GBM | 0.648 | 0.399 | 0.486 | 0.663 | 0.015 | 200 0.1 |
| M0222 | full | exp01 | 1024bit-ECFP6 | RF | 0.631 | 0.446 | 0.513 | 0.655 | 0.024 | 100 |
| M0223 | full | exp02 | 1024bit-ECFP6 | RF | 0.720 | 0.391 | 0.454 | 0.632 | 0.088 | 700 |
| M0224 | full | exp03 | 1024bit-ECFP6 | RF | 0.659 | 0.429 | 0.483 | 0.530 | 0.130 | 1000 |
| M0225 | full | exp04 | 1024bit-ECFP6 | RF | 0.534 | 0.546 | 0.554 | 0.594 | 0.060 | 1000 |
| M0226 | full | exp05 | 1024bit-ECFP6 | RF | 0.642 | 0.470 | 0.484 | 0.592 | 0.050 | 600 |
| M0227 | full | exp06 | 1024bit-ECFP6 | RF | 0.656 | 0.403 | 0.474 | 0.506 | 0.151 | 400 |
| M0228 | full | exp07 | 1024bit-ECFP6 | RF | 0.547 | 0.566 | 0.493 | 0.453 | 0.094 | 200 |
| M0229 | full | exp08 | 1024bit-ECFP6 | RF | 0.629 | 0.432 | 0.494 | 0.532 | 0.097 | 1000 |
| M0230 | full | exp09 | 1024bit-ECFP6 | RF | 0.554 | 0.509 | 0.528 | 0.593 | 0.039 | 1000 |
| M0231 | full | exp10 | 1024bit-ECFP6 | RF | 0.627 | 0.491 | 0.519 | 0.617 | 0.010 | 1000 |
| M0232 | full | exp11 | 1024bit-ECFP6 | RF | 0.652 | 0.421 | 0.488 | 0.568 | 0.084 | 100 |
| M0233 | full | exp12 | 1024bit-ECFP6 | RF | 0.705 | 0.400 | 0.485 | 0.646 | 0.059 | 800 |
| M0234 | full | exp13 | 1024bit-ECFP6 | RF | 0.509 | 0.641 | 0.531 | 0.576 | 0.067 | 1000 |
| M0235 | full | exp14 | 1024bit-ECFP6 | RF | 0.480 | 0.648 | 0.492 | 0.486 | 0.006 | 300 |
| M0236 | full | exp15 | 1024bit-ECFP6 | RF | 0.579 | 0.494 | 0.500 | 0.629 | 0.050 | 800 |
| M0237 | full | exp16 | 1024bit-ECFP6 | RF | 0.421 | 0.654 | 0.609 | 0.540 | 0.119 | 50 |
| M0238 | full | exp17 | 1024bit-ECFP6 | RF | 0.664 | 0.386 | 0.496 | 0.601 | 0.063 | 1000 |
| M0239 | full | exp18 | 1024bit-ECFP6 | RF | 0.649 | 0.427 | 0.490 | 0.703 | 0.054 | 50 |
| M0240 | full | exp19 | 1024bit-ECFP6 | RF | 0.596 | 0.467 | 0.509 | 0.660 | 0.064 | 100 |
[truncated: 249,685 more chars]
